# ‘First 1000 days’ health interventions in low- and middle-income countries: alignment of South African policies with high-quality evidence

**DOI:** 10.1080/16549716.2017.1340396

**Published:** 2017-07-18

**Authors:** René English, Nazia Peer, Simone Honikman, Aviva Tugendhaft, Karen J Hofman

**Affiliations:** ^a^ Health Systems Trust, Health Systems Research Unit, Cape Town, South Africa; ^b^ School of Public Health and Family Medicine, University of Cape Town, Cape Town, South Africa; ^c^ Perinatal Maternal Mental Health, The Alan J Flisher Centre for Public Mental Health, University of Cape Town, Cape Town, South Africa; ^d^ Priority Cost Effective Lessons for System Strengthening South Africa, School of Public Health, University of the Witwatersrand, Johannesburg, South Africa

**Keywords:** First 1000 days, maternal, neonatal, child health, South Africa, interventions

## Abstract

**Background**: In South Africa (SA), despite adoption of international strategies and approaches, maternal, neonatal and child (MNC) morbidity and mortality rates have not sufficiently declined.

**Objectives**: To conduct an umbrella review (UR) that identifies interventions in low- and middle-income countries, with a high-quality evidence base, that improve MNC morbidity and mortality outcomes within the first 1000 days of life; and to assess the incorporation of the evidence into local strategies, guidelines and documents.

**Methods**: We included publications about women and children in the first 1000 days of life; healthcare professionals and community members. Comparators were those who did not receive the intervention. Interventions were pharmacological and non-pharmacological. Outcomes were MNC morbidity and mortality. Authors conducted English language electronic and manual searches (2000–2013). The quality of systematic reviews and meta-analyses (SRs/MAs) were reviewed. Interventions were ranked according to level of evidence; and then aligned with SA strategies, policies and guidelines. A tool to extract data was developed and used by two authors who independently extracted data. Summary measures from MAs or summaries of SRs were reviewed and the specificities of the various interventions listed. A search of all local high-level documents was done and these were assessed to determine the specificities of the recommendations and their alignment to the evidence.

**Results**: In total, 19 interventions presented in 32 SRs were identified. Overall, SA’s policymakers have sufficiently included high-quality evidence-based interventions into local policies. However, optimal period of birth spacing (two to five years) is not explicitly promoted nor was ante- and postnatal depression adequately incorporated. Antenatal care visits should be increased from four to about eight according to the evidence.

**Conclusion**: Incorporation of existing evidence into policies can be strengthened in SA. The UR methods are useful to inform policymaking and identify research gaps.

**RESPONSIBLE EDITOR** Nawi Ng, Umeå University, Sweden

## Background

Over the past decade, South Africa (SA) has made progress towards improving maternal, newborn and child (MNC) mortality and morbidity outcomes.[1] High-level endorsement and adoption of evidence-based MNC strategies and approaches have been promoted nationally and numerous high-impact priority cost-effective interventions have been incorporated into policies and guidelines.[2] The ‘first 1000 days’ (from conception to a child’s second birthday) has been an area of focus, as a period shown to have the greatest potential for positive impact on long-term health outcomes, for both mother and child.[3] Successful initiatives such as the up-scaling of the antiretroviral treatment (ART) programme has seen more HIV-infected pregnant women and children initiated on ART;[4,5] the implementation of the Prevention of Mother-to-Child Transmission (PMTCT) programme has resulted in a dramatic reduction in the national mother-to-child HIV transmission rate;[6] and the introduction of the pneumococcal and rotavirus vaccines in 2009 has been associated with declines in child pneumonia and diarrhoeal rates respectively. Despite these and other accomplishments, SA did not achieve the MNC health Millennium Development Goals.[7,8] Many factors may contribute to this observation, including the quality of and heterogeneity of care among the 52 districts,[8] and various contextual (burden of disease, geography and rurality, lack of resources) and upstream factors (local policies, political bias and buy-in), including social determinants of health. Another reason could be attributed to the degree of incorporation of the specifics of the evidence base underpinning MNC interventions into local policies, strategies and guidelines.[1,2,9–17] It is therefore of value for decision makers to constantly review the strength of the existing evidence base and its applicability to the local setting, and how well the evidence is translated into written local documents, and to identify some of the ‘know-do’ gaps.

This review presents an overview of recent systematic reviews (SRs) and meta-analyses (MAs) that report on effective clinical and public health interventions targeting various aspects or levels of care in the first 1000 days of life ‒ with a specific focus on low-and middle-income countries (LMIC) and primary health care (PHC) ‒ and on the outcomes of these on MNC mortality *and* morbidity in the first 1000 day period.

## Methods

Umbrella reviews summarise and present findings of existing SRs and MAs,[18] and are promoted by the Joanna Briggs Institute (JBI).[19,20] The methodology is similar to that proposed by the Cochrane Collaboration for synthesising results from multiple SRs.[21] These reviews aim to provide relevant evidence to healthcare decision-makers, thereby ‘allowing the findings of separate reviews to be compared and, thus multiple syntheses of existing review data’ [18, p. 103]. It does not aim to reproduce the methods employed for appraisal of primary studies; instead, it provides ‘an overall picture of findings for particular questions’.

## Protocol and registration

Given the methodology employed there was no need to register the protocol. Nonetheless, the authors aimed to adhere to the PRISMA [22] and JBI group’s [19] reporting guidelines.

## Eligibility criteria

### Study characteristics

This review only includes English-language, peer-reviewed SRs and MAs that focused on MNCH interventions implemented in the first 1000 days of life window in LMIC and PHC settings; published between 2000 and 2013.

### Types of participants and comparators

Participants were women and children in the first 1000 days of life; trained and untrained healthcare professionals; and community members. Comparators were the same as the aforementioned, except without having received the intervention.

### Types of interventions

The interventions were either pharmacological (prevention and treatment) or non-pharmacological (behaviour and or approach).

### Context/setting

The context was LMIC settings and focused on PHC settings. However, we also included vulnerable populations, immigrants or minority groups in high-income countries (HICs); these would refer to racial or ethnic differences in the minority populations and the general population.

### Outcomes

MNC mortality and morbidity measured in the first 1000 days were the primary outcomes.

### Information sources

The authors conducted electronic searches in the Cochrane Database of SRs, the WHO/Reproductive Health Library (WHORHL), Cumulative Index to Nursing and Allied Health Literature (CINAHL), PubMed/MEDLINE database, Google Scholar and Scielo (Scientific Electronic Library Online). Where necessary, unpublished data and reports were requested from authors and experts, and from conference abstracts or other presentations. Manual searches through the references of articles and grey literature were also conducted; duplicates were removed; and all reports and policy documents were removed. The last search was done in January 2014.

### Study selection

**Figure 1.**
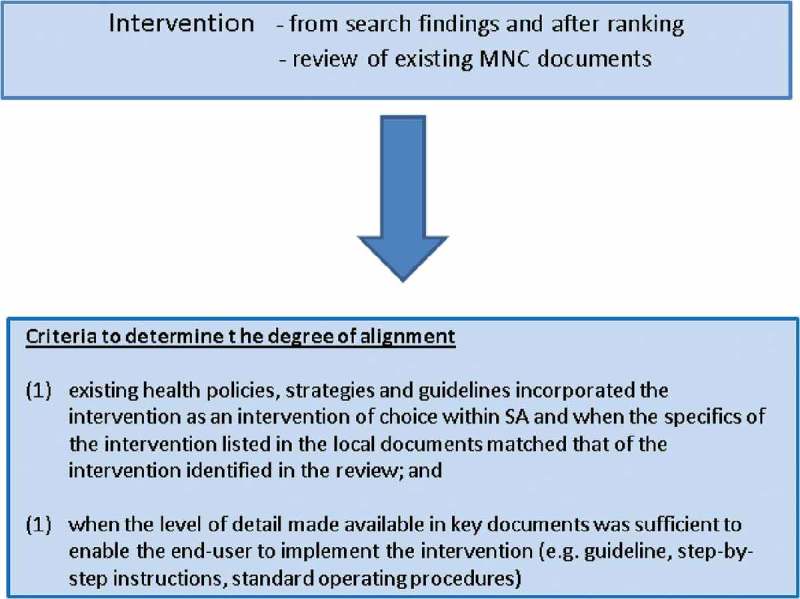
Criteria to determine alignment.

For the initial screening done by two reviewers (NP, JD), only the abstract and title were used (refer to Figure 1, marked B). Using a structured approach, they independently assessed the full texts of selected articles, and then independently examined the selected studies (not blinded to authors or journals) and applied the eligibility criteria for selecting studies (refer to Appendix 8 of Supplemental File). Reviewers discussed each other’s selections, and resolved disagreements through discussion, and consulted a third reviewer (MD) when no consensus was reached.

### Data collection and items

A data extraction sheet, developed and piloted by the study authors, contained the following headings: author and publication year; journal; study design; objectives; number and type of participants; intervention (description of the intervention, setting, who conducted it, training, etc.); summary measures; outcome(s) (mortality, morbidity).

### Risk of bias in individual studies

**Figure 2.**
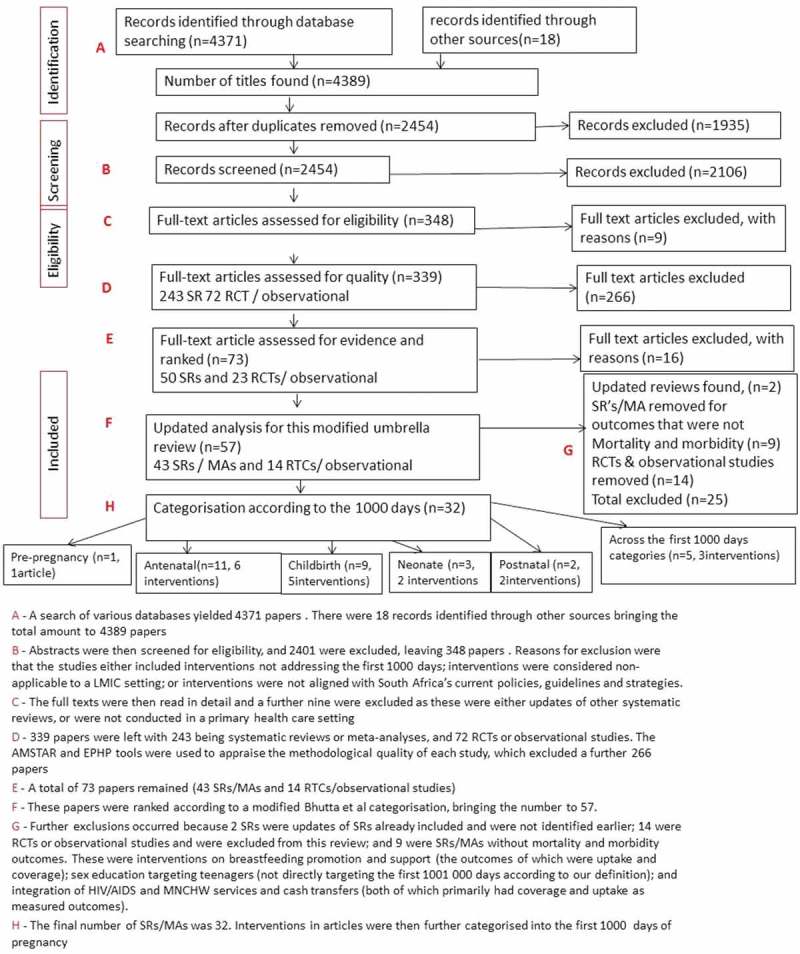
Methodology and results of the search strategy.

AMSTAR (A Measurement Tool to Assess SRs),[23] a quality-assessment tool, was used to grade the SRs and MAs (refer to Appendix 8 in Supplemental File and D in Figure 2).

### Ranking of evidence

All studies deemed of appropriate quality using the AMSTAR tool were then ranked into three categories of importance using a modified version of categories created by Bhutta et al [24] (Table 1), in which evidence-based interventions and the best delivery strategies for PHC in developing countries were identified (refer to Appendix 8 in Supplemental File and E in Figure 2).

**Table 1.** Categories used to rank evidence.

Score	Categories
Evidence of impact on morbidity or mortality^a^	
1	Strong evidence of benefit in efficacy and effectiveness settings and recommended for inclusion in health systems.
2	Moderate level of evidence of impact but recommended for inclusion in health systems.
3	Low level of evidence but recommended for further evaluation in health systems on additional criteria and plausibility.
Evidence and potential for delivery and inclusion in primary care programmes	
1	Strong evidence of benefit in efficacy and effectiveness settings and recommended.
2	Plausible and promising evidence of impact but needs evaluation in health systems at scale.
3	Promising interventions that need further assessment in primary care settings.
Evidence on determinants of child health and behaviour change (eg. fiscal, regulatory)	
1	Strong evidence of benefit in efficacy and effectiveness settings and recommended.
2	Plausible and promising evidence of impact but need more at scale.
3	Low or no evidence of scale with uncertainty if applicability.

^a^Categories are based on a ranking scale developed by Bhutta Z et al. [17] and modified for this review.

## Summary measures and data synthesis

For mortality and morbidity reduction, a relative risk, odds ratio, hazard ratio, or rate ratio were the primary measures of effect. For the continuous outcomes, a difference in means or medians was used. Outcome data from MAs in the SRs were extracted and compiled into a single extraction form (Appendix 2 in Supplemental File).

## Assessing alignment of evidence in local policies, strategies and guidelines

To determine the degree of incorporation of the evidence base into local written strategies, policies and guidelines, so as to gauge translation of knowledge, a search was done to identify all high-level official local MNC strategies, policies and guidelines. All SA national strategies, policies and guidelines released from the health ministry in the last 10 years were retrieved and reviewed; government websites were searched and knowledgeable experts and government officials were contacted directly. Searches were repeated in 2014 and 2015.

Details of how alignment was agreed upon are found in Appendix 8 in Supplemental File and Figure 1. Authors assessed whether these matched in terms of *what* should be done, and *how* it should be implemented.

## Results

### Selection of studies

A search of various databases yielded 4371 papers as presented in Figure 2. There were 18 records identified through other sources, bringing the total amount to 4389 papers (marked A in Figure 2). After adjusting for duplications, 1935 were discarded and a total of 2454 papers remained. The abstracts were then screened for eligibility, and 2401 were excluded, leaving 348 papers (marked B in Figure 2). The methodological quality of each study is marked ‘D’ in Figure 2. A total of 73 papers remained (43 SRs/MAs and 14 RTCs/observational studies) (marked E in Figure 2). Ranking according to a modified Bhutta et al. [24] categorisation, bringing the number to 57, marked F in Figure 2 (Table 1).

The final number of SRs/MAs was 32. Interventions in articles were then further categorised into the first 1000 days, namely pre-pregnancy, antenatal, childbirth, postnatal, and neonate (marked G in Figure 2; Table 2). A further category ‒ ‘across the first 1000 days categories’ ‒ was created for interventions targeting more than one category (marked H in Figure 2).

**Table 2.** Table of interventions according to the stages of pregnancy.

Stage	Intervention	Reference
Pre-pregnancy	Promotion of birth spacing of between 18 and 59 months	[30]
Antenatal	Promotion of a minimum of > or = 4 ANC visits	[33]
	Multiple micronutrient, balanced protein energy and folic acid supplementation	[19,36,37,38]
	Detection and treatment of maternal syphilis	[40]
	Prevention of hypertensive disorders (calcium supplementation/anti-platelets)	[41,42,43]
	Interpersonal psychotherapy to prevent and treat antenatal depression	[44]
	Prevention of mother-to-child transmission (PMTCT)	[46]
Childbirth	Antibiotic therapy for PROM or pPROM	[49,50]
	Corticosteroid administration at presentation of preterm labour	[18,53]
	Magnesium sulphate (IV/IM) versus phenytoin as part of managing eclampsia	[54]
	Supply of clean birth kits for deliveries	[55]
	Presence of birth attendants during delivery	[63,64,65]
Neonate	Promotion of KMC/skin-to-skin contact	[66,67]
	Vitamin A supplementation in term neonates within first 28 days of life	[68,71]
Postnatal	Antidepressants to prevent postnatal depression	[69]
	ARVs to treat HIV/AIDS in children <3 years	[70]
Across the first 1000 days categories	Community-level service delivery interventions	[73,74,75]
	Handwashing with soap or improved water quality to prevent diarrhoea	[78,79]

#### Study characteristics

##### Methods

All 32 SRs/MAs were published in English, of which 28 were SRs without MAs and four were SRs with MAs. The SRs were published between 2001 and 2013, with more than half having been published during or after 2010. The SR incorporated LMIC (*n* = 10), HIC (*n* = 2) or a combination of these (*n* = 20). Categorisation according to the first 1000 days yielded the following: pre-pregnancy (*n *= 1); antenatal (*n *= 11); childbirth (*n *= 9); neonate (*n *= 4); postnatal (*n *= 2); and ‘across the first 1000 days categories’ (*n *= 5).

##### Participants

Participant characteristics for each of the ‘first 1000 day’ categories are presented in Appendix 3 in Supplemental File. Participants were typically pregnant women or women of reproductive age; mothers; infants; children younger than 5 years; traditional birth attendants, community health workers, village health workers, community health aides, skilled birth attendants/skilled attendants, birthing centre practitioners or community midwives, lay counsellors or peer counsellors, nutrition workers, or home visitors.

##### Interventions

The interventions are presented in Appendix 4 in Supplemental File, and in total, 20 interventions (reported in 32 papers) were identified; broadly categorised into pharmacological and non-pharmacological interventions. Ten involved the administration of a supplement/food fortification or medication (oral, intravenous or intramuscular) (i.e. pharmacological), or the implementation of an approach (*n *= 4) with two involving capacitation of health or lay healthcare workers (*n *= 4) or provision of supplies or equipment to healthcare workers (*n *= 1).

##### Outcomes

Mortality and morbidity outcomes were reported in 23 studies. Six studies presented only morbidity outcomes, and three studies presented only mortality outcomes.

##### Methodological quality

The methodological quality of each study is presented in Appendix 6 in Supplemental File. There were 201 SRs or MAs that had reported on mortality and morbidity outcomes, and only these are discussed for the purposes of this modified umbrella review. Of these, 40 scored 11/11 using the AMSTAR tool (i.e. high quality). From the 11 questions, the majority of the SRs or MAs provided a design *a priori*, met the criteria for duplicate study selection and data extraction, performed a comprehensive literature search, presented study characteristics, and declared conflicts of interest. Many studies did not mention whether grey literature was searched, and although most had listed all the included studies, only some had listed excluded studies.

All the SRs or MAs assessed and documented the scientific quality of the included studies. For the question that asked whether the scientific quality of the included studies was used appropriately in formulating conclusions, not all the articles scored a ‘Yes’. Almost all of the studies used appropriate methods to combine the study findings. For the question that asked about assessing publication bias, not all of the studies had done this.

### Pre-pregnancy

#### Promotion of birth spacing (≥18 months – <5 years)

A MA [25] of 67 observational studies involving 11,091,659 pregnant women conducted in 2006 explored the association between birth spacing and the relative risk of adverse perinatal outcomes. Birth spacing was defined as the interval from one birth and the next consecutive birth, or the interval between delivery and a subsequent conception. The outcomes were measured at the following time points: >6 months, 6‒11 months, 12‒17 months, 18‒23 months, 24‒59 months, ≤60 months. Results show that inter-pregnancy intervals shorter than 18 months and longer than 59 months were associated with significantly increased risk of perinatal outcomes. Intervals shorter than six months were associated with increased risks of preterm birth (pooled adjusted odds ratio (OR) (95%CI):1.40 (1.24‒1.58)), low birth weight (OR 1.61 (1.39‒1.86)) and small for gestational age (SGA) (1.26 (1.18‒1.33)) compared to intervals of 18‒23 months. For intervals longer than 59 months, the risks were also increased (OR 1.20 (1.17‒1.24); OR 1.43 (1.27‒1.62); OR 1.29 (1.20‒1.39)), respectively, for the aforementioned outcomes. This trend was also observed for intervals of six to 17 months; there was not enough evidence to determine the effect on foetal or neonatal mortality, although some results suggested increased risk. Substantial statistical heterogeneity was observed for most of the meta-analyses which could affect the interpretation of the results. In summary, the promotion of birth spacing of periods (≥18 months – <5 years) was associated with reduced preterm births, low birth weight and SGA.

#### Local SA strategies, policies, guidelines and practices

SA promotes a range of contraceptive methods.[9,10] Mention is made of birth spacing and the importance thereof for infant and child health in the Health Minister’s Forewords in these documents, but the exact period of optimal birth spacing period is not promoted in the guidelines and therefore does not fully align with existing evidence.

### Antenatal

#### Promotion of a minimum of four antenatal care visits

A SR,[26] comprising seven trials (more than 60,000 women), compared the effects of antenatal care programmes that provide lower numbers of visits for low-risk women (i.e. between four and six visits ‒ each visit focusing on specific evidence-based goal-orientated activities in LMICs) compared with those providing more visits ‒ where what is considered to be ‘usual care’ is provided. In HIC, visits were reduced from an average of 13 or 14 visits to between six and nine visits. Reducing the number of antenatal visits, even when associated with more ‘goal-orientated care’ was associated with increased perinatal mortality in LMIC settings (RR 1.15 (1.01‒1.32), three trials). For other morbidity outcomes (pre-eclampsia, preterm births, vaginal bleeding, Caesarean section, induction of labour, and low birth weight), the differences were not significant. Overall, the reduction in the number of visits was associated with reduced admission to neonatal intensive care units admissions (RR 0.89 (0.79‒1.02)).

#### Local SA strategies, policies, guidelines and practices

At the time of conducting the review, SA promoted [11] basic antenatal care comprising four health facility visits for women with no risk factors (Supplemental File: Appendix 1), with the first visit occurring before 12 weeks’ gestation. Furthermore, community health workers (CHWs) are provided with algorithms and checklists for community-based antenatal and postnatal care;[15] at least four antenatal visits are promoted. Additional visits are required depending on the woman’s clinical condition, and detailed checklists are included in the guidelines. The above evidence however suggests that four ANC may not be sufficient and this requires further review in SA. Furthermore, the WHO has recently proposed raising the number of ANC to eight.[27] At the time of publishing this review, SA had changed the requisite number from four to eight, in line with international criteria.

#### Micronutrients and balanced protein/energy supplementation for improved foetal outcomes

Supplementation of micronutrients and protein energy malnutrition was another broad category of reviews identified. Four studies, each looking at a range of either maternal (e.g. anaemia, weight gain) or child morbidity and mortality outcomes explored: (1) the impact of multiple micronutrient supplements (MMNS) during pregnancy compared to standard iron-folate supplements;[28] (2) the effectiveness of periconceptual folate supplementation, balanced energy and multiple micronutrient supplementation in reducing stillbirths;[29] (3) the effects of advice regarding energy or protein intake or actual supplementation or restriction;[30] and (4) daily oral iron supplementation.[31]

The review comparing MMNS to standard iron-folate supplements [30] comprised data extracted from 17 studies and showed no significant effect thereof on maternal anaemia in the third trimester (RR 1.03 (0.87‒1.22)) but a significant decrease in SGA babies (RR 0.91 (0.86‒0.96)). No significant overall effect on neonatal mortality was observed (RR 1.05 (0.92‒1.19)), whereas for those born at home, increased mortality risk was observed (RR 1.47 (1.13‒1.92)). However, the latter finding was attributed to the standard of care provided to those who delivered at home, given that this increased effect was not observed when analysing data where ≥ 60% of births occurred in facilities (RR 0.94 (0.81‒1.09)). No change in anaemia outcomes were attributed to initiation of the MMNS during the third trimester as opposed to before pregnancy.

A study reporting on 13 RCTs which explored the effectiveness of periconceptual folate, balanced energy and multiple micronutrient supplementation in reducing stillbirths [31] showed a reduction in neural tube defects (NTDs) associated with periconceptual folate supplementation or food fortification when used as primary prevention (RR 0.38 (0.29‒0.51)). A reduction of the primary incidence of NTDs by 41% was demonstrated for folic acid fortification (RR 0.59 (0.52‒0.68), 11 studies). Folic acid fortification was also associated with a non-significant reduction in stillbirths (RR 0.41 (0.16‒1.07)), whereas balanced protein-energy supplementation during pregnancy reduced stillbirths by 45% (RR 0.55 (0.31‒0.97), three studies), although the supporting level of evidence was assessed as low. MMNS was associated with no reduction (RR 0.98 (0.88‒1.10)) and had no significant effect on perinatal mortality either.

A review comprising a mix of trials (five trials; 1 134 women) reviewed the following forms of supplementation: ‘balanced’ energy/protein supplementation (the protein provided less than 25% of the total energy content); high-protein supplementation (the protein provided 25% of the total energy content); and isocaloric protein supplementation (balanced supplements in which the protein replaced an equal quantity of non-protein energy).[32] Nutritional advice to increase energy and protein intake reduced stillbirths (RR 0.55 (0.31‒0.97)) but had no effect on neonatal deaths (RR 0.62 (0.37‒1.05)). However, nutritional advice reduced preterm births (RR 0.46 (0.21‒0.98)) and SGA (RR 0.68 (0.56‒0.84)). No difference was observed for either energy or protein intakes (no change in weighted mean difference (WMD)) or mean gestational age, or pre-eclampsia, when nutritional advice was the primary intervention. Balanced energy/protein supplementation significantly increased gestational maternal weight gain (WMD 20.74 (1.46‒40.02 g/week)), and a non-significant increase in mean birth weight was observed. It also substantially reduced the risk of SGA birth (RR 0.68 (0.56‒0.84))). High *protein* supplementation was associated with a small but non-significant increase in weekly maternal weight gain, and a small non-significant decrease in mean birth weight. A small, non-significant increase in neonatal death was observed (RR 2.78 (0.75‒10.36)). Isocaloric protein supplementation showed no change in weekly maternal weight gain or mean birth weight, although a small increased risk of SGA from one trial (RR 1.35 (1.12‒1.61)) was reported. Energy/protein restriction in women with overweight or high weight gain showed a significant reduction in maternal weight gain (WMD −254.81 (436.56‒73.06) g/week)) and mean birth weight.

In another SR, the effect of daily oral iron supplementation in pregnant women, either alone or with folate and with other vitamin and minerals, was reviewed.[33] Daily supplementation of folic acid; folic acid plus multiple micronutrients versus multiple micronutrients; folic acid supplementation with a placebo group; and folic acid plus multiple micronutrients versus multiple micronutrients were explored. Other combinations were also explored (Supplemental File: Appendix 1). Supplementation started before pregnancy and was discontinued 12 weeks thereafter. The results showed that iron supplementation decreased the risk of low birth weight (<2500 g) compared to controls (8.4% versus 10.2%, average risk ratio (RR) 0.81; 95% confidence interval (CI) 0.68–0.97, 11 trials, 8480 women), and resulted in a higher mean birth weight for those infants whose mothers received iron during pregnancy (average mean difference (MD) 30.81; 95% CI 5.94–55.68, 14 trials, 9385 women). The risk of maternal anaemia at term decreased by 70% (RR 0.30; 95% CI 0.19–0.46, 14 trials, 2199 women), and iron deficiency at term by 57% (RR 0.43; 95% CI 0.27–0.66, seven trials, 1256 women).

#### Local SA strategies, policies, guidelines and practices

Iron and folate supplementation, multiple micronutrient supplementation [11,13] and healthy eating for optimal weight management during pregnancy and lactation are promoted in PHC and community-level programmes, including in schools [13], as part of basic antenatal care for pregnant women in SA. Evidence-based interventions for antenatal detection of malnutrition are also promoted. Furthermore regulations relating to the fortification of foodstuffs were amended (2005) and guidelines for the fortification of wheat and maize were published. The policies align with the existing evidence base.

#### Detection and treatment of maternal syphilis 28 days before birth

Syphilis detection and treatment evidence is presented in a review that aimed to estimate the effect on syphilis-related stillbirths and neonatal mortality of detection and treatment of active syphilis in pregnancy with at least 2.4 MU benzathine penicillin (or equivalent), compared to those who did not receive this treatment at least 28 days prior to birth. A random-effects MA [32] of eight studies produced an estimated 82% reduction in stillbirths for syphilis RR 0.18 (95% CI 0.10‒0.33). Perinatal mortality (stillbirth and early neonatal mortality) was significantly reduced (RR 0.20 (95% CI 0.13–0.32)), as were preterm births for those who received penicillin treatment (RR 0.36 (95% CI. 0.27–0.47)). The incidence of congenital syphilis in live-born infants was also reduced (RR 0.03 (0.02‒0.07)).

#### Local SA strategies, policies, guidelines and practices

Rapid syphilis screening is to be conducted at the first antenatal care visit, with a second test done between 32 and 34 weeks, should the first test be negative.[11] All women who receive no antenatal care and present for the first time in labour, and those whose syphilis status is not known at the time, should have a rapid syphilis screening test done. Guidance for the diagnosis and syndromic treatment of chancroid is also presented in the guideline, and treatment is with benzathine penicillin 2.4 MU, regardless of titre levels, demonstrating alignment with the evidence base.

#### Use of calcium/antiplatelets to prevent hypertensive disorders

Two broad approaches to prevent pre-eclampsia and its complications were identified: calcium supplementation, and use of antiplatelets.

Calcium supplementation (1 g/day) from at least 34 weeks’ gestation compared to placebo was reviewed for women with low/average or high risk of hypertensive disorders, and those with low or adequate dietary calcium intake.[33] Eleven good-quality studies show that calcium supplementation was associated with reduced hypertension in the aforementioned groups (overall (RR 0.58 (0.43‒0.79)). Pre-eclampsia was reduced in those receiving calcium supplementation (RR 0.35 (0.20‒0.60)), particularly for those at high risk of developing hypertension (RR 0.22 (0.12‒0.42)) and those with low baseline dietary calcium intake (RR 0.29 (0.16‒0.54)). For women at risk of developing hypertension, reduced preterm delivery (RR 0.45 (0.24‒0.83)) and low birth weight (<2500 g) (RR 0.45 (0.22‒0.95)) were observed. Too few events were reported to determine the effect on maternal deaths, and there was too little evidence to assess stillbirth or death before discharge.

The role of calcium supplementation (2 g/day) during pregnancy in reducing hypertension was assessed using 10 RCT studies conducted in developing countries,[34] with a view to assess its effect on maternal and neonatal mortality. All-cause neonatal mortality was reduced (RR 0.70 (0.56‒0.88)). Gestational hypertension (±proteinuria) (RR 0.55 (0.36‒0.85)) and pre-eclampsia (RR 0.41 (0.24‒0.69)) were also reduced, as was the risk of preterm birth (RR 0.88 (0.78‒0.99)). A non-significant reduction was noted for severe eclampsia (RR 0.70 (0.46‒0.69)). Women with higher pre-pregnancy risks also showed reductions in pre-eclampsia (RR 0.18 (0.07‒0.42)). Non-significant reductions in low birth weight and SGA were demonstrated. One study in the review reported that the all-cause maternal mortality was reduced (RR 0.17 (0.03‒0.76)).

Antiplatelet agents (primarily aspirin) for prevention of primary and secondary pre-eclampsia were explored. Fifty-nine randomised trials were included in the review, and pregnant women with normal blood pressure, chronic hypertension, and pregnancy-induced or gestational hypertension were the participants.[35] When comparing antiplatelet agents versus placebo or no treatment for primary prevention of pre-eclampsia and its complications, any reported deaths (stillbirths, neonates, infants) were reduced (RR 0.86 (0.76‒0.98)), more so for those at high risk (RR 0.69 (0.53‒0.90)). Administration was also associated with reductions in women at high risk of pregnancy-induced hypertension (RR 0.54 (0.41‒0.70)) and those at high risk of proteinuric pre-eclampsia (RR 0.75 (0.66‒0.95)). The risk of pre-eclampsia was also reduced (RR 0.83 (0.77‒0.89)). Administration of higher doses (>75 mg/day) was also associated with a lower risk of proteinuric pre-eclampsia (RR 0.64 (0.51‒1–0.80)). Borderline significant reductions were demonstrated for preterm birth and SGA. Antiplatelet agents versus placebo or no treatment for the secondary prevention of pre-eclampsia and its complications in women with gestational hypertension showed no statistical difference for any reported deaths. Proteinuric pre-eclampsia (RR 0.60 (0.45‒0.78)) was reduced. Reductions in preterm birth and SGA (non-significant) were shown. Only one trial showed reduction in very low birth weight (RR 0.24 (0.09‒0.65)).

#### Local SA strategies, policies, guidelines and practices

Alignment with the evidence base is demonstrated in that all women are to receive calcium supplementation throughout their pregnancies.[11] Low-dose aspirin (75 mg) is indicated for those who had a prior pregnancy loss due to severe pre-eclampsia or abruption placenta, and is to be prescribed for pregnant women as from 12 weeks of pregnancy until 34 weeks’ gestation. It is advised that these women be managed at a regional hospital by a specialist or through shared care at a district hospital.[11]

#### Interpersonal psychotherapy to prevent and treat antenatal depression

The prevention and treatment of antenatal depression using psychosocial and psychological interventions in the form of interpersonal psychotherapy in impoverished women (16 sessions of 45 minutes delivered by a trained therapist) compared to usual antepartum care was explored.[36] Most therapists were social workers with a Master’s degree, one had a PhD and another was a medical doctor. All had psychotherapy experience. However, only one trial involving 38 women was retrieved in the review process. Interpersonal psychotherapy, compared to a parenting education programme, was shown to reduce maternal depressive symptomatology immediately post-treatment using a Clinical Global Impression Scale (RR 0.46 (0.26‒0.83) and the Hamilton Rating Scale for depression (non-significant change) (RR 0.82 (0.65‒1.03)). The evidence was therefore considered to be inconclusive to promote interpersonal psychotherapy on a large scale for antenatal depression.

#### Local SA strategies, policies, guidelines and practices

Mention is made of depression in the Guidelines for Maternity Care in SA 2015,[11] but in the context of stopping methyldopa after delivery in women who had gestational hypertension. It was advised that another antihypertensive be used. Depression is also mentioned in the context of HIV treatment regarding the cessation of efavirenz in active psychiatric disease accompanied by psychotic symptoms; the medication is not to be stopped if depression is diagnosed. The guidelines do recommend that HIV-infected women with active psychiatric disease be referred for review by a psychiatrist, and the guideline also recommends that mental health matters be addressed as part of preconception care. The National Mental Health Policy Framework and Strategic Plan 2013‒2020 [14] proposes that maternal mental health treatment programmes be incorporated into routine antenatal and postnatal packages. No further details regarding treatment are provided in either guideline. More should be done to better treat and manage antenatal depression.

#### Prevention of mother-to-child transmission of HIV

A SR [37] was conducted to determine whether, and to what extent, ART regimens that aim to reduce the risk of mother-to-child transmission of HIV decrease the transmission risk and its impact on maternal and infant morbidity and mortality. Pregnant women with HIV infection or infants born to mothers with HIV infection received the intervention. The drug combinations and outcomes and summary measures are presented in Supplemental File: Appendix 2. The results showed that a combination of antiretroviral drugs ‒ namely, triple therapy ‒ are more effective than single regimens for decreasing mother-to-child transmission (MTCT). A combination of antepartum zidovudine, and intrapartum and postpartum zidovudine and lamivudine (3TC) administered to mothers and to infants for seven days postpartum, or a regimen where a single dose of nevirapine was administered to mothers in labour and babies at delivery, is both effective and feasible. In late presentation of an HIV-infected woman, post-exposure prophylaxis of a single dose of nevirapine at delivery and zidovudine to the infant for the first six weeks of life is beneficial in reducing MTCT.

#### Local SA strategies, policies, guidelines and practices

Alignment is demonstrated in that locally, newly diagnosed pregnant women are started on fixed-dose combination (FDC) comprising tenofovir+lamivudine (or emtricitabine)+efavirenz (lifelong) on the same day.[15] If the woman presents during labour and tests HIV-positive, single-dose nevirapine+single-dose truvada+zidovudine should be administered three-hourly in labour. For the infant, if the mother is on lifelong ART for more than four weeks prior to delivery, nevirapine is prescribed at birth and then daily for six weeks. If the mother received no ART before or during delivery and tests positive for HIV more than 72 hours after delivery, or if she is newly diagnosed within 72 hours after delivery, or if the mother started ART less than four weeks prior to delivery, the infant receives nevirapine immediately and then daily for 12 weeks if breastfed.

### Childbirth

#### Antibiotic treatment during with premature rupture of membranes and rupture of membranes

A SR [38] was conducted to evaluate the immediate and long-term effects on maternal infectious morbidity, neonatal morbidity and mortality, and longer-term childhood development, of administering any antibiotic compared to placebos to women with premature rupture of membranes (PROM) before 37 weeks. The results of 22 trials showed that chorioamnionitis (RR 0.66 (0.46‒0.96)), babies born within 48 hours (RR 0.71 (0.58‒0.57)), neonatal infections (RR 0.67 (0.52‒0.85)), and an abnormal cerebral ultrasound scan prior to discharge from hospital (RR 0.81 (0.68‒0.98)) were significantly reduced when antibiotics were administered. Days spent in the neonatal ICU were also decreased when antibiotics were used (mean difference (MD) −5.05 (−9.77 to −0.33). When comparing erythromycin and co-amoxyclav, delivery within 48 hours was less common after co-amoxyclav use (RR 1.14 (1.02‒1.28)). Necrotising enterocolitis was decreased after use of erythromycin (RR 0.46 (0.23‒0.94)) and the risk was increased after use of co-amoxyclav (RR 4.72 (1.57‒14.23).

The second SR [39] comprising 18 RCTs examined the effect on neonatal mortality of administration of antibiotics to women with preterm premature rupture of membranes (pPROM). All cause neonatal mortality or mortality before discharge had a RR of 0.90 (0.72–1.12). The risks of respiratory distress syndrome (RR 0.88 (0.80‒0.97)), intraventricular haemorrhage (RR 0.67 (0.49‒0.92)) and early onset confirmed sepsis (RR 0.61 (0.48‒0.77)) were decreased in all groups. The necrotising enterocolitis risk remained unchanged (RR 0.76 (0.56‒1.05)).

#### Local SA strategies, policies, guidelines and practices

The 2015 SA Maternity Guidelines [11] state that antibiotic treatment is indicated for preterm labour (onset of labour after gestation of ≥24 weeks and before 37 completed weeks of pregnancy), premature rupture of membranes (gestational age ≥34 weeks (or estimated foetal weight ≥2 kg if gestation unknown)) and chorioamnionitis, demonstrating alignment with evidence. The details of the various indications are presented in Appendix 1 in Supplemental File. The guidelines also advise healthcare providers on managing patients who present at primary health care facilities and for referral to the next level of care.

#### Corticosteroid administration at presentation of preterm labour

A review of 10 trials [40] explored administration of different types of corticosteroids (dexamethasone or betamethasone) and various regimens, timing of administration and administration modes thereof for women at risk of preterm labour (PTL) (before 37 weeks) as a result of either spontaneous PTL, preterm pre-labour rupture of membranes, or elective preterm birth. Dexamethasone use was associated with a reduction in the incidence of intraventricular haemorrhage compared to betamethasone (RR 0.44 (0.21‒0.92)). No statistically significant changes were observed for respiratory distress syndrome, bronchopulmonary dysplasia, severe intraventricular haemorrhage, perinatal death, and mean birth weight.

Focusing specifically in LMICs, a review of the effect of antenatal steroid administration to women before anticipated PTL (<36 weeks) compared with treatment (ventilation and/or surfactant) or no placebo, and the effect thereof on neonatal mortality [41] included 44 studies, including 18 RCTs. Neonatal mortality was significantly reduced in those receiving antenatal steroids (RR 0.69 (0.58‒0.81)). A larger effect size for mortality was observed in middle-income countries compared to high-income countries (RR 0.47 (0.35‒0.64)). A 37% reduction in morbidity was observed (RR 0.63 (0.49‒0.81)).

#### Local SA strategies, policies, guidelines and practices

As per the evidence corticosteroid treatment for PTL (age ≥34 weeks (or estimated foetal weight ≥2 kg if gestation unknown)) and PROM (gestational age 24‒33 weeks or estimated foetal weight 600g–1999 g) is presented in the 2015 SA Maternity Guidelines.[11] The details of the various indications are presented in Supplemental File: Appendix 2. Options of betamethasone or dexamethasone are presented.

#### Magnesium sulphate (intravenous/intramuscular) versus phenytoin as part of managing eclampsia

To assess the effects of magnesium sulphate (intravenous (IV)/intramuscular (IM)) use compared to phenytoin on eclampsia (before or after delivery; irrespective of being on an anticonvulsant), an analysis of seven trials involving 972 women was conducted.[42] Magnesium sulphate was associated with a large reduction in the recurrence of seizures compared to phenytoin (RR 0.34 (0.24‒0.49)). A non-significant reduction in maternal mortality was observed for three trials (RR 0.50 (0.24‒1.05)). A single trial showed a reduction in pneumonia risk (RR 0.44 (0.24‒0.79)), ventilation (RR 0.68 (0.50‒0.91), and admission to ICU (RR 0.67 (0.5‒0.89) for magnesium compared to phenytoin. In the neonate, a single trial also demonstrated fewer admissions to a special baby-care unit (RR 0.73 (0.58‒0.91)) or fewer babies’ deaths, or fewer special baby-care unit admissions for more than seven days (RR 0.77 (0.63‒0.95)). No difference in perinatal mortality was observed (RR 0.85 (0.67‒1.09)).

#### Local SA strategies, policies, guidelines and practices

IV magnesium sulphate to prevent convulsions is advised in the Guidelines for Maternity Care in SA 2015.[11] If the waiting time for transfer to the next level of care is anticipated to be longer than 4 hours, IM administration in addition to the IV dose is advised as part of the preventative treatment.

#### Supply of clean birth kits for deliveries

The state of knowledge regarding the effects of the use of birth kits on clean birth practices for newborn and maternal outcomes was explored in a SR where traditional birth attendants (TBA)/skilled birth attendants (SBA), *dayas* (traditional birth attendants in Pakistan), and *dais* (traditional birth attendants in Egypt) were provided with birth kits. The variations thereof are presented in Supplemental File: Appendix 3. One study [43] reported a reduction in perinatal mortality (OR 0.70 (95%CI 0.59‒0.82)), stillbirths (OR 0.69 (95%CI 0.57‒0.83)), and NNMR (OR 0.71 (95%CI 0.62‒0.83).[44] Another study in the SR showed a reduction in NNMR (RR 0.17 (95%CI 0.13‒0.23)),[45] and a reduction in tetanus-related mortality (RR 0.01 (95%CI 0.001‒0.09)). In another study, there was a 21.83% decrease in NNMR in a four-year period.[46] A reduction in sepsis (RR 0.12 (95%CI 0.02‒0.93),[47] and in omphalitis [48–51] were also demonstrated. In terms of maternal outcomes, one study demonstrated a reduction in maternal mortality (OR 0.74 (95%CI 0.45‒1.23)), puerperal sepsis (OR 0.17 (95%CI 0.13‒0.23)), and haemorrhage (OR 0.61 (95%CI 0.47‒0.79). A reduction in puerperal sepsis was demonstrated in two studies (OR 0.11 (95%CI 0.01‒1.06) and OR 0.31 (95%CI 0.18‒0.54)). In all three studies, women who delivered at home reported higher birth-kit use than those who delivered in facilities: home 63%; facility 54%; home 81.8%, facility 67.7%. Where the intervention package included a birth kit, reduced newborn mortality was demonstrated (three studies; one intervention including training and support for TBAs). The intervention group demonstrated increased referral to emergency obstetric care (OR 1.50; 95% CI 1.19‒1.91). Another study showed a relative reduction in maternal mortality (OR 0.74; 95% CI 0.45‒1.23).[52]

#### Local SA strategies, policies, guidelines and practices

Clean and safe deliveries are promoted as part of routine labour care. However, clean birth kits are not widely distributed in communities, as women are encouraged to give birth in facilities staffed with skilled birth attendants.

#### Presence of birth attendance during delivery

Skilled birth attendance (SBA) was reviewed in a study that explored changes in stillbirths and perinatal mortality due to the provision of SBA and the provision of Emergency Obstetric Care (EOC).[53, p. 2] A SBA was defined as:

an accredited health professional – such as a midwife, doctor or nurse – who has been educated and trained to proficiency in the skills needed to manage normal (uncomplicated) pregnancies, childbirth and the immediate postnatal period, and in the identification, management and referral of complications in women and newborns.

Data were retrieved from 21 studies, and two studies demonstrated a significant reduction in stillbirths due to SBA (RR 0.77 (0.69‒0.85)). Overall, perinatal mortality was decreased by 12% (RR 0.88 (0.82‒0.95)), and the provision of basic and comprehensive emergency obstetric care reduced intrapartum stillbirths by between 45% and 74% (IQR: 30–40%).

The effect of traditional birth attendance (TBA) was explored in a SR comprising only four studies.[54] TBA training on TBA and maternal behaviours thought to mediate positive pregnancy outcomes did reduce stillbirths (OR 0.69 (0.57‒0.83)) and neonatal deaths (OR 0.71 (0.61‒0.82)), although the results came from only one study. No changes were observed for maternal mortality and perinatal death. However, obstructed labour was increased (OR 1.26 (1.03‒1.54) in the intervention groups, whereas the frequency of haemorrhage or postpartum haemorrhage and mean volume of blood loss was decreased (OR 0.61 (0.47‒0.79)). In the intervention cluster, the frequency of puerperal sepsis was significantly reduced (OR 0.17 (0.13‒0.23)).

A SR was conducted to explore which cadres of staff, in terms of non-facility births, would have an impact on intrapartum-related deaths.[55] MA was only possible for assessing the effect of community SBA on perinatal mortality and early neonatal mortality (ENM). Training community SBAs reduced perinatal mortality (RR 0.88 (0.83‒0.95)) and ENM (RR 0.87 (0.79‒0.97)). Furthermore, integrated CHW packages focusing on the primary and secondary prevention of these outcomes through CHWs also showed a reduction in perinatal mortality (RR 0.72 (0.62‒0.84)) and ENM (RR 0.64 (0.56‒0.73)).

#### Local SA strategies, policies, guidelines and practices

Skilled midwifery and obstetric services, including skilled attendance at all births and mothers’ waiting units, is encouraged as standard practice in SA. The Maternity Guidelines specifically state that ‘the practice of home births should be discouraged’.[11]

### Neonate

#### Promotion of kangaroo mother care/skin-to-skin contact

The results of a review of 16 studies exploring kangaroo mother care (KMC) to reduce morbidity and mortality in low-birth-weight (<2500 g) infants compared to usual neonatal care before or after initial stabilisation using usual care were presented in a single SR.[52] When assessed at discharge or 40‒41 weeks, KMC was associated with reduced mortality risk (RR 0.60 (0.39‒0.93)), nosocomial infection/sepsis (RR 0.42 (0.24‒0.73)), hypothermia (RR 0.23 (0.10‒0.55)), and reduced length of hospital stay duration by 2.4 days. At most recent follow-up, KMC was associated with reduction in mortality risk (RR 0.68 (0.48‒0.96)) and severe infection/sepsis (RR 0.57 (0.40‒0.80)).

Early skin-to-skin contact (SSC) for healthy newborn infants (i.e. naked baby between breasts of mother) was compared to routine care (swaddled infants, dressed and held in their mothers’ arms, placed in a crib or under radiant warmers).[56] Early SSC was assessed within the first minute after birth (birth SSC), within 30‒40 minutes after birth (very early SSC), and between one and 24 hours after birth (early SSC). Twenty-four RCTs were included, but data from one study demonstrated a statistically non-significant improvement in cardiorespiratory stability in late preterm infants with SCC (mean difference 2.88 (0.53‒5.23). In two trials, a significantly higher blood-glucose level was observed 70‒90 minutes following birth in SSC infants (MD 10.56 (8.40‒12.72)).

#### Local SA strategies, policies, guidelines and practices

KMC/skin-to-skin contact is promoted for sick neonates and neonatal emergencies and for hypothermia in infants presenting with severe acute malnutrition and premature/preterm neonates.[57]

#### Vitamin A supplementation in term neonates within the first 28 days of life

Vitamin A supplementation in term neonates within the first 28 days of life and its impact on mortality in developing countries was explored in a review of seven trials.[58] Term neonates were defined as those born between 37 and 42 weeks’ gestation up to 28 days post-birth. The comparator was placebo or no supplementation. Data from three studies show a significant reduction in infant mortality at six months in those receiving vitamin A supplementation (RR 0.82 (0.68‒0.99)). Data from five studies showed a reduction in infant mortality at six months as well (RR 0.86 (0.77‒0.97)). No significant effect in infant mortality at 12 months of age was shown (RR 1.03 (0.87‒1.23)).

#### Local SA strategies, policies, guidelines and practices

Vitamin A supplementation for term neonates is only indicated for prematurity/preterm, severe under-nutrition, persistent diarrhoea, clinical signs of deficiency, or measles. Prophylaxis starts at 6–11 months and is continued every six months until 5 years of age. Vitamin A supplementation is therefore not routinely given to neonates within the first 28 days of life in SA [59]. This follows the World Health Organization recommendation that the evidence base is as yet insufficient to promote this practice [60]. Food fortification of maize and wheat, however, is widely promoted in SA and is regulated as part of health legislation in the country.

### Postnatal

#### Antidepressants to prevent postnatal depression

To explore the effectiveness of various antidepressant drugs with (1) standard clinical care, or (2) any other form of intervention (e.g. hormonal, psychological or social support), as well as the potential adverse effects on the foetus/infant in non-depressed women, or those who had given birth within the previous six weeks,[61] two trials consisting of 73 participants (women with previous postpartum depression) were reviewed. No benefit of nortryptiline initiated within 24 hours of delivery (*n *= 26) was demonstrated over a placebo for preventing a recurrence of major postpartum depression, or in time to recurrence (exact log-rank<0.001, exact *p* = 0.83). Sertraline initiated within 24 hours after delivery was associated with prevention of a recurrence, although the numbers of patients in the latter trial were recruited from a psychiatric outpatient setting and may have been more motivated to accept treatment.

#### Local SA strategies, policies, guidelines and practices

Incorporation of this evidence is the same as that documented earlier in this paper in relation to antenatal depression prevention and treatment.

#### Antiretrovirals to treat HIV/AIDS in children <3 years

A SR reviewing ART in HIV-infected children under 3 years of age ‒ the best time to initiate ART in this age group and the best line of treatment [62] ‒ comprised eight studies addressing treatment initiation (two studies), what to initiate (two studies), substitution of lopanavir/ritonavir (LPV/r) with nevirapine (NVP) (one study), whether to adopt an induction-maintenance ART approach (one study), and whether to interrupt treatment (one study). The details of the various treatment regimens are presented in Supplemental File: Appendix 2. Treatment initiation in asymptomatic infants with good immunological status was associated with a significant reduction in mortality or disease progression (HR 0.25 (0.12‒0.51)) in one trial. Evidence also showed that a LPV/r-based first-line regimen is more efficacious than one based on NVP, irrespective of the use of PMTCT (hazard for treatment failure at 24 weeks: HR 1.79 (1.33‒2.41)), for virological failure at 24 weeks (HR 1.84 (1.29‒2.63)). The review also proposed that although a four-drug induction-maintenance approach for 36 weeks followed by three-drug ART compared to standard three-drug ART was associated with short-term virological (OR 1.99 (1.09‒3.62) and immunological (CD4 count mean difference 1.70 (0.61‒2.79) benefits during the induction phase, this was not recommended as a routine treatment strategy, as long-term benefits were not observed.

#### Local SA strategies, policies, guidelines and practices

According the SA national guidelines (2015),[63] children <3 years or older children weighing <10 kg should be given (Abacavir) + 3TC (lamivudine) + LPV/r (lopinavir/ritonavir). Doses are based on the child’s weight with adjustments as the child grows. Furthermore, the guidelines provide that children aged 5 years or younger should receive ARTs as soon as possible regardless of clinical staging. Immediate initiation of infant ART should be done with first positive HIV PCR, whilst waiting for confirmatory test results. Children on ART should initially be seen monthly for regular follow-ups and monitoring. Once they stabilise, they need to be followed up for three to six monthly. This is in line with the evidence base presented above.

### Vitamin A supplementation in children aged 6 months to 5 years

To evaluate the effect of vitamin A supplementation (VAS) for preventing morbidity and mortality in children, a SR was conducted comprising 43 RCTs involving children living in the community and aged 6 months to 5 years [64]. Synthetic vitamin A was given orally compared to either placebo or treatment-as-usual for the control groups. Various doses and frequencies were also examined. Meta-analyses for 17 trials reveal a 24% reduction in the risk of all-cause mortality for vitamin A compared with controls (RR 0.76 (0.69‒0.83)). Vitamin A supplementation was associated with a reduced incidence of diarrhoea (RR 0.85 (0.82‒0.87)) and risk of diarrhoea mortality (RR 0.72 (0.57‒0.91)). This risk reduction was also observed for measles morbidity (RR 0.50 (0.37‒0.67)). No significant increase in cause-specific measles mortality, respiratory disease or meningitis was observed, nor was a significant effect on respiratory disease incidence or episodes of hospitalisations due to diarrhoea or pneumonia observed.

### Local SA strategies, policies, guidelines and practices

The guidelines for vitamin A supplementation are as described in the previous section on this indicator, with supplementation of children being considered standard practice. Administration of vitamin A now forms part of the standard care package provided by community health workers.[65]

### Across the first 1000 days

#### Community-level service delivery interventions

The effects of lay health worker (LHW) interventions in primary and community health care on maternal and child health and the management of infectious diseases was explored by reviewing 55 studies and 3568 LHWs (paid or voluntary) which included community health workers, village health workers, birth attendants, peer counsellors, nutrition workers, and home visitors.[66] Compared to usual care, LHW interventions showed a non-significant reduction in mortality for children <5 years old (RR 0.75 (0.55‒1.03)) and neonates (RR 0.76 (0.57‒1.02)); however, the quality of the studies was low. Child morbidity (fever, acute respiratory infection) also demonstrated a non-significant reduction (RR 0.86 (0.75‒0.99)). The study further explored the effect of LHW interventions for promoting breastfeeding compared to usual care and showed a small but significant increase in breastfeeding (RR 1.24 (1.14‒1.61)) and ongoing breastfeeding (any form) at 12 months post-partum (RR 1.24 (1.10‒1.39)).

An SR was conducted of five studies examining the effectiveness of community-based intervention packages in reducing maternal and neonatal morbidity and mortality and improving neonatal outcomes.[67] The intervention included additional MNCHW training (over and above usual training) of outreach workers in the community in the form of lectures, and supervised hands-on training in a healthcare facility and/or within the community. In terms of maternal mortality, community-based packages using outreach workers were not shown to reduce maternal mortality (RR 0.70 (0.59‒1.02)). More specifically, building support groups as part of the aforementioned package, or mobilised community and home visits during antenatal and postnatal periods, were not associated with any change. Training of traditional birth attendants (TBAs) who made home visits during the antenatal period and delivery were shown to reduce maternal mortality (RR 00.70 (0.51‒0.96)). For neonatal mortality, community-based packages using the outreach workers were shown to reduce neonatal mortality (RR 0.76 (0.68‒0.85)). Moreover, in terms of reducing neonatal mortality, the following were shown to significantly reduce neonatal deaths: (1) building support through home visitations and community mobilisations (RR 0.79 (0.68‒0.92)); (2) community mobilisation and home-based neonatal treatment (RR 0.66 (0.47‒0.93)); and (3) home-based care and sepsis management as part of the package (RR 0.43 (0.27‒0.69; one study). A combination of trained TBAs and home visits showed no effect (RR 0.79 (0.63‒1.01)). Early neonatal death was also decreased through community-based interventions (RR 0.79 (0.64‒0.86)), with (1) community mobilisation and antenatal and postnatal home visits (RR 0.81 (0.69‒0.94)), (2) community support groups (RR 0.76 (0.58‒0.98)), and (3) home-based care (RR 0.45 (0.28‒0.82)) also being effective. No significant change was shown when trained TBAs made antenatal and intrapartum visits. Finally, late neonatal mortality was decreased as well (RR 0.72 (0.60‒-0.83)).

A SR of the effect of community-level interventions on reducing maternal mortality reviewed outcomes in maternity populations or women of childbearing age (15–49 years) in the community (home, school, local clinic; delivered by person within the community; primary care setting).[68] Community-level interventions were compared to usual care, hospital care or community interventions. Thirteen studies (a mix of RCTs and observational studies) were reviewed, but only two trials reported on the effect of perinatal practices on maternal mortality and showed a reduction thereof (RR 0.68 (0.39‒0.98)). No difference was reported for ‘minimal goal-orientated’ versus ‘usual’ antenatal care.

#### Local SA strategies, policies, guidelines and practices

In SA, as part of the PHC Re-engineering Strategy,[17] ward-based outreach teams comprising CHWs led by a nurse are expected to conduct community assessments and mobilise the community; identify and manage minor health problems; screen; promote healthy lifestyles; support mothers and children; and conduct household visits.[12] Information regarding the teams’ training is shown in Appendix 4 in Supplemental File. Four antenatal visits starting at 14 weeks are to be scheduled in the woman’s home, during which pregnancy care, PMTCT promotion, encouragement and follow-up on antenatal care attendance, identification of the risks and danger signs of pregnancy (including birth preparedness and planning) are promoted. Checklists and structured referral forms are used by the teams. Four postnatal visits starting 24 hours after returning home up until 14 days thereafter focus on the provision of essential postnatal care for the mother and related complications, essential care for the neonate, and PMTCT oversight. Through the Integrated Management of Childhood Illness (IMCI) programme, growth and nutrition monitoring are promoted. Vitamin A supplementation is done by CHWs as well.

#### Handwashing with soap or improved water quality to prevent diarrhoea

Two SRs exploring interventions aimed a diarrhoea prevention were identified. The first looked at the effect of handwashing with soap on the risk of diarrhoea in the community.[69] Seventeen studies comprising a mix of intervention and observational designs were selected, with most being of poor quality. Interventions to promote handwashing with soap resulted in a 47% decrease in the risk of diarrhoeal disease (24‒63%), with a 48% reduction in severe enteric infections (35‒66%), and 59% reduction in shigellosis (38‒73%).

In a SR to assess the effectiveness of interventions aiming to improve drinking water quality for diarrhoeal prevention, 33 studies exploring 42 comparisons were retrieved.[70] A wide variety of study designs and interventions was identified, ranging from improvement of quality at the water source to the level of the household. Environmental interventions often accompanied water quality interventions. Diarrhoeal prevention for all ages, including children younger than 5 years, were presented. Source-based interventions were associated with a non-significant reduction in under-5 diarrhoea rates (three trials) (RR 0.93 (0.82‒1.05)). Household interventions significantly reduced under-5 diarrhoea (RR 0.70 (0.54‒0.89)). At the household level, compliance and water quality interventions in the absence of improved water supplies and sanitation were effective.

#### Local SA strategies, policies, guidelines and practices

The South African health minister, in partnership with the Consumer Goods Council of South Africa (CGCSA), launched the Public Hand Hygiene Campaign in November 2014 to raise awareness on the importance of hand-washing in the prevention of infectious diseases. This campaign sought to educate members of the public, corporate and government institutions on hand hygiene as an important measure to prevent diseases and a practical infection control mechanism. The health ministry also initiated the Global Handwashing Day, which is an annual global advocacy day dedicated to increasing awareness and understanding about the importance of handwashing with soap as an easy, effective, and affordable way to prevent diseases and save lives. It was founded by the Global Public-Private Partnership for Handwashing, and is an opportunity to design, test, and replicate creative ways to encourage people to wash their hands with soap at critical times. Provincial departments of health, e.g. the Western Cape government, have started their own Handwashing Day initiatives to promote handwashing and prevent diarrhoea.

## Discussion

This review of SRs and MAs, based on JBI’s umbrella review methodology, aimed to identify a list of interventions implemented and measured in the first 1000 days of life; which have a documented high-quality evidence base; and for which the effects of these interventions on mortality and morbidity outcomes have been measured. Secondly, we assessed the degree to which these interventions are incorporated into written SA policies, strategic documents and national guidelines (i.e. the *what* and the *how*). Nineteen broad categories of interventions, presented in 32 SRs matching the inclusion and quality criteria, were identified. Many interventions presented in this review targeted the mother during the antenatal and childbirth stages, and were primarily pharmacological (preventive and curative) in nature.

### Translation of evidence into written strategic, policy and guideline documents

The review of existing official high-level strategies, policies and guidelines provided evidence of SA’s commitment to ensuring that MNC practices are aligned with policy (e.g. promotion of at least eight ANC visits, supplementation, pharmacological treatments in the perinatal period, promotion of KMC, PMTCT and ART, and skilled birth attendants). However, opportunities for strengthening the *what* and *how* do exist. More effort can be made to explicitly advocate for optimal birth spacing time periods (between two and five years) and this should be incorporated into all documents. Four antenatal visits, which was the standard in SA, is associated with increased mortality in this review and was recently revised to eight but this change should be widely promoted and implemented. In 2016, the WHO released *Improving Quality of Antenatal Care: New Guidelines* which proposes a new ANC model that raises the recommended number of ANC visits from four to eight, thereby increasing the number of opportunities providers have to detect and address preventable complications related to pregnancy and childbirth.[71]

Depression is an area requiring attention as the link between perinatal depression and poor outcomes is known.[72] The prevalence of depression in women in the SA population was estimated as 66.1% (lifetime; any age) in 2009,[73] and observational studies focusing on ante- or postnatal depression/depressed mood estimate the prevalence to be 22%,[74] 39%,[75] and 48.7% in HIV-infected women. The paucity of specifics regarding how to diagnose and treat depression at the ante- and postnatal stages is evident when reviewing the written documents. This could be due to (1) the weak evidence base (only three SRs applicable to the local setting were retrieved; small study samples); (2) the resource-intense and specialised nature of the tested interventions; and (3) limited focus on the link between depression and maternal and infant mortality and morbidity outcomes. Despite local written commitment to address mental health in pregnancy, gaps should urgently be addressed in terms of strengthening the local evidence base and planning for enhanced service delivery and approaches to support affected women. In July 2013, SA adopted the Mental Health Policy Framework (MHPF) and the Strategic Plan 2013‒2020.[14] Although the MHPF refers to the importance of integrating mental health into primary care platforms ‒ in particular maternal and reproductive health services ‒ these adaptations must still be made. Given the high prevalence of perinatal depression documented in SA studies using diagnostic tools,[76–78] it is not clear which staff cadres would be equipped, whether in terms of training or time available, to conduct screening or psychosocial interventions.

Finally, despite the evidence base for vitamin A supplementation for neonates, SA has not implemented this recommendation as the evidence base is not considered conclusive, in line with WHO recommendations. This is an example of an intervention with an underlying quality evidence base, which due to high-level international consensus was not added as an intervention locally.

### Translation of written evidence into practice

Given the close alignment between the evidence and official high-level guidance documents, the expectation is that MNC health outcomes would be optimal. Closer inspection of routine or survey data (where publicly available) on health outcomes, coverage and uptake suggests that there is a gap between policy, implementation and practice. The couple-year protection rate (measuring the proportion of women aged 15‒49 years who are protected against pregnancies using modern contraceptive methods including sterilisation) was estimated to be 48.2% in 2015/16.[79] In 2014/15, only 53.8% of eligible women presented for a first ANC visit before 20 weeks gestation;[80] and about 84.0% of pregnant women had at least one (booked) antenatal visit (2015/16).[81] In 2012, the prevalence of anaemia in females of reproductive age was 23.1%, and iron deficiency levels were 9.7%, despite widespread supplementation promotion and food fortification.[82] In terms of malnutrition, 26.5% of children aged 1–3 years were stunted,[83] the prevalence of vitamin A deficiency in children under 5 years was 43.6% (2012),[83] and food security was shown to be compromised in rural and urban informal areas.[83]

SA is continuing to strengthen prenatal and other care for women in labour (e.g. through the implementation of District Clinical Specialist Teams, roll-out of Essential Steps in the Management of Obstetric Emergencies training, and promotion of births in health facilities) but more must be done. Death data list hypertensive disorders, pregnancy-related sepsis and stillbirths as the primary causes of death other than HIV. Promotion of facility births and skilled birth attendants requires ongoing strengthening: 83.5% of deliveries take place in facilities as a percentage of expected births in the population. Factors associated with deaths include delays in seeking medical help.[84] Diarrhoeal death rates have rapidly declined nationally but remain high in certain rural areas.[85]

Additional positive examples exist. SA leads the way in that MTCT rates have been drastically reduced to 1.1%, and syphilis prevalence among pregnant women was 1.6% in 2011.[86] In September 2016, SA implemented the ‘universal test-and-treat’ initiative for all eligible persons.[87] This will hopefully increase the current treatment initiation rates for pregnant women and children. More must be done to decrease HIV infections among women of reproductive age and teenagers.[88] Finally, community health worker programmes have been implemented as part of a national strategy.

## Strengths and limitations

In this review we focused specifically on PHC settings in LMICs, using relatively stringent quality assessment criteria in line with evidence based thinking practices. Furthermore, given that only SRs/MAs were included and that morbidity and mortality outcomes, and not coverage or uptake, were explored, a number of interventions demonstrated to be high priority in terms of lives saved were not included in our list. The purpose of this review was not to identify the latter but instead to identify interventions with an existing high-quality evidence base. For example, immunisation is a critical intervention to save the lives of neonates, infants and children; the search found four studies on immunisation [83,85,89,90] that were excluded based on the quality assessment score. Similarly, articles found on other important interventions, e.g. antibiotics for infection,[91] oral rehydration solution (ORS),[92,93] and zinc for diarrhoea,[94] were excluded in the quality appraisal or ranking of evidence. Some articles listed interventions implemented outside of the 1000 day window, and in outcomes were not measured in children under the age of 2 years.

## Conclusion

South Africa has done well to ensure that MNC interventions with a high-quality evidence base are incorporated into local strategies, policies and guidelines. However, for certain interventions the specificities of the evidence can be better incorporated to ensure alignment of policy with evidence. This paper suggests that ongoing review of new evidence is important for identification of evidence gaps. Further, certain interventions such as the identification and treatment of perinatal depression may not significantly impact on mortality, but in a country like SA where its contribution to the burden of disease is steadily increasing, more can be done to better identify and manage it. What we have also demonstrated is that not all high impact interventions, known to significantly reduce mortality and morbidity in the first 1000 days – when applied during that period – have a high quality evidence base, particularly when using mortality and morbidity as outcomes and when applying stringent quality assessment criteria. Regardless, SRs, MAs and methods to synthesise these are useful for regularly summarising the evidence base underpinning health interventions and is a useful tool to inform policymaking and identify research gaps.

Finally, the application of the umbrella review and PRISMA methodologies have located those interventions with a strong evidence base. Excluded from the review were those presented in papers that did not meet high-quality criteria, or those not presented in SRs or MAs. Furthermore, only morbidity and mortality were covered. This has captured some interventions that have some demonstrated effect on morbidity but possibly not mortality (e.g. depression), but has excluded others where the immediate measured outcomes were coverage or uptake (e.g. aspects of breastfeeding). When reviewing the evidence, the methodologies used and outcomes measured undoubtedly influence which interventions are identified and this should be considered during policy making processes.

## Supplementary Material

Supplementary File

## Paper context

South Africa has not met maternal, neonatal and child targets despite significant high-level commitments. A review of systematic reviews and meta-analyses was conducted to summarise evidence and determine the specificities of maternal, neonatal and child interventions that target morbidity and mortality. Local official strategies, policies and guidelines were reviewed to determine how well these interventions are incorporated locally. Better attention to perinatal depression detection and management, and community-level programmes is required; more action is required to shift knowledge into practice on the ground.
