# Supplementary material for: ‘First 1000 days’ health interventions in low- and middle-income countries: alignment of South African policies with high-quality evidence
Source: Glob Health Action. 2017 Jul 18;10(1):1340396. doi: 10.1080/16549716.2017.1340396 (PMC5533118; doi:10.1080/16549716.2017.1340396)
Supplement: Supplementary File [file zgha_a_1340396_sm7611.docx]

**Supplementary file**

File name: Supplementary file

File format: Microsoft Word

Description of data: Data tables labeled three to eleven that are referred to in the text

**Appendix 1** – Table of interventions mapped to the SA policy identified

**Appendix 2** – Table showing data summary

**Appendix 3 –** Table with a detailed summary of the interventions identified

**Appendix 4 -** Table of study characteristics

**Appendix 5 -** Table summary of included studies

**Appendix 6 -** Table showing Quality appraisal of studies using AMSTAR tool and PRISMA questions

**Appendix 7 -** Table showing Ranking of studies with Modified Bhutta et al criteria

**Appendix 8 –** Table showing the different stages in study selection

**Appendix 9 -** Table of excluded studies

**Search String**

**Appendix 1** – Table of interventions mapped to the SA policy identified

| **Category of first 1000 days interventions** | **Interventions** | **What is the intervention according to the evidence base?** | **Who does the intervention ultimately target according to the evidence base?** | **How is the intervention to primarily be delivered according to the evidence base?** | **Type** | **Morbidity (effect)** | **Mortality (effect)** | **Promotion of intervention/practice in South African policy, strategic documents and guidelines** | **Local guidelines** |
| --- | --- | --- | --- | --- | --- | --- | --- | --- | --- |
| **Pre-pregnancy** | **Promotion of birth spacing of between ≥18 months and <60 month** | Birth spacing: interval from one birth and the next consecutive birth; interval between delivery and a following conception (Conde-Agudelo, A, 2006)27 | Women of childbearing age who have recently given birth | Promotion of use and provision of family planning by health care providers | Non-pharmacological | Preterm births (Protective) | Fetal (Insufficient evidence) | Use of contraception is promoted in the following: | Birth spacing is reported as referred to in the Foreward and is promoted throughout the policy document. An exact period is not mentioned.  The following methods are promoted in the guideline: hormonal; intrauterine; emergency; barrier; voluntary sterilisation; fertility-based awareness methods (previously known as natural family planning); lactational amenorrhoea; abstinence and traditional (herbal mixtures, intercrural sex, breastfeeding, abstinence after childbirth). The policy also aims to promote the use of long-acting hormonal contraceptive sub-dermal implant providing contraceptive coverage for a period of 3 years. |
|  |  |  |  |  |  |  |  |  |  |
|  |  |  |  |  |  | LBW (Protective) | Neonatal (Insufficient evidence) | National Department of Health (NDoH). Overview and summary of the National Contraception and Fertility Planning Policy and Service Delivery Guidelines and National Contraception Clinical Guidelines. 2012. |  |
|  |  |  |  |  |  |  |  |  |  |
|  |  | Comparator: various time intervals (< 6 months; 6-11 months; 12-17 months; 18-23; 24-59 months; ≤60 months) |  |  |  | SGA (Protective) |  | Strategic Plan for Maternal, Newborn, Child and Women’s Health (MNCWH) and Nutrition in South Africa 2012 - 2016 |  |
|  |  |  |  |  |  |  |  |  |  |
|  |  |  |  |  |  |  |  |  |  |
| **Antenatal** | **Promotion of a minimum of ≥4 antenatal care visits as part of the antenatal care** **programme** | Promotion of women receiving a minimum of 4 antenatal care visits (goal-orientated in LMIC) during each pregnancy, with a consideration of increasing the frequency in settings where perinatal mortality is high, the standard of care is poor and are considered to have high risk pregnancies. (Dowswell, T, 2010)28 | Pregnant women considered to be low-risk | In health facilities by health care providers | Non-pharmacological | Pre-eclampsia (No significant difference) | Peri-natal mortality (Not Protective) | South African National Department of Health. Guidelines For Maternity Care In South Africa. Pretoria: 2015 | The schedule of (basic) antenatal care visits are as follows for women without risk factors: - Early booking visit (preferably <12 weeks), then at 20, 26-28, 32-34, and 38 weeks, and 41 weeks if still pregnant by then  - Women with risk factors, whose return visits schedules will depend on their specific problems. |
|  |  |  |  |  |  |  |  |  |  |
|  |  |  |  |  |  | Preterm births (No significant difference) |  |  |  |
|  |  | Comparator: Usual care. In LMIC – to between 4-6 visits; HIC – from 13-14 visits to between 6-9 visits) |  |  |  | Vaginal bleeding(No significant difference) |  |  |  |
|  |  |  |  |  |  |  |  |  |  |
|  |  |  |  |  |  | C-Section(No significant difference) |  |  |  |
|  |  |  |  |  |  | Induction of labour |  |  |  |
|  |  |  |  |  |  | (No significant difference) |  |  |  |
|  |  |  |  |  |  | LBW(No significant difference) |  |  |  |
|  |  |  |  |  |  | Admission to neonatal intensive care units (Protective for LIC) |  |  |  |
|  | **Multiple micronutrient and balanced protein energy supplementation** | Twelve studies used multiple micronutrient supplement formula called UNIMMAP which consisted of 30 mg iron, 400 μg folic acid, 15 mg zinc 2 mg copper, 65 μg selenium, 800μg RE vitamin A, 1.4 mg vitamin B1, 1.4 mg vitamin B2, 18 mg niacin, 1.9mg vitamin B6, 2.6 μg vitamin B12, 70 mg vitamin C, 5μg vitamin D, 10 mg vitamin E and 150 μg iodine. The minimum number of micronutrients in the studies was nine. The UNIMAPP formulation containing 30 mg iron and 0.4 mg folic acid was provided in nine trials. The intervention group in the one study received multiple micronutrient tablets containing 0.8 mg of folic acid along with separate iron and folic acid supplementation. Another study showed the multi-micronutrient tablet contained 10 mg of ferrous fumarate and 0.15 mg of folic acid along with supplemental iron-folate. A third study included 60mg iron and 0.4 mg folic acid. Fourth study provided 62.4 mg iron and 0.215 mg folic acid in the multi-micronutrient formulation. (Haider, B, 2011)36 | Pregnant women (number of total participants not mentioned) | (Not clearly specified but assume Healthcare providers deliver the intervention.) | Pharmacological | Maternal anaemia (No change) | Neonatal mortality (No effect) | Roadmap for nutrition in South Africa (2013-2017); Foodstuffs Cosmetics And Disinfectants Act, 1972 (Act No. 54 Of 1972) Regulations Relating To The Fortification Of Certain Foodstuffs: Amendment” (2005) | Ferrous sulphate tablets 200 mg daily, to prevent anaemia. Folic acid tablets 5 mg daily. Multiple micronutrient supplements and targeted supplementary feeding to undernourished individuals (HIV, TB) - Community nutrition programmes, clinic, PHC and hospitals. Healthy eating for optimal weight management during pregnancy and lactation. Implementation of evidence based interventions for detection of malnutrition during pregnancy i.e. MUAC measurement antenatally, including pregnant women and families of children under 24 months; populations with high % of Children 6-23 months with Weight-for-Age (W/A) <2 z-scores.  Foodstuffs Cosmetics And Disinfectants Act, 1972 (Act No. 54 Of 1972) Regulations Relating To The Fortification Of Certain Foodstuffs: Amendment - fortification of maize and wheat. Bbalanced protein energy is not routinely provided. |
|  |  | Comparator: Usual care which is only Iron folate supplementation |  |  |  | SGA (Protective) | Neonatal mortality if born at home (Not Protective) | Refer above | Refer above |
|  |  |  |  |  |  |  |  |  |  |
|  |  |  |  |  |  |  |  |  |  |
|  | **Folic acid, protein energy and multiple micronutrient supplementation** | Provision of folic acid supplementation, folate food fortification, MMN or balanced energy supplementation. (Imdad, A, 2011)31 | Pregnant women | In facilities by health care providers | Pharmacological |  |  | Refer above | Refer above |
|  |  |  |  |  | Peri-conceptual folic acid supplementation | Neural tube defects (Protective) | Stillbirths (Not significant) |  |  |
|  |  | Comparator: usual care with folic acid alone |  |  |  |  |  |  |  |
|  |  |  |  |  | Folic acid fortification | Neural tube defects (Protective) |  |  |  |
|  |  |  |  |  |  |  |  |  |  |
|  |  |  |  |  | Balanced energy supplementation; |  | Stillbirths (Not significant) |  |  |
|  |  |  |  |  |  |  |  |  |  |
|  |  |  |  |  | MMNS |  | Stillbirths (Not significant) |  |  |
|  |  |  |  |  |  |  |  |  |  |
|  |  |  |  |  |  |  |  |  |  |
|  |  |  |  |  |  |  |  |  |  |
|  |  |  |  |  |  |  | Stillbirths (Not significant) |  |  |
|  |  |  |  |  |  |  | Perinatal mortality (Not significant) |  |  |
|  |  |  |  |  |  |  |  |  |  |
|  | **Supplementation containing balanced/protein energy supplementation during pregnancy** | (1)      Nutritional advice to increase energy and protein intake | Pregnant women who were told what amounts of protein and other food groups to eat, they were given a milk drink with the supplement in it, chocolate coloured liquid supplements, biscuits etc. | In facilities by health professionals | Pharmacological | Stillbirths (Protective) |  | Refer above | Refer above |
|  |  |  |  |  |  |  |  |  |  |
|  |  |  |  |  |  | Preterm birth (Protective) |  |  |  |
|  |  | (2)      Balanced energy/protein supplementation (provides <25% total energy) |  |  |  | SGA (Protective) |  |  |  |
|  |  |  |  |  |  |  |  |  |  |
|  |  |  |  |  |  | Maternal weight gain (Protective) |  |  |  |
|  |  |  |  |  |  | Birth weight (Not significant) |  |  |  |
|  |  | (3)      High protein supplements (provides 25% total energy) |  |  |  |  |  |  |  |
|  |  |  |  |  |  |  |  |  |  |
|  |  |  |  |  |  | Maternal weight gain (Not significant) |  |  |  |
|  |  |  |  |  |  |  |  |  |  |
|  |  | (4)      Isocaloric protein supplements (protein replaced equal quantity of non-protein energy) |  |  |  | Birth weight (Adverse) |  |  |  |
|  |  |  |  |  |  |  |  |  |  |
|  |  |  |  |  |  |  | Neonatal death (Adverse) |  |  |
|  |  | (5) Energy/protein restriction in women with overweight or high weight gain (Kramer, M, 2003)32 |  |  |  | Maternal weight gain (No effect) |  |  |  |
|  |  |  |  |  |  |  |  |  |  |
|  |  |  |  |  |  | Birth weight (No effect) |  |  |  |
|  |  | Comparator: usual care |  |  |  | SGA (Adverse) |  |  |  |
|  |  |  |  |  |  |  |  |  |  |
|  |  |  |  |  |  |  |  |  |  |
|  |  |  |  |  |  | Maternal weight gain (Protective) |  |  |  |
|  | **Daily oral iron supplementation** | Any supplements containing iron and folic acid versus same supplements without iron nor folic acid (no iron nor folic acid or placebo) (Peña-Rosas, J,2012)38 | Pregnant women of any gestational age and parity | Physicians or other healthcare professionals including midwives, dieticians or social workers | Pharmacological |  |  | Refer above | Ferrous sulphate tablets 200 mg daily (contains 60mg of iron) and Folic acid tablets 5 mg daily |
|  |  | Comparator: Usual care |  |  |  |  |  |  |  |
|  |  |  |  |  | Any supplements containing iron versus same supplements without iron or no treatment/placebo (no iron or placebo) |  |  |  | Severe anaemia (haemoglobin <8g/dl, symptoms or pallor): |
|  |  |  |  |  |  |  |  |  |  |
|  |  |  |  |  |  | LBW (Protective) | Neonatal death (Not significant) |  | Pregnant women should be started on iron (ferrous sulphate 200mg (1 tablet) three times daily) and folic acid (5mg (1 tablet) daily) supplementation immediately. With Vitamin C 500mg. |
|  |  |  |  |  |  |  |  |  |  |
|  |  |  |  |  |  | Birth weight (Protective) | Maternal Mortality (Not significance) |  |  |
|  |  |  |  |  |  | Premature birth (less than 37 weeks gestation) (Not significant) |  |  | Mild anaemia (haemoglobin >8g/dl but <10g/dl): |
|  |  |  |  |  |  |  |  |  |  |
|  |  |  |  |  |  | Congenital anomalies (Not significant) |  |  | If the woman is less than 34 weeks pregnant, she should be seen again in 4 weeks and the haemoglobin checked again. If it is still low she should be referred to hospital. If she is 34 weeks pregnant or more she needs to be referred to hospital for follow-up and delivery. |
|  |  |  |  |  |  | Maternal anaemia (Protective) |  |  |  |
|  |  |  |  |  |  | Maternal iron deficiency at term (Protective) |  |  |  |
|  |  |  |  |  |  | Severe anaemia (Protective) |  |  |  |
|  |  |  |  |  |  | Infection during pregnancy, placental malaria and parasitaemia (Not significant) |  |  |  |
|  |  |  |  |  |  | Very premature birth (Protective) |  |  |  |
|  |  |  |  |  |  | Infant ferritin (Protective) |  |  |  |
|  |  |  |  |  |  | VLBW, SGA, low apgar score, mean infant Hb levels at 3 and 6 months, admission to special care, head circumference at birth, stunting at long term follow-up (All Not significant) |  |  |  |
|  |  |  |  |  |  | Maternal Hb concentration (Protective) |  |  |  |
|  |  |  |  |  |  | Severe anaemia (Not significant) |  |  |  |
|  |  |  |  |  |  | Severe anaemia at postpartum (Protective) |  |  |  |
|  |  |  |  |  |  | Transfusion provided (Protective) |  |  |  |
|  |  |  |  |  |  | Puerperal infection (Protective) |  |  |  |
|  |  |  |  |  |  |  |  |  |  |
|  |  |  |  |  |  | Ante- or postpartum haemorrhage, individual side effects, placental abruption, preterm rupture of the membranes, |  |  |  |
|  |  |  |  |  |  | pre-eclampsia and moderate anaemia in the postpartum period (All Not significant) |  |  |  |
|  |  |  |  |  |  |  |  |  |  |
|  |  |  |  |  |  |  |  |  |  |
|  |  |  |  |  |  | LBW (Protective) |  |  |  |
|  |  |  |  |  |  | Infant birth weight (Protective) |  |  |  |
|  |  |  |  |  |  | Premature birth (Not significant) |  |  |  |
|  |  |  |  |  |  | Congenital anomalies (Protective) |  |  |  |
|  |  |  |  |  |  | Maternal anaemia (Protective) |  |  |  |
|  |  |  |  |  |  | Maternal iron deficiency (Not significant) |  |  |  |
|  |  |  |  |  |  | Severe anaemia (Not significant) |  |  |  |
|  |  |  |  |  |  | Maternal Hb concentration at term (Protective) |  |  |  |
|  |  |  |  |  |  | Maternal Hb concentration within 6 weeks postpartum (Protective) |  |  |  |
|  |  |  |  |  |  | Maternal severe or moderate anaemia at postpartum (Protective) |  |  |  |
|  |  |  |  |  |  | Severe anaemia (Protective) |  |  |  |
|  |  |  |  |  |  | LBW (Not significant) |  |  |  |
|  |  |  |  |  |  | Maternal anaemia (Protective) |  |  |  |
|  |  |  |  |  |  | Iron deficiency at term (Protective) |  |  |  |
|  |  |  |  |  |  | Maternal severe anaemia at any time (Protective) |  |  |  |
|  |  |  |  |  |  | Infant ferritin concentration at 6 months (Protective) |  |  |  |
|  |  |  |  |  |  | Very premature birth (Protective) |  |  |  |
|  |  |  |  |  |  | Maternal Hb concentration (Protective) |  |  |  |
|  |  |  |  |  |  |  |  |  |  |
|  |  |  |  |  |  | Transfusion provided (Protective) |  |  |  |
|  |  |  |  |  |  |  |  |  |  |
|  |  |  |  |  |  |  |  |  |  |
|  |  |  |  |  | Any supplements containing iron and folic acid | LBW (Not significant) |  |  |  |
|  |  |  |  |  | versus same supplements without iron nor folic acid (no iron nor folic acid or placebo) | Infant birth weight (Protective) |  |  |  |
|  |  |  |  |  |  |  |  |  |  |
|  |  |  |  |  |  | Premature birth (Not significant) |  |  |  |
|  |  |  |  |  |  | Maternal anaemia (Protective) |  |  |  |
|  |  |  |  |  |  | Maternal iron deficiency anaemia (Protective) |  |  |  |
|  |  |  |  |  |  | Maternal hb concentration (Protective) | Neonatal death (Not significant) |  |  |
|  |  |  |  |  |  | LBW (Protective) |  |  |  |
|  |  |  |  |  |  | Infant birth weight (Protective) |  |  |  |
|  |  |  |  |  |  | Premature birth (Not significant) |  |  |  |
|  |  |  |  |  |  | Congenital abnormality (Not significant) |  |  |  |
|  |  |  |  |  |  | Maternal anaemia (Protective) |  |  |  |
|  |  |  |  |  |  | Maternal severe anaemia (Protective) |  |  |  |
|  |  |  |  |  |  | Maternal Hb (Protective) |  |  |  |
|  |  |  |  |  |  |  |  |  |  |
|  |  |  |  |  |  |  |  |  |  |
|  |  |  |  |  |  |  |  |  |  |
|  |  |  |  |  |  | Mean maternal Hb levels at term Protective) |  |  |  |
|  |  |  |  |  |  |  |  |  |  |
|  |  |  |  |  |  |  |  |  |  |
|  |  |  |  |  | Supplementation with iron alone versus no treatment/placebo |  |  |  |  |
|  |  |  |  |  |  |  |  |  |  |
|  |  |  |  |  |  |  |  |  |  |
|  |  |  |  |  |  |  |  |  |  |
|  |  |  |  |  |  |  |  |  |  |
|  |  |  |  |  |  |  |  |  |  |
|  |  |  |  |  |  |  |  |  |  |
|  |  |  |  |  |  |  |  |  |  |
|  |  |  |  |  |  |  |  |  |  |
|  |  |  |  |  |  |  | Neonatal death (Not significant) |  |  |
|  |  |  |  |  |  |  |  |  |  |
|  |  |  |  |  |  |  |  |  |  |
|  |  |  |  |  |  |  |  |  |  |
|  |  |  |  |  |  |  |  |  |  |
|  |  |  |  |  |  |  |  |  |  |
|  |  |  |  |  |  |  |  |  |  |
|  |  |  |  |  |  |  |  |  |  |
|  |  |  |  |  | Supplementation with iron + folic acid versus no |  |  |  |  |
|  |  |  |  |  | Treatment/placebo |  |  |  |  |
|  |  |  |  |  |  |  |  |  |  |
|  |  |  |  |  |  |  |  |  |  |
|  |  |  |  |  |  |  |  |  |  |
|  |  |  |  |  |  |  |  |  |  |
|  |  |  |  |  |  |  |  |  |  |
|  |  |  |  |  |  |  |  |  |  |
|  |  |  |  |  |  |  |  |  |  |
|  |  |  |  |  |  |  |  |  |  |
|  |  |  |  |  | Supplementation with iron + folic acid versus folic acid alone (without iron) supplementation |  |  |  |  |
|  |  |  |  |  |  |  |  |  |  |
|  |  |  |  |  |  |  |  |  |  |
|  |  |  |  |  |  |  |  |  |  |
|  |  |  |  |  |  |  |  |  |  |
|  |  |  |  |  |  |  |  |  |  |
|  |  |  |  |  | Supplementation with iron + other vitamins and minerals supplementation versus same other vitamins and minerals (without iron) supplementation |  |  |  |  |
|  | **Detection and treatment of maternal syphilis >28 days before delivery** | Physical examination, serological testing and treated with at least 2.4 million units of penicillin G (Blencowe, H, 2011)34 | Pregnant women who tested positive for syphilis | Healthcare providers | Pharmacological | Preterm (Protective) | Stillbirths (Protective) | Guidelines for maternity care in South Africa 2015 | Rapid syphilis screening must be done at the first antenatal visit. A second test should be done at between 32 and 34 weeks if the first test if done before 20 weeks and is negative. If “unbooked” or results are not known at labour a rapid syphilis test should be conducted.  All women are to be treated if a positive screening test results obtained, regardless of titre.  Benzathine penicillin, 2.4 million units IM once weekly, for 3 doses. |
|  |  | Comparator: untreated infected women |  |  |  | Congenital syphilis (Protective) | Perinatal mortality (Protective) |  |  |
|  |  |  |  |  |  |  | Neonatal death (Protective) |  |  |
|  |  |  |  |  |  |  |  |  |  |
|  | **Use of calcium supplementation for prevention of hypertensive disorders** | At least 1g/day of calcium (Hofmeyr, G, 2006)35 | Low/moderate or high risk pregnant women 34 weeks and those with low dietary calcium intake | Health care providers | Pharmacological | Hypertension (Protective) |  | Guidelines for maternity care in South Africa 2015 | “All maternity facilities must provide calcium supplementation to all women throughout their antenatal care and ensure the detection, early referral and timely delivery of women with hypertension in pregnancy.”   Calcium tablets 1000 mg daily, to prevent complications of pre-eclampsia (e.g. calcium carbonate (168mg) 2 tablets orally, 3 times daily with food are to be given to all pregnant women. This is best taken 4 hours before or after iron supplements.” |
|  |  |  |  |  |  | Pre-eclampsia (Low + high risk dietary calciaum +high pregnancy risk) (Protective) |  |  |  |
|  |  | Comparator: placebo |  |  |  | Gestational hypertension (Protective) |  |  | " |
|  |  |  |  |  |  | Severe eclampsia (Protective) |  |  |  |
|  |  |  |  |  |  | LBW (Protective) |  |  |  |
|  |  |  |  |  |  | SGA (Protective) |  |  |  |
|  | **Use of calcium supplementation for prevention of hypertensive disorders** | At least 2g/day of calcium (Imdad, A, 2011)36 | Pregnant women in LMIC settings | Health care providers | Pharmacological | Severe morbidities (admission to intensive care unit, eclampsia, severe pre-eclampsia, placental abruption, HELLP syndrome, renal failure, maternal death) (Protective) |  | Refer above | Refer above |
|  |  | Comparator: comparison group either received a placebo or just observed |  |  |  | Eclampsia (Protective) |  |  |  |
|  |  |  |  |  |  |  |  |  |  |
|  |  |  |  |  |  | Severe pre-eclampsia (Protective) |  |  |  |
|  |  |  |  |  |  | Pre-eclampsia (Protective) |  |  |  |
|  |  |  |  |  |  | Gestational hypertension (+-proteinuric) (Protective) |  |  |  |
|  |  |  |  |  |  | Preterm births(Protective) |  |  |  |
|  |  |  |  |  |  | Low birth weight (Protective) |  |  |  |
|  |  |  |  |  |  | SGA (Protective) |  |  |  |
|  |  |  |  |  |  | In women with higher pre-pregnancy risk of developing gestational hypertension (Protective) |  |  |  |
|  |  |  |  |  |  | Women at higher risk of development of hypertension during pregnancy (Protective) |  |  |  |
|  | **Anti-platelet agents for prevention of primary or secondary pre-eclampsia** | Antiplatelet agents (Primarily aspirin) (Duley, L, 2007 )37 | Pregnant women with normal blood pressures, chronic hypertension, pregnancy-induced or gestational hypertension | Health care providers | Pharmacological | Pregnancy induced HPT in high risk women (Protective) | Fetal or neonatal deaths (Protective) | Guidelines for maternity care in South Africa 2015 | “Low dose aspirin (75 mg; or a quarter of a standard tablet) taken daily from the 12th week of pregnancy until 34 weeks gestational age. Normally prescribed for those who have had a previous pregnancy loss due to severe pre-eclampsia or abruptio placenta. These women must be managed at a regional hospital by a specialist or by shared care with a district hospital.” |
|  |  | Comparator: placebo |  |  |  | Proteinuric pre-eclampsia (more so if >75mg/d) (Protective) |  |  |  |
|  |  |  |  |  |  | Preterm birth (Protective) |  |  |  |
|  |  |  |  |  |  | SGA (Protective) |  |  |  |
|  | **Interpersonal psychotherapy to prevent and treat antenatal depression** | 16, 45-minutes sessions of IPT over a 16-week period using cognitive behavioural therapy techniques (Dennis, C, 2007)39 | Impoverished women in HIC settings | Trained therapist | Non-pharmacological | Depression (Protective) |  | The Mental Health Care Act, 17 Of 2002 | Guidelines for maternity care in South Africa: For women who have had gestational hypertension: “Preferably, stop the methyldopa after delivery (as it can exacerbate post-partum depression) and switch to other anti-hypertensive medication if needed.“  HIV treatment in “Women with active psychiatric disease Efavirenz is contraindicated in active psychiatric disease that involves psychotic symptoms: it is not contraindicated in depression. All women with active psychiatric disease need referral, and review by a psychiatrist.”  Mental health issues are to be addressed as part of preconception care  National Mental Health Policy Framework And Strategic Plan 2013-2020: Proposal that “Treatment programmes for maternal mental health as part of the routine antenatal and postnatal care package” |
|  |  |  |  |  |  |  |  | National Mental Health Policy Framework and Strategic Plan (2013-2020) |  |
|  |  | Comparator: parenting education programme that consisted of 16 therapist-led weekly educational sessions for 45 minutes |  |  |  |  |  | National Guidelines for Maternity Care (2015) |  |
|  | **Prevention and management of HIV and prevention of mother to child transmission in pregnancy (PMTCT)** | 1.       Any antiretroviral regimen with the specific aim of decreasing the risk of mother-to-child transmission of HIV infection. | Health facility setting | Clinic staff | Pharmacological |  |  | National Department of Health, National consolidated guidelines for the prevention of mother-to-child transmission of HIV (PMTCT) and the management of HIV in children, adolescents and adults, April 2015. | Fixed dose combination (FDC) for pregnant and breastfeeding HIV Positive mothers |
|  |  | 2.       Antiretrovirals versus placebo (Breastefeeding and not breastfeeding) |  |  | ARVs vs placebo (Breastfeeding population) |  |  |  | If HIV status is known (NVP < 28 weeks. Triple therapy ZDV (antenatal)+ ZDV +3TC (intrapartum) + postpartum (1wk) +sdNVP (72 hours after delivery)+ZDV for the next 6 wks) |
|  |  | 3.       Longer versus shorter regimens using the same antiretrovirals (Breastfeeding and not breastfeeding) |  |  | DITRAME |  |  |  | If HIV status in unknown (no previous drugs, late presentation. PEP -single dose NVP after birth +ZDV 6 weeks |
|  |  | 4.       Antiretroviral regimens using different drugs and durations of treatment (Breastfeeding and not breastfeeding) |  |  |  |  |  |  |  |
|  |  | 5.       TRIPLE regimens versus other (Breastfeeding and not breastfeeding) |  |  |  | HIV infection (Protective) | Infant death (Protective) |  | NVP at birth and then daily for 6 weeks |
|  |  | 6.       TRIPLE regimen versus TRIPLE regimen (Breastfeeding and not breastfeeding) (Siegfried, N, 2011)40 |  |  |  | Premature delivery (Protective) | Stillbirth (Protective) |  |  |
|  |  |  |  |  |  | LBW (Protective) | Maternal death (Protective) |  | <1.0kg: 2mg/kg initially |
|  |  |  |  |  |  | Severe anaemia (Mother and child Not significant) |  |  | Birth to 6 weeks 1.0-2.5kg birth weight: 10mg/d |
|  |  |  |  |  |  | Severe neutropenia (Mother and child Not significant) |  |  | Birth to 6 weeks ≥ 2.5kg birth weight: 15mg/d |
|  |  |  |  |  |  | HIV infection (Protective) |  |  | 6 weeks to 12 weeks: 20mg |
|  |  |  |  |  |  | Congenital abnormality (Not significant) |  |  | 6 weeks to 6 months: 20mg/d |
|  |  |  |  |  |  |  |  |  | 6 months to 9 months: 30mg/d |
|  |  |  |  |  |  | Severe maternal clinical disorder (Not significant) |  |  | >9 months: 40mg/day |
|  |  |  |  |  | RETRO-CI |  | Infant death (Not significant) |  | " |
|  |  |  |  |  |  | HIV infection (Protective) | Postpartum death (Not significant) |  |  |
|  |  |  |  |  |  |  | Stillbirth (Not significant) |  |  |
|  |  |  |  |  |  |  |  |  |  |
|  |  |  |  |  |  | HIV infection (Not significant) |  |  |  |
|  |  |  |  |  |  | Frequency of grade 3 and grade 4 laboratory events for mother and child (Not significant) |  |  |  |
|  |  |  |  |  |  |  | Infant deaths (Not significant) |  |  |
|  |  |  |  |  | PETRA PETRA-a and PETRA b |  | Stillbirths (Not significant) |  |  |
|  |  |  |  |  |  |  |  |  |  |
|  |  |  |  |  |  |  | Infant death (Not significant) |  |  |
|  |  |  |  |  | PETRA-c | HIV infection (Protective) | Stillbirth (Not significant) |  |  |
|  |  |  |  |  |  |  |  |  |  |
|  |  |  |  |  |  |  |  |  |  |
|  |  |  |  |  |  | Premature deliver (Not significant) |  |  |  |
|  |  |  |  |  |  |  |  |  |  |
|  |  |  |  |  |  | LBW (Not significant) |  |  |  |
|  |  |  |  |  |  | HIV transmission (Protective) |  |  |  |
|  |  |  |  |  | ARVs vs placebo (Non- breastfeeding population) |  |  |  |  |
|  |  |  |  |  |  | For all outcomes (Not significant) | Infant death (Not significant) |  |  |
|  |  |  |  |  | PACTG 076 |  | Stillbirth (Not significant) |  |  |
|  |  |  |  |  |  |  |  |  |  |
|  |  |  |  |  |  |  | Stillbirth (Not significant) |  |  |
|  |  |  |  |  | Limpongsanur | For all outcomes (Not significant) |  |  |  |
|  |  |  |  |  |  |  | Infant deaths (Not significant) |  |  |
|  |  |  |  |  |  |  |  |  |  |
|  |  |  |  |  | THAI-CDC |  |  |  |  |
|  |  |  |  |  |  | HIV infection (Not significant) |  |  |  |
|  |  |  |  |  |  | Low birth weight (Protective) | Stillbirth (Protective) |  |  |
|  |  |  |  |  |  | Premature delivery (Protective) |  |  |  |
|  |  |  |  |  | Longer versus shorter regimens using same ARVs (breastfeeding) |  |  |  |  |
|  |  |  |  |  | Longer versus shorter regimens using same ARVs (non-breastfeeding) | Premature birth (Protective) |  |  |  |
|  |  |  |  |  | PHPT-a | HIV infection (Protective) | Infant mortality (Protective) |  |  |
|  |  |  |  |  |  | LBW (not Protective) |  |  |  |
|  |  |  |  |  |  |  |  |  |  |
|  |  |  |  |  |  |  |  |  |  |
|  |  |  |  |  | PHPT-b |  | Stillbirth (Protective) |  |  |
|  |  |  |  |  |  |  |  |  |  |
|  |  |  |  |  | Bhoopat, 2005 |  |  |  |  |
|  |  |  |  |  |  |  | Infant mortality (Protective) |  |  |
|  |  |  |  |  |  | HIV infection (Not significant) |  |  |  |
|  |  |  |  |  |  |  |  |  |  |
|  |  |  |  |  |  | HIV infection (Protective) | Stillbirths (Not significant) |  |  |
|  |  |  |  |  |  | LBW (Not significant) |  |  |  |
|  |  |  |  |  | REGIMENS USING DIFFERENT DRUGS AND DURATIONS OF TREATMENT (breastfeeding) |  |  |  |  |
|  |  |  |  |  |  | HIV infection (Protective) |  |  |  |
|  |  |  |  |  | HIVNET 012 |  |  |  |  |
|  |  |  |  |  |  |  |  |  |  |
|  |  |  |  |  | Taha 2003 |  |  |  |  |
|  |  |  |  |  |  |  |  |  |  |
|  |  |  |  |  |  |  |  |  |  |
|  |  |  |  |  |  |  |  |  |  |
|  |  |  |  |  | Taha, 2004; Thistle,2007; Chi 2004 | All outcomes (Not significant) |  |  |  |
|  |  |  |  |  | REGIMENS USING DIFFERENT DRUGS AND DURATIONS OF TREATMENT (Non- breastfeeding) |  | Infant death (Not significant) |  |  |
|  |  |  |  |  |  |  |  |  |  |
|  |  |  |  |  | PACTG 316 |  |  |  |  |
|  |  |  |  |  | All other trials yielded no significance for all outcomes |  |  |  |  |
|  |  |  |  |  |  | HIV infection (Protective) |  |  |  |
|  |  |  |  |  | TRIPLE REGIMENS VERSUS OTHER (Breastfeeding and non-breastfeeding) | All outcomes (Not significant) |  |  |  |
|  |  |  |  |  |  | All outcomes (Not significant) for all trials |  |  |  |
|  |  |  |  |  |  |  |  |  |  |
|  |  |  |  |  |  |  |  |  |  |
| **Childbirth** |  |  |  |  |  |  |  |  |  |
|  | **Antibiotic treatment during premature rupture of membranes (PROM)** | Antibiotics: penicillin; beta-lactams; or macrolides. Erythromycin versus co-moxiclav. Use of surfactant. (Kenyon, S, 2010)42 | Pregnant women with PROM before 37 weeks | Health care providers | Pharmacological | Chorioamnionitis (Protective) |  | Guidelines for maternity care in South Africa 2015 | Antibiotic treatment for Preterm Labour: |
|  |  | Comparator: placebo |  |  |  | Babies born within 48 hours (Protective) |  |  | Women who present with “Rupture of the membranes before the onset of labour (refer if no spontaneous labour within 12 hours)” during labour must be referred from community health centre (PHC centre) to a hospital. |
|  |  |  |  |  |  | Neonatal infection (Protective) |  |  |  |
|  |  |  |  |  |  | Days in neonatal ICU (Protective) |  |  | “This is defined as the onset of labour after the gestation of ≥ 24 w0d and before 37 completed weeks (37 w0d) of |
|  |  |  |  |  |  | Abnormal cerebral ultrasound scan prior to discharge from hospital (Protective) |  |  | pregnancy. Management depends on the gestational age and/or estimated fetal weight (by palpation or ultrasound). |
|  |  |  |  |  |  | Delivery within 48 hours (Protective) |  |  | The onset of labour is determined by: |
|  |  |  |  |  |  | Necrotising enterocolitis (Protective) |  |  | Documented regular uterine contractions (at least one every 10 min), with |
|  | **Antibiotic treatment during preterm premature rupture of membranes (pPROM)** | Antibiotics: Women with pre-term rupture of membranes were treated with antibiotics: ampicillin, erythromycin, penicillin, or amoxicillin, mezlocillin, piperacillin, or combinations (Cousens, S, 2010)43 | Pregnant women with pPROM | Health care providers | Pharmacological | Respiratory distress syndrome Protective) | All-cause neonatal mortality/mortality before discharge (Protective) | Refer above | Refer above |
|  |  |  |  |  |  | Necrotising enterocolitis (Not significant) |  |  |  |
|  |  | Comparator: usual care |  |  |  | Intra-ventricular hemorrhage (Protective) |  |  |  |
|  |  |  |  |  |  | Confirmed sepsis (Protective) |  |  |  |
|  |  |  |  |  |  |  |  |  |  |
|  | **Corticosteroid administration at presentation of preterm labour for accelerating fetal lung maturation** | Dexamethasone or betamethasone (various regimens, different timings and administration routes) (Brownfoot, F 2009)44 | Women with a singleton or multiple pregnancy expected to give birth preterm (before 37 weeks) as a result of either spontaneous preterm labour, preterm prelabour rupture of membranes or elective preterm birth | Hospitals by health care providers | Pharmacological |  |  | Guidelines for maternity care in South Africa 2015 | Preterm labour: “Gestational age ≥ 34 weeks (or estimated fetal weight ≥ 2 kg if gestation unknown)” “If cervix <6 cm dilated, continue tocolysis. Try and continue suppression of labour until 48 hours have passed since the first dose of β-methasone. Give steroids (preferably betamethasone 12 mg IM; repeated after 24 hours, or dexamethasone 4 mg (1 ampoule) 8 hourly for 48 hours (total dose of 24mg).”  “If the estimated fetal weight is <1500 g, transfer the mother to a hospital with neonatal intensive care facilities (specialist hospital). Gestational age <26 weeks or estimated fetal weight <900 g. Transfer from a clinic or community health centre to hospital. Allow labour to proceed. If the baby is born alive, resuscitate actively and transfer it from a clinic or community health centre to hospital.”  Prelabour Rupture Of The Membranes “Gestational age 24-33 weeks or estimated fetal weight 600 g –1 999* g 􀁸 Transfer from a clinic or community health centre to hospital. 􀁸 􀁸 Give steroids (preferably betamethasone 12 mg IM repeated after 24 hours, or dexamethasone 4 mg IM 8 hourly for 6 doses).” |
|  |  |  |  |  | Dexamethasone versus betamethasone |  |  |  |  |
|  |  | Comparator: Dexamethasone or betamethasone (various regimens, different timings and administration routes) |  |  |  | Intra-ventricular haemorrhage (Protective) | Perinatal death (Not significant) |  |  |
|  |  |  |  |  |  | Severe Intra-ventricular haemorrhage (Not significant) |  |  |  |
|  |  |  |  |  |  | NICU, lower fetal heart rate , higher breathing times, higher level of fetal movement (Dexamethasone adverse) |  |  |  |
|  |  |  |  |  | Dexamethasone (Oral vs IM) | Oral Dexamethasone (Neonatal sepsis adverse) |  |  |  |
|  |  |  |  |  |  |  |  |  |  |
|  |  |  |  |  |  | All outcomes (Not significant) |  |  |  |
|  |  |  |  |  |  |  |  |  |  |
|  |  |  |  |  |  |  |  |  |  |
|  |  |  |  |  |  |  |  |  |  |
|  |  |  |  |  |  |  |  |  |  |
|  |  |  |  |  | Betamethasone acetate + phosphate versus betamethasone phosphate |  |  |  |  |
|  | **Corticosteroid administration at presentation of preterm labour for accelerating fetal lung maturation** | Antenatal steroids administration of corticosteroids to women in preterm labour (Mwansa-Kambafile, J, 2010)45 | Pregnant women before anticipated labour (<36 weeks) in LMIC | Health care providers | Pharmacological | Morbidity (Respiratory Distress Syndrome) (Protective) | Neonatal mortality (Protective) | Refer above | Refer above |
|  |  |  |  |  |  | Preterm birth (Protective) |  |  |  |
|  |  | Comparator: placebo or no treatment |  |  |  |  |  |  |  |
|  | **Magnesium sulphate versus phenytoin as part of managing eclampsia** | Magnesium sulphate (IV or IM) - 4 g as a loading dose, and maintenance therapy was either by an intramuscular regimen or an infusion of 1 g/hour | Administered to women with eclampsia before or after delivery, irrespective of whether on an anticonvulsant) | Healthcare provider | Pharmacological | Recurrence of seizures (Protective) | Maternal mortality (Not significant) | Guidelines for maternity care in South Africa 2015 | “Severe hypertension, imminent eclampsia, eclampsia and HELLP syndrome must be recognised as life threatening conditions (Major Alerts) requiring urgent attention. All maternity facilities must be able to administer magnesium sulphate to prevent convulsions, administer rapid acting agents to lower severely raised blood pressure , provide close monitoring prior to and following delivery and manage fluid balance safely.”  “SEVERE PRE-ECLAMPSIA OR IMMINENT ECLAMPSIA The patient should be stabilised and have immediate priority transfer to a specialist hospital. 􀁸 Insert Foley catheter and plot urine output. 􀁸 Put up a drip and give RESTRICTED IV fluid – use a 200 mL Ringers lactate ensuring against fluid overload. Give 80 mL per hour. 􀁸 Initiate magnesium sulphate (MgS04) prophylaxis against the development of seizures. Dilute 4 ampoules (4 gram) in 200 mL Ringer Lactate and infuse over 20 minutes. If transfer will take longer than 4 hours, also give 5 g MgS04 deep intramuscularly in each buttock (a total dose of 14 g). For quick transfer (specialist centre close by) the 4 g IV is sufficient.” |
|  |  | (Duley, L, 2010 )46 |  |  |  |  |  |  |  |
|  |  |  |  |  |  | Cerebrovascular accident (Not significant) | Stillbirth (No clear difference) |  |  |
|  |  | Comparator: phenytoin - a loading dose of 1000 to 1500mg as a short intravenous infusion, followed by a maintenance infusion of half the loading dose 12 hours later. As phenytoin is only used for prevention of further fits, another agent (usually a benzodiazapine) may have been used for control of the acute convulsion. |  |  |  |  |  |  |  |
|  |  |  |  |  |  | Any serious morbidity (Not significant) |  |  |  |
|  |  |  |  |  |  |  | Perinatal death (No clear difference) |  |  |
|  |  |  |  |  |  | Serious maternal morbidity - Pneumonia (Protective) |  |  |  |
|  |  |  |  |  |  | Renal failure ( Not significant) | Neonatal death (No clear difference) |  |  |
|  |  |  |  |  |  |  |  |  |  |
|  |  |  |  |  |  | ICU admission (Protective) |  |  |  |
|  |  |  |  |  |  |  |  |  |  |
|  |  |  |  |  |  | Ventilation (Protective) |  |  |  |
|  |  |  |  |  |  |  |  |  |  |
|  |  |  |  |  |  | APGAR scores (Below 8 at 1 min) (Protective) |  |  |  |
|  |  |  |  |  |  |  |  |  |  |
|  |  |  |  |  |  | APGAR scores (Below 8 at 5 min) (No difference) |  |  |  |
|  |  |  |  |  |  |  |  |  |  |
|  |  |  |  |  |  | Admission to special care baby unit (Protective) |  |  |  |
|  | **Supply of clean delivery birth kits for deliveries** | The use of a birth kit defined as any disposable kit intended for routine use in the intrapartum period, specifically at the delivery of the baby primarily used at home (Hundley, V, 2012) 47 | Pregnant women who are delivering at home | Traditional birth attendants/ Skilled birth attendants/Dais/Dayas | Non-pharmacological | Omphalitis (Protective) | Early and Late neonatal mortality (Protective) | Guidelines for maternity care in South Africa 2015 | To promote safe motherhood in South Africa, “clean and safe delivery - to ensure that all health workers have the knowledge, skills and equipment to perform clean and safe delivery and provide postpartum care to mother and baby.” |
|  |  | Comparator: no kit |  |  |  |  | Tetanus related mortality (Protective) |  |  |
|  |  |  |  |  |  | Sepsis (Protective) |  |  |  |
|  |  |  |  |  |  |  |  |  |  |
|  | **Provision of skilled birth attendance (SBA) and provision of emergency obstetric care** | SBA and/or emergency obstetric care (Yakoob, M, 2011)56 | Pregnant women delivering | Skilled birth attendants | Pharmacological and non-pharmacological |  | Stillbirths (Protective) | South African National Department of Health. Guidelines For Maternity Care In South Africa. Pretoria: 2015 | “PROVISION OF SKILLED MIDWIFERY AND OBSTETRIC SERVICES The three levels of the district health care pyramid (family and community, health centre, and district hospital) must function in an efficient and cost-effective manner. Midwives and doctors are the best equipped to provide technologically appropriate care to women during their reproductive lives. To prevent maternal deaths, all hospitals must offer caesarean section and blood transfusion facilities. The practice of home deliveries, whether by professional or lay midwives, is not encouraged.”  “The practice of home delivery should be discouraged; all women should try and deliver in a facility with a skilled attendant.”  “SKILLED ATTENDANCE AT BIRTH AND MOTHERS’ WAITING AREAS Health services should make every effort to ensure skilled professional attendance at birth for all pregnant women. Mothers’ waiting areas are boarding facilities at Community Health Centres (CHCs) and District Hospitals where women with advanced pregnancy may stay until they go into labour. Such facilities should be established in rural areas and in districts where transport problems and remoteness make it difficult for women to access skilled attendants at birth.”  “POSTPARTUM HAEMORRHAGE AFTER VAGINAL DELIVERY Prevention PPH and its complications can be prevented by: 􀁸 Ensuring skilled attendance at delivery if necessary by providing maternity waiting homes in rural areas.” |
|  |  | SBA: “accredited health professional – such as a midwife, doctor or nurse – who has been educated and trained to proficiency in the skills needed to manage normal (uncomplicated) pregnancies, childbirth and the immediate postnatal period, and in the identification, management and referral of complications in women and newborns.” |  |  |  |  | Perinatal mortality (Protective) |  |  |
|  |  | Basic Emergency (or Essential) Obstetric Care (BEOC): “7 “signal functions” that include: the use of intravenous/intramuscular antibiotics, intravenous/intramuscular oxytocin, intravenous/intramuscular anticonvulsants, manual removal of retained placenta and removal of |  |  |  |  |  |  |  |
|  |  | retained products of conception (e.g. by Manual Vacuum Aspiration), assisted vaginal delivery and basic newborn resuscitation” |  |  |  |  |  |  |  |
|  |  | Comprehensive Emergency (or Essential) Obstetric Care (CEOC): “includes all BEOC signal functions plus Caesarean section and blood transfusion” |  |  |  |  |  |  |  |
|  |  | Comparators: Either usual care or strengthening referral systems |  |  |  |  |  |  |  |
|  | **Provision of traditional birth attendance (TBA)** | TBAs (Trained/untrained) trained on traditional birth attendance and maternal behaviours thought to mediate positive pregnancy outcomes. Specific training courses, over periods of time, supervision, continuing of education) | Lactating mothers, pregnant women followed through the postpartum period, and women recently delivered or referred to a health facility by TBAs, or both. | Care provided through TBAs | Non-pharmacological | PPH, Puerperal sepsis, mean blood loss (Not significant) | Stillbirths (Protective) | Refer above | Refer above |
|  |  | (Sibley, L, 2009)62 |  |  |  |  | Perinatal mortality (Protective) |  |  |
|  |  | Comparator: no training or usual training |  |  |  |  | Neonatal mortality (Protective) |  |  |
|  |  |  |  |  |  |  | Maternal death (nor significant) |  |  |
|  |  |  |  |  |  |  |  |  |  |
|  | **Different community cadres during pregnancy and childbirth (skilled birth attendants, traditional birth attendants, community health workers)** | Care by different community cadres (community-based skilled birth attendants, trained traditional birth attendants, and community health workers) during pregnancy and childbirth (Darmstadt, G, 2009)58 | Pregnant women | Traditional birth attendants, community health workers, village health workers, community health aides, skilled birth attendant/skilled attendant, birthing centre, community midwives | Pharmacological and non-pharmacological |  |  | Refer above | Refer above |
|  |  |  |  |  | Skilled birth attendants |  |  |  |  |
|  |  |  |  |  |  |  |  |  |  |
|  |  |  |  |  |  |  | Perinatal mortality (Protective) |  |  |
|  |  |  |  |  |  |  | Intrapartum-related neonatal mortality (Protective) |  |  |
|  |  |  |  |  | TBA training |  |  |  |  |
|  |  |  |  |  |  |  | Perinatal mortality (Protective) |  |  |
|  |  |  |  |  |  |  | Intrapartum-related neonatal mortality (Protective) |  |  |
|  |  |  |  |  |  |  | Perinatal mortality (Protective) |  |  |
|  |  |  |  |  | CHW packages of care |  | Early neonatal mortality rate (Protective) |  |  |
|  |  |  |  |  |  |  | Intrapartum-related neonatal mortality (Protective) |  |  |
|  |  |  |  |  |  |  |  |  |  |
|  |  |  |  |  |  |  |  |  |  |
| **Neonate** |  |  |  |  |  |  |  |  |  |
|  | Promotion of kangaroo mother care (KMC) | Comparisons of KMC with conventional neonatal care in LBW infants. Comparisons of early onset KMC (starting within 24 hours post-birth) with late onset KMC (starting after 24 hours post birth) in LBW infants (Conde-Agudelo, A, 2011)59 | Pregnant women and their low birth weight (<2500gm) infant | Health care providers | Non-pharmacological | Nosocomial infection/sepsis (Protective) | Risk of mortality (Protective) | Standard treatment guidelines and essential medicines list – hospital level 2013  Standard treatment guidelines and essential medicines list – primary level 2014 | Promoted for : Sick neonate and neonatal emergencies (primary care) Hypothermia for infants presenting with severe acute malnutrition, premature/preterm neonate (hospital) Premature/preterm neonate (hospital care) |
|  |  | Comparator: conventional neonatal care, Late onset KMC |  |  |  | Hypothermia (Protective) |  | Tshwane declaration on breastfeeding, 2011 |  |
|  |  |  |  |  |  | Length of hospital stay (Protective) |  | Strategic Plan for Maternal, Newborn, Child and Women’s Health (MNCWH) and Nutrition in South Africa 2012 – 2016 |  |
|  |  |  |  |  |  | Severe infection/sepsis (Protective) |  | Roadmap for nutrition in South Africa. 2013-2017. Pretoria: 2013 |  |
|  |  |  |  |  |  | Measures of infant growth (Protective) |  | Baby friendly health initiative, 1994 |  |
|  |  |  |  |  |  |  |  | South Africa’s National Strategic Plan For A Campaign On Accelerated Reduction Of Maternal And Child Mortality In Africa (CARMMA) 2011 |  |
|  |  |  |  |  |  |  |  |  |  |
|  | Promotion of early skin-to-skin contact (SSC) | Early SSC (naked baby between breasts of mother) | Pregnant women and their health newborn infants | Health care providers | Non-pharmacological | Cardio-respiratory stability (Protective) |  | Refer above | Refer above |
|  |  | (Moore, E, 2012)60 |  |  |  | Blood glucose (Protective) |  |  |  |
|  |  | Comparator: Routine care (swaddled infants, dressed and held in mother’s arms, placed in crib or under radiant warmers) |  |  |  | Infant body weight change (Not significant) |  |  |  |
|  |  |  |  |  |  |  |  |  |  |
|  |  |  |  |  |  |  |  |  |  |
|  |  |  |  |  |  |  |  |  |  |
|  | Vitamin A supplementation in term neonates within the first 28 days of life | Oral dose of vitamin A (50,000 IU) within 24 hours of delivery or within the first 48 hours of delivery and the second dose within 24 hours of the first dose. (Imdad A, 2010)69 | Neonates at least 2500g without any serious medical condition | Health facility by health professional | Pharmacological | Diarrhoea incidence (Protective) | All cause mortality (Protective) | WHO Guideline: Vitamin A supplementation in infants and children 6–59 months of age, 2011 | This is not done in SA. In South Africa Vitamin A is given from 6 months to 59 months preventatively. The dosage is a single dose of 100 000 IU at age 6 months, A single dose of 200 000 IU at 12 months, then a dose of 200 000 IU every 6 months up to 59 months. For curative purposes, infants below 6 months can get 50 000 IU. |
|  |  |  |  |  |  | Diarrhoea prevalence (Protective) | Mortality between 13 and 60 months(Protective) | National Vitamin A Supplementation Policy Guidelines For South Africa, 2012 | Mothers receive a single dosage of 200,00mg dosage of Vitamin A postpartum. Vitamin A given in the first 4 weeks as preventative is not given in SA only curative |
|  |  | Comparator: placebo |  |  |  | Measles incidence (Protective) | Measles mortality (Not significant) | Roadmap for nutrition in South Africa. 2013-2017. Pretoria: 2013 |  |
|  |  |  |  |  |  | Malaria prevalence (Not significant) | Meningitis mortality (Not significant) | Infant and Young Child Feeding Policy (2013) |  |
|  |  |  |  |  |  | LRTI incidence (Not significant) | LRTI mortality (Not significant) |  |  |
|  |  |  |  |  |  | LRTI prevalence (Not significant) |  |  |  |
|  |  |  |  |  |  | Bitots spots prevalence (Protective) |  |  |  |
|  |  |  |  |  |  | Night blindness (Protective) |  |  |  |
|  |  |  |  |  |  | Xeropthalmia incidence (Not significant) |  |  |  |
|  |  |  |  |  |  | Xeropthalmia prevalence (Protective) |  |  |  |
|  |  |  |  |  |  | Number of VAD children (Protective) |  |  |  |
|  |  |  |  |  |  | Vitamin A serum levels (Protective) |  |  |  |
|  | Vitamin A supplementation in term neonates within the first 28 days of life | Supplementation with vitamin A within the first 28 days of life (Haider B, 2011)62 | All term neonates (born between 37 to 42 weeks of gestational age) up to 28 days after birth were included. | Health facility by health professional | Pharmacological | Diarrhoea (Not significant) | risk of infant mortality at six months (Protective) | Refer above | Refer above |
|  |  | Comparator: control (placebo or no supplementation). |  |  |  | Acute Respiratory (Not significant) | infant mortality at 12 months of age (Not significant) |  |  |
|  |  |  |  |  |  |  |  |  |  |
| **Postnatal** | Antidepressants to prevent postnatal depression | Antidepressants: Nortyptiline or sertraline (Howard)65 | In non-depressed women, or those who gave birth within the previous 6 weeks | Health care providers | Pharmacological | Recurrence of postpartum (Not significant) |  | As above for depression | As above for depression |
|  |  |  |  |  |  | major depressive disorder (Not significant) |  |  |  |
|  |  | Comparator: placebo |  |  |  | Rate of recurrence (Not significant) |  |  |  |
|  |  |  |  |  |  | Time to recurrence (Not significant) |  |  |  |
|  |  |  |  |  |  | Recurrence of postpartum major depressive disorder (Protective) |  |  |  |
|  |  |  |  |  |  | Time to recurrence (Protective) |  |  |  |
|  | Antiretrovials to treat HIV/AIDS in children < 3 years | The following types of intervention were assessed: | Perinatally HIV-infected children under 3 years of age | Healthcare providers | Pharmacological | Mortality or disease progression (Protective) |  | National Department of Health, National consolidated guidelines for the prevention of mother-to-child transmission of HIV (PMTCT) and the management of HIV in children, adolescents and adults, December 2015. | Abacavir + lamivudine + lopinavir/ritonavir |
|  |  | - Timing of treatment: use of early compared to deferred ART |  |  |  | median CD4 cell count (Not significant) |  | Strategic Plan for Maternal, Newborn, Child and Women’s Health (MNCWH) and Nutrition in South Africa 2012 - 2016 |  |
|  |  | - Choice of treatment: use of NNRTI- versus PI-based regimens, in combination with any NRTI backbone |  |  |  | Treatment failure at 24 weeks, virological failure at 24 weeks and larger difference in time to virological failure (or death) (Protective for NVP and not LPV/r based regimens when ART was initiated in the first year of life.) |  |  |  |
|  |  | - Substitution of LPV/r with NVP following initiation with PIbased regimens. |  |  |  | Greater CD4 rise and better viral load response at 24 weeks (4-drugNNRTI-based regimen for 36 weeks, followed by 3-drug ART. (More Protective than standard 3-drug NNRTI-based ART at 36 weeks) |  |  |  |
|  |  | - Interruption of treatment, compared to continuous early ART |  |  |  |  |  |  |  |
|  |  | Induction-maintenance treatment: Initiating ART with more than 3 antiretroviral drugs for an induction period, then moving to maintenance treatment with a standard 3-drug regimen (Penazzato, M, 2014) 66 |  |  |  |  |  |  |  |
|  |  | Comparators mention with intervention |  |  |  |  |  |  |  |
| **Across the first 1000 days categories** | | | | | | | | | |
|  | **Lay health care worker (LHW) in primary and community health care** | LHWs (Paid or voluntary) – community health workers, village health corkers, birth attendants, peer counsellors, nutrition workers, home visitors (Lewin, S, 2010)71 | Mothers, neonates and children | LHWs provide care for a broad range of health issues such as maternal and child health and the management of infectious diseases. | Non-pharmacological | Child morbidity (Fever, acute respiratory infection) (Protective) | Children mortality <5yrs (Not significant) | Promoted in the following: | Four antenatal visits starting at 14 weeks are to be scheduled in the women’s home and pregnancy care, PMTCT promotion, encouragement and follow up as to whether women attended ANC and identifications of the risks and danger signs of pregnancy, including birth preparedness and planning are promoted. Checklists and structured referral forms are to be used by the teams. Four postnatal visits starting 24 hours after returning home up until 14 days thereafter focuses on the provision of essential postnatal care for the mother and related complications; essential care for the neonate and PMCTC oversight. Through the Integrated Management of Childhood Illness (IMCI) programme growth and nutrition monitoring are promoted. Vitamin A supplementation is done by CHWs as well. |
|  |  |  |  |  |  |  | Neonates (Not significant) | National Department of Health, PHC Outreach team toolkit, Pretoria: 2011. |  |
|  |  | Comparator: usual care |  |  |  |  |  | Roadmap for nutrition in South Africa. 2013-2017. Pretoria: 2013 |  |
|  |  |  |  |  |  |  |  | Strategic Plan for Maternal, Newborn, Child and Women’s Health (MNCWH) and Nutrition in South Africa 2012 – 2016 |  |
|  |  |  |  |  |  |  |  | Reducing Maternal and Child Mortality Through Strengthening Primary Healthcare (RMCHSA). Standard Operating Procedure (SOP) for Family Planning for use by Community Health Workers. 2013. |  |
|  |  |  |  |  |  |  |  | Provincial Guidelines For The Implementation Of The Three Streams Of PHC Re-Engineering, 2011 |  |
|  | Community-based intervention packages using community health workers | Additional MNCHW training (over and above usual training) of outreach workers in the form of lectures, supervised hands-on training in healthcare facility and/or within community (Lassi, Z, 2010)72 | Women of reproductive age group, particularly pregnant women at any period of gestation. | Through provision of community-based intervention packages for preventing maternal and newborn illness and death. | Non-Pharmacological | Overall | Maternal mortality (No significant) | Refer above | Refer above |
|  |  | Training TBA who made home visits during anenatal period and delivery |  |  |  |  |  |  |  |
|  |  |  |  |  |  |  |  |  |  |
|  |  | Community based outreach workers |  |  |  |  |  |  |  |
|  |  |  |  |  |  |  |  |  |  |
|  |  | Building support through home visitations and community mobilization |  |  |  |  | Maternal mortality (Protective) |  |  |
|  |  | Community mobilization and home-based neonatal treatment |  |  |  |  |  |  |  |
|  |  | Home-based care and sepsis management as part of package |  |  |  |  |  |  |  |
|  |  | TBAs trained and home visits |  |  |  |  | Neonatal mortality (Positive) |  |  |
|  |  | Overall |  |  |  |  |  |  |  |
|  |  | Community mobilization and antenatal and post natal visits |  |  |  |  | Neonatal mortality (Protective) |  |  |
|  |  | Community support groups |  |  |  |  |  |  |  |
|  |  | Home-based care |  |  |  |  | Neonatal mortality (Protective) |  |  |
|  |  | TBAs made home visits antenatally and intrapartum |  |  |  |  |  |  |  |
|  |  |  |  |  |  |  |  |  |  |
|  |  |  |  |  |  |  | Neonatal deaths (Protective) |  |  |
|  |  |  |  |  |  |  |  |  |  |
|  |  |  |  |  |  |  | Neonatal mortality (No effect) |  |  |
|  |  |  |  |  |  |  | Early neonatal deaths (Protective) |  |  |
|  |  |  |  |  |  |  | Early neonatal deaths (Protective) |  |  |
|  |  |  |  |  |  |  | Early neonatal deaths (Protective) |  |  |
|  |  | Comparator: Usual maternal and newborn care services from local government and non-government facilities. |  |  |  |  | Early neonatal deaths (Protective) |  |  |
|  |  |  |  |  |  |  | Early neonatal deaths (No effect) |  |  |
|  |  |  |  |  |  |  | Late neonatal mortality (Protective) |  |  |
|  |  |  |  |  |  |  |  |  |  |
|  |  |  |  |  |  |  |  |  |  |
|  | **Community-level interventions** | Interventions delivered in the community (home, school, local clinic; delivered by any person within the community (health personnel/lay person); primary care setting) (Kidney, E, 2009)73 | Pregnant women or women of childbearing age (15-49 years) participating in the community intervention | Community settings | Non-pharmacological |  | Maternal mortality (Protective) 2 Studies | Refer above | Refer above |
|  |  |  |  |  |  |  |  |  |  |
|  |  | Minimal goal-oriented versus usual antenatal care |  |  |  |  |  |  |  |
|  |  | Comparator: Comparable populations experiencing either "usual care", including hospital based care, or other community interventions |  |  |  |  |  |  |  |
|  |  |  |  |  |  |  |  |  |  |
|  |  |  |  |  |  |  |  |  |  |
|  |  |  |  |  |  |  | Maternal mortality ( No effect) |  |  |
|  |  |  |  |  |  |  |  |  |  |
|  | **Hand washing with soap** | Using soap to wash hands in various settings in the community (urban, rural, childcare, refugee camp settings) (Curtis, V, 2003)75 | Adults and children | Promoting hand washing with soap in communities | Non-pharmacological | Diarrhoea (Protective |  | Roadmap for nutrition in South Africa. 2013-2017. Pretoria: 2013 | Health Minister Dr Aaron Motsoaledi, in partnership with the Consumer Goods Council of South Africa (CGCSA) launched the Public Hand Hygiene Campaign in November 2014 to raise awareness on the importance of hand-washing in the prevention of infectious diseases. This campaign seeks to educate members of the public, corporate and government institutions on the importance of hand hygiene as an important measure to prevent diseases and a practical infection control mechanism. |
|  |  |  |  |  |  | Severe enteric infections (Protective |  | National Integrated Plan For Early Childhood Development In South Africa 2005–2010 |  |
|  |  | Soap and hand washing with or without lessons. Studies were set in childcare facilities, or domestic hand washing practice. Many different types of, and occasions for, hand washing were recorded including washing by child-carers, by children, and by adult study respondents. Hand wash occasions reported included: after defecation or after the toilet, after cleaning up a child or handling dirty nappies, before eating, and before preparing or handling food. In some studies soap was not specified. (Curtis, V, 2003) |  |  |  |  |  | Provinces have also created their own hand washing policies (seen in the western cape and Kwazulu-Natal) |  |
|  |  |  |  |  |  |  |  |  |  |
|  |  |  |  |  |  | Shigellosis (Protective) |  |  |  |
|  | **Improved water quality** | Interventions to improve the microbial quality of drinking water for preventing diarrhea at water source and; household (improved water storage, approaches for treating water in the, chlorination, solar disinfection, filtration, or combined flocculation and disinfection). (Clasen, T, 2007)77 | Residents living in the community | Environmental interventions at the water source or collection point | Pharmacological | <5yr Diarrhoea (Not significant) |  | Department of Water and Forestry. Drinking Water Quality Framework for South Africa. Pretoria: 2006 | In recognition of the importance of safe drinking water to public health, the Department of Water Affairs and Forestry (DWAF) initiated a project to draft a Drinking Water Quality Framework for South Africa to enable effective management of drinking water quality and the protection of public health. The Framework is based on a preventative risk management approach, which is comprehensive from catchment to consumer. |
|  |  | Comparator: Not clearly defined |  |  |  |  |  |  | " |
|  |  |  |  |  |  |  |  |  |  |
|  |  |  |  |  |  |  |  |  |  |
|  |  |  |  |  |  | <5yrs (Not significant) |  |  |  |

**Appendix 2** – Table showing data summary

| **Pre-Pregnancy** | | | | | | | | | | | | | | | | |
| --- | --- | --- | --- | --- | --- | --- | --- | --- | --- | --- | --- | --- | --- | --- | --- | --- |
| **Topic** | **Author** | **Year** | **Objective** | **Morbidity** | | | | | | **Mortality** | | | | | | **Comments** |
| Birth spacing and risk of adverse perinatal outcomes: a meta-analysis | Conde-Agudelo, A **27.** | 2006 | To examine the association between birth spacing and relative risk of adverse perinatal outcomes | Inter-pregnancy intervals < 6 months compared to intervals of 18-23 months | | Pre-term birth | | OR 1.40; 95% CI, (1.24-1.58), I2, 69% | | Association between birth spacing and risk of fetal and neonatal death for intervals < 6 months and > 50 months | | Fetal | | Evidence of increased risk |  | Heterogeneity quite high between studies. |
|  |  |  |  | (pooled adjusted analysis) | | LBW | | OR 1.61; 95% CI, (1.39-1.86, I2, 87%) | |  |  |  |  |  |  |  |
|  |  |  |  |  | |  |  |  |  |  |  | Neonatal | | Evidence of increased risk |  |  |
|  |  |  |  |  | | SGA | | OR 1.26; 95% CI, (1.18-133), I2;, 89%) | |  |  |  |  |  |  |  |
|  |  |  |  | Inter-pregnancy intervals > 60 months compared to intervals of 18-23 months | | Pre-term birth | | OR 1.20; 95% CI, (1.17-1.24), I2,95% | |  | |  | |  |  |  |
|  |  |  |  | (pooled adjusted analysis) | | LBW | | OR 1.43; 95% CI, (1.27-1.62), I2, 84% | |  |  |  |  |  |  |  |
|  |  |  |  | (pooled analysis) | | SGA | | OR 1.29; 95% CI, (1.20-1.39), I2, 88% | |  |  |  |  |  |  |  |
|  |  |  |  |  | | LBW | | 3.25 (3.09-3.41) | |  |  |  |  |  |  |  |
|  |  |  |  |  | | SGA | | 1.52 (1.40-1.64) | |  |  |  |  |  |  |  |
|  |  |  |  | Increase in risk for each month that the inter-pregnancy interval was lengthened from 59 months, increase (95% CI) | | Pre-term | | 0.55 (0.49-0.61) | |  | |  | |  |  |  |
|  |  |  |  |  |  | LBW | | 0.91 (0.83-0.99) | |  |  |  |  |  |  |  |
|  |  |  |  |  |  | SGA | | 0.76 (0.71-0.81) | |  |  |  |  |  |  |  |
| **Antenatal** |  |  |  |  | |  | |  | |  | |  | |  |  |  |
| Alternative versus standard packages of antenatal care for low-risk pregnancy | Dowswell, T **28** | 2010 | To compare the effects of antenatal care programmes providing a reduced number of antenatal care visits for low-risk women with programmes providing the standard schedule of visit, and to assess the views of the care providers and the women receiving antenatal care. | Pre-eclampsia | |  | | RR 0.95; 95% CI, (0.80-1.12) | | Maternal mortality | |  | | RR 1.13; 95% CI, (0.50-2.57) |  |  |
|  |  |  |  |  |  |  |  |  |  |  |  |  |  |  |  |  |
|  |  |  |  |  |  |  |  | (similar levels of hypertensive disorders) | |  |  |  |  | (No stat sign diff between groups) |  |  |
|  |  |  |  | Preterm births | | Overall | | RR 1.02; 95% CI, (0.94-1.11) | | Perinatal mortality | |  | | RR 1.14; 95% CI, (1.00-1.31) |  |  |
|  |  |  |  |  |  |  |  |  |  |  |  |  |  |  |  |  |
|  |  |  |  |  |  |  |  | No clear difference | |  |  |  |  | (increased for those randomised to reduced visits group; difference borderline stat sign) |  |  |
|  |  |  |  | Vaginal bleeding | |  | | RR 1.13; 95% CI, (0.92-1.40) | |  | | LMIC | | RR 1.15; 95% CI, (1.01-1.32) |  |  |
|  |  |  |  |  |  |  |  |  |  |  |  |  |  |  |  |  |
|  |  |  |  |  |  |  |  | No clear difference. Interpret with caution as no clear definition. | |  |  |  |  | Higher in reduced visits group |  |  |
|  |  |  |  | C-section | |  | | RR 0.99; 95% CI, (0.91-1.08) | |  | |  | |  |  |  |
|  |  |  |  |  |  |  |  |  |  |  |  |  |  |  |  |  |
|  |  |  |  |  |  |  |  | Similar numbers underwent C- section | |  |  |  |  |  |  |  |
|  |  |  |  |  |  |  |  |  |  |  |  |  |  |  |  |  |
|  |  |  |  |  |  |  |  |  |  |  |  |  |  |  |  |  |
|  |  |  |  | Induction of labour | |  | | RR 0.97; 95% CI, (0.86-1.09) | |  | |  | |  |  |  |
|  |  |  |  | LBW (<2500g) | |  | | RR 1.04 (0.97-1.11) | |  | |  | |  |  |  |
|  |  |  |  | Admitted ICU | |  | | HIC – No difference in two groups | |  | |  | |  |  |  |
|  |  |  |  |  |  |  |  | LIC – RR 0.86; 95% CI (0.74 – 0.99) Effect was favourable | |  |  |  |  |  |  |  |
| Effect of multiple micronutrient supplementation during pregnancy on maternal and birth outcomes | Haider, B **30** | 2011 | To evaluate the evidence of the impact of multiple micronutrient supplements during pregnancy, in comparison with standard iron-folate supplements, on specific maternal and pregnancy outcomes of relevance to the Lives Saved Tool (LiST). | Maternal anaemia in 3rd Trimester | |  | | RR 1.03; 95% CI, (0.87-1.22) 4 RCTs no significant effect | | Neonatal | |  | | RR1.05; 95% CI, (0.92-1.19) Not significant in 9 RCTs |  |  |
|  |  |  |  | SGA | |  | | RR 0.91; 95% CI, (0.86 -0.96) Significant in 14 studies | |  |  | Birth at home | | RR 1.47; 95% CI, (1.13-1.92) |  |  |
|  |  |  |  |  |  |  |  |  |  |  |  | ≥60% of births occurring in facility | | RR 0.94; 95% CI, (0.81-1.09) |  |  |
| The effect of folic acid, protein energy and | Imdad, A 31 | 2011 | To assess the evidence of | Peri-conceptual folic acid supplementation | | NTD | | RR 0.38; 95% CI, (0.29-051) | |  | |  | |  |  |  |
| multiple micronutrient supplements in |  |  | the impact of three different nutritional interventions |  |  |  |  |  |  |  |  |  |  |  |  |  |
| pregnancy on stillbirths |  |  | during pregnancy on stillbirths |  |  |  |  |  |  |  |  |  |  |  |  |  |
|  |  |  |  |  |  |  |  |  |  |  |  |  |  |  |  |  |
|  |  |  |  |  |  |  |  |  |  |  |  |  |  |  |  |  |
|  |  |  |  |  |  |  |  |  |  |  |  |  |  |  |  |  |
|  |  |  |  |  |  |  |  |  |  |  |  |  |  |  |  |  |
|  |  |  |  | 7 studies : Peri-conceptual folic acid supplementation | |  | |  | |  | |  | |  |  |  |
|  |  |  |  | 11 studies: fortification of food with folic acid | |  | |  | |  | |  | |  |  |  |
|  |  |  |  | Folic acid fortification | | NTD | | RR 0.59; 95% CI, (0.52-0.68) | | Folic acid fortification | | Still births | | RR 0.41; 95% CI, (0.16-1.07) |  |  |
|  |  |  |  | Balanced protein energy supplementation during pregnancy | |  | |  | |  | | Still births | | RR 0.55 (0.31-0.97) |  |  |
|  |  |  |  |  |  |  |  |  |  |  |  |  |  | Low level |  |  |
|  |  |  |  | MMN Supplements | |  | |  | |  | | Still births | | RR 0.98 (0.88-1.10) |  |  |
|  |  |  |  |  |  |  |  |  |  |  |  |  |  | Not significant |  |  |
|  |  |  |  |  | |  | |  | |  | | Perinatal mortality | | RR 1.07 (0.92-1.25) |  |  |
|  |  |  |  |  |  |  |  |  |  |  |  |  |  | Not significant |  |  |
| Energy and protein intake in pregnancy | Kramer, S **32** | 2007 | To assess the advice to increase or reduce energy or protein intake, or actual energy or protein energy supplementation or restriction, during pregnancy on energy and protein intakes, gestational weight gain, and the outcome of pregnancy | Nutritional advice to increase energy and protein intakes (5 trials; 1134 women) | | For energy intake | | WMD 105.61 (-18.94-230.15kcal/day) | | Nutritional advice to increase energy and protein intakes (4 trials) | | Stillbirth | | RR 0.55 (0.31-0.97) |  | Not sure what the comparators are: seems to be trials where actual PES is given. |
|  |  |  |  |  |  |  |  |  |  |  |  |  |  |  |  |  |
|  |  |  |  |  |  |  |  |  |  |  |  |  |  | Reduced |  |  |
|  |  |  |  |  | | For protein | | WMD 17.99 (-1.48-37.45g/day) | |  | | Neonatal death | | RR 0.62 (0.37-1.05) |  |  |
|  |  |  |  |  |  |  |  |  |  |  |  |  |  |  |  |  |
|  |  |  |  |  | | Preterm birth | | RR 0.46 (0.21-0.98) | |  | |  | |  |  | Significant reduction (associated with advice) |
|  |  |  |  |  | | Mean gestational age | | WMD -0.10 (-0.48-0.28), week | |  | |  | |  |  |  |
|  |  |  |  |  | | Pre-eclampsia | | RR 0.89 (0.42-1.88) | |  | |  | |  |  | 1 study |
|  |  |  |  | Balanced energy/protein supplementation (13 trials; 4665) | | Gestational weight gain | | WMD 20.74 (1.46-40.02g/week) | |  | |  | |  |  | Significant heterogeneity in results |
|  |  |  |  |  |  |  |  |  |  |  |  |  |  |  |  |  |
|  |  |  |  |  |  |  |  | (small but significant increase with supplementation) | |  |  |  |  |  |  |  |
|  |  |  |  |  | | Mean birth weight | | WMD 37.62 (-0.21-75.45), g | |  | |  | |  |  | Increase |
|  |  |  |  |  | | Birth length, head circumference | | No statistical difference | |  | |  | |  |  |  |
|  |  |  |  |  | | SGA | | RR 0.68 (0.56-0.84) | |  | |  | |  |  | Substantially reduced; see comment below table* $ |
|  |  |  |  | High protein supplementation (2 trials, 1076 women) | | Weekly maternal weight gain | | WMD 2.98 (-33.03-38.99), g/week | | High protein supplementation (1 trials) | | Neonatal death | | RR 2.78 (0.75-10.36) |  |  |
|  |  |  |  |  |  |  |  |  |  |  |  |  |  |  |  |  |
|  |  |  |  |  |  |  |  | No difference in weekly maternal weight gain | |  |  |  |  | (nonsignificant increase) |  |  |
|  |  |  |  |  | | Birth weight | | WMD -58.37 (-146.23-29.50) | |  | |  | | RR 0.81 (0.31-2.15) |  |  |
|  |  |  |  |  |  | (effect on fetal growth | |  | |  |  |  |  |  |  |  |
|  |  |  |  |  |  |  | | No difference | |  |  |  |  | Small nonsignificant reduction |  |  |
|  |  |  |  | Isocaloric protein supplementation (3 trials, 966 women) | | Weekly maternal weight gain | | WMD 51.68 (-75.06-178.42), g/week | |  | | Insufficient data | |  |  | Significant heterogeneity) |
|  |  |  |  |  |  |  |  |  |  |  |  | Maternal health outcomes not reported | |  |  |  |
|  |  |  |  |  | | Mean birthweight | | WMD 33.45 (-157.88-224.77), g | |  | |  | |  |  |  |
|  |  |  |  |  | | SGA (1 trial) | | RR 1.35 (1.12-1.61) | |  | |  | |  |  |  |
|  |  |  |  |  |  |  |  |  |  |  |  |  |  |  |  |  |
|  |  |  |  |  |  |  |  | (small increased risk) | |  |  |  |  |  |  |  |
|  |  |  |  | Energy/protein restriction in women with overweight or high weight gain (3 trials, 384) | | Energy restriction on maternal weight gain | | WMD -254.81 (-436.56—73.06), g/week | |  | |  | |  |  |  |
|  |  |  |  |  |  |  |  |  |  |  |  |  |  |  |  |  |
|  |  |  |  |  |  | (significant reduction in weight gain) | |  |  |  |  |  |  |  |  |  |
|  |  |  |  |  | | Proteinuric pre-eclampsia or pregnancy-induced hypertension (with or without proteinuria) | | No effect | |  | |  | |  |  |  |
|  |  |  |  |  |  |  |  |  |  |  |  |  |  |  |  |  |
|  |  |  |  |  |  |  |  | Small number of trials and participants provide insufficient evidence | |  |  |  |  |  |  |  |
| Daily oral iron supplementation during pregnancy (Review) | Peña-Rosas, J **38** | 2012 | To assess the effects of daily oral supplements for pregnant women, either alone or in conjunction with folic acid, or with other vitamins and minerals as a public health intervention | Any supplements containing iron versus same | | LBW (less than 2500 g) | | (RR) | |  | | Neonatal death | | No clear evidence of any difference between groups |  |  |
|  |  |  |  | supplements without iron or no treatment/placebo | |  |  | 0.81; 95% (CI) 0.68 to 0.97 | |  |  |  |  | (average RR 0.90; 95% CI 0.68 to 1.19) |  |  |
|  |  |  |  |  |  |  |  |  |  |  |  |  |  |  |  |  |
|  |  |  |  |  | | Birth weight | | MD = 30.81 g (95% CI 5.94 to 55.68) | |  | | Maternal mortality | | No difference |  |  |
|  |  |  |  |  | | Premature birth (less than 37 weeks’ gestation) | | RR 0.88; 95% CI 0.77 to 1.01 | |  | |  | |  |  |  |
|  |  |  |  |  |  |  |  | Not signfiicant | |  |  |  |  |  |  |  |
|  |  |  |  |  |  |  |  |  |  |  |  |  |  |  |  |  |
|  |  |  |  |  |  |  |  | ). | |  |  |  |  |  |  |  |
|  |  |  |  |  | | Congenital anomalies | | RR0.86; 95% CI 0.55 to 1.35 | |  | |  | |  |  |  |
|  |  |  |  |  |  |  |  | No clear evidence of any | |  |  |  |  |  |  |  |
|  |  |  |  |  |  |  |  | difference between groups | |  |  |  |  |  |  |  |
|  |  |  |  |  |  |  |  |  |  |  |  |  |  |  |  |  |
|  |  |  |  |  | | Maternal anaemia at term (Hb less than 110 g/L at 37 weeks’ | | RR 0.30; 95% CI 0.19 to 0.46 | |  | |  | |  |  |  |
|  |  |  |  |  |  | gestation or more) | | Substantial heterogeneity | |  |  |  |  |  |  |  |
|  |  |  |  |  |  |  | | (heterogeneity: T² | |  |  |  |  |  |  |  |
|  |  |  |  |  |  |  | | = 0.40, I² = 80%, Chi² test for heterogeneity P < 0.00001. | |  |  |  |  |  |  |  |
|  |  |  |  |  | | Maternal iron deficiency at term (based on any indicator of iron status at 37 weeks’ | | RR 0.43; 95% | |  | |  | |  |  |  |
|  |  |  |  |  |  | gestation or more) | | CI (0.27 to 0.66) | |  |  |  |  |  |  |  |
|  |  |  |  |  |  |  |  |  |  |  |  |  |  |  |  |  |
|  |  |  |  |  | | Maternal iron deficiency anaemia at term (Hb below 110 g/L | | Average RR 0.33; | |  | |  | |  |  |  |
|  |  |  |  |  |  | and at least one additional laboratory indicator at 37 weeks’ | | 95% CI (0.16 to 0.69) | |  |  |  |  |  |  |  |
|  |  |  |  |  |  | gestation or more) | |  | |  |  |  |  |  |  |  |
|  |  |  |  |  |  |  |  |  |  |  |  |  |  |  |  |  |
|  |  |  |  |  | | Severe (Hb < 70/L) anaemia at any time during the second or | | RR 0.22; 95%CI 0.01 to 3.20 | |  | |  | |  |  |  |
|  |  |  |  |  |  | third trimester | |  |  |  |  |  |  |  |  |  |
|  |  |  |  |  | | Infection during pregnancy | | RR 1.16; 95% CI 0.83 to 1.63 | |  | |  | |  |  |  |
|  |  |  |  |  |  |  |  | No difference | |  |  |  |  |  |  |  |
|  |  |  |  |  | | Placental malaria and parasitaemia | | No difference | |  | |  | |  |  |  |
|  |  |  |  |  | | Very premature birth (less than 34 weeks’ gestation) | | RR 0.51; 95% CI | |  | |  | |  |  |  |
|  |  |  |  |  |  |  |  | 0.29 to 0.91 | |  |  |  |  |  |  |  |
|  |  |  |  |  | | Infant ferritin concentration at six months in μg/L | | MD: 11.00 (95% CI 4.37 to 17.63) | |  | |  | |  |  |  |
|  |  |  |  |  |  |  |  |  |  |  |  |  |  |  |  |  |
|  |  |  |  |  | | Very | | No evidence of statistical significance | |  | |  | |  |  |  |
|  |  |  |  |  |  | LBW; infant SGA mean Apgar | |  |  |  |  |  |  |  |  |  |
|  |  |  |  |  |  | score or low Apgar score at five minutes; mean infant Hb levels at | |  |  |  |  |  |  |  |  |  |
|  |  |  |  |  |  | three and six months; admission to special care; head circumference | |  |  |  |  |  |  |  |  |  |
|  |  |  |  |  |  | at birth; stunting at long-term follow-up, and breast feeding | |  |  |  |  |  |  |  |  |  |
|  |  |  |  |  |  | at four months | |  |  |  |  |  |  |  |  |  |
|  |  |  |  |  | | Maternal Hb concentration at or near term (in g/L, at 34 | | (MD 8.88 g/L; 95% CI 6.96 | |  | |  | |  |  |  |
|  |  |  |  |  |  | weeks’ gestation or more) and within six weeks postpartum | | to 10.80) (heterogeneity: T² = 13.92, I² = 87%, Chi² test for | |  |  |  |  |  |  |  |
|  |  |  |  |  |  | period (in g/L) | | heterogeneity P < 0.00001 | |  |  |  |  |  |  |  |
|  |  |  |  |  |  |  | | At 6 weeks post partum MD 7.61; 95% CI 5.50 to 9.72; reported | |  |  |  |  |  |  |  |
|  |  |  |  |  |  |  | | in seven studies with 956 women) (heterogeneity: T² = 3.09, I² = | |  |  |  |  |  |  |  |
|  |  |  |  |  |  |  | | 40%, Chi² test for heterogeneity P = 0.12 | |  |  |  |  |  |  |  |
|  |  |  |  |  | | Maternal high Hb concentrations (Hb greater than 130 g/L) | | RR 2.26; 95% CI 1.40 to 3.66). | |  | |  | |  |  |  |
|  |  |  |  |  |  | at any time during second or third trimester | | There was high heterogeneity for this outcome (heterogeneity: T² | |  |  |  |  |  |  |  |
|  |  |  |  |  |  |  | | = 0.44, I² = 89%, Chi² test for heterogeneity P < 0.00001, PI 0.44 | |  |  |  |  |  |  |  |
|  |  |  |  |  |  |  | | to 11.54 ) | |  |  |  |  |  |  |  |
|  |  |  |  |  | | Maternal high Hb concentrations at term (defined as Hb | | RR 3.08; 95% CI 1.28 to 7.41 | |  | |  | |  |  |  |
|  |  |  |  |  |  | greater than 130 g/L at 37 weeks’ gestation or more) | |  |  |  |  |  |  |  |  |  |
|  |  |  |  |  |  |  |  |  |  |  |  |  |  |  |  |  |
|  |  |  |  |  | | Maternal severe anaemia at or near term (Hb less than 70 g/L | | No significant difference RR 0.47; 95% CI | |  | |  | |  |  |  |
|  |  |  |  |  |  | at 34 weeks’ gestation or more) | | 0.01 to 44.11 | |  |  |  |  |  |  |  |
|  |  |  |  |  |  |  |  |  |  |  |  |  |  |  |  |  |
|  |  |  |  |  | | Severe anaemia at postpartum (Hb less than 80 g/L) | | RR 0.04; 95% CI 0.01 to 0.28 | |  | |  | |  |  |  |
|  |  |  |  |  |  |  |  |  |  |  |  |  |  |  |  |  |
|  |  |  |  |  | | Transfusion provided | | RR 0.61; 95%CI 0.38 to 0.9 | |  | |  | |  |  |  |
|  |  |  |  |  |  |  |  |  |  |  |  |  |  |  |  |  |
|  |  |  |  |  | | Puerperal infection | | RR 0.68; 95% CI 0.50 to 0.92 | |  | |  | |  |  |  |
|  |  |  |  |  |  |  |  |  |  |  |  |  |  |  |  |  |
|  |  |  |  |  | | Ante- or postpartum | | No statistical significance | |  | |  | |  |  |  |
|  |  |  |  |  |  | haemorrhage, individual side effects, placental abruption, | |  |  |  |  |  |  |  |  |  |
|  |  |  |  |  |  | preterm rupture of the membranes, pre-eclampsia and moderate anaemia in the postpartum period | |  |  |  |  |  |  |  |  |  |
|  |  |  |  | Any supplements containing iron and folic acid | | LBW (less than 2500 g) | | RR1.07; | |  | | Neonatal death | | No significant difference |  |  |
|  |  |  |  | versus same supplements without iron nor folic acid | |  |  | 95% CI 0.31 to 3.74) | |  |  |  |  |  |  |  |
|  |  |  |  | (no iron nor folic acid or placebo) | |  |  | No significant differences | |  |  |  |  |  |  |  |
|  |  |  |  |  | | Infant birth weight | | (MD 57.73; 95% CI 7.66 to 107.79) | |  | |  | |  |  |  |
|  |  |  |  |  | | Premature birth (less than 37 weeks’ gestation) | | No difference | |  | |  | |  |  |  |
|  |  |  |  |  |  |  |  |  |  |  |  |  |  |  |  |  |
|  |  |  |  |  | | Congenital anomalies | | RR0.70; 95%CI 0.35 to 1.40) | |  | |  | |  |  |  |
|  |  |  |  |  |  |  |  |  |  |  |  |  |  |  |  |  |
|  |  |  |  |  | | Maternal anaemia at term (Hb less than 110 g/L at 37 weeks’ | | RR 0.34; 95% CI 0.21 to | |  | |  | |  |  |  |
|  |  |  |  |  |  | gestation or more) | | 0.54 | |  |  |  |  |  |  |  |
|  |  |  |  |  |  |  |  |  |  |  |  |  |  |  |  |  |
|  |  |  |  |  | | Maternal iron deficiency at term | | 95% CI 0.06 to 0.99. not statistically significance | |  | |  | |  |  |  |
|  |  |  |  |  | | Maternal iron deficiency anaemia at term | | RR 0.43; 95% CI 0.17 to 1.09 | |  | |  | |  |  |  |
|  |  |  |  |  |  |  |  | No evidence | |  |  |  |  |  |  |  |
|  |  |  |  |  | | Severe anaemia at any time | | No significant difference | |  | |  | |  |  |  |
|  |  |  |  |  | | Maternal Hb concentration at term (L at 37 weeks’ | | MD 16.13 g/L; 95% | |  | |  | |  |  |  |
|  |  |  |  |  |  | gestation or more) | | CI 12.74 to 19.52 | |  |  |  |  |  |  |  |
|  |  |  |  |  |  |  |  |  |  |  |  |  |  |  |  |  |
|  |  |  |  |  |  |  | | MD 17.10; 95% CI 8.44 to 25.76 | |  |  |  |  |  |  |  |
|  |  |  |  |  | | Maternal high Hb concentrations at term (defined as Hb | | No significant difference | |  | |  | |  |  |  |
|  |  |  |  |  |  | greater than 130 g/L) | |  |  |  |  |  |  |  |  |  |
|  |  |  |  |  |  |  |  |  |  |  |  |  |  |  |  |  |
|  |  |  |  |  | | Maternal Hb concentration within six weeks postpartum in | | MD =10.07; 95% | |  | |  | |  |  |  |
|  |  |  |  |  |  | g/L | | CI 7.33 to 12.81 | |  |  |  |  |  |  |  |
|  |  |  |  |  |  |  |  |  |  |  |  |  |  |  |  |  |
|  |  |  |  |  | | Maternal severe or moderate anaemia at postpartum (Hb less | | RR 0.33; 95%CI 0.17 to 0.65 | |  | |  | |  |  |  |
|  |  |  |  |  |  | than 80 g/L) | |  |  |  |  |  |  |  |  |  |
|  |  |  |  |  |  |  |  |  |  |  |  |  |  |  |  |  |
|  |  |  |  |  | | Severe anaemia | | RR 0.05; 95% CI 0.00 | |  | |  | |  |  |  |
|  |  |  |  |  |  |  |  | to 0.76) | |  |  |  |  |  |  |  |
|  |  |  |  | Supplementation with iron alone versus no | | LBW (less than 2500 g, birth weight, premature, congenital abnormalities | | No significant difference | |  | | Neonatal death | | No significant difference |  |  |
|  |  |  |  | treatment/placebo | |  |  |  |  |  |  |  |  |  |  |  |
|  |  |  |  |  | | Maternal anaemia at term (Hb less than 110 g/L at 37 weeks’ | | Average RR | |  | |  | |  |  |  |
|  |  |  |  |  |  | gestation or more) | | 0.29; 95% CI 0.19 to 0.47 substantial | |  |  |  |  |  |  |  |
|  |  |  |  |  |  |  | | heterogeneity in study | |  |  |  |  |  |  |  |
|  |  |  |  |  |  |  | | (heterogeneity:T² = 0.44, I² = 80%, Chi² | |  |  |  |  |  |  |  |
|  |  |  |  |  |  |  |  |  |  |  |  |  |  |  |  |  |
|  |  |  |  |  | | Maternal iron deficiency at term (as defined by as defined by | | RR 0.43; 95% CI (0.27 to 0.66) Substantial (heterogeneity: T² = 0.26, I² = | |  | |  | |  |  |  |
|  |  |  |  |  |  | trialists, based on any indicator of iron status at 37 weeks’ | | 85%, Chi² test for heterogeneity P < 0.00001). | |  |  |  |  |  |  |  |
|  |  |  |  |  |  | gestation or more) | |  | |  |  |  |  |  |  |  |
|  |  |  |  |  |  |  |  |  |  |  |  |  |  |  |  |  |
|  |  |  |  |  | | Maternal iron deficiency anaemia at term (Hb below 110 g/L | | (RR 0.33; 95% CI 0.16 to 0.69). The heterogeneity | |  | |  | |  |  |  |
|  |  |  |  |  |  | and at least one additional laboratory indicator at 37 weeks’ | | between the treatment effects was moderate (I² 49%) | |  |  |  |  |  |  |  |
|  |  |  |  |  |  | gestation or more) | |  | |  |  |  |  |  |  |  |
|  |  |  |  |  | | Maternal severe (Hb < 70 g/L) anaemia at any time during | | RR 0.75; 95% CI 0.02 to 29.10 | |  | |  | |  |  |  |
|  |  |  |  |  |  | the second or third trimester | |  |  |  |  |  |  |  |  |  |
|  |  |  |  |  |  |  |  |  |  |  |  |  |  |  |  |  |
|  |  |  |  |  | | Infant ferritin concentration in the first 6 months (in g/L, | | MD was 11.00 μg/L; 95% CI 4.37 to 17.63 μg/L | |  | |  | |  |  |  |
|  |  |  |  |  |  | counting the last reported measure after birth within this | |  |  |  |  |  |  |  |  |  |
|  |  |  |  |  |  | period) | |  |  |  |  |  |  |  |  |  |
|  |  |  |  |  |  |  |  |  |  |  |  |  |  |  |  |  |
|  |  |  |  |  | | Very premature birth (less than 34 weeks’ gestation) | | RR | |  | |  | |  |  |  |
|  |  |  |  |  |  |  |  | 0.32; 95% CI 0.10 to 1.09 | |  |  |  |  |  |  |  |
|  |  |  |  |  | | Maternal Hb concentration at or near term (in g/L, at 34 | | (MD 8.95; 95% CI 6.37 to 11.53 g/L) Substantial heterogeneity (heterogeneity: T² = 21.70, I² = 89%, Chi² test for | |  | |  | |  |  |  |
|  |  |  |  |  |  | weeks’ gestation or more) | | heterogeneity P < 0.00001) | |  |  |  |  |  |  |  |
|  |  |  |  |  |  |  |  |  |  |  |  |  |  |  |  |  |
|  |  |  |  |  | | Maternal Hb concentration within six weeks postpartum (in | | MD 7.26 g/L; 95% CI 4.78 to 9.74 g/L). | |  | |  | |  |  |  |
|  |  |  |  |  |  | g/L) | | Heterogeneity of the results is T² = 3.99, I² = 44%, Chi² test for | |  |  |  |  |  |  |  |
|  |  |  |  |  |  |  | | heterogeneity P < 0.0001 | |  |  |  |  |  |  |  |
|  |  |  |  |  |  |  | | RR 1.81; 95% CI 1.21 to 2.71) | |  |  |  |  |  |  |  |
|  |  |  |  |  |  |  | | Substantial heterogeneity | |  |  |  |  |  |  |  |
|  |  |  |  |  |  |  | | (heterogeneity: T² = 0.20, I² = 75%, Chi² test for heterogeneity P | |  |  |  |  |  |  |  |
|  |  |  |  |  |  |  | | < 0.0005, 95% PI 0.54 to 6.04 | |  |  |  |  |  |  |  |
|  |  |  |  |  | | Maternal high Hb concentrations at or near term (defined as | | RR 3.67; 95%CI 2.23 to 6.04 | |  | |  | |  |  |  |
|  |  |  |  |  |  | Hb greater than 130 g/L, at 34 weeks’ gestation or more) | | (heterogeneity: | |  |  |  |  |  |  |  |
|  |  |  |  |  |  |  | | T² = 0.23, I² = 63%, Chi² test for heterogeneity P < | |  |  |  |  |  |  |  |
|  |  |  |  |  |  |  | | 0.008. | |  |  |  |  |  |  |  |
|  |  |  |  |  | | Transfusion provided | | RR 0.59; 95% CI 0.37 to 0.94 | |  | |  | |  |  |  |
|  |  |  |  |  |  |  |  |  |  |  |  |  |  |  |  |  |
|  |  |  |  |  | | Maternal well being/satisfaction | | No significant difference | |  | |  | |  |  |  |
|  |  |  |  |  |  |  |  |  |  |  |  |  |  |  |  |  |
|  |  |  |  | Supplementation with iron + folic acid versus no | | LBW | | No significant difference | |  | |  | |  |  |  |
|  |  |  |  | treatment/placebo | |  |  |  |  |  |  |  |  |  |  |  |
|  |  |  |  |  | | Infant birth weight | | 57.73 g heavier 95% CI 7.66 to 107.79 g | |  | |  | |  |  |  |
|  |  |  |  |  | | Premature birth (less than 37 weeks’ gestation) | | No significant difference | |  | | Neonatal death | | No significant difference |  |  |
|  |  |  |  |  |  |  |  |  |  |  |  |  |  |  |  |  |
|  |  |  |  |  | | Maternal anaemia at term (Hb less than 110 g/L at 37 weeks’ | | RR 0.34; 95% CI 0.21 to | |  | |  | |  |  |  |
|  |  |  |  |  |  | gestation or more) | | 0.54 | |  |  |  |  |  |  |  |
|  |  |  |  |  |  |  |  |  |  |  |  |  |  |  |  |  |
|  |  |  |  |  | | Maternal iron deficiency at term (as defined by as defined by | | RR 0.24; 95% CI 0.06 to 0.99 | |  | |  | |  |  |  |
|  |  |  |  |  |  | trialists, based on any indicator of iron status at 37 weeks’ | |  |  |  |  |  |  |  |  |  |
|  |  |  |  |  |  | gestation or more) | |  |  |  |  |  |  |  |  |  |
|  |  |  |  |  |  |  |  |  |  |  |  |  |  |  |  |  |
|  |  |  |  |  | | Maternal iron deficiency anaemia at term (Hb less than 110 | | No evidence of significant differences | |  | |  | |  |  |  |
|  |  |  |  |  |  | g/L and at least one additional laboratory indicator at 37 | |  |  |  |  |  |  |  |  |  |
|  |  |  |  |  |  | weeks’ gestation or more) | |  |  |  |  |  |  |  |  |  |
|  |  |  |  |  |  |  |  |  |  |  |  |  |  |  |  |  |
|  |  |  |  |  | | Maternal severe anaemia at any time during second and third | | RR 0.12, 95% CI 0.02 to | |  | |  | |  |  |  |
|  |  |  |  |  |  | trimester (Hb less than 70 g/L) | | 0.63 | |  |  |  |  |  |  |  |
|  |  |  |  |  |  |  |  |  |  |  |  |  |  |  |  |  |
|  |  |  |  |  | | Maternal anaemia at or near term (Hb less than 110 g/L at | | RR 0.34; 95%CI 0.21 to 0.54 | |  | |  | |  |  |  |
|  |  |  |  |  |  | 34 weeks’ gestation or more) | |  |  |  |  |  |  |  |  |  |
|  |  |  |  |  |  |  |  |  |  |  |  |  |  |  |  |  |
|  |  |  |  |  | | Maternal iron deficiency at or near term (as defined by as | | RR 0.24; 95% CI 0.06 to 0.99 | |  | |  | |  |  |  |
|  |  |  |  |  |  | defined by trialists, based on any indicator of iron status at | |  |  |  |  |  |  |  |  |  |
|  |  |  |  |  |  | 34 weeks’ gestation or more) | |  |  |  |  |  |  |  |  |  |
|  |  |  |  |  |  |  |  |  |  |  |  |  |  |  |  |  |
|  |  |  |  |  | | Maternal Hb concentration at or near term (in g/L at 34 | | MD 16.13 g/L; 95% | |  | |  | |  |  |  |
|  |  |  |  |  |  | weeks’ gestation or more) | | CI 12.74 to 19.52 | |  |  |  |  |  |  |  |
|  |  |  |  |  |  |  | | MD 17.10; 95% CI 8.44 to 25.76 | |  |  |  |  |  |  |  |
|  |  |  |  |  | | Maternal Hb concentration within 6 weeks postpartum in | | MD 10.07; 95% | |  | |  | |  |  |  |
|  |  |  |  |  |  | g/L | | CI 7.33 to 12.81 | |  |  |  |  |  |  |  |
|  |  |  |  | Supplementation with iron + folic acid versus folic | | LBW (less than 2500 g) | | RR 0.84; 95% CI 0.73 to 0.95 | |  | | Neonatal death | | No statistical significance |  |  |
|  |  |  |  | acid alone (without iron) supplementation | |  |  |  |  |  |  |  |  |  |  |  |
|  |  |  |  |  | | Infant birth weight | | MD 32.23; 95% CI 0.86 to 63.60 | |  | |  | |  |  |  |
|  |  |  |  |  | | Premature birth (less than 37 weeks’ gestation) | | No statistical significance | |  | |  | |  |  |  |
|  |  |  |  |  | | Congenital abnormality | | No statistical significance | |  | |  | |  |  |  |
|  |  |  |  |  | | Maternal anaemia at term (at 37 weeks’ gestation or more) | | RR | |  | |  | |  |  |  |
|  |  |  |  |  |  |  |  | 0.34; 95% CI 0.21 to 0.55) | |  |  |  |  |  |  |  |
|  |  |  |  |  | | Maternal severe anaemia at any time during second and third | | (RR 0.06; 95% CI 0.01 to | |  | |  | |  |  |  |
|  |  |  |  |  |  | trimester (Hb less than 70 g/L) | | 0.47 | |  |  |  |  |  |  |  |
|  |  |  |  |  | | Maternal anaemia at or near term (at 34 weeks’ gestation or | | RR | |  | |  | |  |  |  |
|  |  |  |  |  |  | more) | | 0.34; 95% CI 0.21 to 0.55) | |  |  |  |  |  |  |  |
|  |  |  |  |  | | Maternal Hb at or near term (in g/L, at 34 weeks’ gestation | | MD 12.44; 95% CI 0.95 to | |  | |  | |  |  |  |
|  |  |  |  |  |  | or more) | | 23.93 | |  |  |  |  |  |  |  |
|  |  |  |  |  | | Maternal high Hb concentrations at or near term (at 37 | | No statistical significance | |  | |  | |  |  |  |
|  |  |  |  |  |  | weeks’ gestation or more) and during pregnancy | |  |  |  |  |  |  |  |  |  |
|  |  |  |  | Supplementation with iron + other vitamins and | | mean maternal Hb | | RR | |  | |  | |  |  |  |
|  |  |  |  | minerals supplementation versus same other | | levels at term | | 10.85,; 95% CI 7.29 to 14.42 | |  |  |  |  |  |  |  |
|  |  |  |  | vitamins and minerals (without iron) | |  | |  | |  |  |  |  |  |  |  |
|  |  |  |  | supplementation | |  | |  | |  |  |  |  |  |  |  |
|  |  |  |  | Daily oral iron + folic acid + other vitamins and | |  | |  | |  | |  | |  |  |  |
|  |  |  |  | minerals supplementation versus daily oral folic acid | |  |  |  |  |  |  |  |  |  |  |  |
|  |  |  |  | + same other vitamins and minerals (without iron) | |  |  |  |  |  |  |  |  |  |  |  |
|  |  |  |  | supplementation (no studies) | |  |  |  |  |  |  |  |  |  |  |  |
|  |  |  |  | Daily oral iron + folic acid + other vitamins and | |  | |  | |  | |  | |  |  |  |
|  |  |  |  | minerals supplementation versus daily oral same | |  |  |  |  |  |  |  |  |  |  |  |
|  |  |  |  | other vitamins and minerals (without iron nor folic | |  |  |  |  |  |  |  |  |  |  |  |
|  |  |  |  | acid) supplementation (no studies) | |  |  |  |  |  |  |  |  |  |  |  |
| Lives Saved Tool supplement detection and | Blencowe, H **34** | 2011 | Physical examination, serological testing and treated with at least |  | |  | |  | | Stillbirths | | RR 0.18;95% | |  |  | Excluding a large SA study from the analysis showed no evidence of heterogeneity (P=0.65 |
| treatment of syphilis in pregnancy to reduce |  |  | 2.4 million units of penicillin G |  |  |  |  |  |  |  |  | CI. 0.10 - 0.33) | |  |  | I2=0%). The summary risk ratio is little changed (RR 0.15: |
| syphilis related stillbirths and neonatal mortality |  |  | To estimate the effect of detection and treatment of active syphilis in pregnancy with at least 2.4MU benzathine penicillin (or equivalent) on syphilis-related stillbirths and neonatal mortality |  |  |  |  |  |  |  |  | There is some evidence of heterogeneity across these 8 | |  |  | 95% c.i. 0.10 – 0.21 ). |
|  |  |  |  |  |  |  |  |  |  |  |  | studies (P=0.02; I2=56.8%). | |  |  |  |
|  |  |  |  |  |  |  |  |  |  |  |  |  |  |  |  |  |
|  |  |  | Treatment |  | |  | |  | | Stillbirth | | RR 1.3; 95% CI 0.81 – 2,13): RR 1.4; 95% | |  |  |  |
|  |  |  |  |  |  |  |  |  |  |  |  | CI 0.34 – 5.75: | |  |  |  |
|  |  |  |  |  |  |  |  |  |  |  |  | RR 1.4; 95% CI 1.05 – 1.86 | |  |  |  |
|  |  |  |  |  |  |  |  |  |  |  |  | RR | |  |  |  |
|  |  |  |  |  |  |  |  |  |  |  |  | 0.94; 95% CI 0.90 – 0.98: | |  |  |  |
|  |  |  |  |  |  |  |  |  |  |  |  | RR 0.68; 95% CI 0.2 – 2.4 | |  |  |  |
|  |  |  | Syphilis detection |  | |  | |  | | Perinatal mortality | | RR=0.45: 95% CI 0.17 – 1.12 | |  |  |  |
|  |  |  | and penicillin treatment |  |  |  |  |  |  |  |  |  |  |  |  |  |
|  |  |  | Treatment with penicillin |  | |  | |  | | Perinatal | | Reduction | |  |  |  |
|  |  |  |  |  |  |  |  |  |  | mortality | | of 35% with each dose of penicillin received | |  |  |  |
|  |  |  |  |  |  |  |  |  |  |  | | (adjusted RR = 0.65: 95% CI (0.5 – 0.85) | |  |  |  |
|  |  |  |  |  | |  | |  | | Perinatal mortality | | RR 0.34 (0.11 – 1.03) | |  |  |  |
|  |  |  |  |  |  |  |  |  |  |  |  | 7/24 perinatal deaths in | |  |  |  |
|  |  |  |  |  |  |  |  |  |  |  |  | untreated pregnant women, 0/8 in treated women and 4/ | |  |  |  |
|  |  |  |  |  |  |  |  |  |  |  |  | 145 in those negative for syphilis | |  |  |  |
|  |  |  | Treatment with penicillin | Preterm | | RR 0.36; 95% 0.27 – 0.47 | |  | | Neonatal death | | RR 0.20; 95% CI 0.13 – 0.32. | |  |  |  |
|  |  |  |  |  |  | There was no evidence of heterogeneity | |  |  |  |  | There was not strong evidence of heterogeneity | |  |  |  |
|  |  |  |  |  |  | p=0.49, I2 =0.0% | |  |  |  |  | (I-squared=40.5%, p=0.15). | |  |  |  |
|  |  |  |  | Congenital Syphilis | | A random effects meta-analysis produced an | |  | |  | |  | |  |  | These three studies showed some evidence of heterogeneity |
|  |  |  |  |  |  | estimated risk ratio for congenital syphilis of 0.03 (95%c. | |  |  |  |  |  |  |  |  | (p=0.08 I2 =61.3%) |
|  |  |  |  |  |  | i. 0.02 – 0.07) | |  |  |  |  |  |  |  |  |  |
| Calcium supplementation during pregnancy for preventing hypertensive disorders and related problems (Review) | Hofmeyr, G 35 | 2006 | To assess the effects of calcium supplementation during pregnancy on hypertensive disorders of pregnancy and related maternal and child adverse outcomes | High BP with or without proteinuria | | High BP | | RR 0.58; 95% CI0.43-0.79 | | Maternal death | | Too few events | |  |  |  |
|  |  |  |  |  |  |  |  |  |  |  |  |  |  |  |  |  |
|  |  |  |  |  |  |  |  | (smaller effect in larger trials) | |  |  |  |  |  |  |  |
|  |  |  |  |  | | High BP in women at high risk of developing HPT | | RR 0.47; 95% CI0.22-0.97 | | Stillbirth or death before discharge from hospital | | No effect | |  |  |  |
|  |  |  |  |  | | High BP in women with low baseline dietary calcium | | RR 0.38; 95% CI 0.22-0.64 | |  | |  | |  |  |  |
|  |  |  |  | Pre-eclampsia | | Pre-eclampsia | | RR 0.35; 95% CI 0.20-0.60 | |  | |  | |  |  |  |
|  |  |  |  |  |  |  |  |  |  |  |  |  |  |  |  |  |
|  |  |  |  |  |  |  |  | Reduction of risk* | |  |  |  |  |  |  |  |
|  |  |  |  |  | | Pre-eclampsia in women with low risk | | RR 0.49; 95% CI (0.28-0.87) | |  | |  | |  |  |  |
|  |  |  |  |  | | Pre-eclampsia in women with adequate calcium intake | | RR 0.62; 95% CI 0.32-1.20 | |  | |  | |  |  |  |
|  |  |  |  |  | | Pre-eclampsia in women with high risk | | RR 0.22; 95% CI 0.12-0.42 | |  | |  | |  |  |  |
|  |  |  |  |  | | Pre-eclampsia in women with low baseline calcium intake | | RR 0.29; 95% CI0.16-0.54 | |  | |  | |  |  |  |
|  |  |  |  |  | | Placental abruption | | Too few events | |  | |  | |  |  |  |
|  |  |  |  |  | | C-section | | No statistically significant effect in risk | |  | |  | |  |  |  |
|  |  |  |  | Preterm delivery | | Overall | | No effect | |  | |  | |  |  |  |
|  |  |  |  |  | | Women at high risk of developing hypertension | | RR 0.45; 95% CI 0.24-0.83 | |  | |  | |  |  |  |
|  |  |  |  | Birth weight less than 2500g | | Women at risk of hypertension | | RR 0.45; 95% CI 0.22-0.95 | |  | |  | |  |  |  |
|  |  |  |  | Admission to neonatal ICU | | No effect shown | |  | |  | |  | |  |  |  |
| Role of calcium supplementation during pregnancy in reducing risk of developing gestational hypertensive disorders: a meta-analysis of studies from developing countries | Imdad, A **36** | 2011 | To evaluate preventive effect of calcium supplementation during pregnancy on gestational hypertensive disorders and related maternal and neonatal mortality in developing countries. | Severe morbidities (admission to intensive care unit, eclampsia, severe pre-eclampsia, placental abruption, HELLP syndrome, renal failure, maternal death) | | Overall | | RR 0.80; 95% CI 0.70-0.91 | | Maternal mortality | | All-cause (reported in only 1 study) | | RR 0.17; 95% CI 0.03-0.76 |  |  |
|  |  |  |  |  |  |  |  |  |  |  |  |  |  |  |  |  |
|  |  |  |  |  |  |  |  | Moderate quality | |  |  |  |  | Low quality grade for this study; due to low number of events |  |  |
|  |  |  |  | Eclampsia | | Overall | | RR 0.68; 95% CI 0.48-0.97 | | Neonatal mortality | | Overall | | RR 0.70; 95% CI 0.56-0.88 |  |  |
|  |  |  |  |  |  |  |  | Low quality grade | |  |  |  |  |  |  |  |
|  |  |  |  | Severe pre-eclampsia | | Overall | | RR 0.70; 95% CI 0.46-1.05 | |  | |  | |  |  |  |
|  |  |  |  | Pre-eclampsia | | Overall | | RR 0.41; 95% CI 0.24-0.69 | |  | |  | |  |  |  |
|  |  |  |  |  | | In women with higher pre-pregnancy risk of developing gestational hypertension compared to lower risk | | RR 0.18; 95% CI 0.07-0.42 | |  | |  | |  |  |  |
|  |  |  |  |  |  |  |  |  |  |  |  |  |  |  |  |  |
|  |  |  |  |  |  |  |  | High quality | |  |  |  |  |  |  |  |
|  |  |  |  | Gestational hypertension (+-proteinuric) | | Overall | | RR 0.55; 95% CI 0.36-0.85 | |  | |  | |  |  |  |
|  |  |  |  |  |  |  |  |  |  |  |  |  |  |  |  |  |
|  |  |  |  |  |  |  |  | High quality | |  |  |  |  |  |  |  |
|  |  |  |  |  | | Women at higher risk of development of hypertension during pregnancy | | RR 0.32; 95% CI 0.06-1.63 | |  | |  | |  |  |  |
|  |  |  |  |  |  |  |  |  |  |  |  |  |  |  |  |  |
|  |  |  |  |  |  |  |  | (not statistically significant) | |  |  |  |  |  |  |  |
|  |  |  |  | Preterm births | | Overall | | RR 0.88; 95% CI 0.78-0.99 | |  | |  | |  |  |  |
|  |  |  |  | LBW in new born | | Overall | | RR 0.81; 95% CI 0.58-1.12 | |  | |  | |  |  |  |
|  |  |  |  | SGA | | Overall | | RR 0.90; 95% CI 0.59-1.38 | |  | |  | |  |  |  |
| Antiplatelets for prevention of pre-eclampsia and its complications | Duley, L **37** | 2007 | To assess the effectiveness and safety of antiplatelet agents for women at risk of developing pre-eclampsia. | Antiplatelet agents versus placebo or no treatment for the primary prevention of pre-eclampsia and its complications | |  | |  | | Any reported deaths (stillbirths, neonatal deaths, infant deaths) | |  | | RR 0.86; 95% CI 0.76-0.98 |  |  |
|  |  |  |  |  |  |  |  |  |  |  |  |  |  |  |  |  |
|  |  |  |  | Pregnancy-induced hypertension | | Gestational hypertension | | RR 0.95; 95% CI 0.88-1.03 | |  | | High risk | | RR 0.69; 95% CI 0.53-0.90 |  |  |
|  |  |  |  |  |  |  |  |  |  |  |  |  |  |  |  |  |
|  |  |  |  |  |  |  |  | (no overall difference) | |  |  |  |  |  |  |  |
|  |  |  |  |  | | High-risk women | | RR 0.54; 95% CI 0.41-0.70 | |  | |  | |  |  |  |
|  |  |  |  | Proteinuric pre-eclampsia | | Pre-eclampsia | | RR 0.83; 95% CI 0.77-0.89 | |  | |  | |  |  |  |
|  |  |  |  |  | | High-risk women | | RR 0.75; 95% CI 0.66-0.95 | |  | |  | |  |  |  |
|  |  |  |  |  | | Moderate risk | | RR 0.86; 95% CI 0.79-0.95 | |  | |  | |  |  |  |
|  |  |  |  |  | | Higher doses (>75mg/day) | | RR 0.64; 95% CI 0.51-0.80 | |  | |  | |  |  |  |
|  |  |  |  | Placental abruption | |  | | RR 1.10; 95% CI 0.89-1.37 | |  | |  | |  |  |  |
|  |  |  |  |  |  |  |  |  |  |  |  |  |  |  |  |  |
|  |  |  |  |  |  |  |  | (No significant difference in risk of placental abruption) | |  |  |  |  |  |  |  |
|  |  |  |  | Preterm birth (<37 weeks) | |  | | RR 0.92; 95% CI 0.88-0.97 | |  | |  | |  |  |  |
|  |  |  |  | SGA | |  | | RR 0.90; 95% CI 0.83-0.98 | |  | |  | |  |  |  |
|  |  |  |  | Antiplatelet agents versus placebo or no treatment for the secondary prevention of pre-eclampsia and its complications in women with gestational hypertension | |  | |  | | Antiplatelet agents versus placebo or no treatment for the secondary prevention of pre-eclampsia and its complications in women with gestational hypertension | |  | |  |  |  |
|  |  |  |  | Proteinuric pre-eclampsia | |  | | RR 0.60; 95% CI 0.45-0.78 | | Any reported deaths (stillbirths, neonatal deaths, infant deaths) | |  | | RR 1.02; 95% CI 0.72-1.45 |  |  |
|  |  |  |  |  |  |  |  |  |  |  |  |  |  |  |  |  |
|  |  |  |  |  |  |  |  |  |  |  |  |  |  | (No statistical difference) |  |  |
|  |  |  |  | Preterm birth (<37 weeks) | |  | | RR 0.87; 95% CI 0.75-0.99 | |  | |  | |  |  |  |
|  |  |  |  | SGA | |  | | RR 0.76; 95% CI 0.52-1.10 | |  | |  | |  |  |  |
|  |  |  |  | LBW (<2500g) | |  | | RR 0.24; 95% CI 0.09-0.65 | |  | |  | |  |  |  |
|  |  |  |  |  |  |  |  |  |  |  |  |  |  |  |  |  |
|  |  |  |  |  |  |  |  | One trial | |  |  |  |  |  |  |  |
|  |  |  |  | C-section | |  | | RR 0.87; 95% CI 0.31-2.47 | |  | |  | |  |  |  |
|  |  |  |  |  |  |  |  |  |  |  |  |  |  |  |  |  |
|  |  |  |  |  |  |  |  | No difference; small trial | |  |  |  |  |  |  |  |
| Psychosocial and psychological interventions for treating | Dennis, C **39** | 2007 | To assess the effects, on mothers and their families, of psychosocial and psychological interventions | Maternal depressive symptomatology immediately post-reduction | | Inter-personal psychotherapy compared to a | | RR 0.46 | |  | |  | |  |  |  |
| antenatal depression (Review) |  |  | compared with usual antepartum care in the treatment of antenatal depression. | (variously defined) | | parenting education programme (using Clinic Global Impression Scale) | | (0.26-0.83) | |  |  |  |  |  |  |  |
|  |  |  |  |  |  |  |  |  |  |  |  |  |  |  |  |  |
|  |  |  |  |  | |  | | 1 trial | |  |  |  |  |  |  |  |
|  |  |  |  |  | |  | | decrease in risk of depression sympomatology | |  |  |  |  |  |  |  |
|  |  |  |  |  |  |  |  |  |  |  |  |  |  |  |  |  |
|  |  |  |  |  | |  | | n=38 | |  |  |  |  |  |  |  |
|  |  |  |  |  |  |  |  |  |  |  |  |  |  |  |  |  |
|  |  |  |  |  | | On the Hamilton Rating Scale for Depression | | RR 0.82 (0.65-1.03) | |  | |  | |  |  |  |
| Antiretrovirals for reducing the risk of mother-to-child | Siegfried, N **40.** | 2011 | To determine whether, and to what extent, antiretroviral regimens aimed at decreasing the risk of mother-to-child transmission of HIV infection achieve a clinically useful decrease in transmission risk, and what effect these interventions have on maternal and infant mortality and morbidity. | Antiretrovirals versus placebos (three trials): Breastfeeding | | DITRAME | | HIV infection : | | Infant death: | |  | |  |  |  |
| transmission of HIV infection |  |  |  |  |  |  |  | 4-8 weeks (Efficacy 32.00%; | | First week after | |  |  |  |  |  |
|  |  |  |  |  |  |  |  | 95% CI 1.50 to 62.50) | | birth (RR 2.03; 95% CI 0.51 to 8.00), | |  |  |  |  |  |
|  |  |  |  |  |  |  |  |  |  |  |  |  |  |  |  |  |
|  |  |  |  |  |  |  |  | 3 to 4 months (Efficacy 33.07%; 95% | | First 4 to 8 weeks | |  |  |  |  |  |
|  |  |  |  |  |  |  |  | CI 5.57 to 60.57) | | (RR 1.77; 95% CI 0.53 to 5.97) | |  |  |  |  |  |
|  |  |  |  |  |  |  |  |  |  |  |  |  |  |  |  |  |
|  |  |  |  |  |  |  |  | 6 months (Efficacy 34.55%; 95% CI 9.05 to | | First 3 to 4 months (RR | |  |  |  |  |  |
|  |  |  |  |  |  |  |  | 60.05) | | 0.74; 95% CI 0.35 to 1.58) | |  |  |  |  |  |
|  |  |  |  |  |  |  |  |  |  |  |  |  |  |  |  |  |
|  |  |  |  |  |  |  |  | 12 months (Efficacy 34.31%; 95% CI 9.30 to 59.32) | | First 6 months (RR 0.62; 95% CI 0.35 to 1.09) | |  |  |  |  |  |
|  |  |  |  |  |  |  |  |  |  |  |  |  |  |  |  |  |
|  |  |  |  |  |  |  |  | 18 months (Efficacy 29.74%; 95% CI 2.73 to 56.75) | | First 12 months (RR 0.75; 95% CI 0.48 | |  |  |  |  |  |
|  |  |  |  |  |  |  |  |  | | to 1.17) | |  |  |  |  |  |
|  |  |  |  |  |  |  |  |  |  |  |  |  |  |  |  |  |
|  |  |  |  |  |  |  |  |  | | The first 18 months (RR 0.80; 95% CI 0.53 to | |  |  |  |  |  |
|  |  |  |  |  |  |  |  |  | | 1.21). | |  |  |  |  |  |
|  |  |  |  |  | |  | | Premature delivery: | | Stillbirth (RR 0.14; 95% CI 0.02 to 1.17) | |  | |  |  |  |
|  |  |  |  |  |  |  |  | RR 0.14; 95% CI 0.03 to 0.58 | |  |  |  |  |  |  |  |
|  |  |  |  |  | |  | | LBW: | | Maternal death by 6 weeks: No significant difference | |  | |  |  |  |
|  |  |  |  |  |  |  |  | RR 0.92; 95% CI 0.57 | |  |  |  |  |  |  |  |
|  |  |  |  |  |  |  |  | to 1.47 | |  |  |  |  |  |  |  |
|  |  |  |  |  | |  | | Mother: | |  | |  | |  |  |  |
|  |  |  |  |  |  |  |  | Severe anaemia at day 8 or 45 days postpartum: No significant difference | |  |  |  |  |  |  |  |
|  |  |  |  |  |  |  |  |  |  |  |  |  |  |  |  |  |
|  |  |  |  |  |  |  |  | Severe neutropenia: No significant difference | |  |  |  |  |  |  |  |
|  |  |  |  |  | |  | | Infant: | |  | |  | |  |  |  |
|  |  |  |  |  |  |  |  | Severe anaemia: No significant difference | |  |  |  |  |  |  |  |
|  |  |  |  |  |  |  |  |  |  |  |  |  |  |  |  |  |
|  |  |  |  |  |  |  |  | Severe neutropenia: No significant difference | |  |  |  |  |  |  |  |
|  |  |  |  |  | | RETRO-CI | | HIV infection: | | Infant death: | |  | |  |  |  |
|  |  |  |  |  |  |  |  | 4 to 8 weeks (Efficacy 43.78%; 95% CI 8.78 to 78.78) | | First week after birth: | |  |  |  |  |  |
|  |  |  |  |  |  |  |  |  | | RR 0.13; 95% CI 0.02 to 0.99 | |  |  |  |  |  |
|  |  |  |  |  |  |  |  | 3 to 4 months (Efficacy 36.95%; 95%CI 2.94 to 70.96) | |  | |  |  |  |  |  |
|  |  |  |  |  |  |  |  |  | | First 3 to 4 months after birth: RR 0.15; 95% CI 0.05 to 0.49 | |  |  |  |  |  |
|  |  |  |  |  | |  | | Mother: | | Stillbirth: no difference | |  | |  |  |  |
|  |  |  |  |  |  |  |  | Severe maternal clinical disorder: no significant difference | |  |  |  |  |  |  |  |
|  |  |  |  |  |  |  |  |  |  |  |  |  |  |  |  |  |
|  |  |  |  |  |  |  |  | Severe laboratory abnormality: no significant difference | |  |  |  |  |  |  |  |
|  |  |  |  |  | |  | | Infant: | | Postpartum death: no significant difference | |  | |  |  |  |
|  |  |  |  |  |  |  |  | Congenital abnormality: no significant difference | |  |  |  |  |  |  |  |
|  |  |  |  |  |  |  |  |  |  |  |  |  |  |  |  |  |
|  |  |  |  |  |  |  |  | Severe laboratory abnormality: no significant difference | |  |  |  |  |  |  |  |
|  |  |  |  |  | | PETRA | | HIV infection: | | Infant death: | |  | |  |  |  |
|  |  |  |  |  |  | PETRA-a | | 4 to 8 weeks (Efficacy 62.75%; 95% CI 40.76 to | | 4-8 weeks: no significant difference | |  |  |  |  |  |
|  |  |  |  |  |  |  | | 84.74) | |  | |  |  |  |  |  |
|  |  |  |  |  |  |  | |  | | First 18 months: no significant difference | |  |  |  |  |  |
|  |  |  |  |  |  |  | | Combined endpoint of HIV infection or death at 4-8 weeks: | |  | |  |  |  |  |  |
|  |  |  |  |  |  |  |  |  |  |  |  |  |  |  |  |  |
|  |  |  |  |  | |  | |  | | Stillbirths: no significant difference | |  | |  |  |  |
|  |  |  |  |  | | PETRA-b | | HIV infection: | | Infant death: | |  | |  |  |  |
|  |  |  |  |  |  |  |  | 4 to 8 weeks (Efficacy 41.83%; 95% CI 12.82 to 70.84) | | 4-8 weeks: no significant difference | |  |  |  |  |  |
|  |  |  |  |  |  |  |  |  |  |  |  |  |  |  |  |  |
|  |  |  |  |  |  |  |  | First 18 months: no significant difference | | First 18 months: no significant difference | |  |  |  |  |  |
|  |  |  |  |  |  |  |  |  |  |  |  |  |  |  |  |  |
|  |  |  |  |  |  |  |  |  |  |  |  |  |  |  |  |  |
|  |  |  |  |  | |  | | Risk of HIV infection or death: | | Stillbirths: no significant difference | |  | |  |  |  |
|  |  |  |  |  |  |  |  | 4 to 8 weeks | |  |  |  |  |  |  |  |
|  |  |  |  |  |  |  |  | (Efficacy 41.83%; 95% CI 12.82 to 70.84) | |  |  |  |  |  |  |  |
|  |  |  |  |  |  |  |  |  |  |  |  |  |  |  |  |  |
|  |  |  |  |  |  |  |  | First 18 months: no significant difference | |  |  |  |  |  |  |  |
|  |  |  |  |  | | PETRA-C | | HIV infection: | | Infant death: | |  | |  |  |  |
|  |  |  |  |  |  |  |  | 4 to 8 weeks: no significant difference | | 4-8 weeks: no significant difference | |  |  |  |  |  |
|  |  |  |  |  |  |  |  |  |  |  |  |  |  |  |  |  |
|  |  |  |  |  |  |  |  | First 18 months: no significant difference | | First 18 months: no significant difference | |  |  |  |  |  |
|  |  |  |  |  |  |  |  |  |  |  |  |  |  |  |  |  |
|  |  |  |  |  | |  | | Combined outcome | | Stillbirths: no significant difference | |  | |  |  |  |
|  |  |  |  |  |  |  |  | of HIV infection or death: | |  |  |  |  |  |  |  |
|  |  |  |  |  |  |  |  | 4 to 8 weeks: No significant difference | |  |  |  |  |  |  |  |
|  |  |  |  |  |  |  |  |  |  |  |  |  |  |  |  |  |
|  |  |  |  |  |  |  |  | First 18 months: No significant difference | |  |  |  |  |  |  |  |
|  |  |  |  |  | |  | | Mother: | |  | |  | |  |  |  |
|  |  |  |  |  |  |  |  | Frequency of grade 3 and 4 laboratory events before week 6: No significant difference | |  |  |  |  |  |  |  |
|  |  |  |  |  | |  | | Infant: | |  | |  | |  |  |  |
|  |  |  |  |  |  |  |  | Frequency of grade 3 and 4 laboratory events: No significant difference | |  |  |  |  |  |  |  |
|  |  |  |  |  |  |  |  |  |  |  |  |  |  |  |  |  |
|  |  |  |  |  |  |  |  | Frequency of neurological events up to 18 months: No significant difference | |  |  |  |  |  |  |  |
|  |  |  |  |  |  |  |  |  |  |  |  |  |  |  |  |  |
|  |  |  |  |  |  |  |  | Frequency of congenital abnormalities: No significant difference | |  |  |  |  |  |  |  |
|  |  |  |  | Antiretrovirals versus placebos (three trials): Non Breastfeeding | | PACTG 076 | | HIV infection: | | Infant death in the first 18 months: No significant difference | |  | |  |  |  |
|  |  |  |  |  |  |  |  | At 18 months (Efficacy 66.22%; 95%CI 33.94 to 98.50) | |  |  |  |  |  |  |  |
|  |  |  |  |  | |  | | Premature delivery: No significant difference | | Stillbirth rates: No significant difference | |  | |  |  |  |
|  |  |  |  |  | |  | | Low birth weight: No significant difference | |  | |  | |  |  |  |
|  |  |  |  |  | |  | | Mother: | |  | |  | |  |  |  |
|  |  |  |  |  |  |  |  | Severe haematological toxicity: No significant difference | |  |  |  |  |  |  |  |
|  |  |  |  |  |  |  |  |  |  |  |  |  |  |  |  |  |
|  |  |  |  |  |  |  |  | Severe chemistry toxicity: No significant difference | |  |  |  |  |  |  |  |
|  |  |  |  |  | |  | | Infant: Frequency of anaemia in first 6 weeks: (44 with | |  | |  | |  |  |  |
|  |  |  |  |  |  |  |  | ZDV vs 24 with placebo; p=0.001) | |  |  |  |  |  |  |  |
|  |  |  |  |  | | Limpongsanurak: | | HIV transmission at 6 months: No difference | | Stillbirths: No significant difference | |  | |  |  |  |
|  |  |  |  |  |  |  |  | (Efficacy 8.59%; 95% CI -26.63 to 43.81) | |  |  |  |  |  |  |  |
|  |  |  |  |  | | Thai-CDC | | HIV infection: | | Stillbirths: no significant effect | |  | |  |  |  |
|  |  |  |  |  |  |  |  | At birth: no significant effect | |  |  |  |  |  |  |  |
|  |  |  |  |  |  |  |  | 4-8 weeks: Efficacy 50.26%; 95% CI 13.80 to 86.72 | |  |  |  |  |  |  |  |
|  |  |  |  |  |  |  |  |  |  |  |  |  |  |  |  |  |
|  |  |  |  |  | |  | | Low birth weight: no significant effect | | Infant deaths in the first 4-8 weeks: no significant effect | |  | |  |  |  |
|  |  |  |  |  | |  | | Mother: Severe postpartum | |  | |  | |  |  |  |
|  |  |  |  |  |  |  |  | anaemia: no significant difference | |  |  |  |  |  |  |  |
|  |  |  |  |  | |  | | Infant: | |  | |  | |  |  |  |
|  |  |  |  |  |  |  |  | Grade 3 haematological effects: no significant difference | |  |  |  |  |  |  |  |
|  |  |  |  |  |  |  |  | Growth, haematological, immunological or clinical events: no significant difference | |  |  |  |  |  |  |  |
|  |  |  |  | Longer versus shorter regimens using the same antiretrovirals (Breastfeeding) | | Thistle 2004 | | HIV infection rates at birth | | No significant difference | | Risk of infant deaths | | Not statistically different during the following time periods after delivery: |  |  |
|  |  |  |  |  |  |  |  |  |  | (Efficacy -15.10%, 95% CI -152.08 to 121.88), 4-8 weeks (Efficacy 16.72% 95% CI -42.43 to 75.87), 3 to 4 months (Efficacy 13.95%, 95% CI -38.50 to 66.40), 6 months (Efficacy 8.28%, | |  |  | 4 to 8 weeks (RR 1.00; 95% CI 0.21 to 4.85), |  |  |
|  |  |  |  |  |  |  |  |  |  | 95% CI -41.76 to 58.32) and 12 months (Efficacy 8.62%, 95% CI -33.93 to 51.17) | |  |  | 3 to 4 months (RR 1.75; 95% CI 0.53 to 5.81), 6 months (RR 2.00; |  |  |
|  |  |  |  |  |  |  |  |  |  |  | |  |  | 95% CI 0.71 to 5.66) |  |  |
|  |  |  |  |  |  |  |  |  |  |  | |  |  | 12 months (RR 2.00; 95% CI 0.71 to |  |  |
|  |  |  |  |  |  |  |  |  |  |  | |  |  | 5.66). |  |  |
|  |  |  |  |  | |  | | Prematurity | | Not significantly different (RR | | Still births | | None |  |  |
|  |  |  |  |  |  |  |  |  |  | 1.75, 95% CI 0.53 to 5.81) | |  |  |  |  |  |
|  |  |  |  | Longer versus shorter regimens using the same antiretrovirals (non-Breastfeeding) | | PHPT-1 a | | HIV infection rates in infants | | No significant difference in HIV infection rates in infants | | Infant deaths | | There was also no significant | (Efficacy 14.61%; 95% CI -30.80 to 60.02). |  |
|  |  |  |  |  |  |  |  |  |  | at 6 months (Efficacy 24.42%; 95% CI -20.25 to 69.09) | |  |  | difference in infant deaths during the first 6 months (RR 0.82; |  |  |
|  |  |  |  |  |  |  |  |  |  |  | |  |  | 95% CI 0.24 to 2.82), |  |  |
|  |  |  |  |  | |  | | LBW (higher) | | (RR 1.65; 95% CI 1.04 to 2.60) | | Stillbirth rate | | (RR 0.55; 95% CI 0.23 to |  |  |
|  |  |  |  |  |  |  |  |  |  |  |  |  |  | 1.33) and the risk of premature delivery (RR 2.01; 95% CI 0.94 |  |  |
|  |  |  |  |  |  |  |  |  |  |  |  |  |  | to 4.31). |  |  |
|  |  |  |  |  | | PHPT-1 b | | Premature birth (RR 1.86; 95%CI 0.84 to 4.12) | | The long-short regimen significantly reduced HIV infection rate at 6 months (Efficacy 45.33%; 95% CI 1.39 to 89.31). | | Risk of infant mortality | | There was no significant difference in the risk of infant | reduced this |  |
|  |  |  |  |  |  |  |  |  |  |  |  |  |  | mortality during the first 6 months (RR 1.38; 95% CI 0.44 to | was not statistically significant (Efficacy 37.08%; 95% CI -6.88 |  |
|  |  |  |  |  |  |  |  |  |  |  |  |  |  | 4.31), | to 81.04). |  |
|  |  |  |  |  | |  | | LBW | | RR 1.50; 95% CI 0.92 to 2.43 | | Stillbirth | | (RR 0.33; |  |  |
|  |  |  |  |  |  |  |  |  |  |  |  |  |  | 95% CI 0.11 to 1.01). |  |  |
|  |  |  |  |  |  |  |  |  |  |  |  |  |  |  |  |  |
|  |  |  |  |  | | PHPT-2 | | HIV infection rates | | Birth: Efficacy 37.50%; 95% CI -40.94 to 115.94) | | Infant death rates | | The first 6 months was significantly | Risk of HIV infection or death at 6 months |  |
|  |  |  |  |  |  |  |  |  |  | Six months: Efficacy 28.57%; 95% CI | |  |  | reduced in the NVP-NVP arm (RR 0.20; 95% CI 0.04 to 0.91) | was not statistically significant (Efficacy 45.00%; 95% CI -4.00 |  |
|  |  |  |  |  |  |  |  |  |  | -26.09 to 83.23 | |  |  |  | to 94.00) |  |
|  |  |  |  |  |  |  |  |  |  | Not significantly different in the two groups | |  |  |  |  |  |
|  |  |  |  |  | |  | |  | |  | | Stillbirth rates | | (RR 0.25; 95% CI 0.05 to 1.17) |  |  |
|  |  |  |  |  |  |  |  |  |  |  |  |  |  | and low birth weight (RR 0.85; 95% CI 0.60 to 1.19) were not |  |  |
|  |  |  |  |  |  |  |  |  |  |  |  |  |  | statistically significant. |  |  |
|  |  |  |  |  | | Bhoopat 2005 | | HIV infection rates | | Not significantly different at birth | |  | |  |  |  |
|  |  |  |  |  |  |  |  |  |  | (no infected babies with the ’long course’ regimen vs. 1 infected | |  |  |  |  |  |
|  |  |  |  |  |  |  |  |  |  | baby with the ’short course regimen; Efficacy 100.00%; 95% CI - | |  |  |  |  |  |
|  |  |  |  |  |  |  |  |  |  | 294.36 to 494.36) and 3 to 4 months (no infected babies with the | |  |  |  |  |  |
|  |  |  |  |  |  |  |  |  |  | ’long course’ regimen vs 4 infected babies with the ’short course’ | |  |  |  |  |  |
|  |  |  |  |  |  |  |  |  |  | regimen’; Efficacy 100.00%; 95% CI -16.50 to 216.50). | |  |  |  |  |  |
|  |  |  |  |  |  |  |  |  |  |  |  |  |  |  |  |  |
|  |  |  |  | Regimens using different drugs and durations | | HIVNET 012 | | HIV infection rates | | 4-8 weeks (Efficacy 41.00%; 95% | | Infant mortality | | No specific differences in the first week after birth (RR 2.50; 95% CI 0.49 to | 4-8 weeks (Efficacy 41.74%; 95% CI 14.30 |  |
|  |  |  |  | of treatment | |  |  |  |  | CI 11.84 to 70.16); | |  |  | 12.79), | to 69.18), 3 to 4 months (Efficacy 40.00%; 95% CI 14.34 to |  |
|  |  |  |  | (breastfeeding) | |  |  |  |  | 3-4 months (Efficacy 38.91%; 95%CI 11.24 to 66.58); | |  |  | first 4 to 8 weeks (RR 2.50; 95% CI 0.79 to 7.89) | 65.66), 12 months (Efficacy 32.17%; 95% CI 8.51 to 55.83) |  |
|  |  |  |  |  | |  |  |  |  | 12 months (Efficacy 35.98%; 95% CI 9.25 to 62.71); | |  |  | first 18months (RR 1.24; 95%CI 0.81 to 1.89). | and 18 months (Efficacy 32.57%; 95% CI 9.93 to 55.21). |  |
|  |  |  |  |  | |  |  |  |  | 18 months (Efficacy 39.15%; 95% CI 13.81 to 64.49). | |  |  |  |  |  |
|  |  |  |  |  |  |  |  |  |  |  |  |  |  |  |  |  |
|  |  |  |  |  | |  | |  | |  | | Risk of stillbirths | | (RR 2.00; |  |  |
|  |  |  |  |  |  |  |  |  |  |  |  |  |  | 95% CI 0.18 to 21.94) or low birth weight (RR 0.67; 95% CI |  |  |
|  |  |  |  |  |  |  |  |  |  |  |  |  |  | 0.35 to 1.29). |  |  |
|  |  |  |  |  |  |  |  |  |  |  |  |  |  |  |  |  |
|  |  |  |  |  | | Taha 2003 | | HIV infection | | 4 to 8 weeks (Efficacy 36.79%; 95% CI 3.57 to 70.01). | | Risk of death | | the first 4 to 8 weeks (RR 1.26; 95% CI 0.60 to 2.67). |  |  |
|  |  |  |  |  |  |  |  |  |  |  |  |  |  |  |  |  |
|  |  |  |  |  | | Taha 2004 | | HIV infection | | 4 to 8 weeks (Efficacy -15.60%; 95% CI -50.21 to 19.01) or in the risk of infant death in the first 4 to 8 weeks (RR 1.74; 95% CI 0.51 to 5.91). | |  | |  |  |  |
|  |  |  |  |  |  |  |  |  |  |  |  |  |  |  |  |  |
|  |  |  |  |  | | Thistle 2007 | | HIV infection | | There was no difference | | Infant deaths | | four to six weeks was similar in both | four to six weeks, no statistically significant difference |  |
|  |  |  |  |  |  |  |  |  |  | at birth between the groups (Efficacy -5.45%; | |  |  | groups (RR = 1.08; 95% CI 0.61 to 1.92). | between the groups (Efficacy 3.97%,95%CI -26.14 to 34.08). |  |
|  |  |  |  |  |  |  |  |  |  | 95%CI -79.95 to 69.05) | |  |  | Adverse events |  |  |
|  |  |  |  |  |  |  |  |  |  | four to six weeks, HIV infection | |  |  |  |  |  |
|  |  |  |  |  |  |  |  |  |  | efficacy remained statistically non-significant with an efficacy of 9.21% (95%CI -27.56 to 45.8) | |  |  |  |  |  |
|  |  |  |  |  | | Chi 2007 | | HIV infection | | Not statistically different between | | risk of death | | four to eight weeks after birth was not |  |  |
|  |  |  |  |  |  |  |  |  |  | groups (Efficacy 20.40%, 95%CI -66.86 to 107.66) or at four | |  |  | statistically different between the groups (RR 1.26; 95%CI 0.34 |  |  |
|  |  |  |  |  |  |  |  |  |  | to eight weeks (Efficacy 28.93%, 95%CI -40.84 to 98.70). | |  |  | to 4.61), |  |  |
|  |  |  |  |  | |  | |  | |  | | Risk of stillbirth | | (RR 1.99, 95%CI 0.18 to |  |  |
|  |  |  |  |  |  |  |  |  |  |  |  |  |  | 21.77). |  |  |
|  |  |  |  | Regimens using different drugs and durations | | PACTG 316 | | HIV infection | | Birth (Efficacy 2.22%, 95% CI -140.39 to 144.83) | | Deaths | | 4 to 8 weeks (RR 0.60, 95%CI 0.14 to 2.50). |  |  |
|  |  |  |  | of treatment | |  |  |  |  | 4 to 8 weeks (Efficacy 12.50%, 95% CI -82.89 to 107.89) or in | |  |  |  |  |  |
|  |  |  |  | (non -breastfeeding) | |  |  |  |  | low birth weight (RR 1.14; 95% CI 0.85 | |  |  |  |  |  |
|  |  |  |  |  | |  |  |  |  | to 1.53) or prematurity (RR 1.05, 95% CI 0.83 to 1.32). | |  |  |  |  |  |
|  |  |  |  |  |  |  |  |  |  |  |  |  |  |  |  |  |
|  |  |  |  |  | |  | |  | |  | | Stillbirth | | No significant difference in the risks (RR 2.99; |  |  |
|  |  |  |  |  |  |  |  |  |  |  |  |  |  | 95% CI 0.12 to 73.33), |  |  |
|  |  |  |  |  | | SAINT | | HIV | | No significant difference in HIV | |  | |  |  |  |
|  |  |  |  |  |  |  |  | infection | | infection rates at 4 to 8 weeks (Efficacy 24.39%; 95% CI -4.15 to 52.93) | |  |  |  |  |  |
|  |  |  |  |  | |  | | LBW | | No significance (RR 1.41; 95% CI 0.45 to 4.42) | | Infant death rates | | 4 to 8 weeks 1.01 (0.54 to 1.89) |  |  |
|  |  |  |  |  | | Gray 2006 | | HIV infection | | No significant difference at | | Infant mortality | | No significant difference in the first |  |  |
|  |  |  |  |  |  |  |  |  |  | birth (Efficacy 26.65%; 95% CI -122.44 to 175.74), | |  |  | 6 months (RR 2.97; 95% CI 0.83 to 10.61). |  |  |
|  |  |  |  |  |  |  |  |  |  | 4 to 8 weeks | |  |  |  |  |  |
|  |  |  |  |  |  |  |  |  |  | (Efficacy -120.05%; 95% CI -301.07 to 60.97), | |  |  |  |  |  |
|  |  |  |  |  |  |  |  |  |  | 3 to 4 months | |  |  |  |  |  |
|  |  |  |  |  |  |  |  |  |  | (Efficacy -144.51%; 95% CI -329.90 to 40.88) | |  |  |  |  |  |
|  |  |  |  |  |  |  |  |  |  | 6 months | |  |  |  |  |  |
|  |  |  |  |  |  |  |  |  |  | (Efficacy -116.07%; 95% CI -280.57 to 48.43). | |  |  |  |  |  |
|  |  |  |  |  | | Gray 2006 b: ddI versus ZDV | | HIV infection | | No significant difference | | Infant mortality | | No significant difference 6 months (RR 1.94; 95% CI 0.50 to 7.52). |  |  |
|  |  |  |  |  |  |  |  |  |  | at birth (Efficacy 52.66%; 95% CI -88.01 to 193.33), | |  |  |  |  |  |
|  |  |  |  |  |  |  |  |  |  | 4 to 8 weeks (Efficacy -42.02%; 95% CI -205.79 to 121.75), | |  |  |  |  |  |
|  |  |  |  |  |  |  |  |  |  | 3 to 4 months (Efficacy -113.03%; 95% CI -290.84 to 64.78) | |  |  |  |  |  |
|  |  |  |  |  |  |  |  |  |  | 6 months (Efficacy -89.29%; 95%CI -247.73 to 69.15). | |  |  |  |  |  |
|  |  |  |  |  | | Gray 2006 c: d4T plus ddI versus ZDV | | HIV infection | | No significant difference | | Infant mortality | | No significant difference in the first 6 months (RR 0.66; 95% CI 0.11 to 3.86). |  |  |
|  |  |  |  |  |  |  |  |  |  | at birth (Efficacy 49.43%; 95%CI -94.88 to 193.74), | |  |  |  |  |  |
|  |  |  |  |  |  |  |  |  |  | 4 to 8 weeks | |  |  |  |  |  |
|  |  |  |  |  |  |  |  |  |  | (Efficacy 24.15%; 95% CI -127.10 to 175.40), | |  |  |  |  |  |
|  |  |  |  |  |  |  |  |  |  | 3 to 4 months | |  |  |  |  |  |
|  |  |  |  |  |  |  |  |  |  | (Efficacy -1.14%; 95% CI -158.84 to 156.56) | |  |  |  |  |  |
|  |  |  |  |  |  |  |  |  |  | 6 months (Efficacy | |  |  |  |  |  |
|  |  |  |  |  |  |  |  |  |  | 17.86%; 95% CI -118.79 to 154.51). | |  |  |  |  |  |
|  |  |  |  |  | | Kiarie 2003 | | HIV infection | | No significant difference at 4 to 8 weeks | | Infant mortality | | No significant difference in first 4 to 8 weeks |  |  |
|  |  |  |  |  |  |  |  |  |  | (Efficacy 59.09%; 95% CI -3.90 to 122.08). | |  |  | (RR 0.99; 95% CI 0.21 to 4.72), |  |  |
|  |  |  |  |  | |  | | Prematurity | | No significant difference (RR 1.97; 95% CI 0.37 to 10.42) | | Stillbirth | | No significant difference (RR 1.48; 95% CI 0.25 to 8.58), |  |  |
|  |  |  |  |  |  |  |  |  |  |  |  |  |  |  |  |  |
|  |  |  |  |  | |  | | LBW | | No significant difference (RR 1.97; 95% CI 0.18 to 21.24). | |  | |  |  |  |
|  |  |  |  |  |  |  |  |  |  |  |  |  |  |  |  |  |
|  |  |  |  |  | | Gray 2005 | | HIV infection | | No significant difference at 4-8 weeks (Efficacy 35.37%; 95% CI -10.00 to 80.74) | | Infant mortality | | No significant difference at first 4 to 8 weeks was also similar in the two groups (RR 1.15; 95% CI 0.52 to 2.54) |  |  |
|  |  |  |  |  |  |  |  |  |  | and 3 to 4 months (Efficacy 39.69%; 95% CI -1.08 to 80.46). | |  |  |  |  |  |
|  |  |  |  |  |  |  |  |  |  |  |  |  |  |  |  |  |
|  |  |  |  |  | | Mashi | | For HIV infection | | At birth, there were no differences between the groups (Efficacy -64.38%, 95%CI -183.72 to 54.96). | | Infant deaths | | In the first month not statistically |  |  |
|  |  |  |  |  |  |  |  |  |  | At one | |  |  | different between groups (RR 0.54, 95%CI 0.22 to 1.35). |  |  |
|  |  |  |  |  |  |  |  |  |  | month post-partum not statistically significantly | |  |  |  |  |  |
|  |  |  |  |  |  |  |  |  |  | different between the groups (Efficacy -16.22%; 95%CI | |  |  |  |  |  |
|  |  |  |  |  |  |  |  |  |  | -102.24 to 69.80). | |  |  |  |  |  |
|  |  |  |  |  |  |  |  |  |  |  |  |  |  |  |  |  |
|  |  |  |  |  | |  | | Premature | | No difference in the incidence of | | Stillbirth rates | | Similar in both groups (RR 1.50, 95%CI 0.43 |  |  |
|  |  |  |  |  |  |  |  | delivery | | (RR = 1.00, 95%CI 0.40 to 2.50). | |  |  | to 5.28) |  |  |
|  |  |  |  |  |  |  |  |  |  |  |  |  |  |  |  |  |
|  |  |  |  | TRIPLE regimens versus other | | Chung 2008 | | HIV infection | | No babies were infected with HIV at birth in either group; at 6months post-delivery there was no statistically significant difference | | Infant deaths | | No infant deaths | There was no statistically significant difference at |  |
|  |  |  |  | (Breastfeeding) | |  |  |  |  | in HIV infection between groups (Efficacy -84.62%, 95%CI: | |  |  | in the TRIPLE arm and one at 2 weeks from sepsis (RR 0.31, | two weeks (Efficacy |  |
|  |  |  |  |  | |  |  |  |  | -490.35 to 321.11). | |  |  | 95%CI 0.01 to 7.30). | -84.62%, 95%CI: -490.35 to 321.11), or at 12months (Efficacy |  |
|  |  |  |  |  | |  |  |  |  |  | |  |  |  | 35.90%; 95%CI: -118.33 to 190.13). |  |
|  |  |  |  |  | |  | | Weighing less than | | There were five infants weighing less than | | Stillbirths | | There were two stillbirths |  |  |
|  |  |  |  |  |  |  |  | 2.5kg | | 2.5kg in the TRIPLE group and none in the ZDV/sdNVP group | |  |  | in the TRIPLE group and one in the ZDV/sdNVP group |  |  |
|  |  |  |  |  |  |  |  |  | | (RR 10.29, 95%CI 0.59 to 177.97) but this was not statistically | |  |  | (RR 1.87, 95%CI 0.18 to 19.47). |  |  |
|  |  |  |  |  |  |  |  |  |  |  |  |  |  |  |  |  |
|  |  |  |  | TRIPLE regimens versus other | | Kesho Bora | | HIV infection | | No statistically significant difference between groups in HIV infection at birth (Efficacy 18.18%, 95%CI -83.48 to | | Infant deaths | | No statistical significance between groups (RR 2.00, | The incidence of HIV infection or death at four to eight weeks was higher in the non- |  |
|  |  |  |  | (mixed feeding) | |  |  |  |  | 119.84) or at four to eight weeks (Efficacy 31.25%, 95%CI - | |  |  | 95%CI 0.37 to 10.88), nor in the first 4 to 8 weeks (RR 0.86, | TRIPLE group (RR 20.00, 95%CI -34.13 to 74.13) but this was |  |
|  |  |  |  |  | |  |  |  |  | 29.29 to 91.79). | |  |  | 95%CI 0.29 to 2.53), or first 6 months (RR 0.78, 95%CI 0.43 to 1.43). | not statistically significant. At 6 months, the HIV infection or |  |
|  |  |  |  |  | |  |  |  |  |  | |  |  | In the first 12months,more babies died in the non-TRIPLE group although this difference was not statistically significant (RR 0.65, 95%CI 0.40 to 1.07). | death incidence remained higher in the non-TRIPLE group (RR 34.13, 95%CI [-0.29 to 68.55) and at 12 months this difference was statistically significant (RR 36.20, 95%CI 5.92 to 66.48). |  |
|  |  |  |  |  | |  |  |  |  | At 6 months, HIV infection higher but not statistically significantly so in the non-TRIPLE group (Efficacy | |  |  |  |  |  |
|  |  |  |  |  | |  |  |  |  | 42.35%, 95%CI -0.57 to 85.27). | |  |  |  |  |  |
|  |  |  |  |  |  |  |  |  |  |  |  |  |  |  |  |  |
|  |  |  |  |  | |  |  |  |  | At 12 months HIV infection was statistically significantly higher in the non-TRIPLE group | |  |  |  |  |  |
|  |  |  |  |  | |  |  |  |  | (Efficacy = 42.11%, 95%CI 0.66 to 83.56). | |  |  |  |  |  |
|  |  |  |  |  |  |  |  |  |  |  |  |  |  |  |  |  |
|  |  |  |  |  | |  | | Weighing less than 2.5kg | | No statistical difference in those weighing less than 2.5kg (RR 1.25, 95%CI 0.86 | | Stillbirth | | Same between groups (RR |  |  |
|  |  |  |  |  |  |  |  |  |  | to 1.82). | |  |  | 1.00, 95%CI 0.25 to 3.95). |  |  |
|  |  |  |  |  |  |  |  |  |  |  |  |  |  |  |  |  |
|  |  |  |  |  | |  | | Prematurity | | No statistical difference (RR 1.44, 95%CI 0.93 to | |  | |  |  |  |
|  |  |  |  |  |  |  |  |  |  | 2.24) or | |  |  |  |  |  |
|  |  |  |  |  |  |  |  |  |  |  |  |  |  |  |  |  |
|  |  |  |  | TRIPLE regimens versus other | | Mma Bana | | HIV infection | | There was no significant difference in HIV infection rates at birth | | Infant dying | | The differences between groups in the number |  |  |
|  |  |  |  | Breastfeeding | |  |  |  |  | (Efficacy -189.47%; 95%CI -715.29 to 336.35) with three babies | |  |  | of infants dying in the first eight days after birth (RR 1.96; 95%CI |  |  |
|  |  |  |  |  | |  |  |  |  | infected in the TZV group and one in the CBV group. | |  |  | 0.18 to 21.53) and in the first six months after birth (RR 0.98; |  |  |
|  |  |  |  |  | |  |  |  |  | At three | |  |  | 95%CI 0.35 to 2.76) were not significantly different. |  |  |
|  |  |  |  |  | |  |  |  |  | months the incidence in infant HIV was still not significantly | |  |  |  |  |  |
|  |  |  |  |  | |  |  |  |  | different (5 versus 1) between groups (Efficacy -390.88%; 95%CI | |  |  |  |  |  |
|  |  |  |  |  | |  |  |  |  | -981.63 to 199.87). | |  |  |  |  |  |
|  |  |  |  |  |  |  |  |  |  |  |  |  |  |  |  |  |
|  |  |  |  |  |  |  |  |  |  |  |  |  |  |  |  |  |
|  |  |  |  |  | |  | | Prematurity | | Incidence of | | Stillbirth | | Stillbirth rates did not differ. Statistically significant |  |  |
|  |  |  |  |  |  |  |  |  |  | prematurity defined as gestational age < 37 weeks was higher in the | |  |  | between the groups (RR 1.54; 95%CI 0.51 to 4.66). |  |  |
|  |  |  |  |  |  |  |  |  |  | CBV group (RR 0.66; 95%CI 0.47 to 0.95) but not statistically | |  |  |  |  |  |
|  |  |  |  |  |  |  |  |  |  | significant | |  |  |  |  |  |
|  |  |  |  |  |  |  |  |  |  |  |  |  |  |  |  |  |
|  |  |  |  |  | |  | | LBW less than 2.5kg | | Low birth weight less than 2.5kg was also higher in the | |  | |  |  |  |
|  |  |  |  |  |  |  |  |  |  | CBV group but was not statistically significant (RR 0.79; 95%CI 0.53 to 1.19). | |  |  |  |  |  |
| **Child Birth** |  |  |  |  | |  | |  | |  | |  | |  |  |  |
| Antibiotics for preterm rupture of membranes (Review) | Kenyon, S **42** | 2010 | To evaluate the immediate and long-term effects of administering antibiotics to women with PROM before 37 weeks, on maternal infectious morbidity, neonatal morbidity and mortality, and longer-term childhood development. | Any antibiotic versus placebo | | Chorioamnionitis | | RR 0.66; 95% CI 0.46-0.96 | |  | |  | |  |  |  |
|  |  |  |  |  | | Babies born within 48 hours | | RR 0.71; 95% CI 0.58-0.87 | |  | |  | |  |  |  |
|  |  |  |  |  | | Neonatal infection | | RR 0.67; 95% CI 0.52-0.85 | |  | |  | |  |  |  |
|  |  |  |  |  | | Days in neonatal ICU (mean difference (MD) (days spent)) | | --5.05; 95% CI -9.77- -0.33 | |  | |  | |  |  |  |
|  |  |  |  |  | | Abnormal cerebral ultrasound scan prior to discharge from hospital | | RR 0.81; 95% CI 0.68-0.98 | |  | |  | |  |  |  |
|  |  |  |  | Erythromycin versus co-amoxyclav | | Delivery within 48 hours | | RR 1.14; 95% CI 1.02-1.28 | |  | |  | |  |  |  |
|  |  |  |  |  |  |  |  |  |  |  |  |  |  |  |  |  |
|  |  |  |  |  |  |  |  | (less common after co-amoxyclav | |  |  |  |  |  |  |  |
|  |  |  |  |  | | Necrotising enterocolitis | | RR 0.46; 95% CI 0.23-0.94 | |  | |  | |  |  |  |
|  |  |  |  |  |  |  |  |  |  |  |  |  |  |  |  |  |
|  |  |  |  |  |  |  |  | (Less frequent after erythromycin | |  |  |  |  |  |  |  |
| Antibiotics for pre-term pre-labour rupture of membranes: prevention of neonatal deaths due to complications of pre-term birth and infection | Cousens, S **43** | 2010 | To review the evidence for and estimate the effect on neonatal mortality due to pre-term birth complications or infection, of administration of antibiotics to women with pPROM, in low and middle-income countries | Severe morbidity | | Respiratory distress syndrome | | RR 0.88 (0.80-0.97) | | All-cause neonatal mortality/mortality before discharge | |  | | RR 0.90 (0.72-1.12) |  |  |
|  |  |  |  |  | | Necrotising enterocolitis | | RR 0.76 (0.56-1.05) | |  | |  | |  |  |  |
|  |  |  |  |  | | Intra-ventricular haemorrhage | | RR 0.67 (0.49-0.92) | |  | |  | |  |  |  |
|  |  |  |  |  | | Confirmed sepsis | | RR 0.61 (0.48-0.77) | |  | |  | |  |  |  |
| Different corticosteroids and regimens for accelerating fetal lung maturation for women at risk of preterm birth | Brownfoot, FC **44** | 2009 | To assess the effects of different corticosteroid regimens for women at risk of preterm birth | Dexamethasone versus betamethasone | | Intraventricular haemorrhage | | RR 0.44 (0.21-0.92) | |  | |  | |  |  |  |
|  |  |  |  |  |  |  |  |  |  |  |  |  |  |  |  |  |
|  |  |  |  |  |  |  |  | Dexamethasone reduced risk | |  |  |  |  |  |  |  |
|  |  |  |  |  | | Severe intraventricular haemorrhage | | RR 0.40 (0.13-1.24) | |  | |  | |  |  |  |
|  |  |  |  |  | |  | |  | |  | |  | |  |  |  |
|  |  |  |  |  | | Infants admitted to neonatal ICU | | RR 3.83 (1.24-11.87) | |  | |  | |  |  |  |
|  |  |  |  |  |  |  |  | (more dexamethasone admitted) | |  |  |  |  |  |  |  |
|  |  |  |  |  | | Neonatal sepsis | | RR 1.30 (0.78-2.19) | |  | |  | |  |  |  |
|  |  |  |  |  | | Necrotising enterocolitis | | RR 1.29 (0.38-4.40) | |  | |  | |  |  |  |
|  |  |  |  |  | | Retinopathy of prematurity | | RR 0.93 (0.59-1.47) | |  | |  | |  |  |  |
|  |  |  |  |  | | Patent ductus arteriosus | | RR 1.19 (0.56-2.49) | |  | |  | |  |  |  |
|  |  |  |  |  | | Apgar score at 5 min | | MD -0.20 (-0.89-0.49), 1 trial | |  | |  | |  |  |  |
|  |  |  |  |  | | Apgar score < 7 at 5 min | | RR 0.97 (0.43-2.18_ | |  | |  | |  |  |  |
|  |  |  |  |  | | Head circumference | | MD -0.50 (-1.55-0.55) | |  | |  | |  |  |  |
|  |  |  |  |  | | Vasopressor use | | RR 0.44 (0.17-1.11) | |  | |  | |  |  |  |
|  |  |  |  |  | | Dexamethasone (oral versus intramuscular injection), 1 trial | |  | |  | |  | |  |  |  |
|  |  |  |  |  | | Intraventricular haemorrhage | | RR 4.24 (0.96-18.33) | |  | |  | |  |  |  |
|  |  |  |  |  |  |  |  | No diff | |  |  |  |  |  |  |  |
|  |  |  |  |  | | <34 weeks gestation at birth | | RR 4.92 (1.12-21.55) | |  | |  | |  |  |  |
|  |  |  |  |  |  |  |  |  |  |  |  |  |  |  |  |  |
|  |  |  |  |  |  |  |  | In favour of IM route | |  |  |  |  |  |  |  |
|  |  |  |  |  | | Respiratory distress syndrome | | RR 1.15 (0.75-1.77) | |  | |  | |  |  |  |
|  |  |  |  |  | | Perinatal death | | RR 1.48 (0.45-4.90) | |  | |  | |  |  |  |
|  |  |  |  |  | | Birth weight | | RR MD -0.05 (-0.27-0.17), kg | |  | |  | |  |  |  |
|  |  |  |  |  | | Neonatal sepsis | | RR 8.48 (1.121-64.93) | |  | |  | |  |  |  |
|  |  |  |  |  |  |  |  |  |  |  |  |  |  |  |  |  |
|  |  |  |  |  |  |  |  | Higher in oral | |  |  |  |  |  |  |  |
|  |  |  |  |  | | All neonatal sepsis occurring < 34 weeks | | RR 5.09 (0.63-41.45) | |  | |  | |  |  |  |
|  |  |  |  |  | | Betamethasone acetate+phosphate versus betamethasone phosphate, 1 trial | |  | |  | |  | |  |  |  |
|  |  |  |  |  | | Respiratory distress syndrome | | RR 0.19 (0.01-3.91) | |  | |  | |  |  |  |
|  |  |  |  |  |  |  |  | (NO sig differ) | |  |  |  |  |  |  |  |
|  |  |  |  |  | | Intraventricular haemorrhage | | RR 0.32 (0.01-7.69) | |  | |  | |  |  |  |
|  |  |  |  |  | | Perinatal death | | RR 0.32 (0.01-7.69) | |  | |  | |  |  |  |
|  |  |  |  |  | | LBW | | RR 1.21 (0.86-1.72) | |  | |  | |  |  |  |
|  |  |  |  |  | | Birth weight | | MD -0.10 (-0.44-0.24), kg | |  | |  | |  |  |  |
|  |  |  |  |  | | Transfer to NICU (for respiratory distress) | | RR 0.11 (0.01-1.93) | |  | |  | |  |  |  |
|  |  |  |  |  |  |  |  |  |  |  |  |  |  |  |  |  |
|  |  |  |  |  |  |  |  | In favour of two meds | |  |  |  |  |  |  |  |
| Antenatal steroids in preterm labour for | Mwansa-Kambafwile, J **45** | 2010 | To review the evidence for and estimate the effect on cause-specific |  | |  | |  | | Effects on neonatal deaths due to direct complications of preterm birth (<36 weeks of gestation) | |  | |  |  |  |
| the prevention of neonatal deaths due to |  |  | neonatal mortality of administration of antenatal steroids to women with anticipated preterm labour, with additional analysis for the effect in low- and middle-income countries. |  |  |  |  |  |  |  |  |  |  |  |  |  |
| complications of preterm birth |  |  |  |  |  |  |  |  |  |  |  |  |  |  |  |  |
|  |  |  |  |  | |  | |  | | Antenatal steroids preterm | | Neonatal mortality | | RR 0.69 (0.58-0.81) |  |  |
|  |  |  |  |  | |  | |  | | Very preterm | |  | | RR 0.66 (0.56-0.78) |  |  |
|  |  |  |  |  | |  | |  | | Variation of mortality effect size in LMICs | |  | | RR 0.47 (0.35-0.64) Larger reduction in neonatal mortality in middle-income vs high-income settings |  |  |
|  |  |  |  | Respiratory distress syndrome | |  | | RR 0.63(0.49-0.81) | |  | |  | |  |  |  |
| Magnesium sulphate versus phenytoin for eclampsia (Review) | Duley, L **46.** | 2010 | To assess the effects of magnesium sulphate compared with phenytoin when used for the care of women with eclampsia. | Recurrence of seizures | |  | | RR 0.34 (0.24-0.49) | | Maternal mortality | | Overall | | RR 0.50 (0.24-1.05) |  |  |
|  |  |  |  |  |  |  |  |  |  |  |  |  |  |  |  |  |
|  |  |  |  |  |  |  |  | (favoured for MgS) | |  |  |  |  | (Favours Mgs over phenytoin although the difference is not stat sign. |  |  |
|  |  |  |  | Cerebrovascular accident | |  | | RR 0.54 (0.20-1.46) | | Stillbirth | |  | | RR 0.83 (0.61-1.13) |  |  |
|  |  |  |  |  |  |  |  |  |  |  |  |  |  |  |  |  |
|  |  |  |  |  |  |  |  | No sign difference | |  |  |  |  |  |  |  |
|  |  |  |  |  |  |  |  |  |  |  |  |  |  |  |  |  |
|  |  |  |  |  |  |  |  | One trial | |  |  |  |  |  |  |  |
|  |  |  |  | Any serious morbidity (stroke, renal failure, HELLP/DIC, pulmonary oedema, cardiac arrest, or as reported) | |  | | RR 0.94 (0.73-1.20) | | Perinatal death | |  | | RR 0.85 (0.67-1.09) |  |  |
|  |  |  |  |  |  |  |  |  |  |  |  |  |  |  |  |  |
|  |  |  |  |  |  |  |  | No significant difference | |  |  |  |  |  |  |  |
|  |  |  |  | Serious maternal morbidity | | Pneumonia | | RR 0.44 (0.24-0.79) | | Neonatal death | |  | | RR 0.95 (0.59-1.53) |  |  |
|  |  |  |  |  |  |  |  |  |  |  |  |  |  |  |  |  |
|  |  |  |  |  |  |  |  | (Reduced risk for women allocated to MgS) | |  |  |  |  |  |  |  |
|  |  |  |  |  | | Renal failure | | RR 1.52 (0.98-2.36) | | Death or in special care baby unit for more than 7 days | | RR 0.77 (0.63-0.95) | |  |  |  |
|  |  |  |  |  |  |  |  |  |  |  |  |  |  |  |  |  |
|  |  |  |  |  |  |  |  | (increased risk for MgS; diff not sign diff) | |  |  |  |  |  |  |  |
|  |  |  |  |  | | Liver failure, HELPP syndrome, coagulopathy, pulmonary oedema, respiratory depression, cardiac arrest. | |  | |  | |  | |  |  |  |
|  |  |  |  | ICU admission | |  | | RR 0.67 (0.50-0.89) | |  | |  | |  |  |  |
|  |  |  |  |  |  |  |  |  |  |  |  |  |  |  |  |  |
|  |  |  |  |  |  |  |  | (reduced for MgS) | |  |  |  |  |  |  |  |
|  |  |  |  | Ventilation | |  | | RR 0.68 (0.50-0.91) | |  | |  | |  |  |  |
|  |  |  |  |  |  |  |  | (reduced for MgS) | |  |  |  |  |  |  |  |
|  |  |  |  | APGAR scores (below 8 at 1 min) | |  | | RR 0.78 (0.66-0.93) | |  | |  | |  |  |  |
|  |  |  |  |  |  |  |  | In favour of MgS | |  |  |  |  |  |  |  |
|  |  |  |  | APGAR scores (below 8 at 5 min) | |  | | RR 0.86 (0.52-1.43) | |  | |  | |  |  |  |
|  |  |  |  |  |  |  |  |  |  |  |  |  |  |  |  |  |
|  |  |  |  |  |  |  |  | No difference | |  |  |  |  |  |  |  |
|  |  |  |  | Admission to a special care baby unit | |  | | RR 0.73 (0.58-0.91) | |  | |  | |  |  |  |
|  |  |  |  |  |  |  |  |  |  |  |  |  |  |  |  |  |
|  |  |  |  |  |  |  |  | (Less likely) | |  |  |  |  |  |  |  |
| Are birth kits a good idea? A systematic review of the evidence | Hundley, V 47 | 2012 | To identify the current state of knowledge regarding the effects of births kits on clean birth | Components of the birth kits | | Sepsis | | RR=0.12 (95%CI0.02,0.93) | | Perinatal mortality rate | | OR=0.70 (95%CI0.59,0.82) Jokhio | |  |  |  |
|  |  |  | practices and on new born and maternal outcomes |  |  |  | | Garner | |  | |  | |  |  |  |
|  |  |  |  |  |  |  | |  | |  | | OR=0.69 (95%CI0.57,0.83) | |  |  |  |
|  |  |  |  |  |  |  | |  | |  | | Jokhio | |  |  |  |
|  |  |  |  |  |  |  | | RR=0.49 (95%CI 0.43,0.56) Mullany, | | Stillbirth | |  | |  |  |  |
|  |  |  |  |  |  | Omphalitis | | OR=0.42 (95%CI 0.18,0.97) Darmstadt | |  | | OR=0.71 (95%CI0.62,0.83) | |  |  |  |
|  |  |  |  |  |  |  | | OR=0.08 (95%CI 0.03,0.19) Winani | |  | | Jokhio | |  |  |  |
|  |  |  |  |  |  |  | | RR= 0.45 (95%CI 0.25 to 0.81) | |  | | RR=0.17 (95%CI0.13,0.23) | |  |  |  |
|  |  |  |  |  |  |  | | Tsu | |  | | Meegan | |  |  |  |
|  |  |  |  |  |  |  | |  | | Neonatal Mortality | |  | |  |  |  |
|  |  |  |  |  |  |  | |  | |  | | NNMR in 4years =21.83% | |  |  |  |
|  |  |  |  |  |  |  | | OR=0.17 (95%CI0.13,0.23) Jokhio | |  | | (Kapoor) | |  |  |  |
|  |  |  |  |  |  |  | | OR=0.11 (95%CI0.01,1.06) Darmstadt | |  | |  | |  |  |  |
|  |  |  |  |  |  |  | | OR=0.31 (95%CI0.18,0.54) Winani | |  | | RR=0.01 (95%CI0.001,0.09) | |  |  |  |
|  |  |  |  |  |  |  | |  | |  | | Meegan | |  |  |  |
|  |  |  |  |  |  |  |  |  |  |  |  |  |  |  |  |  |
|  |  |  |  |  |  | puerperal sepsis | | OR=0.61 (95%CI0.47,0.79) Jokhio | |  | |  | |  |  |  |
|  |  |  |  |  |  |  | |  | |  | | OR=0.74 (95%CI0.45,1.23) | |  |  |  |
|  |  |  |  |  |  |  | |  | |  | | Jokhio | |  |  |  |
|  |  |  |  |  |  |  |  |  |  |  |  |  |  |  |  |  |
|  |  |  |  |  |  |  |  |  |  |  |  |  |  |  |  |  |
|  |  |  |  |  |  |  |  |  |  |  |  |  |  |  |  |  |
|  |  |  |  |  |  |  |  |  |  |  |  |  |  |  |  |  |
|  |  |  |  |  |  |  | |  | | Tetanus related mortality | |  | |  |  |  |
|  |  |  |  |  |  |  |  |  |  |  |  |  |  |  |  |  |
|  |  |  |  |  |  |  |  |  |  |  |  |  |  |  |  |  |
|  |  |  |  |  |  | haemorrhage | |  | |  | |  | |  |  |  |
|  |  |  |  |  |  |  |  |  |  |  |  |  |  |  |  |  |
|  |  |  |  |  |  |  | |  | | Maternal mortality | |  | |  |  |  |
|  |  |  |  |  | |  | |  | |  | | Community mobilisation and home based neonatal reduction | | RR 0.66 (0.47-0.93) |  |  |
|  |  |  |  |  | |  | |  | |  | | TBA trained and home visits | | RR 0.79 (0.63-1.01) |  |  |
|  |  |  |  |  | |  | |  | | Early neonatal mortality | |  | | RR 0.74 (0.64-0.86) |  |  |
|  |  |  |  |  | |  | |  | |  | | Training TBAs who made home visits antenatally + intrapartum | | RR 0.85 (0.52-1.39) |  |  |
|  |  |  |  |  | |  | |  | |  | | Community support groups/ women’s groups | | RR 0.76 (0.58-0.98) |  |  |
|  |  |  |  |  | |  | |  | |  | | Community mobilisation and antenatal and postnatal visits | | RR 0.81 (0.69-0.94) |  |  |
|  |  |  |  |  | |  | |  | |  | | Home based neonatal care | | RR 0.45(0.28-0.72) |  |  |
|  |  |  |  |  | |  | |  | | Late neonatal mortality | |  | | RR 0.72 (0.65-0.83) |  |  |
| The effect of providing skilled birth attendance | Yakoob, M 56**.** | 2011 | To review available evidence to ascertain |  | |  | |  | | Still Births | | Role of birth attendance | | RR 0.77 (0.69-0.85) |  |  |
| and emergency obstetric care in preventing |  |  | the effect of provision of skilled birth attendance as well as basic and emergency obstetric care on stillbirths |  |  |  |  |  |  |  |  |  |  | 2 studies significantly reduced |  |  |
| stillbirths |  |  |  |  |  |  |  |  |  |  |  |  |  |  |  |  |
|  |  |  |  |  | |  | |  | | Perinatal mortality | |  | | RR 0.88 (0.82-0.95) |  |  |
|  |  |  |  |  | |  | |  | |  | | Provision of basic and comprehensive emergency obstetric care | | Could avert 45%-75% intrapartum still births although IDR fell between 30% -40% (Delphi) |  |  |
| Traditional birth attendant training for improving health behaviours and pregnancy outcomes (Review) | Sibley, L **62** | 2009 | To assess the effects of TBA training on health behaviours and pregnancy outcomes. | TBA or maternal behaviours thought to mediate positive pregnancy | |  | |  | | Maternal mortality | | Difference in favour of women living in intervention clusters (non-sign) | | OR 0.74 (0.45-1.22) |  |  |
|  |  |  |  |  |  |  |  |  |  |  |  |  |  |  |  |  |
|  |  |  |  | Advice about immediate feeding of colostrum | | Advice on immediate feeding (3 months) | | OR 1.21 (0.39-3.79) | | Perinatal death | |  | | OR 1.02 (0.59-1.76) |  |  |
|  |  |  |  |  |  |  |  | (increase in percentage of intervention of TBAs who advised on immediate feeding) | |  |  |  |  |  |  |  |
|  |  |  |  |  | | Advice on immediate feeding (7 months) | | OR 1.37 (0.62-3.03) | | Stillbirth | |  | | OR 0.69 (0.57-0.83) |  |  |
|  |  |  |  |  |  |  |  |  |  |  |  |  |  | One study |  |  |
|  |  |  |  | Advice about introduction of complementary foods | | At 3 months | | OR 3.11 (1.63-5.92) | | Neonatal death | |  | | OR 0.71 (0.61-0.82) |  |  |
|  |  |  |  |  |  |  |  |  |  |  |  |  |  |  |  |  |
|  |  |  |  |  |  |  |  | Effect-size estimates of the post-test difference at this time point favour the intervention group. | |  |  |  |  | 1 study |  |  |
|  |  |  |  |  | | At 7 months | | OR 2.07 (1.10-3.90) | |  | |  | |  |  |  |
|  |  |  |  | Frequency of referral | |  | | Adjusted OR 1.50 (1.18-1.90)** | |  | |  | |  |  |  |
|  |  |  |  | Obstructed labour | |  | | OR 1.26 (1.03-1.54) | |  | |  | |  |  |  |
|  |  |  |  |  |  |  |  |  |  |  |  |  |  |  |  |  |
|  |  |  |  |  |  |  |  | (higher in women living in intervention clusters | |  |  |  |  |  |  |  |
|  |  |  |  | Frequency of haemorrhage, frequency of postpartum haemorrhage and mean volume of blood loss | |  | | OR 0.61 (0.47-0.79) | |  | |  | |  |  |  |
|  |  |  |  |  |  |  |  |  |  |  |  |  |  |  |  |  |
|  |  |  |  |  |  |  |  | Lower in women living in intervention clusters | |  |  |  |  |  |  |  |
|  |  |  |  |  | | Postpartum haemorrhage | | OR 0.94 (0.76-1.17) | |  | |  | |  |  |  |
|  |  |  |  |  |  |  |  |  |  |  |  |  |  |  |  |  |
|  |  |  |  |  |  |  |  | (non-significant difference) | |  |  |  |  |  |  |  |
|  |  |  |  |  | | Mean blood loss (mean weight difference) | | MWD 2.00 (-7.39-11.39) | |  | |  | |  |  |  |
|  |  |  |  | Frequency of puerperal sepsis | |  | | OR 0.17 (0.13-0.23) | |  | |  | |  |  |  |
|  |  |  |  |  |  |  |  |  |  |  |  |  |  |  |  |  |
|  |  |  |  |  |  |  |  | (significance lower in intervention cluster) | |  |  |  |  |  |  |  |
| 60 million non-facility births: Who can deliver in community settings to reduce intrapartum-related deaths? | Darmastadt, G 63 | 2010 | To review the evidence for the effect of care by different community cadres | Skilled child birth care in the community | |  | |  | |  | |  | |  |  |  |
|  |  |  | during pregnancy and childbirth |  |  |  |  |  |  |  |  |  |  |  |  |  |
|  |  |  |  | Training skills competency of community based SBAs | |  | |  | |  | |  | |  |  |  |
|  |  |  |  | Community midwives: intrapartum related mortality effect | |  | |  | |  | |  | |  |  |  |
|  |  |  |  |  |  |  |  |  |  |  |  |  |  |  |  |  |
|  |  |  |  |  |  |  |  |  |  |  |  |  |  |  |  |  |
|  |  |  |  |  |  |  |  |  |  |  |  |  |  |  |  |  |
|  |  |  |  |  |  |  |  |  |  |  |  |  |  |  |  |  |
|  |  |  |  |  |  |  |  |  |  |  |  |  |  |  |  |  |
|  |  |  |  |  |  |  |  |  |  |  |  |  |  |  |  |  |
|  |  |  |  | Effect of community skilled birth attendance on intrapartum related mortality meta-analysis | |  | |  | | Peri-natal mortality | | Training community SBAs | | RR 0.88 (0.83-0.95) |  |  |
|  |  |  |  |  |  |  |  |  |  | ENMR | |  | | RR 0.87 (0.79-0.97) |  |  |
|  |  |  |  | Training TBAs for providing labour and childbirth care | |  | |  | |  | |  | |  |  |  |
|  |  |  |  | Trained TBAs for the prevention of intrapartum-related mortality | |  | |  | |  | |  | |  |  |  |
|  |  |  |  | Trained TBAs for the prevention, recognition and referral of the non-breathing baby | |  | |  | |  | |  | |  |  |  |
|  |  |  |  | TBS training programs: intrapartum –related mortality effect | |  | |  | |  | |  | |  |  |  |
|  |  |  |  | Using CHWs to promote birth preparedness and care-seeking with or without provision of new baby care at birth | |  | |  | |  | |  | |  |  |  |
|  |  |  |  | Integrated CHW packages: meta-analysis of effect on intrapartum related mortality | |  | |  | | Positive prevention of intrapartum related outcomes through CHWs | | PMR | | RR 0.72 (0.62-0.84) |  |  |
| **Neonate** |  |  |  |  | |  | |  | |  | |  | |  |  |  |
| Kangaroo mother care to reduce morbidity and mortality in low birthweight infants (Review) | Conde-Agudelo, A **59** | 2011 | To determine whether there is evidence to support the use of KMC in LBW infants as an alternative to conventional neonatal care | KMC versus conventional neonatal care | |  | |  | | KMC versus conventional neonatal care | |  | |  |  |  |
|  |  |  |  | Infection/illness | | In stabilised LBW infants at latest follow-up | | RR 0.57 (0.40-0.80) | | Mortality | | Risk of mortality at discharge or 40-41 weeks post menstrual age | | RR 0.60 (0.39-0.93) |  |  |
|  |  |  |  |  |  |  |  |  | |  |  |  |  | (reduction) |  |  |
|  |  |  |  |  |  |  |  | (Reduction in severe infection/sepsis at | |  |  |  |  |  |  |  |
|  |  |  |  |  | | Severe illness at 6 months f/u | | RR 0.30 (0.14-0.67) | |  | | Continuous KMC | | RR 0.60 (0.38-0.96) |  |  |
|  |  |  |  |  | | Nosocomial infections/sepsis at discharge or 40-41 weeks’ gestational age | | RR 0.472 (0.24-0.73) | |  | | Initiated within 10 days post-birth | | RR 0.57 (0.36-0.89) |  |  |
|  |  |  |  |  | | LRTI at 6 months f/u | | RR 0.37 (0.15-0.89) | |  | | Unstabilised infants | | RR 0.57 (0.33-1.00) |  |  |
|  |  |  |  |  | | Hypothermia at discharge or 40-41 weeks’ corrected gestational age | | RR 0.23 (0.10-0.55) | |  | | LMIC | | RR 0.58 (0.37-0.90) |  |  |
|  |  |  |  |  | | Mild/moderate infection or illness at latest f/u | | RR 1.28 (0.87-1.88) | |  | | Intermittent trials, initiated after 10 days post-birth, high-income countries, KMC in stabilised infants | | No beneficial effect |  |  |
|  |  |  |  |  | | Diarrhoea at 6 months f/u | | RR 0.65 (0.35-1.20) | |  | |  | |  |  |  |
|  |  |  |  |  | | Readmission to hospital | | RR 0.60 (0.34-1.06) | |  | |  | |  |  |  |
|  |  |  |  | Infant growth | | Weight gain per day | | RR MD 3.9 (1.9-5.8)g | |  | |  | |  |  |  |
|  |  |  |  |  |  |  |  |  |  |  |  |  |  |  |  |  |
|  |  |  |  |  |  |  |  | KMC gained more weight | |  |  |  |  |  |  |  |
|  |  |  |  |  | | Length | | RR MD 0.29 (0.27-0.31) | |  | |  | |  |  |  |
|  |  |  |  |  | | Head circumference | | RR MD 0.18 (0.09-0.27) | |  | |  | |  |  |  |
|  |  |  |  | Length of stay in hospital | |  | | 2.4 (0.7-4.1), days | |  | |  | |  |  |  |
| Early skin-to-skin contact for mothers and their healthy newborn infants (Review) | Moore, E 60 | 2012 | To assess the effects of early SSC on breastfeeding, physiological adaptation, and behaviour in healthy mother-newborn dyads | Neonatal intensive care unit admissions | |  | | No significant differences between groups | |  | |  | |  |  |  |
|  |  |  |  | Infant body weight change | | Day 14 post birth | | MD -8.00 (-175.60-156.61) | |  | |  | |  |  |  |
|  |  |  |  |  |  |  |  |  |  |  |  |  |  |  |  |  |
|  |  |  |  |  |  |  |  | No statistical difference | |  |  |  |  |  |  |  |
| Vitamin A supplementation for preventing morbidity and | Imdad, A **69.** | 2010 | To evaluate the effect of vitamin A supplementation (VAS) for preventing morbidity and mortality in children aged 6 months to 5 | Vitamin A | | Diarrhoea Incidence | | Decrease in diarrhoea | | All-cause mortality | | RR = 0.76 (95% CI 0.69 to 0.83), though there was moderate | |  |  |  |
| mortality in children from 6 months to 5 years of age (Review) |  |  | years |  |  |  |  | incidence (RR = 0.85 (95% CI 0.82 to 0.87)), though statistical | |  |  | heterogeneity (Chi² = 29.10, df = 15 (P = 0.02); I² = 48%) | |  |  |  |
|  |  |  |  |  |  |  |  | heterogeneity was substantial and highly significant (Chi² | |  |  |  | |  |  |  |
|  |  |  |  |  |  |  |  | = 218.62, df = 11 (P < 0.00001); I² = 95%), | |  |  | The effect during the first year of life was similar (RR = 0.82 (95% | |  |  |  |
|  |  |  |  |  |  |  |  |  | |  |  | CI 0.74 to 0.91)), but the statistical heterogeneity was substantial | |  |  |  |
|  |  |  |  |  |  |  |  |  | |  |  | (Chi² = 33.85, df = 11 (P = 0.0004); I² = 67%) | |  |  |  |
|  |  |  |  |  | | Diarrhoea prevalence | | (RR = 1.08 | | mortality between 13 and 60months | | (RR = 0.75 (95% CI 0.64 to 0.88)) with moderate and | |  |  |  |
|  |  |  |  |  |  |  |  | (95% CI 1.05 to 1.12)) though statistical heterogeneity was substantial | |  |  | significant statistical heterogeneity (Chi² = 9.29, df = 4 (P = 0.05); | |  |  |  |
|  |  |  |  |  |  |  |  | and highly significant (Chi² = 15.76, df = 2 (P = 0.0004); | |  |  | I² = 57%). | |  |  |  |
|  |  |  |  |  |  |  |  | I² = 87%). | |  |  |  | |  |  |  |
|  |  |  |  |  | | Measles incidence | | (RR = 0.50 (95% CI 0.37 to 0.67)) with no important | | Diarrhoea mortality | | (RR = 0.72 (95% CI 0.57 to 0.91)) with no important | |  |  |  |
|  |  |  |  |  |  |  |  | heterogeneity (Chi² = 0.55, df = 5 (P = 0.99); I² = 0%), | |  |  | heterogeneity (Chi² = 6.12, df = 6 (P = 0.41); I² = 2%), | |  |  |  |
|  |  |  |  |  |  |  |  |  |  |  |  |  |  |  |  |  |
|  |  |  |  |  | | Malaria prevalence | | Not statistically | | Measles mortality | | Not statistically significant | |  |  |  |
|  |  |  |  |  |  |  |  | significant (RR = 0.72 (0.42 to 1.23)) and there was no | |  |  | (RR = 0.80 (95% CI 0.51 to 1.24)). There was no important | |  |  |  |
|  |  |  |  |  |  |  |  | important heterogeneity (Chi² = 0.03, df = 1 (P = 0.87); I² = 0%). | |  |  | heterogeneity (Chi² = 0.40, df = 4 (P = 0.98); I² = 0%), | |  |  |  |
|  |  |  |  |  |  |  |  |  |  |  |  |  |  |  |  |  |
|  |  |  |  |  | | LRTI incidence | | No combined effect | | Meningitis mortality | | Not statistically significant (RR = 0.57 (95%CI 0.17 to 1.88). | |  |  |  |
|  |  |  |  |  |  |  |  | (RR = 1.14 (0.95 to 1.37)) with no important | |  |  | There was no important heterogeneity (Chi² = 0.75, df = 2 (P = | |  |  |  |
|  |  |  |  |  |  |  |  | heterogeneity (Chi² = 7.66, df = 6 (P = 0.26); I² = 22%). | |  |  | 0.69); I² = 0%). | |  |  |  |
|  |  |  |  |  |  |  |  |  |  |  |  |  |  |  |  |  |
|  |  |  |  |  | | LRT | | inconclusive | | LRTI mortality | | not statistically significant (RR = 0.78 (95%CI 0.54 | |  |  |  |
|  |  |  |  |  |  | prevalence | | but suggests benefit (RR = 0.46 (95% CI 0.21 to 1.03)). | |  |  | to 1.14)). There was no important heterogeneity (Chi² = 7, df = | |  |  |  |
|  |  |  |  |  |  |  | |  | |  |  | 6 (P = 0.32); I² = 14%). | |  |  |  |
|  |  |  |  |  |  |  |  |  |  |  |  |  |  |  |  |  |
|  |  |  |  |  | | Bitot’s spots prevalence | | RR = 0.45 | |  | |  | |  |  |  |
|  |  |  |  |  |  |  |  | (95% CI 0.33 to 0.61with substantial and significant heterogeneity | |  |  |  |  |  |  |  |
|  |  |  |  |  |  |  |  | (Chi² = 8.25, df = 3 (P = 0.04); I² = 64%). | |  |  |  |  |  |  |  |
|  |  |  |  |  |  |  |  |  |  |  |  |  |  |  |  |  |
|  |  |  |  |  | | Night blindness prevalence | | RR = 0.32 (95% CI 0.21 to 0.50 | |  | |  | |  |  |  |
|  |  |  |  |  |  |  |  | with no heterogeneity (Chi² = 0.19, df = 1 (P = 0.66); I² = 0%). | |  |  |  |  |  |  |  |
|  |  |  |  |  | | xerophthalmia incidence | | No combined effect on (RR = 0.85 (95% | |  | |  | |  |  |  |
|  |  |  |  |  |  |  |  | CI 0.70 to 1.03), though statistical heterogeneity was substantial | |  |  |  |  |  |  |  |
|  |  |  |  |  |  |  |  | and significant (Chi² = 2.69, df = 1 (P = 0.10); I² = 63%). | |  |  |  |  |  |  |  |
|  |  |  |  |  |  |  |  |  |  |  |  |  |  |  |  |  |
|  |  |  |  |  |  |  |  |  |  |  |  |  |  |  |  |  |
|  |  |  |  |  | | xerophthalmia prevalence | | RR = 0.31 (95% CI 0.22 to 0.45)) | |  | |  | |  |  |  |
|  |  |  |  |  |  |  |  | with no statistical heterogeneity (Chi² = 0.22, df = 1 (P = 0.64); | |  |  |  |  |  |  |  |
|  |  |  |  |  |  |  |  | I² = 0%). | |  |  |  |  |  |  |  |
|  |  |  |  |  | | number of VAD | | RR = 0.71 (95% CI 0.65 to 0.78)); however, statistical | |  | |  | |  |  |  |
|  |  |  |  |  |  | children | | heterogeneity was substantial and significant (Chi² = 13.58, df = | |  |  |  |  |  |  |  |
|  |  |  |  |  |  |  | | 3 (P = 0.004); I² = 78%). | |  |  |  |  |  |  |  |
|  |  |  |  |  | | Vitamin A serum levels | | Higher in the Vitamin | |  | |  | |  |  |  |
|  |  |  |  |  |  |  |  | A group (SMD = 0.31 (95% CI 0.26 to 0.36)), however statistical | |  |  |  |  |  |  |  |
|  |  |  |  |  |  |  |  | heterogeneity was substantial and significant (Chi² = 270.23, df = | |  |  |  |  |  |  |  |
|  |  |  |  |  |  |  |  | 13 (P < 0.00001); I² = 95%). | |  |  |  |  |  |  |  |
|  |  |  |  |  |  |  |  |  |  |  |  |  |  |  |  |  |
| Neonatal vitamin A supplementation for the prevention of mortality and morbidity in term neonates in developing countries (Review) | Haider, B 62 | 2011 | To evaluate the role of Vitamin A supplementation in term neonates in developing countries with respect to the prevention of mortality and morbidity | Neonatal Vit A supplementation versus placebo | |  | |  | | Neonatal Vit A supplementation versus placebo | |  | |  |  |  |
|  |  |  |  | Cause-specific infant mortality at 6 months of age: diarrhoea and acute respiratory infections | |  | |  | | All-cause mortality at 6 months of age | | Term infants | | RR 0.82 (0.68-0.99) |  |  |
|  |  |  |  |  |  |  |  |  |  |  |  |  |  |  |  |  |
|  |  |  |  |  |  |  |  |  |  |  |  |  |  | (Supplementation lowers risk) |  |  |
|  |  |  |  | All infants (1 study) | | Diarrhoea | | RR 0.20 (0.02-1.68) | | All-cause mortality at 6 months of age | | All infants | | RR 0.86 (0.77-0.97) |  |  |
|  |  |  |  |  |  |  |  |  |  |  |  |  |  |  |  |  |
|  |  |  |  |  |  | Respiratory | | RR 0.66 (0.11-3.91) | | All-cause infant mortality at 6 months of age (per years of follow-up) | | Term neonates | | RR 0.91 (0.73-1.13) |  |  |
|  |  |  |  |  |  |  |  |  |  |  |  |  |  | (No sign effect of rate of death from any cause at 6 months of age in those that received Vit A compared to control. |  |  |
|  |  |  |  | All infants (1 study), per years of f/u | | Diarrhoea | | RR 0.67 (0.32-1.39) | |  | | All infants | | RR 0.91 (0.77-1.06) |  |  |
|  |  |  |  |  | | Acute Respiratory | | RR 1.00 (0.56-1.79) | |  | | 6 months | | RR 0.89 (0.75-1.05) |  |  |
|  |  |  |  |  | |  | |  | | All-cause infant mortality at 12 months of age | | Term neonates | | RR 0.95 (0.72-1.26) |  |  |
| **Post Natal** |  |  |  |  | |  | |  | |  | |  | |  |  |  |
| Antidepressant prevention of postnatal depression (Review) | Howard, L **65** | 2009 | To evaluate the effectiveness of different antidepressant drugs in addition to standard clinical care in the prevention of postnatal depression | Nortryptiline versus placebo | | Recurrence of postpartum major depressive disorder | | No difference | |  | |  | |  |  |  |
|  |  |  |  |  | | Rate of recurrence | | No difference | |  | |  | |  |  |  |
|  |  |  |  |  | | Time to recurrence | | No difference | |  | |  | |  |  |  |
|  |  |  |  | Sertraline versus placebo | | Recurrence of postpartum major depressive disorder | | Sertraline was more effective | |  | |  | |  |  |  |
|  |  |  |  |  | | Time to recurrence | | Longer in the sertraline-treated women compared with placebo-treated women (p=0.012) | |  | |  | |  |  |  |
| Optimisation of antiretroviral therapy in HIV-infected children under 3 years of age (Review) | Penazzato, M **66** | 2014 | To evaluate when to start ART in young children (less than 3 years); what ART to start with, comparing first-line non-nucleoside reverse transcriptase inhibitor (NNRTI) and; whether alternative strategies should be used to optimize antiretroviral treatment in this population protease inhibitor (PI)-based regimens | Timing of treatment: use of early compared to deferred ART | | Immunological response | | PEHSS study median CD4 cell count was not significantly | | Infant death | | Pooled HR | | Mortality + Disease progression |  |  |
|  |  |  |  |  |  |  |  | different between groups 12 months after ART initiation (immediate | |  |  | for time to death two trials = 0.36 (95%CI 0.18-0.74) | |  |  |  |
|  |  |  |  |  |  |  |  | group 33% vs deferred group 32%; P=0.70) | |  |  |  | |  |  |  |
|  |  |  |  |  | |  | | CHER study -mean changes from baseline | |  | |  | |  |  |  |
|  |  |  |  |  |  |  |  | in the CD4 percentage were reported and absolute difference between | |  |  |  |  |  |  |  |
|  |  |  |  |  |  |  |  | the early-therapy group and the deferred-therapy group was | |  |  |  |  |  |  |  |
|  |  |  |  |  |  |  |  | 12.3%(p<0.001) at 12weeks, 11.5%(p<0.001) at 32weeks, 9.3% | |  |  |  |  |  |  |  |
|  |  |  |  |  |  |  |  | (p<0.001) at 24 weeks and 6.7% by week 40 | |  |  |  |  |  |  |  |
|  |  |  |  | Choice of treatment: use of NNRTI- versus PI-based regimens, | | Virological failure (defined as a confirmed plasma | | HR = 1.84 (95%CI | |  | |  | | Treatment failure (a composite of virological failure or discontinuation |  |  |
|  |  |  |  | in combination with any NRTI backbone | | HIV-1 RNA level of less than 1 log10 copies per millilitre below | | 1.29-2.63) times higher for children starting ART with a NVPbased | |  |  |  |  | of the study drugs for any reason, including death) |  |  |
|  |  |  |  |  | | the study entry level at 12 to 24 weeks after the initiation of | | regimen compared to those starting with a LPV/r-based regimen | |  |  |  |  |  |  |  |
|  |  |  |  |  | | treatment or a confirmed plasma HIV-1 RNA level of more than | | (p=0.0008) | |  |  |  |  |  |  |  |
|  |  |  |  |  | | 400 copies per millilitre at 24 weeks) | |  | |  |  |  |  |  |  |  |
|  |  |  |  |  | |  | |  | |  | |  | | Virological failure (defined as a confirmed plasma |  |  |
|  |  |  |  |  |  |  |  |  |  |  |  |  |  | HIV-1 RNA level of less than 1 log10 copies per millilitre below |  |  |
|  |  |  |  |  |  |  |  |  |  |  |  |  |  | the study entry level at 12 to 24 weeks after the initiation of |  |  |
|  |  |  |  |  |  |  |  |  |  |  |  |  |  | treatment or a confirmed plasma HIV-1 RNA level of more than |  |  |
|  |  |  |  |  |  |  |  |  |  |  |  |  |  | 400 copies per millilitre at 24 weeks) |  |  |
|  |  |  |  | Substitution of LPV/r with NVP following initiation with PI-based regimens. | | NEVEREST trial - risk of having at least one VL greater than 50 copies/ mL | | Lower in children substituting NVP for LPV/r after a median of 9 months on LPV/r-based regimen (having achieved | |  | |  | |  |  |  |
|  |  |  |  |  |  |  |  | virological suppression) compared to those remaining on a LVP/rbased regimen (HR=0.62, 95%CI 0.41-0.92, p=0.02) | |  |  |  |  |  |  |  |
|  |  |  |  |  | | Confirmed virological failure (>1000 | | Higher among children substituting LPV/r with | |  | |  | |  |  |  |
|  |  |  |  |  |  | copies/mL) | | NVP as compared to those remaining on LPV/r (HR=10.19, 95% | |  |  |  |  |  |  |  |
|  |  |  |  |  |  |  | | CI 2.36, 43.94, p=0.002) | |  |  |  |  |  |  |  |
|  |  |  |  |  | | CD4% increase | | Lower | |  | |  | |  |  |  |
|  |  |  |  |  |  |  |  | in the control (LPV/r) group compared to the NVP group (RR= | |  |  |  |  |  |  |  |
|  |  |  |  |  |  |  |  | 0.22,95% CI 0.07-0.74, p=0.01) | |  |  |  |  |  |  |  |
|  |  |  |  |  | | Weight-for-age Z | | Similar on average, but fewer children in the NVP | |  | |  | |  |  |  |
|  |  |  |  |  |  | scores | | group experienced a decline in weight-for-age (RR=0.32, 95% CI | |  |  |  |  |  |  |  |
|  |  |  |  |  |  |  | | 0.11-0.94, p=0.04 | |  |  |  |  |  |  |  |
|  |  |  |  |  | | Grade 3 or 4 elevation in ALT | | More common in the NVP group but events were rare | |  | |  | |  |  |  |
|  |  |  |  |  |  | levels | | and no association was found with study arm (RR=1.80, 95% CI | |  |  |  |  |  |  |  |
|  |  |  |  |  |  |  | | 0.55-5.97, p=0.33 | |  |  |  |  |  |  |  |
|  |  |  |  |  | | Grade 3 or 4 neutropenia | | Rare and similar across arms (RR=1.72, 95% CI 0.42-6.99, | |  | |  | |  |  |  |
|  |  |  |  |  |  |  |  | p=0.45) | |  |  |  |  |  |  |  |
|  |  |  |  | Induction-maintenance treatment: Initiating ART with more | | The primary endpoint (mean change in CD4 percentage from baseline) | | No significant difference at 72 weeks (mean difference 0.70 [95%CI -0.51, 1.91] p= 0.33) or 144 weeks (mean difference -0.20 [95%CI -1.48, | | Mortality | | No difference between groups (HR 0.71 [95%CI 0.16, 3.21] p=0.66) | | Mortality and disease progression |  |  |
|  |  |  |  | than 3 antiretroviral drugs for an induction period, then moving | |  |  | 1.08] p= 0.69) | |  |  |  |  |  |  |  |
|  |  |  |  | to maintenance treatment with a standard 3-drug regimen | |  |  |  | |  |  |  |  |  |  |  |
|  |  |  |  |  | |  | | 36 weeks (not the primary | |  | |  | |  |  |  |
|  |  |  |  |  |  |  |  | endpoint, but the end of the induction period in Arms B and C) | |  |  |  |  |  |  |  |
|  |  |  |  |  |  |  |  | there was a significantly greater CD4 increase in the 4-drug, compared | |  |  |  |  |  |  |  |
|  |  |  |  |  |  |  |  | to 3-drug, arms (mean difference 1.70 [95%CI 0.61, 2.79] | |  |  |  |  |  |  |  |
|  |  |  |  |  |  |  |  | p=0.002) | |  |  |  |  |  |  |  |
|  |  |  |  |  | | At 24 weeks virological response was better | | (OR 1.99 [95%CI 1.09, 3.62] p=0.02, | |  | |  | |  |  |  |
|  |  |  |  |  |  | in those children who received an induction-maintenance regimen | |  |  |  |  |  |  |  |  |  |
|  |  |  |  |  |  | (Arms B and C) than in those who received a standard 3-drug | |  |  |  |  |  |  |  |  |  |
|  |  |  |  |  |  | regimen (Arm A) | |  |  |  |  |  |  |  |  |  |
|  |  |  |  |  | | At 48 weeks the effect is not maintained | | (OR 0.96 | |  | |  | |  |  |  |
|  |  |  |  |  |  |  |  | [95%CI 0.50, 1.83] p=0.90) and 144 weeks (OR 1.03 [95%CI | |  |  |  |  |  |  |  |
|  |  |  |  |  |  |  |  | 0.51, 2.07] p=0.94 | |  |  |  |  |  |  |  |
|  |  |  |  | Planned treatment interruption | | OPH-03 trial - | | 0.05 vs -0.08 (MD= -0.13, 95% CI -0.31 to 0.05, p=0.1) | |  | |  | |  |  |  |
|  |  |  |  |  |  | 6-month mean change in weight-for-age Z-score | |  |  |  |  |  |  |  |  |  |
|  |  |  |  |  | | Height-for-age Z-score | | 0.06 vs -0.04 (MD= - 0.10, 95% CI -0.33 to 0.13, p= 0.39) | |  | |  | |  |  |  |
|  |  |  |  |  | | Weight for- height Z-score | | 0.07 vs -0.04 (MD= -0.11, 95% CI -0.32 to 0.10, p= 0.30) | |  | |  | |  |  |  |
|  |  |  |  |  | | One serious adverse event was observed in each arm | | (OR=1.00, 95% CI 0.006 to 17.12 p=1.00) | |  | |  | |  |  |  |
| **Across the first 1000 days categories** | | | | | | | | | | | | | | | | |
| Lay health workers in primary and community health care for | Lewin, S **71** | 2010 | To assess the effects of LHW interventions in primary and community health care on maternal and child health and the management | LHW intervention to reduce mortality and morbidity in children <5yrs with usual care | |  | | RR 0.86 (0.75-0.99) low quality | | Mortality in children <5yrs | |  | | RR 0.75 (0.55-1.03) low quality |  |  |
| maternal and child health and the management of infectious |  |  | of infectious diseases. |  |  |  |  |  |  |  |  |  |  |  |  |  |
| diseases (Review) |  |  |  | Child morbidity (fewer acute respiratory infection ) <5yrs | |  |  |  |  | Neonatal mortality | |  | | RR 0.76 (0.57-1.02) low quality |  |  |
|  |  |  |  | LHW interventions to promote breastfeeding with usual care | |  | |  | |  | |  | |  |  |  |
|  |  |  |  | LHW interventions to provide support to mothers of sick children with usual care | | Not possible to combine in meta-analysis | | | | Evidence of small benefit for child health and development | | | | |  |  |
|  |  |  |  | Rest of outcomes – no change or not appropriate | | | | | | | | | | |  |  |
| Community-based intervention packages for reducing | Lassi, Z **72.** | 2010 | To assess the effectiveness of community-based intervention packages in reducing maternal and neonatal morbidity and mortality; and |  |  | |  | | Maternal mortality | |  | | RR 0.77 (0.59-1.02) no impact | |  |  |
| maternal and neonatal morbidity and mortality and |  |  | improving neonatal outcomes. |  |  |  |  |  | Community –based intervention packages | | Building support groups | | RR 0.84 (0.36-1.95) | |  |  |
| improving neonatal outcomes (Review) |  |  |  |  |  |  |  |  |  | |  |  |  |  |  |  |
|  |  |  |  |  |  |  |  |  |  | | Mobilised community and made home visits during antenatal and post-natal periods | | RR 0.72 (0.49-1.05) | |  |  |
|  |  |  |  |  |  |  |  |  |  |  |  |  |  |  |  |  |
|  |  |  |  |  |  |  |  |  |  | | Training of TBAs who then made home visits during antenatal period and during delivery | | RR 0.70 | |  |  |
|  |  |  |  |  |  |  |  |  |  | |  |  | (0.51-0.96) Significant impact | |  |  |
|  |  |  |  |  |  | |  | | Neonatal | | -Build support and advocacy groups | | RR 0.76 (0.68-0.84) | |  |  |
|  |  |  |  |  |  |  |  |  | Community-based intervention packages | | -Home visitations along with community mobilisation | | RR 0.79 (0.68-0.92) | |  |  |
| Systematic review of effect of community-level interventions to | Kidney, E **73** | 2009 | To provide a systematic review of the effectiveness of community level |  |  | |  | | Maternal | |  | | OR 0.62 (0.39-0.98) decrease in mortality | |  |  |
| reduce maternal mortality |  |  | Interventions to reduce maternal mortality. |  |  |  |  |  | 2 Trials Peri-natal practices | |  |  |  |  |  |  |
|  |  |  |  |  |  | |  | | Minimal goal oriented versus usual antenatal care | |  | | OR 1.09 (0.53-2.25) No difference. Cohort studies were of low quality and did not contribute further evidence | |  |  |
|  |  |  |  |  |  | |  | | Cohort studies were of low quality and did not contribute further evidence | | | | | |  |  |
| Effect of washing hands with soap on diarrhoea risk in the community: a systematic review | Curtis, V **75.** | 2003 | To determine the effects of washing hands with soap on diarrhoea risk and estimated potential reductions in diarrhoea mortality. | Diarrhoea |  | | RR 1.88 (1.31-2.68) | |  | |  | |  | |  |  |
|  |  |  |  |  |  |  |  |  |  |  |  |  |  |  |  |  |
|  |  |  |  |  |  |  | Reduction | |  |  |  |  |  |  |  |  |
|  |  |  |  |  |  | |  | |  | |  | |  | |  |  |
| Interventions to improve water quality for preventing | Clasen, T **76.** | 2007 | To assess the effectiveness of interventions to | Water source versus household interventions | Source based <5yrs | | RR 0.93 (0.82-1.05) | |  | |  | |  | |  |  |
| diarrhoea: systematic review and meta-analysis |  |  | improve the microbial quality of drinking water for | Source based |  |  |  |  |  |  |  |  |  |  |  |  |
|  |  |  | preventing diarrhoea | Household based | Household based diarrhoea | | RR 0.76 (0.66-0.88) | |  |  |  |  |  |  |  |  |
|  |  |  |  |  | <5yrs | |  |  |  |  |  |  |  |  |  |  |
|  |  |  |  | Types of household interventions | <5yrs | | Decrease | |  | |  | |  | |  |  |
|  |  |  |  | Household chlorination |  |  |  |  |  |  |  |  |  |  |  |  |
|  |  |  |  | Household filters |  | | Decrease | |  | |  | |  | |  |  |
|  |  |  |  | Solar disinfection |  | | Decrease | |  | |  | |  | |  |  |
|  |  |  |  | Household based flocculants disinfection |  | | No difference in diarrhoea episodes | |  | |  | |  | |  |  |

**Appendix 5 –** Table with a detailed summary of the interventions identified

| **Field** | **Study Design** | **Author** | **Title** | **Year** | **Journal** | **Number of studies** | **No of participants** | **Objectives** | **Target population** | **Intervention** | **Outcome** |
| --- | --- | --- | --- | --- | --- | --- | --- | --- | --- | --- | --- |
| **Pre-pregnancy** | | | | | | | | | | | |
| Promotion of birth spacing of between 18 and 59 months | Meta-analysis | Conde-Agudelo, A 27 | Birth spacing and risk of adverse perinatal outcomes: a meta-analysis. | 2006 | JAMA | 67 studies (52 cohort / cross sectional + 15 case control) | 11 091 659 pregnancies | To examine the association between birth spacing and relative risk of adverse perinatal outcomes | pregnant women | Inter-pregnancy interval is used as a measure of birth spacing. IPI is defined as time elapsed between the woman’s last delivery and the conception of the next pregnancy | Inter-pregnancy intervals (IPIs) of <6 months as compared to IPIs of 18–23 months are associated with a statistically significant higher risk of low birth weight (OR 1.61; 95% CI: 1.39 to 1.86) preterm birth (OR 1.40; 95% CI: 1.24 to 1.58) and SGA infants (OR 1.26; 95% CI: 1.18 to 1.33). For IPIs > 60 months, Pre-term birth OR 1.20; 95% CI:1.17-1.24), LBW OR 1.43; 95% CI: 1.27-1.62), SGA OR 1.29; 95% CI: 1.20-1.39). Increase in risk for each month that the inter-pregnancy interval was shortened from 18 months, increase (95% CI) Pre-term 1.92; 95 %CI: 1.80-3.04) LBW OR 3.25; 95% CI: 3.09-3.41) and SGA OR 1.52; 95% CI:1.40-1.64). IPIs shorter than 18 months and longer than 59 months are significantly associated with increased risk of adverse perinatal outcomes. |
| **Antenatal** | | | | | | | | | | | |
| Promotion of a minimum of > or = 4 ANC visits | Systematic review | Dowswell, T 28 | Alternative versus standard packages of antenatal care for low-risk pregnancy. | 2010 | Cochrane | 7 trials  4 – HIC (1 England, 3 USA) 3 – LMIC (1 WHO, 2 – Zimbabwe) | More 60 000 women | To assess whether similar clinical outcomes can be achieved with reducing the number of ANC visits for low-risk women and comparing them to the standard schedule of visits of antenatal care | Pregnant women attending antenatal care at low risk of developing complications during labour | Intervention –provision of a schedule of reduced number of ANC visits:  • England – reduced to 7 visits • Two USA – reduced to 8 visits • One USA – reduced to 9 visits • WHO – reduced to 4 visits • Zimbabwe – reduced to 5 visits • Zimbabwe – reduced to 6 visits | No difference in maternal mortality between the two groups (RR 1.13; 95% CI 0.50 to 2.57)  Both groups had similar levels of HPT (inc pre-eclampsia) (RR 0.95; 95% CI 0.80 to 1.12) Perinatal Mortality increased for those in the reduced groups (RR 1.14; 95% CI 1.00-1.31). In LMIC only (RR 1.15; 95% CI 1.01 to 1.32). No clear difference in preterm births. Maternal Morbidity – no clear differences in vaginal bleeding, postpartum haemorrhage, similar numbers of women underwent C-section, induction of labour. Neonatal morbidity – no difference in LBW, no difference in number admitted to ICU (HIC), favourable in LIC – (RR 0.86; 95% CI 0.74 – 0.99) |
| Multiple Micronutrient, Balanced Protein Energy and Folic Acid supplementation | Systematic Review | Haider, B 30 | Effect of multiple micronutrient supplementation during pregnancy on maternal and birth outcomes | 2011 | BMC Public Health | 17 studies (14 trials): 8 RCTs and 6 were cluster RCTs | Not mentioned | (1) To estimate the effectiveness of peri-conecptional folic acid supplementation in reducing neural tube defects (NTDs) related to stillbirths. (2) To estimate the effectiveness of balanced protein energy supplementation during pregnancy in reducing all-cause stillbirths. (3) To estimate the effectiveness of multiple micronutrient supplementation during pregnancy in reducing all-cause stillbirths. (this summary only includes the balanced protein energy and multiple micronutrients) | Pregnant women | Twelve studies used multiple micronutrient supplement formula called UNIMMAP which consisted of 30 mg iron, 400 μg folic acid, 15 mg zinc 2 mg copper, 65 μg selenium, 800μg RE vitamin A, 1.4 mg vitamin B1, 1.4 mg vitamin B2, 18 mg niacin, 1.9mg vitamin B6, 2.6 μg vitamin B12, 70 mg vitamin C, 5μg vitamin D, 10 mg vitamin E and 150 μg iodine.  The minimum number of micronutrients in the studies was nine. The UNIMAPP formulation containing 30 mg iron and 0.4 mg folic acid was provided in nine trials. The intervention group in the one study received multiple micronutrient tablets containing 0.8 mg of folic acid along with separate iron and folic acid supplementation. Another study showed the multi-micronutrient tablet contained 10 mg of ferrous fumarate and 0.15 mg of folic acid along with supplemental iron-folate. A third study included 60mg iron and 0.4 mg folic acid. Fourth study provided 62.4 mg iron and 0.215 mg folic acid in the multi-micronutrient formulation. | Multiple micronutrient supplementation had no significant effect on maternal anaemia in the third trimester compared to iron-folate based on data from 4 RCTs (RR = 1.03; 95% CI: 0.87 – 1.22). There was a statistically significant reduction in the risk of small for gestational age infants with multiple micronutrient supplements (RR = 0.91; 95% CI: 0.86 – 0.96) based on fourteen studies. There was no statistically significant increase in the risk of neonatal mortality based on nine RCTs. (RR = 1.05; 95% CI: 0.92 – 1.19) There was an increased risk of neonatal mortality in studies with majority of births at home (RR = 1.47, 95% CI 1.13-1.92) |
| Multiple micronutrients | Meta-analyses | Imdad, A 31 | The effect of folic acid, protein energy and multiple micronutrient supplements in pregnancy on stillbirths. | 2011 | BMC Public Health | 18 studies (7 evaluated the effect of peri-conceptional folic acid supplementation and 11 studies assessed fortification of food with folic acid). | 2186 pregnancies | (1) To estimate the effectiveness of peri-conecptional folic acid supplementation in reducing neural tube defects (NTDs) related to stillbirths. (2) To estimate the effectiveness of balanced protein energy supplementation during pregnancy in reducing all-cause stillbirths. (3) To estimate the effectiveness of multiple micronutrient supplementation during pregnancy in reducing all-cause stillbirths. (this summary only includes the balanced protein energy and multiple micronutrients) | Women in general and some studies focussed on pregnant women | peri-conceptional folic acid supplementation | Pooled results of three RCTs for prevention of recurrent NTDs showed a reduction of 70 % in recurrence of NTDs (RR 0.30; 95 % CI 0.14-0.65). Effect of folic acid fortification on prevention of neural tube defects. Meta-analysis based on results of 11 before and after studies. (RR 0.59; 95% CI 0.52- 0.68). |
|
|
| Balanced Protein Energy during pregnancy | Systematic Review | Kramer, S 32 | Energy and protein intake in pregnancy. | 2003 | Cochrane | Five trials of nutritional advice, involving 1134 women, were included. 13 trials (4665 women),  Two trials involving 1076 women,  Three trials, involving 966 women, Three trials, involving 384 women | 8225 | To assess the effects of dietary advice, supplementation, or restriction on gestational weight gain, pre-eclampsia, and/or pregnancy outcomes. | Pregnant women. For the assessment of dietary restriction, pregnant women with either high pregnancy weight or high gestational weight gain. | 1.Nutritional advice to increase energy and protein intakes 2. Balanced energy/protein supplementation 3. High-protein supplementation 4. Isocaloric protein supplementation 5. Energy/protein restriction in women with overweight or high weight gain | 1.Nutritional advice to increase energy and protein intakes No consistent benefit on pregnancy outcomes  2. Balanced energy/protein supplementation No statistically significant differences were found in birth length and birth head circumference. However, the incidence of small-for-gestational-age (SGA) birth was reduced substantially (RR 0.68, 95% CI 0.56 to 0.84). Reductions in stillbirth (RR 0.55, 95% CI 0.31 to 0.97) and neonatal death (RR 0.62, 95% 0.37 to 1.05) are based on only four trials  3. High-protein supplementation Nothing significant  4. Isocaloric protein supplementation In one trial there was an increased risk of small for- gestational-age (SGA) birth (RR 1.35, 95% CI 1.12 to 1.61)  5. Energy/protein restriction in women with overweight or high weight gain Significant reduction in weekly maternal weight gain, although the magnitude of the reduction was much larger in the one trial (random effects WMD-254.81 (95% CI -436.56 to -73.06) g/week). No effect on pregnancy-induced hypertension or preeclampsia. |
| Iron and Folic Acid Supplementation | SR | Pena-Rosas, JP 33 | Daily oral iron supplementation during pregnancy. | 2012 | Cochrane | 60 RCTs with 47 combined into a meta-analyses | 27,402 women | To assess the effects of daily oral iron supplements for pregnant women, either alone or in conjunction with folic acid, or with other vitamins and minerals | Pregnant women of any gestational age and parity. | Intervention was daily supplements. In one trial women received less than 400 μg (0.4 mg) of folic acid per day. In another study women consumed 800 μg (0.8 mg) folic acid plus multiple micronutrients versus multiple micronutrients, another study 2000 μg (2.0 mg) per day folic acid supplementation with a placebo group and the last two studies 4000 μg (4.0 mg) per day folic acid plus multiple micronutrients versus multiple micronutrients in one study. The other study had folic acid 4000 μg (4.0 mg) four comparison groups: one with folic acid with iron and calcium, one with folic acid plus iron, calcium and multiple micronutrients, another with iron, calcium and multiple micronutrients without folic acid and a control group that only received iron and calcium with no multiple micronutrients nor folic acid  In all trials women started supplementation before pregnancy and discontinued it after 12 weeks of pregnancy. | Overall, women taking iron supplements were less likely to have low birthweight newborns (below 2500 g) compared with controls (8.4% versus 10.2%, average risk ratio (RR) 0.81; 95% confidence interval (CI) 0.68 to 0.97, 11 trials, 8480 women) and mean birthweight was 30.81 g greater for those infants whose mothers received iron during pregnancy (average mean difference (MD) 30.81; 95% CI 5.94 to 55.68, 14 trials, 9385 women). Preventive iron supplementation reduced the risk of maternal anaemia at term by 70% (RR 0.30; 95% CI 0.19 to 0.46, 14 trials, 2199 women) and iron deficiency at term by 57% (RR 0.43; 95% CI 0.27 to 0.66, seven trials, 1256 women). |
| Detection and Treatment of maternal Syphillis | Systematic Review | Blencowe, H 34 | Lives saved tool supplement detection and treatment of syphilis in pregnancy to reduce syphilis related stillbirths and neonatal mortality. | 2011 | BMC Public Health | 24 observational studies | Not specified in article | To estimate the effect of detection and treatment of active syphilis in pregnancy with at least 2.4MU benzathine penicillin (or equivalent) on syphilis-related stillbirths and neonatal mortality. | The population of interest is pregnant women with active syphilis | intervention is the serologic detection of syphilis in pregnant women,. The comparison group is is pregnant women with active syphilis who do not receive at least 2.4 million units at least 28 days prior to delivery | Evidence for the effectiveness of detection and treatment of active syphilis in pregnancy in reducing stillbirths and perinatal mortality (8 observational studies) A random-effects meta-analysis of all eight studies produced an estimated 82% reduction in stillbirths for syphilis RR of 0.18 (95% CI 0.10 - 0.33).   Evidence for the effectiveness of detection and treatment of active syphilis in pregnancy in reducing neonatal mortality  Perinatal Mortality (stillbirth and early neonatal mortality):  2 cohort studies (both from SA)  A meta-analysis of these five studies results in an estimated RR of 0.20 (95% CI 0.13 – 0.32)   Evidence for the effectiveness of detection and treatment of active syphilis in pregnancy in reducing preterm births Seven studies gives a RR of 0.36 (95% CI. 0.27 – 0.47) for preterm birth with penicillin treatment  Evidence for the effectiveness of detection and treatment of active syphilis in pregnancy in reducing the incidence of congenital syphilis in live born infants 3 observational studies. RR=0.03 (0.02 - 0.07) |
| Calcium Supplementation to prevent hypertensive disorders | Systematic Review | Hofmeyr, GJ 35 | Calcium supplementation during pregnancy for preventing hypertensive disorders and related problems. | 2006 | Cochrane | 11 trials | 6894 women | To assess the effects of calcium supplementation during pregnancy on hypertensive disorders of pregnancy and related maternal and child adverse outcomes. | Pregnant women, regardless of the risk of hypertensive disorders of pregnancy. | Supplementation with calcium from at the latest 34 weeks of pregnancy; compared with placebo treatment. Intended supplementation with at least 1 g of calcium per day. | High blood pressure with or without proteinuria less high blood pressure with calcium supplementation (10 trials, 6634 women: (RR) 0.58, 95% (CI) 0.43 to 0.79), Greater effect amongst women at high risk of developing hypertension (four trials, 327 women: RR 0.47, 95% CI 0.22 to 0.97), and those with low baseline dietary calcium (five trials, 1582 women: RR 0.38, 95% CI 0.22 to 0.64).  Pre-eclampsia The overall effect was a reduction in the risk of preeclampsia (11 trials, 6894women: RR0.35, 95%CI 0.20 to 0.60). Pre-eclampsia reduction is significant for women at low risk (six trials, 6307 women: RR 0.49, 95% CI 0.28 to 0.87), Pre-eclampsia was considerably reduced in women at high risk of hypertension (five trials, 587 women: RR 0.22, 95% CI 0.12 to 0.42), and those with low baseline calcium intake (six trials, 1842 women: RR 0.29, 95% CI 0.16 to 0.54). Preterm delivery did appear to be reduced among women at high risk of developing hypertension (four trials, 568 women: RR 0.45, 95% CI 0.24 to 0.83). There were fewer babies with birthweight, less than 2500 g in women at high risk of hypertension (two trials, 449 women: RR 0.45, 95% CI 0.22 to 0.95), |
| Calcium Supplementation to prevent hypertensive disorders | Systematic Review | Imdad, A 36 | Role of calcium supplementation during pregnancy in reducing risk of developing gestational hypertensive disorders: a meta-analysis of studies from developing countries. | 2011 | BMC Public Health | 10 RCTs | 11405 | To evaluate preventive effect of calcium supplementation during pregnancy on gestational hypertensive disorders and related maternal and neonatal mortality in developing countries. | Pregnant women (all in developing countries, starting period of calcium supplementation in all the included studies was before 20-32 weeks of gestation and continued till delivery | The dose of calcium ranged from 0.5 g/day to 2 g/day. | MATERNAL OUTCOMES Maternal mortality I trial. (RR 0.17; 95% CI 0.03-0.76).  Eclampsia 1 trial. a relative risk of 0.68 (95 % CI 0.48-0.97)  Severe eclampsia A 30% reduction in calcium group compared to control, however the results were not statistically significant [(RR 0.70; 95% CI 0.46- 1.05). This was not statistically significant.  Pre-eclampsia 10 studies from developing world. A reduction of 59% [RR 0.41; 95 % CI 0.24-0.69]. The reduction was more marked in participants with a higher pre-pregnancy risk of developing gestational hypertensive disorders [RR 0.18, 95 % CI 0.07-0.42, random model] compared to that of low risk women [RR 0.51, 95 % CI 0.30-0.87].  Gestational hypertension 6 studies from developing countries. A significant reduction of 45 % in risk of development of gestational hypertension in women receiving calcium supplementation. [RR 0.55; 95 % CI 0.36-0.85]  NEONATAL OUTCOMES Neonatal mortality 1 trial. Significant reduction of 30 % in the intervention group compared to placebo (RR 0.70; 95 % CI 0.56-0.88).  Preterm birth 5 trials. A significant reduction of 12% (RR 0.88; 95% CI 0.78-0.99)  SGA 2 trials. A non-significant reduction in risk of small for gestational babies (RR 0.90; 95 % 0.59-1.38) (data not shown).  LBW No impact of calcium supplementation compared to control (RR 0.81 95% CI 0.58-1.12) |
|  |  |  |  |  |  |  |  |  |  |  |  |
| Antiplatelet agents for preventing pre-eclampsia | Systematic Review | Duley, L 37 | Antiplatelet agents for preventing pre-eclampsia and its complications. | 2007 | Cochrane | 59 trials involving | 37,560 women | To assess the effectiveness and safety of antiplatelet agents for women at risk of developing pre-eclampsia. | Pregnant women considered to be at risk of developing preeclampsia | (1) Antiplatelet agents versus placebo or no treatment for the primary prevention of pre-eclampsia and its complications (2) Antiplatelet agents versus placebo or no treatment for secondary prevention of pre-eclampsia and its complications in women with gestational hypertension | A. Antiplatelet agents versus placebo or no treatment for the primary prevention of pre-eclampsia and its complications overall difference in the risk of gestational hypertension statistically significant reduction in risk in one pre-specified subgroup of 838 high-risk women enrolled in 12 small trials that reported gestational hypertension ((RR 0.54, 95%CI 0.41 to 0.70) Overall there is a 17% reduction in the risk of proteinuric pre-eclampsia associated with the use of antiplatelet agents ((46 trials with 32,891 women, RR 0.83, 95% CI 0.77 to 0.89). In the 21 trials (26,984women) that evaluated 75mg/day or less of aspirin the RR is 0.88 (95%CI 0.81 to 0.95), in the 17 trials (3061 women) evaluating more than 75 mg/day of aspirin the RR is 0.64 (95% CI 0.51 to 0.80) and in the five trials of 506 women evaluating more than 75 mg/day of aspirin plus dipyridamole the RR is 0.30 (95% CI 0.15 to 0.60). (8%) Reduction in the risk of delivery before 37 completed weeks  Stillbirths ((RR 0.92, 95% CI 0.88 to 0.97 there is a 14% reduction in the risk of death in the antiplatelet group ((RR 0.86, 95%CI 0.76 to 0.98), 10% reduction in the risk of small-for-gestational age births ((RR 0.90, 95% CI 0.83 to 0.98)  B. Antiplatelet agents versus placebo or no treatment for secondary prevention of pre-eclampsia and its complications in women with gestational hypertension In five trials (1643 women), there was a 40% reduction in the relative risk of proteinuric pre-eclampsia (RR 0.60, 95% CI 0.45 to 0.78). In three trials (1451 women), there is a 13% reduction in the relative risk of preterm birth at less than 37 weeks (RR 0.87, 95% CI 0.75 to 0.99). One small trial (100 women) reported a statistically significant reduction in the relative risk of the baby being born with a low birthweight (RR 0.24, 95% CI 0.09 to 0.65). |
| Interpersonal psychotherapy to prevent and treat antenatal depression | Systematic review | Dennis, C 39 | Psychosocial and psychological interventions for treating ante-natal depression | 2007 | Cochrane | 1 RCT | 38 | The primary objective of this review was to assess the effects, on mothers and their families, of psychosocial and psychological interventions compared with usual antepartum care in the treatment of antenatal depression. | Pregnant women identified with antenatal depression (variously defined) | Any form of standard or usual care compared to a variety of non-pharmaceutical interventions - including psycho educational strategies, cognitive behavioural therapy, interpersonal psychotherapy, non-directive counselling, various supportive interactions, and tangible assistance - delivered via telephone, home or clinic visits, or individual or group sessions antenatally by a professional (nurse, midwife, childbirth educator, physician) or lay person (a specially trained woman from the community, a student). | Interpersonal psychotherapy, compared to a parenting education program, was associated with a reduction in the risk of depressive symptomatology immediately post-treatment using the Clinical Global Impression Scale (one trial, n = 38; relative risk (RR) 0.46, 95% confidence interval (CI) 0.26 to 0.83) and the Hamilton Rating Scale for Depression (one trial, n = 38; RR 0.82, 95% CI 0.65 to 1.03). |
| Prevention and management of HIV and prevention of mother to child transmission in pregnancy (PMTCT) | Systematic Review | Siegfried, N 40 | Antiretrovirals for reducing the risk of mother-to-child transmission of HIV infection. | 2011 | Cochrane | Twenty-five trials | 18,901 participants | To determine whether, and to what extent, antiretroviral regimens aimed at decreasing the risk of mother-to-child transmission of HIV infection achieve a clinically useful decrease in transmission risk, and what effect these interventions have on maternal and infant mortality and morbidity. | Pregnant women with HIV infection or infants born to mothers with HIV infection. | Any antiretroviral regimen with the specific aim of decreasing the risk of mother-to-child transmission of HIV infection. 1. Nucleoside analogue reverse transcriptase inhibitors: i) This class of drugs includes zidovudine (ZDV, previously known as AZT), lamivudine (3TC), didanosine (ddI), stavudine (d4T) and abacavir (ABC). 2. Non-nucleoside analogue reverse transcriptase inhibitors. i) This class of drugs includes nevirapine (NVP), delavirdine and efavirenz. 3. Protease inhibitors. i) This class of drugs includes indinavir, ritonavir, nelfinavir and saquinavir. Some of the more commonly used antiretroviral drugs in pregnancy are: Zidovudine, Nevirapine and Combination ARV treatment | Drug vs placebo ZDV given to mothers from 36 to 38 weeks gestation, during labour and for 7 days after delivery significantly reduced HIV infection at 4-8 weeks (Efficacy 32.00%; 95% CI 1.50 to 62.50), 3 to 4 months (Efficacy 33.07%; 95% CI 5.57 to 60.57), 6 months (Efficacy 34.55%; 95% CI 9.05 to 60.05), 12 months (Efficacy 34.31%; 95% CI 9.30 to 59.32) and 18 months (Efficacy 29.74%; 95% CI 2.73 to 56.75). ZDV given to mothers from36 weeks gestation and during labour significantly reduced HIV infection at 4 to 8 weeks (Efficacy 43.78%; 95% CI 8.78 to 78.78) and 3 to 4 months (Efficacy 36.95%; 95% CI 2.94 to 70.96) but not at birth. ZDV plus lamivudine (3TC) given to mothers from 36 weeks gestation, during labour and for 7 days after delivery and to babies for the first 7 days after birth (PETRA ’regimen A’) significantly reduced HIV infection (Efficacy 62.75%; 95% CI 40.76 to 84.74) and a combined endpoint of HIV infection or death (Efficacy 62.75 [, ]61.00%; 95% CI 40.76 to 84.74) at 4 to 8 weeks but these effects were not sustained at 18 months. ZDV plus 3TC given to mothers from the start of labour until 7 days after delivery and to babies for the first 7 days after birth (PETRA ’regimen B’) significantly reduced HIV infection (Efficacy 41.83%; 95% CI 12.82 to 70.84) and HIV infection or death at 4 to 8 weeks (Efficacy 35.91%; 95% CI 8.41 to 63.41) but the effects were not sustained at 18 months. Drugs and duration A single dose of NVP given to mothers at the onset of labour plus a single dose of NVP given to their babies immediately after birth (’HIVNET 012 regimen’) compared with ZDV given to mothers during labour and to their babies for a week after birth resulted in lower HIV infection rates at 4-8 weeks (Efficacy 41.00%; 95% CI 11.84 to 70.16), 3-4 months (Efficacy 38.91%; 95% CI 11.24 to 66.58), 12 months (Efficacy 35.98 [9.25, 62.71]36.00%; 95% CI 8.56 to 63.44) and 18 months (Efficacy 39.15%; 95% CI 13.81 to 64.49). In addition, the NVP regimen significantly reduced the risk of HIV infection or death at 4-8 weeks (Efficacy 41.74%; 95% CI 14.30 to 69.18), 3 to 4 months (Efficacy 40.00%; 95% CI 14.34 to 65.66), 12 months (Efficacy 32.17%; 95% CI 8.51 to 55.83) and 18 months (Efficacy 32.57 [9.93, 55.21]33.00%; 95% CI 9.93 to 55.21). A single dose of NVP given to babies immediately after birth plus ZDV given to babies for 1 week after birth compared with a single dose of NVP given to babies only significantly reduced the HIV infection rate at 4 to 8 weeks (Efficacy 36.79%; 95% CI 3.57 to 70.01).  ZDV given to mothers from 14 to 34 weeks gestation and during labour and to babies for the first 6 weeks after birth significantly reduced HIV infection in babies at 18 months (Efficacy 66.22%; 95% CI 33.94 to 98.50). ZDV given to mothers from 36 weeks gestation and during labour with no treatment to babies (’Thai-CDC regimen’) significantly reduced HIV infection at 4 to 8 weeks (Efficacy 50.26%; 95% CI 13.80 to 86.72) but not at birth.  Triple therapy In a breastfeeding population, a trial of TRIPLE regimen commenced at 34 weeks compared with only ZDV for the same period until labour when sdNVP was added found no babies infected with HIV at birth in either group and at 6 months post delivery there was no statistically significant difference in HIV infection between groups (Efficacy -84.62%, 95%CI: -490.35 to 321.11). The infants in the TRIPLE group did not receive any drugs while those in the ZDV group received sdNVP at birth. In a non-breastfeeding population, a trial compared a protease inhibitor-based TRIPLE regimen combination of lopinavir/ritonavir, ZDV and lamivudine from 26 to 34 weeks gestation through 6 months post-partum with a shorter regimen of ZDV from 28 to 36 weeks, then ZDV and 3TC and sdNVP at onset of labour, followed by ZDV and 3TC for one week after delivery. Infants in both groups received sdNVP within 72 hours of delivery and ZDV for one week. There was no statistically significant difference between groups in HIV infection at birth (Efficacy 18.18%, 95%CI -83.48 to 119.84) or at four to eight weeks (Efficacy 31.25%, 95%CI - 29.29 to 91.79). At six months, HIV infection was higher but not statistically significantly so in the non-TRIPLE group (Efficacy 42.35%, 95%CI -0.57 to 85.27). At 12 months HIV infection was statistically significantly higher in the non-TRIPLE group (Efficacy = 42.11%, 95%CI 0.66 to 83.56). At 6 months, the HIV infection or death incidence remained higher in the non-TRIPLE group (RR 34.13, 95%CI [-0.29 to 68.55) and at 12 months this difference was statistically significant (RR 36.20, 95%CI 5.92 to 66.48). |
| **Childbirth** | | | | | | | | | | | |
| Treatment of preterm rupture of membranes (PROM)and preterm prelabour rupture of membranes (pPROM) | Systematic Review | Kenyon, S 42 | Antibiotics for preterm rupture of membranes. | 2010 | Cochrane | 22 trials, | 6800 women and babies | To evaluate the immediate and long-term effects of administering antibiotics to women with PROM before 37 weeks, on maternal infectious morbidity, neonatal morbidity and mortality, and longer-term childhood development. | Women with preterm (less than 37 weeks) rupture of the membranes | • Ten trials tested broad spectrum penicillin either alone or in combination. • Five trials tested macrolide antibiotics (erythromycin) either alone or in combination and one tested clindamycin and gentamycin. The duration of treatment varied between two doses and 10 days with five trials opting for a maximum of seven days of treatment. Four trials treated women until delivery.  • In four trials, women were treated with oral antibiotic alone.  • Three trials, women were treated with intravenous antibiotic alone.  • Six trials, women were treated with a combination of intravenous and oral antibiotics • The six non-placebo controlled but randomised studies, which contributed data to the outcome measure perinatal death alone, were: Two trials compared three versus five days of ampicillin | The use of antibiotics following preterm rupture of membranes (PROM) is associated with a statistically significant reduction in chorioamnionitis (risk ratio (RR) 0.66, 95% confidence interval (CI) 0.46 to 0.96. There was a significant reduction in the numbers of babies born within 48 hours (RR 0.71, 95%CI 0.58 to 0.87). Neonatal infection (RR 0.67, 95% CI 0.52 to 0.85) (12 trials/1680 babies) was statistically significantly reduced in the babies whose mothers received antibiotics. One trial assessed the use of surfactant and it found a statistically significant reduction (RR 0.83, 95% CI 0.72 to 0.96) of babies requiring oxygen.   Overall the numbers of babies requiring oxygen therapy (RR 0.88, 95% CI 0.81 to 0.96) (one trial/4809 babies). The babies in the treatment groups spent 5.05 days less in neonatal intensive care (mean difference (MD) -5.05, 95%CI -9.77 to -0.33) (three trials/225 babies) and their birth weight was greater by 54 g (MD53.83, 95% CI 7.06 to 100.60) (12 trials/6374 babies). There was a significant reduction in the number of babies with an abnormal cerebral ultrasound scan prior to discharge from hospital (RR0.81, 95%CI 0.68 to 0.98) (12 trials/6289 babies).   Erythromycin versus co-amoxiclav: There was no significant difference in any index of neonatal morbidity except for necrotising enterocolitis, which was statistically significantly less frequent after erythromycin (RR 0.46, 95% CI 0.23 to 0.94) No obvious disadvantage to the three day regimen. |
| Treatment of preterm rupture of membranes (PROM)and preterm prelabour rupture of membranes (pPROM) | Systematic Review | Cousens, S 43 | Antibiotics for pre-term pre-labour rupture of membranes: prevention of neonatal deaths due to complications of pre-term birth and infection. | 2010 | Int J Epidemiol | 18 RCTs - All HIC except for 3 (Turkey, Mozambique and Chile) | 4581 newborns. | To review the evidence for and estimate the effect on neonatal mortality due to pre-term birth complications or infection, of administration of antibiotics to women with pPROM, in low and middle-income countries | Women with a risk of preterm labour | Women with pre-term rupture of membranes were treated with antibiotics. The antibiotic used, dose and duration varied between trials. Six trials used ampicillin, four erythromycin, two penicillin and one each used amoxicillin, mezlocillin, piperacillin. Two studies used combinations of two or more of these antibiotics, whereas one trial used a combination of clindamycin and gentamycin. | Effect on all-cause neonatal mortality 15 studies. (RR) was 0.90 [95% confidence interval (CI) 0.72, 1.12; P=0.33] Effects on severe morbidity 13 studies respiratory distress syndrome RR= 0.88 (95% CI 0.80, 0.97) risk of necrotizing enterocolitis RR= 0.76 (95% CI 0.56, 1.05) intra-ventricular haemorrhage RR= 0.67 (95% CI 0.49, 0.92). Sepsis RR= 0.61 (95% CI 0.48, 0.77)  STUDIES IN LOW AND MIDDLE-INCOME COUNTRIES: none |
| Cortico-steroids for preventing respiratory distress syndrome | SR | Brownfoot, FC 44 | Different corticosteroids and regimens for accelerating foetal lung maturation for women at risk of preterm birth. | 2009 | Cochrane | Ten trials | 1089 women and 1161 infants | To assess the effects of different corticosteroid regimens. To assess the effects on foetal and neonatal morbidity and mortality, on maternal morbidity and mortality and on the child and adult in later life or administering different types of corticosteroids (dexamethasone or betamethasone) different corticosteroid dose regimens, including timing, frequency and mode of administration | Women with a singleton / multiple pregnancy | In 9 trials dexamethasone was compared to betamethasone.  Four different corticosteroid regimens were used: • five trials compared 24 mg dexamethasone (6 mg, four doses, 12 hourly) and 24mg betamethasone (12 mg, two doses, 24 hourly)  • two trials compared 24 mg dexamethasone (12 mg, two doses, 12 hourly) and 24mg betamethasone (12 mg, two doses, 12 hourly) • one trial compared 16 mg dexamethasone (4 mg, four doses, 12 hourly) and 24 mg betamethasone (6 mg, four doses, 12 hourly) • one trial compared 24 mg dexamethasone (12 mg, two doses, 12 hourly) and 24 mg betamethasone (12 mg, two doses, 24 hourly)  One trial of 170 women and 188 infants compared 32 mg oral dexamethasone (8 mg, four doses, 12 hourly) and 24 mg intramuscular dexamethasone (6 mg, four doses, 12 hourly). | dexamethasone decreased the incidence of intra-ventricular haemorrhage compared with betamethasone (RR 0.44, 95%CI 0.21, 0.92) |
| Cortico-steroids for preventing respiratory distress syndrome | Systematic Review | Mwansa-Kambafwile, J 45 | Antenatal steroids in preterm labour for the prevention of neonatal deaths due to complications of preterm birth | 2010 | Int J Epidemiol | 18 RCTs (14 in high-income countries and 4 in Brazil, South Africa, Tunisia and Jordan) There were 19 observational studies. All were high income countries except for two (one in Iran and the other in Brazil). | Neonates | To review the evidence globally for and estimate the effect on neonatal mortality due to preterm birth complications of antenatal steroid administration to women before anticipated preterm labour | Women in preterm labour or at high risk of preterm labour | antenatal steroids were given as therapy in premature labour and where birth occurred between 24 h and 7 days after treatment. A placebo or a suitable control group that was similar to the experimental group except that it did not receive antenatal steroids | CAUSE-SPECIFIC MORTALITY 18 RCTs (mostly high income countries) RR= 0.69 (0.58-0.81) 4 RCTs (all middle income countries) RR= 0.47 (0.35 – 0.64)  Two MIC (Brazil and Iran) Observational R = 0.55 (0.40–0.76)  SEVERE MORBIDITY 21 RCT (most came from HIC) R= 0.66 (0.59 – 0.73) 4 RCTs (All middle income) R= 0.63 (0.49 – 0.81) |
| Magnesium Sulphate for treatment of pregnancy induced hypertention (HPT) or eclampsia | Systematic review | Duley, L 46 | Magnesium sulphate versus phenytoin for eclampsia. | 2010 | Cochrane | Seven trials, 2-USA, 2-SA 1-Brazil, India, Collaborative trial | 972 women | To assess the effects of magnesium sulphate compared with phenytoin when used for the care of women with eclampsia. | Women with a clinical diagnosis of eclampsia at randomisation irrespective of whether they were before or after deliver | Most of the women who received magnesium sulphate in these trials had 4 g as a loading dose, and maintenance therapy was either by an intramuscular regimen or an infusion of 1 g/hour: • Three trials, magnesium sulphate maintenance was given by intravenous infusion. • One trial maintenance therapy was given by intramuscular injection.  • Another study each hospital in the study chose whether they used intravenous or intramuscular maintenance therapy. • One study used a loading dose only. | MATERNAL OUTCOMES No statistical significance for Maternal death.  Magnesium sulphate is associated with at least a halving in the risk ratio of recurrence of seizures, when compared with phenytoin (RR 0.34, 95% CI 0.24 to 0.49; six trials, 972 women). There was a reduction in the risk ratio of pneumonia for women allocated magnesium sulphate rather than phenytoin (RR 0.44, 95%CI 0.24 to 0.79; one trial; 775women). The risk ratio of admission to an intensive care unit was reduced for women allocated magnesium sulphate, rather than phenytoin (RR 0.67, 95% CI 0.50 to 0.89; one trial; 775 women), and so was the need for ventilation (RR 0.68, 95% CI 0.50 to 0.91; two trials; 825 women).  BABY OUTCOMES No clear difference between the treatment groups in the risk ratio of stillbirth, perinatal death, neonatal death. Women who received magnesium sulphate rather than phenytoin were less likely to have a baby with an Apgar below eight at one minute (RR 0.78, 95% CI 0.66 to 0.93; one trial; 518 infants). Infants born to women allocated magnesium sulphate rather than phenytoin were less likely to be admitted to a special care baby unit (RR 0.73, 95% CI 0.58 to 0.91; one trial; 518 babies), and less likely to stay in the special care baby unit for more than seven days (RR 0.53, 95% CI 0.33 to 0.86; one trial; 518 babies). Infants born to women allocated magnesium sulphate rather than phenytoin also had a reduced risk ratio of death or in special care baby unit for more than seven days (RR 0.77, 95% CI 0.63 to 0.95; one trial; 643 babies). |
| Provision of Clean Delivery kits | Systematic review | Hundley, V 47 | Are birth kits a good idea? A systematic review of the evidence | 2011 | Midwifery | 9 studies | Not mentioned | Traditonal birth attendants/ Skilled birth attendants/ SBA, Dayas (Traditonal birth attendants in Pakistan), Dais (traditional birth attendants in Egypt) | To identify the current state of knowledge regarding the effects of births kits on clean birth practices and on newborn and maternal outcomes | • The birth kit was primarily used in home settings or within a facility (3 studies).  • Kit + education (TBAs) + antenatal outreach clinics (3- day training course for TBAs on: antenatal, intrapartum and postpartum care; clean birth; use of a birth kit; referring women for emergency obstetric care; care of the newborn). • Kit + community health promotion + education (TBAs). TBA training on: ‘use of clean water instead of dung compound on the freshly cut umbilical cord’. Health promotion interventions not described. • Kit + education (women). Women and relatives received a demonstration regarding the use of the clean blade and spirit. • Kit + tetanus toxoid immunisation+ education (dais). Some local dais received training’ but the details of this are not given. • Kit + topical chlorhexidine for some groups+ education (women). (Women received ‘basic educational messages regarding umbilical cord care’ and ‘infant thermal care’. The birth kit included a pictorial insert.) • Kit + education (dayas) + education (women) + motivational strategies (SBAs). (Dayas received training on importance of antenatal care, how to use a birth kit and how to dispose of it. Women received education on the importance of a birth kit, its use and disposal. Pictorial insert in birth kit included danger signs that required immediate referral.) • Kit + education (dayas) + education (women) + motivational strategies (SBAs). (Dayas received training module on the WHO six cleans, how to use a birth kit and how to dispose of it. Women received education on the importance of a birth kit, its use and disposal. Pictorial insert in birth kit included danger signs that required immediate referral.) • Birth kit + education (women). Women received an explanation on how to use the birth kit components with aid of pictorial instructions, and education on the WHO six cleans. • Kit + public awareness campaign. (No specific educational intervention but the birth kit included a pictorial insert) The “six cleans” include clean hands, clean perineum, clean delivery surface, clean cord cutting implement, clean cord tying, and clean cord care | NEONATAL OUTCOMES One study (Jokhio) showed a reduction in PNMR OR=0.70 (95%CI0.59,0.82), Stillbirth OR=0.69 (95%CI0.57,0.83), NNMR OR=0.71 (95%CI0.62,0.83)  Another study (Meegan)showed a reduction in NNMR = RR=0.17 (95%CI0.13,0.23) (Meegan) showed a tetanus related mortality (RR=0.01 (95%CI0.001,0.09) Another study (Kapoor) showed a Relative decrease in NNMR in 4years =21.83%  Another study (Garner)showed a reduction in sepsis. RR=0.12 (95%CI0.02,0.93)   Omphalitis (4studies) Mullany, RR=0.49 (95%CI 0.43,0.56) Darmstadt,OR=0.42 (95%CI 0.18,0.97) Winani, OR=0.08 (95%CI 0.03,0.19) Tsu, RR= 0.45 (95%CI 0.25 to 0.81)  Jokhio showed a reduction in maternal mortality OR=0.74 (95%CI0.45,1.23), puerperal sepsis, OR=0.17 (95%CI0.13,0.23), haemorrhage OR=0.61 (95%CI0.47,0.79) Darmstadt and Winani showed a reduction in puerperal sepsis OR=0.11 (95%CI0.01,1.06), OR=0.31 (95%CI0.18,0.54) .In three studies women who delivered at home reported higher birth kit use than those who delivered in facilities home 63%; facility 54% home 81.8%, facility 67.7%; An intervention package which included a birth kit was associated with reduced newborn mortality in three studies One intervention including training and support for TBAs and, as a result, the intervention group demonstrated increased referral to emergency obstetric care (OR 1.50; 95% CI 1.19, 1.91). |
| Presence of birth attendants during delivery | Meta-analysis | Yakoob, M 56 | The effect of providing skilled birth attendance and emergency obstetric care in preventing stillbirths. | 2011 | BMC Public Health | 21 studies. The studies identified were all conducted within Africa or Asia. | Not mentioned | To ascertain the effect of provision of skilled birth attendance as well as basic and emergency obstetric care on stillbirths | Pregnant women | Community based skilled birth attendants and basic emergency obstetric car and comprehensive emergency obstretric care. A skilled birth attendant is described as an accredited health professional – such as a midwife, doctor or nurse – who has been educated and trained to proficiency in the skills needed to manage normal (uncomplicated) pregnancies, childbirth and the immediate postnatal period, and in the identification, management and referral of complications in women and newborns.  Basic Emergency (or Essential) Obstetric Care (BEOC) comprises of 7 “signal functions” that include: the use of intravenous/intramuscular antibiotics, intravenous/intramuscular oxytocin, intravenous/intramuscular anticonvulsants, manual removal of retained placenta and removal of retained products of conception(e.g. by Manual Vacuum Aspiration), assisted vaginal delivery and basic newborn resuscitation . Comprehensive Emergency (or Essential) Obstetric Care (CEOC) includes all BEOC signal functions plus Caesarean section and blood transfusion | Impact of skilled birth attendance on stillbirth. Four before and after studies RR =0.77 (0.69 - 0.85). Impact of community-based skilled birth attendance on perinatal mortality. 4 trials RR = 0.88 [0.82, 0.95] Provision of basic and comprehensive emergency obstetric care |
| Presence of birth attendants during delivery | Systematic review | Sibley, L 62 | Traditional birth attendant training for improving health behaviours and pregnancy outcomes. | 2007 | Cochrane | Four studies, (Pakistan, Malawi, Bangladesh, Guatemala) | Over 2000 TBAs and nearly 27,000 women | To assess effects of TBA training on health behaviours and pregnancy outcomes. | Traditional birth attendants (TBAs) and lactating mothers living in the intervention and control areas | In three studies, the authors categorized TBAs targeted by interventions as ’untrained’ TBAs . However, in two of these studies, the large majority of TBAs in both the intervention and control groups had received some prior biomedical training through government or non-governmental organizations, or both; for example, 71% of TBAs in the Bangladesh study and in the Guatemala study had been given government training since 1935. In the remaining study all TBAs had received some prior biomedical training  In two studies, the interventions consisted of educational instruction in management of normal delivery, timely detection and referral of women with obstetric complications, as well as importance of linking women to essential obstetric care services. In each study, TBA training was part of a package of interventions including community and improved facility-based care components.  In one study, the improvements involved staff training in essential and emergency obstetric care and clinical outreach by team physicians.  In another study other, improvements consisted of staff training in standard protocols for managing maternal and neonatal emergencies and sensitization to TBAs and women referred by TBAs. | Advice about introduction of complementary foods The effect-size estimate of the post-test difference at both three months (OR= 3.11 (95% ci 1.63 to 5.92, p < 0.001) and seven months follow up (OR= 2.07 (95% ci 1.10 to 3.90, p = 0.02) favour the intervention group Women living in intervention clusters were significantly more likely to have been referred to any health facility for a complication of pregnancy, delivery, or postpartum period, than women living in control clusters (adjusted OR= 1.50, 95% ci 1.18 to 1.90) Comparison of the baseline to post-intervention difference for timely referral of obstetric complications of malpresentation, prolonged labour and preterm labour among women referred by TBAs was significant for intervention communities (OR = 2.25, p = 0.007) but not control communities (OR 1.17, p = 0.510), and there was no significant difference for timely referral between the groups maternal outcomes frequency of obstructed labour was significantly higher among women living in intervention clusters compared with women living in control clusters (cluster adjusted OR= 1.26, 95% ci 1.03 to 1.54) Frequency of haemorrhage (antepartum, intrapartum, and postpartum combined) was significantly lower among women living in the intervention clusters, compared with women living in the control clusters (adjusted OR= 0.61, 95% ci 0.47 to 0.79) Frequency of puerperal sepsis was significantly lower among women living in intervention clusters compared with control clusters (adjusted OR= 0.17, 95% ci 0.13 to 0.23)  Neonatal Outcomes Perinatal deaths among singeltons were lower in the intervention groups adjusted OR= 0.70, 95% ci 0.59 to 0.83) in the intervention communities before and after the intervention, respectively, corresponding to significant decrease in death rate from 22% to 12% (OR= 2.13, p = 0.032) the stillbirth rate difference was significant, 31% lower in intervention compared with control clusters (adjusted OR= 0.69, 95% ci 0.57 to 0.83) the neonatal death rate difference was significant, 29% lower in the intervention compared with the control clusters (3.5% versus 4.88, adjusted OR= 0.71, 95% ci 0.61 to 0.82) |
| Presence of birth attendants during delivery | SR | Darmstadt, G 58 | 60 Million non-facility births: who can deliver in community settings to reduce intrapartum-related deaths? | 2009 | Int J Gynaecol Obstet | For community-based skilled birth attendants 4 studies were used in meta-analyses for Perinatal mortality and 3 for Early neonatal mortality rate. For community health worker packages 4 studies were used in the Meta-analysis for Perinatal mortality and 3 for Early neonatal death. | No total number mentioned | To review the evidence for the effect of care by different community cadres during pregnancy and childbirth. The effect of provision of skilled birth attendance as well as basic and emergency obstetric care on stillbirths. | Skilled/ trained birth attendants, traditional birth attendants who assist with deliveries. Community health workers | SBA’s medically qualified providers with midwifery skills (midwife, nurse or doctor) who have been trained to proficiency in the skills necessary to manage normal deliveries and diagnose, manage, or refer obstetric complications, ideally who live in, and are part of, the community they serve. They must be able to manage normal labour and delivery, perform essential interventions, start treatment and supervise the referral of mother and baby for interventions that are beyond their competence or not possible in a particular setting. SBAs may provide domiciliary childbirth care in the home or in community birthing centers.  Community birthing centers may range from a simple “maternity home” to a rural hospital that is staffed 24 hours a day by an SBA who provides basic emergency obstetric care (includes BEmOC, caesarean delivery and blood transfusion).  CHWs members of the communities where they work, selected by the communities, answerable to the communities for their activities, supported by the health system but not necessarily a part of its organization, and have shorter training than professional workers. CHWs differ from TBAs in that they tend to be younger, more educated, and less closely bound to traditional care practices. Characteristics and training of CHWs may vary by region and even within countries, however, depending on local policy | INCREASING SKILLED CHILDBIRTH CARE IN THE COMMUNITY A meta-analysis of observational before-and-after data from 4 studies of SBA training showed a reduction in PMR (RR 0.88; 95% CI, 0.83–0.95) and a 13% reduction in ENMR (RR 0.87; 95% CI, 0.79–0.97) . A range of 22%–47% reduction in mortality of the “non-breathing baby” in three studies.  TRAINING TBAS FOR PROVIDING LABOR AND CHILDBIRTH CARE No meta-analysis,   USING CHWS TO PROMOTE BIRTH PREPAREDNESS AND CARE-SEEKING, WITH OR WITHOUT PROVISION OF NEWBORN CARE AT BIRTH: In a meta-analysis that included all the available higher-quality evaluations of primary and secondary prevention of intrapartum-related outcomes through CHWs (2cRCTs and 2 quasi-experimental trials the pooled effect on PMR was RR 0.72 (95% CI, 0.62–0.84) and on ENMR was RR 0.64 (95% CI, 0.56–0.73) |
| **Neonatal** | | | | | | | | | | | |
| Kangaroo Mother Care | Systematic review | Conde-Agudelo, A 59 | Kangaroo mother care to reduce morbidity and mortality in low birthweight infants. | 2011 | Cochrane | Sixteen studies | 2518 infants | To determine whether there is evidence to support the use of KMC in LBW infants as an alternative to conventional neonatal care. | Infants born with LBW (less than 2500g) | (1) Comparisons of KMC with conventional neonatal care in LBW infants. This was regardless of duration of intervention, breast- feeding patterns, and irrespective of whether discharge from hospital was early or not. (2) Comparisons of early onset KMC (starting within 24 hours post-birth) with late onset KMC (starting after 24 hours post- birth) in LBW infants, irrespective of infant stabilization status. | Overall, KMC was associated with a statistically significant reduction in the risk of mortality at discharge or 40 – 41 weeks’ postmenstrual age (typical RR 0.60, 95% CI 0.39 to 0.93) subgroup of studies conducted in low/middle income countries (mortality at discharge or 40 - 41 weeks’ corrected gestational age: six studies, 1554 infants; typical RR 0.58, 95% CI 0.37 to 0.90. The trial in which KMC was used in unstabilised infants (RR 0.57, 95% CI 0.33 to 1.00) KMC decreased length of hospital stay by 2.4 days (95%CI 0.7 to 4.1) in a meta-analysis of nine studies that used intermittent KMC. Mothers of the KMC group were more satisfied with method of care than mothers of the control group (91% versus 78%; RR 1.17, 95% CI 1.05 to1.30; 269 mothers)  KMC was associated with an increase in the likelihood of exclusive breastfeeding at discharge or 40 – 41 weeks’ postmenstrual age (RR 1.21, 95% CI 1.08 to 1.36 four studies, 1197 mothers) and at 1 - 3 months follow up ( RR 1.20, 95% CI 1.01 to 1.43; five studies, 600 mothers) or any (exclusive and/or partial) breastfeeding at discharge or 40 - 41 weeks’ postmenstrual age ( RR 1.25, 95% CI 1.06 to 1.47; eight studies, 1440 mothers) at 1 - 2 months follow up RR 1.33, 95% CI 1.00 to 1.78; six studies, 538 mothers) and at three months follow up (RR 1.14, 95% CI 1.06 to 1.23; five studies, 924 mothers) criteria in the great majority of included studies. Infection / Illness In stabilized LBW infants, KMC was associated with a statistically significant reduction in severe infection/sepsis at latest follow up ( RR 0.57, 95% CI 0.40 to 0.80 NNT for benefit 19, 95% CI 13 to 40; six trials, 1250 infants) severe illness at six months follow up (RR0.30, 95%CI 0.14 to 0.67;NNTfor benefit 8, 95%CI 7 to 17; one trial, 283 infants) nosocomial infection/sepsis at discharge or 40 - 41 weeks’ corrected gestational age (RR 0.42, 95% CI 0.24 to 0.73; I2 = 0%; NNT for benefit 17, 95%CI 13 to 37; two trials, 777 infants) lower respiratory tract disease at six months follow up (RR 0.37, 95% CI 0.15 to 0.89; NNT for benefit 13, 95% CI 9 to 73; one trial, 283 infants) and hypothermia at discharge or 40 - 41 weeks’ corrected gestational age (RR 0.23, 95% CI 0.10 to 0.55; I2 = 56%; NNT for benefit 4, 95% CI 3 to 7; four trials, 469 infants; ) Infant growth KMC infants gained more weight per day (typical MD 3.9 g, 95% CI 1.9 to 5.8; nine trials, 936 infants) and length (typicalMD0.29cm, 95%CI 0.27 to 0.31; two trials, 251 infants) and head circumference (typical MD 0.18 cm, 95% CI 0.09 to 0.27; three trials, 369 infants) per week than controls. Compared with late onset KMC, early onset KMC was associated with a statistically significant reduction in body weight loss from birth to 48 hours post-birth (MD 43.3 g, 95% CI 5.5 to 81.1) and length of hospital stay (MD -0.9 days, 95% CI -1.2 to -0.6) |
| Kangaroo Mother Care | Systematic review | Moore, E 60 | Early skin-to-skin contact for mothers and their healthy newborn infants. | 2012 | Cochrane | 34 RCTs | 2177 participants (mother-infant dyads). | To assess the effects of early Skin to skin care (SSC) on breastfeeding, physiological adaptation, and behaviour in healthy mother-newborn dyads. | Mothers and their healthy full term or late preterm newborn infants (34 to less than 37 completed weeks’ gestation) having early SSC starting less than 24 hours after birth, and controls undergoing standard patterns of care. | Early SSC for term or late preterm infants can be divided into several subcategories.  (1) In ’birth SSC’, the infant is placed prone skin-to-skin on the mother’s abdomen or chest during the first minute post birth. The infant is suctioned while on the mother’s abdomen or chest, if medically indicated, thoroughly dried and covered across the back with a prewarmed blanket. To prevent heat loss, the infant’s head may be covered with a dry cap that is replaced when it becomes damp. Ideally, all other interventions are delayed until at least the end of the first hour post birth or the first successful breastfeeding.  (2) In ’very early SSC’, beginning approximately 30 to 40 minutes post birth, the naked infant, with or without a cap, is placed prone on the mother’s bare chest. A blanket is placed across the infant’s back.  (3) ’Early SSC’ can begin anytime between one and 24 hours post birth. The baby is naked (with or without a diaper and cap) and is placed prone on the mother’s bare chest between the breasts. The mother may wear a blouse or shirt that opens in front, or a hospital gown worn backwards, and the baby is placed inside the gown so that only the head is exposed. What the mother wears and how the baby is kept warm and what is placed across the baby’s back may vary. What is most important is that the mother and baby are in direct ventral-to-ventral SSC and the infant is kept dry and warm. The characteristics of the intervention varied greatly between studies.  Duration of skin-to-skin (SSC) ranged from approximately 15 minutes in 4 studies to a mean of 37 of 48 hours (84%) of continuous SSC. In this study all dyads received 24 minutes of SSC before randomization. Another study reported that SSC mothers gave SSC 22% of the time and held their wrapped infants for 11.6% of the observation period. Although SSC began by 0 to 15 minutes post birth in 18 of the 34 studies, the SSC dyads in another study could not begin until four hours post birth because of hospital policy. SSC did not begin until a mean of 21.3 hours post birth in another study of late preterm infants 34 to 36 weeks’ gestational age. In 22 of the 34 studies the infants were given the opportunity to suckle during SSC but only five studies documented the success of the first breastfeeding using a validated instrument, the Infant Breastfeeding Assessment Tool. The amount of assistance the mothers received with breastfeeding during SSC was unclear in many of the research reports. Substantial differences were found between studies in the amount of separation that occurred in the control group. In eight studies infants were removed from their mothers immediately post birth and reunited 12 to 24 hours later. In five studies the mothers held their swaddled infants for about five minutes soon after birth and then were separated from their infants. Control mothers held their swaddled infants six times for 60 minutes in one study, 20 minutes in another study, 60 minutes in another study and for two hours in the recovery room in the last study. The swaddled control infants in one study were reunited with their mothers after the episiotomy repair. Control infants in a study were separated from their mothers for a mean of 21 minutes and in in another study for a mean of 51 minutes post-caesarean birth. There were four groups in a further study; an SSC group, a mother’s arms group where the infants were held swaddled or dressed, a nursery group and a reunion group where the infants were taken to the nursery immediately post birth for 120 minutes but reunited with their mothers for rooming-in on the postpartum unit. In the last study control mothers held their wrapped infants 13.9% of the time (M = 6.67 hours). | Breastfeeding rates/duration More SSC dyads were still breastfeeding one to four months post birth (average risk ratio (RR) 1.27, 95% confidence interval (CI) 1.06 to 1.53) (13 studies) Seven studies with 324 mother/infant pairs reported data on the duration of breastfeeding in days. Six of the seven studies found a longer duration of breastfeeding in the SSC dyads (mean difference (MD) 42.55 days, 95% CI -1.69 to 86.79). SSC infants had higher SCRIP scores (SCRIP scores (a measure of infant cardio- respiratory stability in preterm infants that evaluates infant heart rate, respiratory rate and oxygen saturation) during the first six hours post birth, indicating better stabilization (MD 2.88, 95% CI 0.53 to 5.23) (Analysis 1.3). A subset of infants below 1800 grams birth weight also demonstrated better stabilization (MD 4.92, 95% CI -1.67 to 11.51) but this result did not reach statistical significance  Blood glucose 75 to 90 minutes following the birth was measured in two studies with 94 infants; blood glucose was higher in SSC infants (MD 10.56 mg/dL, 95% CI 8.40 to 12.72) and this result was statistically significant. More infants were exclusively breastfeeding up to three to six months post birth in three studies (n = 149) (RR 1.97, 95% CI 1.37 to 2.83)  For maternal – infant bonding SSC dyads appeared more mutual and reciprocal (MD1.30, 95%CI 0.24 to 2.36) than those who were separated immediately post birth and later reunited for rooming-in |
| Vitamin A Supplementation - preventive | Systematic review, | Haider, B 62 | Neonatal vitamin A supplementation for the prevention of mortality and morbidity in term neonates in developing countries. | 2011 | Cochrane | Seven studies | 51,446 neonates | To evaluate the role of vitamin A supplementation in term neonates in developing countries with respect to the prevention of mortality and morbidity | All term neonates (born between 37 to 42 weeks of gestational age) up to 28 days after birth were included. | • Indonesia – The neonates were randomly assigned to receive a single oral dose of vitamin A (50,000 IU) or placebo within 24 hours of delivery.  • Nepal -administered vitamin A (50,000 IU in < one month old neonates and 100,000 in one to five month old infants) or placebo • India - neonates were given two doses of vitamin A or placebo with the first dose being administered within the first 48 hours of delivery and the second dose within 24 hours of the first dose. • Zimbabwe - The vitamin A dose for mothers was 400,000 IU and for neonates it was 50,000 IU • Bangladesh - neonates were administered vitamin A (50,000 IU) • Guinea Bissau-All neonates with Birth weight at least 2500 g, without any serious medical condition or malformation, for whom parental consent was available were randomised to either oral drops of vitamin A (50,000 IU) or placebo. | All-cause infant mortality at six months of age: risk ratios based on cumulative risk (%) Infant death risk is 18% lower than control, which is statistically significant (typical RR 0.82; 95% CI 0.68 to 0.99) (Three studies) but showed a statistically significant reduction of 14%in the risk of death from any cause for neonates supplemented with vitamin A as compared to control (typical RR 0.86; 95% CI 0.77 to 0.97) for the five studies. All-cause infant mortality at 12 months of age Vitamin A supplementation failed to show any significant effect on infant mortality at 12 months of age compared to control (typical rate ratio 1.03; 95% CI 0.87 to 1.23; I2 49%). |
| Infant Vitamin A Supplementation - preventive | Systematic Review | Imdad, A 69 | Vitamin A supplementation for preventing morbidity and mortality in children from 6 months to 5 years of age. | 2011 | Cochrane | 43 trials in 19 countries | 215633 children | To evaluate the effect of vitamin A supplementation (VAS) for preventing morbidity and mortality in children aged 6 months to 5 years. | Children living in the community and aged 6 months to 5 years | Synthetic oral Viitamin A Supplement was compared to either placebo or treatmentas- usual control groups, including trials of various doses and frequencies. Co-interventions (for example,multiple vitamin ormineral supplementation), must have been identical in both groups. | All – cause mortality: 17 trials –Vitamin A was associated with a 24% reduction in all-cause mortality (RR = 0.76 (95% CI 0.69 to 0.83)), The effect during the first year of life was similar (RR = 0.82 (95% CI 0.74 to 0.91)), Only 5 trials (7%) measured mortality between 13 and 60months, and the effect was similar (RR = 0.75 (95% CI 0.64 to 0.88)) Location: Eleven trials were conducted in Asia (RR = 0.69 (95% CI 0.61 to 0.79)), 5 in Africa (RR = 0.85 (0.73 to 0.98)), and 1 in Latin America (RR = 1.00 (0.14 to 7.08)). Age: Four trials reported separate effects for children aged 6 to 12 months (RR = 0.59 (95% CI 0.43 to 0.82)) and children aged 1 to 5 years (RR = 0.68 (0.57 to 0.81)) Diarrhoea mortality: Seven trials reported a combined 28% reduction in diarrhoea mortality (RR = 0.72 (95% CI 0.57 to 0.91)) Diarrhoea: Thirteen trials reported an 18% decrease in diarrhoea incidence (RR = 0.85 (95% CI 0.82 to 0.87)), Measles: Six trials reported a 50% decrease in measles incidence (RR = 0.50 (95% CI 0.37 to 0.67)) Vision – Bitot’s spots: Four trials reported a 53% reduction in Bitot’s spots prevalence (RR = 0.45 (95% CI 0.33 to 0.61)) Night blindness: One trial (Herrera 1992) reported a 47%reduction in night blindness incidence (RR = 0.53 (95% CI 0.28 to 0.99)). Two trials reported a 68% reduction night blindness prevalence (RR = 0.32 (95% CI 0.21 to 0.50))  Xeropthalmia: Two trials reported a 69% reduction in xerophthalmia prevalence (RR = 0.31 (95% CI 0.22 to 0.45))  Vit A deficiency: Four trials reported a 29% reduction in the number of VAD children (RR = 0.71 (95% CI 0.65 to 0.78)) Hospitalisation: One study reported a reduction of the likelihood of hospitalisations that approached statistical significance (RR = 0.64 (95% CI 0.40 to 1.02)) and a 38% reduction in the number of hospitalisations (RR = 0.62 (95% CI 0.42 to 0.93)). Vomiting (within 48 hours): Three trials reported a significant increase in risk of vomiting (RR= 2.75 (95%CI 1.81 to 4.19)) Fontanelle: Three trials reported fontanelle side effects, but only one could be analysed because the others reported insufficient data, which reported no effect (RR = 5.00 (95% CI 0.24 to 103.72)). |
| **Postnatal** | | | | | | | | | | | |
| Preventation and Treatment of Postnatal depression | Systematic review | Howard, L 65 | Antidepressant treatment for post-natal depression | 2001 | Cochrane | Two trials | 73 participants | To evaluate the effectiveness of different antidepressant drugs in addition to standard clinical care in the prevention of postnatal depression. To compare the effectiveness of different antidepressant drugs and with any other form of intervention for postnatal depression i.e. hormonal, psychological or social support. To assess any adverse effects of antidepressant drugs in either the mother or the foetus/infant. | Women who were pregnant or had given birth in the last six weeks, who were not taking any antidepressant medication at the start of the trial. | Any type of antidepressant medication at any dose alone or in combination with another treatment initiated in at least one arm of a trial compared with any other treatment, or placebo, or standard clinical care. Nortriptyline: - bedtime dose of 3 capsules given as soon as possible after birth, ideally within 24 hours, increase daily for 1 week postpartum, 20, 30, 40, 50, 60, 70mg/day and 75 mg to day 21. Serum level from day 14 determined dose from then on to achieve 50-150ng/ml serum level. Dose tapered from week 17 at 33% per week and discontinued at week 20 postpartum Sertraline: - single post breakfast dose in 2 identical opaque gelatin capsules | Only two eligible trials of antidepressants for the prevention of postnatal depression were identified. The first provided no evidence for the effectiveness of nortriptyline in preventing recurrence of postnatal depression, though the trial may have been underpowered. Less than half the women eligible for the trial took part, possibly because pregnant and nursing women do not want to take medication. Nortriptyline was no more effective than placebo in preventing a recurrence of postpartum major depressive Disorder.  The other study reported that sertraline was more effective than placebo in preventing a recurrence of postpartum major depression. 1. Fewer recurrences in women treated with sertraline (1/14) (0.07, 95%CI 0.00, 0.34) than placebo (4/8) (0.50, 95%CI, 0.16, 0.84) (Fishers exact p=0.04). 2.Time to recurrence was also quicker in the placebo group (exact Wilcoxon Gehan p=0.02).The observed hazard ratio was 9.09 (95%CI, 0.98, 88.3) |
| Antiretroviral therapy in HIV infected children under 3 | Systematic Review | Penazzato, M 66 | Optimisation of antiretroviral therapy in HIV-infected children under 3 years of age | 2013 | Cochrane | 8 studies addressing when to start treatment (n=2), what to start (n=3), whether to substitute lopinavir/ritonavir (LPV/r) with nevirapine (NVP) (n=1), whether to use an induction-maintenance ART strategy (n=1) and whether to interrupt treatment (n=1) | 3648 infants | To evaluate 1) when to start ART in young children (less than 3 years); 2) what ART to start with, comparing first-line non-nucleoside reverse transcriptase inhibitor (NNRTI) and protease inhibitor (PI)-based regimens; and 3) whether alternative strategies should be used to optimize antiretroviral treatment in this population: induction (initiation with 4 drugs rather than 3 drugs) followed by maintenance ART, interruption of ART and substitution of PI with NNRTI drugs once virological suppression is achieved on a PI-based regimen | perinatally HIV-infected children under 3 years of age | The following types of intervention were assessed: - Timing of treatment: use of early compared to deferred ART - Choice of treatment: use of NNRTI- versus PI-based regimens, in combination with any NRTI backbone - Substitution of LPV/r with NVP following initiation with PIbased regimens. - Interruption of treatment, compared to continuous early ART - Induction-maintenance treatment: Initiating ART with more than 3 antiretroviral drugs for an induction period, then moving to maintenance treatment with a standard 3-drug regimen | Treatment initiation – 2 studies in asymptomatic infants with good immunological status was associated with a 75%reduction (HR=0.25; 95%CI 0.12-0.51; p=0.0002) in mortality or disease progression in the one trial (in SA) with sufficient power to address this question. In a smaller pilot trial (in SA), median CD4 cell count was not significantly different between early and deferred treatment groups 12 months after ART. What to start with – 3 african trials  Regardless of previous exposure to nevirapine for PMTCT, the hazard for treatment failure at 24 weeks was 1.79 (95%CI 1.33, 2.41) times higher in children starting ART with a NVP-based regimen compared to those starting with a LPV/r-based regimen (p=0.0001) with no clear difference in the effect observed for children younger or older than 1 year. The hazard for virological failure at 24 weeks was overall 1.84 (95%CI 1.29, 2.63) times higher for children starting ART with a NVP-based regimen compared to those starting with a LPV/r-based regimen (p=0.0008) with a larger difference in time to virological failure (or death) between the NVP and LPV/rbased regimens when ART was initiated in the first year of life. LPV/r with NVP – 1 SA study Infants starting a LPV/r regimen and achieving sustained virological suppression who then substituted LPV/r with NVP after median 9 months on LPV/r were less likely to develop virological failure (defined as at least one VL greater than 50 copies/mL) compared with infants who started and stayed on LPV/r (HR=0.62, 95%CI 0.41, 0.92, p=0.02). However the hazard for confirmed failure at a higher viral load (>1000 copies/mL) was greater among children who switched to NVP compared to those who remained on LPV/r (HR= 10.19, 95% CI 2.36, 43.94, p=0.002). Induction-Maintenance 1 study in Uganda and Zimbabwe Children undergoing an induction-maintenance ART approach with a 4-drugNNRTI-based regimen for 36 weeks, followed by 3-drug ART, had significantly greater CD4 rise than children receiving a standard 3-drug NNRTI-based ART at 36 weeks (mean difference 1.70 [95%CI 0.61, 2.79] p=0.002) and significantly better viral load response at 24 weeks (OR 1.99 [95%CI 1.09, 3.62] p=0.02). However, the immunological and virological benefits were short-term.  Planned treatment interruption – 1 Kenyan study The one trial of treatment interruption that compared children initiating continuous ART from infancy with children interrupting ART was terminated early because the duration of treatment interruption was less than 3months in most infants. Children interrupting treatment had similar growth and occurrence of serious adverse events as those in the continuous arm. |
| **Across the first 1000 days** | | | | | | | | | | | |
| Community-level service delivery interventions | Systematic Review, | Lewin, S 71 | Lay health workers in primary and community health care for maternal and child health and the management of infectious diseases | 2010 | Cochrane | Eighty-two studies | 3568 | To assess the effects of LHW interventions in primary and community health care on maternal and child health and the management of infectious diseases. | Any lay health worker (paid or voluntary) including community health workers, village health workers, birth attendants, peer counsellors, nutrition workers, home visitors. | A lay health worker is a member of the community who has received some training to promote health or to carry out some healthcare services, but is not a healthcare professional. In the studies in this review, lay health workers carried out different tasks. These included giving help and advice about issues such as child health, child illnesses, and medicine taking. In some studies, lay health workers also treated people for particular health problems. Any intervention delivered by LHWs and intended to improve maternal or child health (MCH) or the management of infectious diseases. We included interventions if the description was adequate for us to establish that it was a LHW intervention. Where such detail was unclear, we contacted study authors, whenever possible,to establish whether the personnel described were LHWs. For the purposes of this review, a MCH or infectious diseases intervention was defined as follows. • Child health: any interventions aimed at improving the health of children aged less than five years. • Maternal health: any interventions aimed at improving reproductive health, ensuring safe motherhood, or directed at women in their role as carers for children aged less than five years. • Infectious diseases: any interventions aimed at preventing, diagnosing, or treating communicable diseases such as tuberculosis, malaria, and diarrhoeal diseases | The diversity of included studies limited meta-analysis to outcomes for four study groups. These analyses found evidence of moderate quality of the effectiveness of LHWs in promoting immunisation childhood uptake (RR 1.22, 95% CI 1.10 to 1.37; P = 0.0004); promoting initiation of breastfeeding (RR = 1.36, 95% CI 1.14 to 1.61; P < 0.00001), any breastfeeding (RR 1.24, 95% CI 1.10 to 1.39; P = 0.0004), and exclusive breastfeeding (RR 2.78, 95% CI 1.74 to 4.44; P <0.0001); and improving pulmonary TB cure rates (RR 1.22 (95% CI 1.13 to 1.31) P <0.0001), when compared to usual care. There was moderate quality evidence that LHW support had little or no effect on TB preventive treatment completion (RR 1.00, 95% CI 0.92 to 1.09; P = 0.99). There was also low quality evidence that LHWs may reduce child morbidity (RR 0.86, 95% CI 0.75 to 0.99; P = 0.03) and child (RR 0.75, 95% CI 0.55 to 1.03; P = 0.07) and neonatal (RR 0.76, 95% CI 0.57 to 1.02; P = 0.07) mortality, and increase the likelihood of seeking care for childhood illness (RR 1.33, 95% CI 0.86 to 2.05; P = 0.20). |
|  |  |  |  |  |  |  |  |  |  |  |  |
| Community-level service delivery interventions | Systematic Review | Lassi, Z 72 | Community-based intervention packages for reducing maternal and neonatal morbidity and mortality and improving neonatal outcomes. | 2010 | Cochrane | 18 cluster-randomised/quasi-randomised trials | (23 353 households, 12 391 total births) and control groups (23 768 households, 11 443 total births). | To assess the effectiveness of community-based intervention packages in reducing maternal and neonatal morbidity and mortality; and improving neonatal outcomes. | Women of reproductive age group, particularly pregnant women at any period of gestation. | Intervention packages that included additional training of outreach workers (residents from community who are trained and supervised to deliver maternal and newborn care interventions to her target population) namely, lady health workers/visitors, community midwives, community/village health workers, facilitators or TBAs in maternal care during pregnancy, delivery and in the postpartum period; and routine newborn care. Additional training was defined as training other than the usual training that health workers received from their governmental or non-governmental organisation and could include a combination of training in providing basic antenatal, natal and postnatal care; preventive essential newborn care, breastfeeding counselling; management and referral of sick newborns; skills development in behaviour change communication and community mobilisation strategies to promote birth and newborn care preparedness. The training sessions have been lectures, supervised hands-on training in a healthcare facility and/or within the community. The control group in these studies was the one that received their usual maternal and newborn care services from local government and non-government facilities. | No reduction in maternal mortality (risk ratio (RR) 0.77; 95% confidence interval (CI) 0.59 to 1.02, random-effects (10 studies, n = 144,956), I² 39%, P value 0.10. However, significant reduction was observed in maternal morbidity (RR 0.75; 95% CI 0.61 to 0.92, random-effects (four studies, n = 138,290), I² 28%; neonatal mortality (RR 0.76; 95% CI 0.68 to 0.84, random-effects (12 studies, n = 136,425), I² 69%, P value < 0.001), stillbirths (RR 0.84; 95% CI 0.74 to 0.97, random-effects (11studies, n = 113,821), I² 66%, P value 0.001) and perinatal mortality (RR 0.80; 95% CI 0.71 to 0.91, random-effects (10 studies, n = 110,291), I² 82%, P value < 0.001) as a consequence of implementation of community-based interventional care packages. It also increased the referrals to health facility for pregnancy related complication by 40% (RR 1.40; 95% CI 1.19 to 1.65, fixed-effect (two studies, n = 22,800), I² 0%, P value 0.76), and improved the rates of early breastfeeding by 94% (RR 1.94; 95% CI 1.56 to 2.42 |
| Community-level service delivery interventions | Systematic Review | Kidney,E 73 | Systematic review of effect of community-level interventions to reduce maternal mortality. | 2009 | BMC Pregnancy Childbirth | 13 | Not specified in article) | Provide a systematic review of the effectiveness of community level interventions to reduce maternal mortality. | General maternity populations or women of childbearing age (15 to 49 years) taking part in a community-level intervention | • Nepal: Facilitator-led women's groups to improve perinatal care practices plus health-service strengthening vs. usual care plus health-service strengthening.  • Pakistan: TBA training and health service integration, issue of sterile delivery kits vs. usual care • Zimbabwe x2 Multi-centre study: Fewer, but goal oriented antenatal visits vs. standard "westernised" antenatal care • Switzerland: Women opting for home vs. hospital birth in "westernised" setting • Senegal: Women in Kaolack delivered mainly by TBAs in district birth centres vs. women in St Louis delivered mainly by midwives in hospital • Gambia: TBA training, village health worker support and obstetric pack vs. no additional care • Bangladesh: Midwives working with community health workers and TBAs to attend home births, manage obstetric complications and accompany referral cases to project clinic vs. routine care (not described) plus access to project clinic • Bangladesh: Access to above Matlab Intervention (refer to Rahman – at the end of this document for details) • Gambia: Early identification of pregnant women by trained TBAs, mobile antenatal unit to treat anaemia and infections; referral/transfer for obstetric emergency treatment; low cost insurance scheme to pay for treatment vs. care by TBAs with minimal tertiary facilities • China: Reorganisation of maternity care to include better clinical governance, education and training of staff, and some community education • China: Maternal and child health providers at grass roots level given two weeks theory training; some also given one month clinical skills training | Improved perinatal care practices for maternal mortality 3 trials OR = 0.62 (0.39, 0.98)  Minimal goal-oriented antenatal care models for maternal mortality OR = 1.09 (0.53, 2.25) No difference |
| Diarrhoea prevention | Meta-analysis | Curtis, V 75 | Effect of washing hands with soap on diarrhoea risk in the community: a systematic review. | 2003 | Lancet Infect Dis | 17 studies (7-intervention, 10 observational) | Not specified in article) | to determine the impact of washing hands with soap on the risk of diarrhoeal diseases in the community | child carers, children, adults in the community | Soap and handwashing with or without lessons. Three studies were set in childcare facilities, whereas the others reported domestic handwashing practice. Many different types of, and occasions for, handwashing were recorded including washing by child carers, by children, and by adult study respondents. Handwash occasions reported included: after defecation or after the toilet, after cleaning up a child or handling nappies, before eating, and before preparing or handling food. One study used the presence of soap in the home as an indicator of handwashing. Two studies did not specify whether soap was used for hand washing or not | Risk estimates for all studies, irrespective of design, with their 95% CIs. The summary risk estimate was 1.74 (95% CI 1.39–2.18), giving an equivalent reduction in risk of 43% (28–54%). The pooled summary estimate was 1.88, equivalent to a 47% reduction in diarrhoea risk associated with handwashing (24–63%). severe outcomes were combined, handwashing was found to be associated with a 48% reduction in severe enteric infections (35–66%) and a 59% reduction in shigellosis (two studies only, 95% CI 38–73%). |
|  |  |  |  |  |  |  |  |  |  |  |  |
| Diarrhoea prevention | Meta-analyses | Clasen, T 76 | Interventions to improve water quality for preventing diarrhoea: a systematic review and meta-analysis. | 2007 | BMJ | 33 studies (22 randomised controlled trials, 11 Quasi-randomised controlled trials) | 55 650 participants | To assess the effectiveness of interventions to improve the microbial quality of drinking water for preventing diarrhoea. | participants of all ages, 10 studies included only under 5s | The interventions to improve drinking water quality were undertaken at the level of either the water source (seven trials) or the household (35 trials). Water source interventions included protected wells, bore holes, or distribution to public tap stands; none included piped in (reticulated) household connections. Household interventions comprised improved water storage (one trial) or one of four approaches for treating water in the home: chlorination (16 trials), solar disinfection (three trials), filtration (eight trials), or combined flocculation and disinfection (seven trials). Apart from solar disinfection and flocculation-disinfection using a water purifying product (PUR sachet; Procter and Gamble), there were potentially important differences in the types of interventions. For example, filtration interventions varied by filter medium and pore size, and chlorination varied by chlorine source, dose, and contact time. Improvements in water quality were often accompanied by other environmental interventions intended to improved water storage in the home, and instruction on basic hygiene regarding contaminated water and diarrhoeal disease. One study included the introduction of oral rehydration therapy However, 14 trials consisted solely of water quality interventions, although ceramic filters and solar disinfection interventions may also improve storage. | Effectiveness: All - 12 studies Rate Ratios= 0.75 (0.65 to 0.87)  All -8 studies Risk Ratios = 0.50 (0.41 to 0.61) 10 - studies Odds Ratios = 0.65 (0.56 to 0.76) Under 5’s 8 studies Rate Ratios = 0.81 (0.69 to 0.95) 6 studies Risk ratios = 0.61 (0.48 to 0.77) 7 studies Odds Ratio = 0.65 (0.46 to 0.91) Effectiveness: water source level Not significant Effectiveness: Household source level all age groups (0.70, 0.56 to 0.88; nine trials) and for under 5s (0.76, 0.66 to 0.88; nine trials). Type of household intervention 8 studies Rate Ratios = 0.62 (0.47 to 0.82) 7 studies Rate Ratios = 0.49 (0.36 to 0.65) 10 studies Odds Ratios = 0.65 (0.56 to 0.76) Under 5s 5 studies Rate Ratios = 0.70 (0.54 to 0.89) Risk Ratios = 6 0.61 (0.48 to 0.77) Odds Ratios = 0.65 (0.46 to 0.91) Types: Chlorination 4 studies Rate Ratios = 0.61 (0.46 to 0.81) 4 studies Under 5s Rate Ratios = 0.41 (0.26 to 0.65) Filtration Under5s 3 Studies Rate Ratios = 0.60 (0.41 to 0.87)  2 studies Risk Ratios = 0.41 (0.21 to 0.79)  Under 5’s 2 studies  Risk Ratios = 0.41 (0.21 to 0.79) 3 studies Odds Ratio = 0.37 (0.27 to 0.49)  Under 5s 3 studies  Odds Ratio = 0.37 (0.22 to 0.62) Solar disinfectant 2 studies Odds Ratio = 0.69 (0.63 to 0.74) Flocculation disinfection 2 studies Odds ratio - 0.77 (0.65 to 0.90) |

**Appendix 4 -** Table of study characteristics

| **Author** | **Year** | **Title** | **Objective(s)** | **Participants (characteristics/total number)** | **Setting / context (eg.setting of interest, acute care, PHC, community, particular geographic location)** | **Intervention (clear succinct details to be described, type of intervention, frequency +/- intensity)** | **Number of databases /sources searched (not sure if we can do this analysis)** | **Number of studies/ types of studies / country of origin of included studies** | **Appraisal instrument and rating (instrument / tool used to assess risk of bias, rigor, study quality)** | **Type of review / method of analysis (eg random effects MA/fixed effect MA etc)** |
| --- | --- | --- | --- | --- | --- | --- | --- | --- | --- | --- |
| **Pre-pregnancy** | | | | | | | | | | |
| Conde-Agudelo, A 27 | 2006 | Birth spacing and risk of adverse perinatal outcomes: a meta-analysis. | To examine the association between birth spacing and relative risk of adverse perinatal outcomes. | 11 091 659 pregnant women | Twenty studies were conducted in the United States. The remaining 47 were conducted in 61 countries from Latin America (22 countries), Asia (20 countries), Africa (11 countries), Europe (7 countries) and 1 Australia. | Birth spacing measurement (interval from one birth and the next consecutive birth) or interval between delivery and a following conception) and inquiry of outcomes: >6 months, 6-11 months, 12-17 months, 18-23 months, 24-59 months, ≤60 months | Six databases including MEDLINE (1966 to January 2006), EMBASE (1980 to January 2006), ECLA (1980 - Janury 2006), POPLINE (1980 - January 2006), CINAHL (1982-January 2006), and LILACS (1982- january 2006) were searched. Unpublished studies were sourced from relevant researchers in the field. | 67 studies (52 cohort / cross sectional + 15 case control) were included. Twenty sudies (30%) were conducted in the United States. The remaining 47 were conducted in 61 countries from Latin America (22 countries), Asia (20 countries), Africa (11 countries), Europe (7 countries) and 1 in Australia. | A checklist was used from the Meta-analysis of Observational studies in Epidemiology (MOOSE) group for reporting Systematic Reviews of observational studies. Study methodological quality was judged by the following 6 validated criteria: 1) pregnancy interval used (adequate if the study used interpregnancy interval; inadequate if the study used birth interval); 2) categorization of exposure (adequate if the study examined ≥4 categories of pregnancy intervals; inadequate if the study examined <4); 3) birth spacing measurement and inquiry of outcomes (adequate if birth spacing measurement and ascertainment of outcomes were made by medicals records or direct measurement; inadequate if not); 4) blinding of both birth spacing status and ascertainment of outcomes (adequate if assessment of both birth spacing status and outcomes was blinded; inadequate if not blinded or unreported); 5) loss to follow-up or exclusions (only for cohort and cross-sectional studies) (adequate if loss to follow-up or nonvalid exclusions; and 6) control for confounding factors (adequate if the study additionally controlled for ≥2 of 5 confounding factors [parity, outcome of the most recent recognized pregnancy, access to prenatal care, breastfeeding, and maternal nutritional status]; inadequate if additionally controlled for <2). Assessment of methodological quality of each study was carried out by 2 of the authors working independently. Differences of opinion were resolved through discussion. | Meta-regression analyses, random effects model and dose-response regression slopes were used to pool data from individual (observational) studies. |
| **Antenatal** | | | | | | | | | | |
| Dowswell, T 28 | 2010 | Alternative versus standard packages of antenatal care for low-risk pregnancy | To compare the effects of antenatal care programmes providing a reduced number of antenatal care visits for low-risk women with programmes providing the standard schedule of visits, and to assess the views of the care providers and the women receiving antenatal care. | Seven trials (more than 60,000 women). Pregnant women attending antenatal care clinics and considered (using criteria defined by the trialists) to be at low risk of developing complications during pregnancy and labour. Authors excluded studies that included primarily high-risk women. | High, low and middle income countries (Although not specifically outlined) | Provision of a schedule of reduced number of visits, with or without goal-oriented antenatal care, compared with a standard schedule of visits. | Authors searched the Cochrane Pregnancy and Childbirth Group’s Trials Register by contacting the Trials Search Co-ordinator (April 2010). The Cochrane Pregnancy and Childbirth Group’s Trials Register ismaintained by the Trials Search Co-ordinator and contains trials identified from: 1. quarterly searches of the Cochrane Central Register of Controlled Trials (CENTRAL); 2. weekly searches of MEDLINE; 3. handsearches of 30 journals and the proceedings of major conferences; 4. weekly current awareness alerts for a further 44 journals plus monthly BioMed Central email alerts. Journals and conference proceedings were also handsearched. Authors searched reference lists of retrieved papers and personal communications and contacted principal investigators of included trials to obtain data on the relevant outcomes which were not reported in the original publication. No language restrictions were applied. | 7 trials were included. Authors included RCTs and quasi-random studies, such as those based on alternate allocation or allocation by days of the week. Both cluster and individually randomised trials were included. | Two review authors independently assessed risk of bias for each study using the criteria outlined in the Cochrane Handbook for Systematic Reviews of Interventions. The following headings: (1) Sequence generation (checking for possible selection bias); (2) Allocation concealment (checking for possible selection bias); (3) Blinding (checking for possible performance bias) Given the nature of the interventions evaluated, blinding of either the care providers or the women receiving care was not generally feasible.Auhors have not formally assessed blinding, but have noted where there was partial blinding, e.g. of outcome assessors. (4) Incomplete outcome data (checking for possible attrition bias through withdrawals, dropouts, protocol deviations). (5) For the Selective reporting bias and other sources of bias, authors recorded any other concerns they had about bias. For example, where outcomes of interest were reported incompletely and so could not be used, where a study failed to include results of a key outcome that would have been expected to have been reported, or where there was baseline imbalance between groups. | Authors carried out statistical analysis using REVMAN. Authors used fixed-effect meta-analysis for combining data in the absence of heterogeneity. For those outcomes where there were moderate or high levels of heterogeneity, where clinically meaningful, authors used random-effects analysis and these results are presented as average treatment effects. For dichotomous data, results were presented as summary risk ratio (RR) with 95% confidence intervals. For continuous data, authors used the mean difference if outcomes were measured in the same way between trials. Authors used the standardised mean difference to combine trials that measured the same outcome, but using differentmethods. If there was evidence in the trials of abnormally distributed data, authors reported this. Authors included three trials in the review where the unit of randomisation was the ’clinic’ rather than the ’individual’. For those outcomes where both types of trials contributed data, authors conducted pooled analyses using the generic inverse variance method with subtotals by unit of randomisation. For the cluster-randomised trials,authors adjusted standard errors to take account of the design effect using the methods described in Gates 2005 and Higgins 2009. Authors used an estimate of the intracluster correlation co-efficient (ICC) derived from the trial or from another source. For most of the outcomes considered in the review authors used the ICCs from one of the included trials (WHO 2001) which have been published (Piaggio 2001). In the additional tables authors described the source of the ICC for each outcome, and carried out sensitivity analyses to investigate the effect of varying the ICC. |
| Haider, B 30 | 2011 | Effect of multiple micronutrient supplementation during pregnancy on maternal and birth outcomes | To evaluate the evidence of the impact of multiple micronutruient supplements during pregnancy, in comparison with standard iron-folate supplements, on specific maternal and pregnancy outcomes of relevance to the Lives Saved Tool (LiST) | Pregnant women (number of total participants not mentioned) | Low-middle income settings | Multiple micronutrients were defined as supplementation with at least 5 micronutrients including the UNIMMAP formulation (which consisted of 30 mg iron, 400 μg folic acid, 15 mg zinc, 2 mg copper, 65 μg selenium, 800 μg RE vitamin A, 1.4 mg vitamin B1, 1.4 mg vitamin B2, 18 mg niacin, 1.9 mg vitamin B6, 2.6 μg vitamin B12, 70 mg vitamin C, 5 μg vitamin D, 10 mg vitamin E and 150 μg iodine) or those with comparable composition. The supplements were compared to maternal iron-folate supplementation. | PubMed, the Cochrane Library, the WHO regional databases and hand search of bibliographies of relevant reviews. The search was done up to December 2009 | 17 studies comprising 14 trials were included. These trials were 8 RCTs and 6 were cluster RCTs. All studies were from low-/middle-income settings. | The CHERG adaptation of the Grading of Recommendations Assessment, Development and Evaluation (GRADE) technique was used based on three components: 1) the volume and consistency of the evidence; 2) the size of the effect, or risk ratio; and 3) the strength of the statistical evidence for an association between the intervention and outcome, as reflected by the p-value. The individual studies were also graded. Three categories of criteria were used to judge quality of individual study evidence in the meta-analysis: 1) study design; 2) study quality; 3) relevance to the objectives of the review. Four grades were given to individual studies: high, moderate, low or very low. A study received an initial score of high if it was randomized or a cluster randomized trial. The grade was decreased by 0.5 to 1 for each study design limitation. In addition, studies reporting an intent-to-treat analysis or with statistically significant strong levels of association (>80% reduction) received 0.5-1 grade increases. Any study with a final grade of very low was excluded on the basis of inadequate study quality. | The assessment of statistical heterogeneity among trials was done by visual inspection i.e. the overlap of the confidence intervals among the studies, and by the Chi square (P-value) of heterogeneity in the meta-analyses. A low P value (less than 0.10) or a large chi-squared statistic relative to its degree of freedom was considered as providing evidence of heterogeneity. TFor the I2 values, roughly an I2 greater than 50% was taken to represent substantial and high heterogeneity. In situations of substantial or high heterogeneity being present, causes were explored, sub-group analyses performed and random effects model was used and although, this random model is not a substitute for a thorough investigation of heterogeneity, it was primarily to take into account heterogeneity that could not be explained. Pooled estimates were generated by generic inverse variance method of meta-analysis using REVMAN software. For all cluster randomized trials, cluster adjusted estimates were used. Authors assumed that there was no interaction between other interventions and the effect of supplementation, since all interventions were randomized. Results are presented as risk ratios (RR) with corresponding 95% confidence intervals (CI). |
| Imdad, A 31 | 2011 | The effect of folic acid, protein energy and multiple micronutrient supplements in pregnancy on stillbirths | (1) To estimate the effectiveness of peri-conecptional folic acid supplementation in reducing neural tube defects (NTDs) related to stillbirths. (2) To estimate the effectiveness of balanced protein energy supplementation during pregnancy in reducing all-cause stillbirths. (3) To estimate the effectiveness of multiple micronutrient supplementation during pregnancy in reducing all-cause stillbirths. (this summary only includes the balanced protein energy and multiple micronutrients) | 2186 Pregnant women | Developing countries | Balanced protein energy supplementation was defined as nutritional supplementation during pregnancy in which proteins provided less than 25% of the total energy content. Multiple micronutrients were defined as supplementation with at least five micronutrients and were compared with iron folic acid supplementation alone | PubMed, Cochrane, WHO regional databases. Authors also reviewed the reference lists of identified articles, existing reviews and meta-analyses to identify studies that were not picked up in the main search. Authors were contacted for any additional data, if required. | 18 studies (7 evaluated the effect of peri-conceptional folic acid supplementation and 11 studies assessed fortification of food with folic acid). | All the studies were then graded according to the CHERG adaptation of the GRADE technique. Each study was allocated a quality score of ‘high’ ‘moderate’ ‘low’ or ‘very low’. This assessment was based on the methodological quality of the study and consistency of results compared to that of other selected studies. Any study getting a final score of ‘very low’ was excluded from the review. | Authors generated meta-analyses where data were available from more than one study. Dichotomous data were combined to get a pooled relative risk. In cases where data from all the studies were not available in dichotomous form and risk ratios were available, meta-analysis was performed byGeneric inverse variance (GIV) method. Fixed models were used for primary analysis and random models were used in case of significant heterogeneity in the pooled estimate. Results are presented as Mantel-Haenszel risk ratios (RR) and corresponding 95% confidence intervals (CI). |
| Kramer, M 32 | 2003 | Energy and protein intake in pregnancy | To assess the effects of advice to increase or reduce energy or protein intake, or of actual energy or protein supplementation or restriction, during pregnancy on energy and protein intakes, gestational weight gain, and the outcome of pregnancy. | Pregnant women. In 5 trials (1134 pregnant women); in 13 trials (4665 pregnant women); in 2 trials (529 pregnant women); in 3 trials (384 pregnant women) For the assessment of dietary restriction, pregnant women with either high pregnancy weight or high gestational weight gain. | Both location and setting was not described | For energy and protein supplementation, no minimum protein content was required; trials of non protein energy were therefore included. Types of supplements included those that were ’balanced’ energy/protein supplements (the protein provided less than 25% of the total energy content), high-protein supplements (the protein provided 25% of the total energy content), and isocaloric protein supplements (balanced supplements in which the protein replaced an equal quantity of non protein energy). | Cochrane Pregnancy and Childbirth Group’s Trials Register contains: (1) quarterly searches of the Cochrane Central Register of Controlled Trials (CENTRAL); (2) monthly searches of MEDLINE; (3) handsearches of 30 journals and the proceedings of major conferences; (4) weekly current awareness search of a further 37 journals. Authors contacted authors for additional data and did not apply any language restrictions. | 13 randomized and quasi-randomized controlled trials were included. No other information was found. | The authors assessed the validity of each study using the criteria outlined in the Cochrane Handbook. (1) Randomization and allocation concealment Authors assigned a quality score for each trial, using the following criteria: (A) adequate concealment of allocation: such as telephone randomization, consecutively numbered sealed opaque envelopes; (B) unclear whether adequate concealment of allocation: such as list or table used, sealed envelopes, or study does not report any concealment approach; (C) inadequate concealment of allocation: such as open list of random number tables, use of case record numbers, dates of birth or days of the week. (2) Attrition bias  The authors assessed completeness to follow up using the following criteria: (A) less than 5% loss of participants; (B) 5% to 9.9% of loss of participants; (C) 10% to 19.9% loss of participants; (D) more than 20% loss of participants. (3) Outcomes measurement bias  Authors assessed blinding using the following criteria: (A) blinding of participants (yes/no/unclear); (B) blinding of caregiver (yes/no/unclear); (C) blinding of outcome assessment (yes/no/unclear). | A statistical analysis using the REVMAN was used for a fixed-effect meta-analysis to combine data in the absence of significant heterogeneity if trials were sufficiently similar. If heterogeneity was found, authors conducted a random-effects meta-analysis. For dichotomous data, authors presented results as a summary relative risk with 95% confidence intervals. For continuous data, authors used the weighted mean difference when outcomes were measured in the same way between trials. |
| Pena-Rosas, J 33 | 2012 | Daily oral iron supplementation during pregnancy (Review) | To assess the effects of daily oral iron supplements for pregnant women, either alone or in conjunction with folic acid, or with other vitamins and minerals as a public health intervention. | Pregnant women of any gestational age and parity. (more than 27,402 women) | In the majority of these studies (52 studies, 86%), the intervention was delivered in hospital or community-based antenatal clinics usually by physicians or other healthcare professionals including midwives, dieticians or social workers. In eight of the studies the intervention was delivered by community workers, traditional birth attendants or village-based healthcare staff, and supplements were provided during visits to women’s homes or in local community settings. The supplements were provided by village-based traditional birth attendants in the study by Menendez 1994. In the Han 2011 trial village nurses made visits to women’s homes to deliver supplements and monitor women’s health. Community health or village workers were involved in delivering supplementation programmes in 6 other trials. | A range of interventions providing daily oral supplementation (e.g. tablets, capsules) containing iron alone, iron + folic acid or iron + other vitamins and minerals. The oral supplements forms included tablets or capsules. Tablets (soluble tablets, effervescent tablets, tablets for use in the mouth, and modified-release tablets) are solid dosage forms containing one or more active ingredients. They are obtained by single or multiple compression (in certain cases they are moulded) and may be uncoated or coated. Capsules are solid dosage forms with hard or soft shells, various shapes and sizes, that contain a single dose of one or more active ingredients. Capsules may be hard, soft, and modified-release capsules and are generally intended for oral administration. Where data were available authors planned to compare the following. 1. Any supplements containing iron versus same supplements without iron or no treatment/placebo (no iron or placebo). 2. Any supplements containing iron and folic acid versus same supplements without iron or folic acid (no iron + folic acid or placebo). 3. Supplementation with iron alone versus no treatment/ placebo. 4. Supplementation with iron + folic acid versus no treatment/ placebo. 5. Supplementation with iron + folic acid versus folic acid alone (without iron) supplementation. 6. Supplementation with iron + other vitamins and minerals supplementation versus same other vitamins and minerals (without iron) supplementation. 7. Supplementation with iron + folic acid + other vitamins and minerals versus folic acid + same other vitamins and minerals (without iron) supplementation. 8. Supplementation with iron + folic acid + other vitamins and minerals versus same other vitamins and minerals (without iron + folic acid) supplementation.  Interventions that combined daily oral iron or iron + folic acid supplementation with co-interventions such as education or other approaches were included only if the other co-interventions were the same in both the intervention and comparison groups. Studies examining supplemental iron alone or vitamins and minerals provided from supplementary food based interventions (i.e. interventions with multiple micronutrient powders, lipid based supplements, fortified complementary foods, and other fortified foods) were excluded. | Authors searched the Cochrane Pregnancy and Childbirth Group’s Trials Register by contacting the Trials Search Co-ordinator (2 July 2012). The Cochrane Pregnancy and Childbirth Group’s Trials Register is maintained by the Trials Search Co-ordinator and contains trials identified from: 1. monthly searches of the Cochrane Central Register of Controlled Trials (CENTRAL); 2. weekly searches of MEDLINE; 3. weekly searches of EMBASE; 4. handsearches of 30 journals and the proceedings of major conferences; 5. weekly current awareness alerts for a further 44 journals plus monthly BioMed Central email alerts. Details of the search strategies for CENTRAL, MEDLINE and EMBASE, the list of hand searched journals and conference proceedings, and the list of journals reviewed via the current awareness service can be found in the ‘Specialized Register’ section within the editorial information about the Cochrane Pregnancy and Childbirth Group. Trials identified through. Unpublished studies were also searched for. There were no language restrictions | 60 Randomised or quasi-randomised trials were found. For the meta-anlayses, forty-three trials, were included. Twenty five trials were in Europe with 12 trials in United Kingdom; two trials in Norway; two trials in Finland, two trials in Sweden, two trials in the Netherlands; one each in Denmark; Ireland; Belgium; France and Italy. Eleven trials were conducted in the Americas with eight trials conducted in the United States of America; one in Canada; one in Ecuador and one in Jamaica. Four trials were conducted in Africa with one trial in South Africa, one in Nigeria; one in Gambia and one in Niger Four trials were conducted in Iran. One trial was conducted in Hong Kong and five in China. Three trials were conducted in Australia. Seven trials were conducted in Asia with one trial each in Myanmar (Burma), Thailand, Nepal ,Vietnam, Philippines, South Korea and Indonesia Most included trials were published in the between years 2000- 2009 and 1980-1989. Two trials were published before 1950’s, three trials in the period 1950-1959, seven trials between 1960- 1969, eight trials between 1970-1979, 13 trials in the period 1980- 1989, nine trials between 1990-1999, 13 trials in the period 2000- 2009 and only five included trials have been published since 2010 to present. | Two review authors independently assessed risk of bias for each study using the criteria outlined in the Cochrane Handbook for Systematic Reviews of Interventions: (1) Sequence generation (checking for possible selection bias). Authors described for each included study the method used to generate the allocation sequence. They assessed the method as: • low risk of bias (any truly random process, e.g. random number table; computer random number generator); • high risk of bias (any non-random process, e.g. odd or even date of birth; hospital or clinic record number); or  • unclear. (2) Allocation concealment (checking for possible selection bias). Authors described for each included study the method used to conceal the allocation sequence and assessed whether intervention allocation could have been foreseen in advance of, or during recruitment, or changed after assignment. Authors assessed the methods as: • low risk of bias (e.g. telephone or central randomisation; consecutively numbered sealed opaque envelopes); • high risk of bias (open random allocation; unsealed or nonopaque envelopes, alternation; date of birth); • unclear. (3) Blinding (checking for possible performance bias). Authors described for each included study the methods used, if any, to blind study participants and personnel from knowledge of which intervention a participant received. Blinding was assessed separately for different outcomes or classes of outcomes and authors noted where there was partial blinding. Authors assessed the methods as: • low, high or unclear risk of bias for women; • low, high or unclear risk of bias for clinical staff; • low, high or unclear risk of bias for outcome assessors. Authors classified blinding “inadequate” if the blinding status of a trial was unclear or the trial was open.(4) Incomplete outcome data (checking for possible attrition bias through withdrawals, dropouts, protocol deviations). Authors assessed losses to follow-up and post-randomisation exclusions systematically for each trial. Authors described for each included study, and for each outcome or class of outcomes, the completeness of data including attrition and exclusions from the analysis. Authors noted whether attrition and exclusions were reported, the numbers included in the analysis at each stage (compared with the total randomised participants), reasons for attrition or exclusion where reported, and whether missing data were balanced across groups or were related to outcomes. Authors assessed methods as: • low risk of bias; • high risk of bias; or • unclear. Authors considered follow-up to be adequate if more than 80% of participants initially randomised in a trial were included in the analysis and any loss was balanced across groups, unclear if the percentage of initially randomised participants included in the analysis was unclear, and inadequate if less than 80% of those initially randomised were included in the analysis or if loss was imbalanced in different treatment groups. (5) Selective reporting bias Authors assessed the methods as: • low risk of bias (where it is clear that all of the study’s prespecified outcomes and all expected outcomes of interest to the review had been reported); • high risk of bias (where not all the study’s pre-specified outcomes had been reported; one or more reported primary outcomes were not pre-specified; outcomes of interest were reported incompletely and so could not be used; study failed to include results of a key outcome that would have been expected to have been reported); • unclear. (6) Other sources of bias Authors assessed whether each study was free of other problems that could put it at risk of bias. We have noted for each included study any important concerns we had about other possible sources of bias. Authors assessed whether each study was free of other problems that could put it at risk of bias: • low risk of further bias; • high risk of further bias; • unclear whether there is a risk of further bias. A sensitiviy analysis was done for overall risk of bias | For dichotomous data, results are presented as summary risk ratio (RR) with 95% confidence intervals (CI). For continuous data,the mean difference (MD) was used if outcomes were measured in the same way between trials. |
| Blencowe, H [34] | 2011 | Lives Saved Tool supplement detection and treatment of syphilis in pregnancy to reduce syphilis related stillbirths and neonatal mortality | To provide quantitative estimates of the effect of antenatal syphilis detection based on serological screening in pregnancy combined with treatment with at least 2.4 million units penicillin (at least a single dose of benzathine penicillin or the equivalent multiple dose schedule of shorter acting penicillin) on syphilis related stillbirths, neonatal mortality and morbidity related to maternal syphilis infection. | Pregnant women with active syphilis | Not mentioned | Serologic detection of syphilis in pregnant women and treatment of women with active syphilis with at least 2.4 million units penicillin given at least 28 days prior to delivery. The comparison group was pregnant women with active syphilis who did not receive at least 2.4 million units at least 28 days prior to delivery. | PubMed, EMBASE, Cochrane Libraries, and all World Health Organisation Regional Databases and included publications in any language. Last searches were done in December 2009. | 24 Observational studies were included | Each study was assessed for limitations and graded according to the CHERG adaptation of the GRADE technique. The evidence was summarised by outcome including a qualitative assessment of study quality and sources of bias adapted from the Cochrane review handbook. CHERG Rules for Evidence Review were applied to the collective evidence to provide an estimate for reduction in stillbirth and congenital syphilis related neonatal mortality. | Separate meta-analyses for the effect of penicillin on incidence of stillbirth, preterm delivery, congenital syphilis, and neonatal mortality. Meta-analyses were conducted with STATA version 10.0. Heterogeneity was assessed using I2 and the chi-squared test. When evidence of heterogeneity was present (p<0.10), a random effects model was used, otherwise a fixed effect was assumed. |
| Hofmeyr, G 41 | 2002 | Calcium supplementation during pregnancy for preventing hypertensive disorders and related problems | To assess the effects of calcium supplementation during pregnancy on hypertensive disorders of pregnancy and related maternal and child adverse outcomes. Subgroup analyses will test these effects on whether : (1)Women are at low/average risk of hypertensive disorders, or at high risk. (2) Women have low or adequate dietary calcium intake prior to trial entry. | Pregnant women (6894) regardless of the risk of hypertensive disorders of pregnancy. Women with diagnosed hypertensive disorders of pregnancy will be excluded. Prespecified subgroups to be compared are: (1) Women at low or average risk of hypertensive disorders of pregnancy (unselected). (2)Women at above average risk of hypertensive disorders of pregnancy. These will include women selected by the trial authors on the basis of an increased risk of hypertensive disorders of pregnancy (eg teenagers, women with previous pre-eclampsia, women with increased sensitivity to angiotensin II, women with pre-existing hypertension). (3) Women or populations with low baseline dietary calcium intke (as defined by trial authors, or if not defined, mean intake less than 900 mg per day). (4) Women or populations with adequate dietary calcium intake (as defined by trial authors, or if not defined, mean intake equal to or greater than 900 mg per day). No complete number of particpants as there are different number of trials per outcome | not mentioned | Supplementation with calcium fromat the latest 34 weeks of pregnancy; compared with placebo treatment. Studies with no placebo were excluded. The initial analysis was limited to supplementation with at least 1 g of calcium per day. | Authors searched the Cochrane Pregnancy and Childbirth Group trials register (September 2003). The Cochrane Pregnancy and Childbirth Group’s trials register is maintained by the Trials Search Co-ordinator and contains trials identified from: 1. quarterly searches of the Cochrane Central Register of Controlled Trials (CENTRAL); 2. monthly searches of MEDLINE; 3. handsearches of 30 journals and the proceedings of major conferences; 4. weekly current awareness search of a further 37 journals. There were also hand searches, conferences searches and contact made with trialist from the previous review that was conducted) | 11 studies were included. The type of studies were published, unpublished and ongoing trials that randomly allocated calcium supplementation during pregnancy to a participant or a placebo in the trial. | There is mention of quality assessment but not how it is done or with what form (s) . In a table of included studies tha is referred to in the Methodological Quality section, there is reference to concealment of allocation and whether the trial was placebo controlled. This was provided for each study. | Categorical data were compared using relative risks and their 95% confidence intervals. Statistical heterogeneity between trials was tested for using the I2 statistic, with values greater than 50% indicating significant heterogeneity. In the absence of significant heterogeneity, data were pooled using a fixed effects model. If there was significant heterogeneity, a random effects model was used and an attempt made to identify potential sources of heterogeneity based on subgroup analyses by risk of hypertensive disorders, baseline dietary calcium intake, trial quality and trial size. For continuous data, pooled estimates of effect size were calculated from a weighted average, with weight based on the inverse of the variance. Comparisons, outcomes and subgroups other than those prespecified in the protocol are identified as 'post hoc’ analyses. |
| Imdad, A 36 | 2011 | Role of calcium supplementation during pregnancy in reducing the risk of developing gestational hypertensive disorders: a meta analysis of studies from developing countries | To evaluate the preventive effect of calcium supplementation during pregnancy on gestational hypertensive disorders and related maternal and neonatal mortality in developing countries. | Pregnant women in developing countries. In three of the included studies, the participants were defined as being at a higher risk of developing hypertension in pregnancy (pregnant teenage girls, women with previous pre-eclampsia or women with positive roll over test). There is no total number of women in the study, but there are for a meta-analysis done per outcome. | No mention of specific setting, but studies are in a developing country | Supplementation of Calcium to pregnant women before 32 weeks of pregnancy and compared to control (placebo or no intervention). The starting period of calcium supplementation in all the included studies was before 20-32 weeks of gestation and continued till delivery.The dose of calcium ranged from 0.5 g/day to 2 g/day. | A comprehensive search of PubMed, Cochrane Library and WHO regional databases was carried out using different terms for calcium and gestational hypertensive disorders. Studies were considered for inclusion irrespective of language or status of publication. The date of last search was March 22, 2010. Additional studies were obtained through hand search of references from identified studies and previous reviews. | 10 trials were included. All the included studies were randomized controlled trials with comparison groups receiving a placebo in all except in two studies in which participants of comparison group were simply observed as controls. Five of the included studies were from Asia and four from South America. One large multicentre trial was conducted by World Health Organization (WHO) in Argentina, Egypt, India, Peru, South Africa and Vietnam | Individual studies were evaluated according to CHERG adaptation of GRADE technique. In this method of qualitative evaluation, all RCTs received an initial score of ‘high’ and an observational study as ‘low’. The study scores were adjusted depending on limitations of the study design. Trials with a final grade of ‘high’ or ‘moderate’ and ‘low grade’ were included in the analysis with exclusion of studies with a final grade of ‘very low’. | Pooled analyses were conducted where data were available from more than one study for an outcome. The results were presented as risk ratios (RR) and 95% confidence intervals (CIs). The assessment of statistical heterogeneity among trials was done by visual inspection i.e. the overlap of the confidence intervals among the studies, and by the Chi square (P-value) of heterogeneity in the meta-analyses. A low P value (less than 0.10) or a large chi-squared statistic relative to its degree of freedom was considered as providing evidence of heterogeneity. The I2 values greater than 50% were taken as substantial and high heterogeneity. In situations of substantial or high heterogeneity being present, causes were explored by sensitivity analysis and random effects model were used. All analyses were conducted using REVMAN software |
| Duley, L 37 | 2007 | Antiplatelet agents for preventing pre-eclampsia and its complications | To assess the effectiveness and safety of antiplatelet agents for women at risk of developing pre-eclampsia. | 37560 pregnant women considered to be at risk of developing pre-eclampsia. This included women with normal blood pressure and those with chronic hypertension, as well as women with pregnancy- induced or gestational hypertension. | No specific mention of setting, but the countries the studies came from were Australia, Austria, Brazil, China, Egypt, Canada, United Kingdom, United States, France, Belgium, Finland, Germany, India, Israel, Italy, Jamaica, Japan, Netherlands, Russia, South Africa, Spain, Tanzania | Comparisons of any antiplatelet agent (such as low-dose aspirin or dipyridamole) with either placebo or no antiplatelet agent. This was regardless of dose and duration of therapy or mode of administration, and irrespective of whether in combination with another agent. | Cochrane Pregnancy and Childbirth Group’s Trials Register (July 2006), the Cochrane Central Register of Controlled Trials (The Cochrane Library 2005, Issue 1), EMBASE (1994 to November 2005) and handsearched congress proceedings of the International and European Societies for the Study of Hypertension in Pregnancy | 59 randomised trials were included. They were from the following countries : Australia, Austria, Brazil, China, Egypt, Canada, United Kingdom, United States, France, Belgium, Finland, Germany, India, Israel, Italy, Jamaica, Japan, Netherlands, Russia, South Africa, Spain, Tanzania | The quality of each included trial was assessed independently done by at least two review authors using the criteria outlined in the Cochrane Handbook for Systematic Reviews of Interventions. Methods used for generation of the randomisation sequence and concealment of allocation are described for each trial, where possible. Each study was assessed for quality of the concealment of allocation, completeness of follow up and blinding using the following criteria. For allocation concealment:The authors allocated a grade to each trial on the basis of allocation concealment: (A) adequate; (B) unclear; or (C) clearly inadequate. Where the method of allocation concealment was unclear, there were attempts to contact study authors to provide further details. Trials with inadequate allocation concealment and trials with quasi-randomised designs, such as alternate allocation and use of record numbers were excluded. For completeness of follow up: (A) less than 3% of participants excluded; (B) 3% to 9.9% of participants excluded; (C) 10% to 19.9% of participants excluded. | Statistical analyses were performed using the REVMAN, with results presented as a summary of relative risk, risk difference and number needed to treat. For each of these, the 95% confidence interval is given in brackets. The I2 statistic was used to assess heterogeneity between trials. In the absence of significant heterogeneity, results were pooled using a fixed-effect model. If substantial heterogeneity was detected (I2 more than 50%), possible causes were explored and subgroup analyses for the main outcomes performed. Heterogeneity that is not explained by subgroup analyses may be modelled using random-effects analysis, where appropriate.Fixed effects and random effects analysis |
| Dennis, C 39 | 2010 | Psychosocial and psychological interventions for treating antenatal depression | The primary objective of this review is to assess the effects, onmothers and their families, of psychosocial and psychological interventions compared with usual antepartum care in the treatment of antenatal depression. | 38 women. Pregnant women identified with antenatal depression (variously defined). | Setting not specified, but trial fund hispanic women in America | Any form of standard or usual care compared to a variety of non-pharmaceutical interventions - including psychoeducational strategies, cognitive behavioural therapy, interpersonal psychotherapy, non-directive counselling, various supportive interactions, and tangible assistance - delivered via telephone, home or clinic visits, or individual or group sessions antenatally by a professional (nurse, midwife, childbirth educator, physician) or lay person (a specially trained woman from the community, a student). | Authors searched theCochrane Pregnancy and Childbirth Group’s Trials Register (September 2006), the Cochrane Collaboration Depression Anxiety and Neurosis Group’s Trials Registers (CCDANCTR-Studies and CCDANCTR-References) (July 2006), the Cochrane Central Register of Controlled Trials (The Cochrane Library 2006, Issue 3), MEDLINE (1966 to July 2006), EMBASE (1980 to July 2006) and CINAHL (1982 to July 2006). They also scanned secondary references and contacted experts in the field to identify other published or unpublished trials. | One randomised control trial from the USA including predominantly hispanic women | Authors assessed the validity of each study using the criteria outlined in the Cochrane Handbook for Systematic Reviews of Interventions (Higgins 2005).Methods used for generation of the randomisation sequence were described for each trial. For (1) Selection bias (randomisation and allocation concealment) authors assigned a quality score for each trial, using the following criteria: (A) adequate concealment of allocation: such as telephone randomisation, consecutively-numbered, sealed opaque envelopes; (B) unclear whether adequate concealment of allocation: such as list or table used, sealed envelopes, or study does not report any concealment approach; (C) inadequate concealment of allocation: such as open list of random-number tables, use of case record numbers, dates of birth or days of the week. For (2) Attrition bias (loss of participants, for example, withdrawals, dropouts, protocol deviations) authors assessed completeness to followup using the following criteria: (A) less than 5% loss of participants; (B) 5% to 9.9% loss of participants; (C) 10% to 19.9% loss of participants; (D) more than 20% loss of participants. For (3) Performance bias (blinding of participants, researchers and outcome assessment) authors assessed blinding using the following criteria: 1. blinding of participants (yes/no/unclear); 2. blinding of caregiver (yes/no/unclear); 3. blinding of outcome assessment (yes/no/unclear). Authors assigned a rating to each trial, compared results and discussed differences until we reach agreement. | Results are presented using relative risk for categorical data and weighted mean difference for continuous data. |
| Siegfried, N 40 | 2011 | Antiretrovirals for reducing the risk of mother-to-child transmission of HIV infection | To determine whether, and to what extent, antiretroviral regimens aimed at decreasing the risk of mother-to-child transmission of HIV infection achieve a clinically useful decrease in transmission risk, and what effect these interventions have on maternal and infant mortality and morbidity. | Pregnant women with HIV infection or infants born to mothers with HIV infection. The 25 trials included 18,901 participants with a median trial sample size of 627 ranging from 50 to 1,844 participants. Twenty-two trials randomised mothers (18 pre-natally and four in labour) and followed up their infants, and three trials randomised infants. | Not mentioned specifically. For geographic location - Most trials (N=20) were conducted in a single country, all in poor regions of the world: Thailand (N=5), South Africa (N=3), Kenya (N=3), Botswana (N=2), Zimbabwe (N=2), Malawi (N= 2), Cote d’Ivoire (N=1), Uganda (N=1) and Zambia (N=1). Of the five multinational trials, three were conducted across countries in Africa: theDITRAME trial was conducted in Cote d’Ivoire and in Burkina Faso; and the PETRA trial was conducted in South Africa, Tanzania and Uganda; and the Kesho Bora trial was conducted in five sites in Burkina Faso, Kenya and South Africa. Two multinational, multicentre trials were conducted in rich countries: the PACTG 076 trial in the USA and France, and the PACTG 316 in the USA, Puerto Rico, Europe, Brazil and the Bahamas. | Any antiretroviral regimen with the specific aim of decreasing the risk of mother-to-child transmission of HIV infection. 1. Antiretrovirals versus placebo (Breastefeeding and not breastfeeding) 2. Longer versus shorter regimens using the same antiretrovirals (Breastfeeding and not breastfeeding) 3. Antiretroviral regimens using different drugs and durations of treatment (Breastfeeding and not breastfeeding) 4. TRIPLE regimens versus other (Breastfeeding and not breastfeeding) 5. TRIPLE regimen versus TRIPLE regimen (Breastfeeding and not breastfeeding) | Authors sought to identify all relevant studies regardless of language or publication status by searching the Cochrane HIV/AIDS Review GroupTrialsRegister, TheCochrane Library,MEDLINE, EMBASE and AIDSearch and relevant conference abstracts. Authors also contacted research organizations and experts in the field for unpublished and ongoing studies. The original review search strategy was conducted in 2002 and updated in 2006 and again in 2009. (1) MEDLINE (1966 to date) via PubMed on 17 February 2004 and updated on 31 January 2005 and again on 9 February 2006. The searches conducted in 2005 and 2006 yielded 265 records in total of which we selected 29 for full article retrieval. (The search in 2004 yielded 276 records but no record was kept of the number of full articles retrieved for that search.) For this update authors searched MEDLINE via PubMed on 17 April 2009 using the strategy documented for the years 2007 to date. (2) EMBASE (2000 to date) on 5April 2004 and updated again on 31 January 2005 and 9 February 2006 using the PubMed strategy modified for EMBASE. (3) AIDSearch (1995 to date) on 31 January 2005 and again on 9 February 2006. (4) The Cochrane Library Controlled Trials Register on 7 April 2004 and again on 31 January 2005 and 9 February 2006. International AIDS conferences were also searched for trials. | There were 25 trials included that were either randomised controlled trials or trials not necessarily randomised. Most trials (N=20) were conducted in a single country, all in poor regions of the world: Thailand (N=5), South Africa (N=3), Kenya (N=3), Botswana (N=2), Zimbabwe (N=2), Malawi (N= 2), Cote d’Ivoire (N=1), Uganda (N=1) and Zambia (N=1). Of the five multinational trials, three were conducted across countries in Africa: theDITRAME trial was conducted in Cote d’Ivoire and in Burkina Faso; and the PETRA trial was conducted in South Africa, Tanzania and Uganda; and the Kesho Bora trial was conducted in five sites in Burkina Faso, Kenya and South Africa. Two multinational, multicentre trials were conducted in rich countries: the PACTG 076 trial in the USA and France, and the PACTG 316 in the USA, Puerto Rico, Europe, Brazil and the Bahamas. | The components of each included trial for risk of bias was assessed using a standard form from the Cochrane guidelines that assessed : Sequence generation, Allocation concealment, Blinding. Selective Reporting or Other forms of bias for each trial was not assessed. | Transmission rates of HIV (these included Cox regression, Kaplan-Meier, Turnbull and life-tables) were reported. Where these were not reported, authors estimated them directly using the published data. Several studies reported the efficacy of the intervention compared to the control. Efficacy, at a specific time, is defined as the preventive fraction in the exposed group compared to the reference group, which is the relative reduction in the proportion infected: 1-(Re/Rf ). This Re is the estimated cumulative rate of transmission in the experimental group and Rf is the estimated cumulative rate of transmission in the reference group. Authors reported the efficacies of the studies up to the specified time-points. For those studies where efficacy and hence confidence intervals were not reported, authors calculated the confidence intervals for the efficacy using the recommended methods (Wilson and Newcombe described in Altman 2005).For analysis of results which are not based on survival analyses e.g. stillbirths, authors present the relative risk for each trial outcome based on the number randomised, not the number analysed, and report the 95% confidence intervals as calculated by REVMAN. |
| **Childbirth** | | | | | | | | | | |
| Kenyon, S 42 | 2010 | Antibiotics for preterm rupture of membranes | To evaluate the immediate and long-term effects of administering antibiotics to women with PROM before 37 weeks, on maternal infectious morbidity, neonatal morbidity and mortality, and longer-term childhood development. | 6800 women and babies. The women were preterm (less than 37 weeks) with rupture of the membranes. | Not clear - but there are developed and developing countries | Comparison of: • any antibiotic versus placebo. Subgroup comparisons for the primary outcome as follows: • all penicillins (excluding co-amoxiclav) versus placebo; • beta lactam (including co-amoxiclav) antibiotics versus placebo;  • macrolide (including erythromycin) antibiotics versus placebo. Additional comparisons: • beta lactam (including co-amoxiclav) antibiotics coamoxiclav versus macrolide antibiotics (including erythromycin); • all penicillins (except co-amoxiclav) versus macrolide antibiotics (including erythromycin). • Antibiotic versus no antibiotic (including non-placebo controlled trials) - perinatal death only. ◦ Subgroup comparison of non-placebo controlled trials only. • Different treatment regimens of same antibiotic. | Cochrane Pregnancy and Childbirth Group’s Trials Register by contacting the Trials Search Co-ordinator (29 April 2010). The Cochrane Pregnancy and Childbirth Group’s Trials Register ismaintained by the Trials Search Co-ordinator and contains trials identified from: 1. quarterly searches of the Cochrane Central Register of Controlled Trials (CENTRAL); 2. weekly searches of MEDLINE; 3. handsearches of 30 journals and the proceedings of major conferences; 4. weekly current awareness alerts for a further 44 journals plus monthly BioMed Central email alerts. No language restrictions were applied.H3 | 22 trials were included. Study types were randomised controlled comparisons of antibiotic administration versus placebo, given to women with preterm rupture of membranes. Also included were comparisons of different antibiotics. For the unambiguous and important outcome of perinatal death alone, authors included trials in the review that were randomised but not placebo controlled. Trials that used inappropriate methods of randomisation were excluded. Where the method of randomisation was not specified in detail in the expectation that their inclusion in the review would encourage the authors to make available further information on the method of randomisation, they were included. Trials where non-randomised cohorts were amalgamated with randomised participants if the results of the randomised participants were not reported separately were excluded.Trials in which postrandomisation exclusions occurred provided there was no evidence that these occurred preferentially in one or other arm of the trials, were included. | Two review authors independently assessed risk of bias for each study using the criteria outlined in the Cochrane Handbook for Systematic Reviews of Interventions. Under the following headings: (1) Sequence generation (checking for possible selection bias), authors describe for each included study the method used to generate the allocation sequence in sufficient detail to allow an assessment of whether it should produce comparable groups. (2) Allocation concealment (checking for possible selection bias), authors describe for each included study the method used to conceal the allocation sequence in sufficient detail and determine whether intervention allocation could have been foreseen in advance of, or during recruitment, or changed after assignment. (3) Blinding (checking for possible performance bias), authors have described for each included study the methods used, if any, to blind study participants and personnel from knowledge of which intervention a participant received. Authors judged studies at low risk of bias if they were blinded, or if they judged that the lack of blinding could not have affected the results. Blinding was assessed separately for different outcomes or classes of outcomes. (4) Incomplete outcome data (checking for possible attrition bias through withdrawals, dropouts, protocol deviations), have been described for each included study, and for each outcome or class of outcomes, the completeness of data including attrition and exclusions from the analysis. (5) Selective reporting bias has been described for each included study how we investigated the possibility of selective outcome reporting bias and what was found. (6) Other sources of bias were described for each included study any important concerns about other possible sources of bias documented. (7) Overall risk of bias . Authors made explicit judgements aboutwhether studies are at high risk of bias, according to the criteria given in the Handbook (Higgins 2009). | For dichotomous data, authors present results as summary risk ratio with 95% confidence intervals. For continuous data, they used the mean difference if outcomes are measured in the same way between trials.They used the standardised mean difference to combine trials thatmeasure the same outcome, but use different methods. Authors have included cluster-randomised trials in the analyses along with individually randomised trials. Their sample sizes would have been adjusted using the methods described in the Handbook using an estimate of the intracluster correlation co-efficient (ICC) derived from the trial (if possible), or from another source. If ICCs from other sources are used, we would have reported this and conducted sensitivity analyses to investigate the effect of variation in the ICC. Statistical analysis using the REVMAN was conducted and a random-effects meta-analysis was done. |
| Cousens, S 43 | 2010 | Antibiotics for pre-term pre-labour rupture of membranes: prevention of neonatal deaths due to complications of pre-term birth and infection | To review the evidence for and estimate the effect on neonatal mortality due to pre-term birth complications or infection, of administration of antibiotics to women with pPROM, in low and middle-income countries. | 1 4581 newborns | Most trials were from high income countries except for three trials . One in Turkey (31 newborns), one trial in Mozambique (106 newborns) and one in Chile (85 newborns). | Women with pre-term rupture of membranes were treated with antibiotics. The antibiotic used, dose and duration varied between trials. | Systematic searches in multiple databases were done to identify studies of antibiotics for pPROM . Authors searched PubMed, Cochrane Libraries  and all World Health Organization Regional Databases, and included publications in any language. | 18 Randomised trials were included. Study types that were included were randomized trials from any setting from low and middle-income settings. Studies were included if antibiotics were given alone or in combination with antenatal steroids and surfactants. | The quality of the evidence provided by each of these studies was assessed using a standard table employing an adapted version of GRADE+I10 developed by the Child Health Epidemiology Reference Group (CHERG). | Statistical analyses to summarize results across studies were performed using Stata version 10 software. Authors performed and presented the results from fixed effects meta-analyses for those outcomes for which the evidence of heterogeneity between studies was weak (P>0.1). Authors performed and presented the results of random effects meta-analyses in others. |
| Brownfoot, F 44 | 2008 | Different corticosteroids and regimens for accelerating foetal lung maturation for women at risk of preterm birth. | To assess the effects on fetal and neonatal morbidity and mortality, on maternal morbidity and mortality, and on the child and adult in later life, of administering different types of corticosteroids (dexamethasone or betamethasone), different corticosteroid dose regimens, including timing, frequency and mode of administration fro women at risk of preterm birth | Women with a singleton or multiple pregnancy expected to give birth preterm (before 37 weeks) as a result of either spontaneous preterm labour, preterm prelabour rupture of membranes or elective preterm birth. 1089 women and 1161 infants | Hospitals | Different types of corticosteroids including dexamethasone, betamethasone, hydrocortisone or any other corticosteroid that can cross the placenta. Different corticosteroid regimens including dose, frequency, timing and route of administration. | The authors searched the Cochrane Pregnancy and Childbirth Group’s Trials Register by contacting the Trials Search Co-ordinator (January 2008). The Cochrane Pregnancy and Childbirth Group’s Trials Register is maintained by the Trials Search Co-ordinator and contains trials identified from: 1. quarterly searches of the Cochrane Central Register of Controlled Trials (CENTRAL); 2. weekly searches of MEDLINE; 3. handsearches of 30 journals and the proceedings of major conferences; 4. weekly current awareness alerts for a further 44 journals plus monthly BioMed Central email alerts. | 10 trials. Two were conducted in the USA , another two in France, two in Israel and one in each of Taiwan , UK , Netherlands and Poland. The trials were conducted over almost two decades from 1990 to 2005. | The validity of each study was assessed using the criteria outlined in the Cochrane Handbook for Systematic Reviews of Interventions. (1) Selection bias (randomisation and allocation concealment): Authors assigned codes using the following criteria: (A) adequate concealment of allocation: such as telephone randomisation, consecutively numbered sealed opaque envelopes; (B) unclear whether adequate concealment of allocation: such as list or table used, sealed envelopes, or study does not report any concealment approach; (C) inadequate concealment of allocation: such as open list of random-number tables, use of case record numbers, dates of birth or days of the week. (2) Attrition bias (loss of participants, e.g. withdrawals, dropouts, protocol deviations): Authors assessed completeness to follow up using the following criteria: (A) less than 5% loss of participants; (B) 5% to 9.9% loss of participants; (C) 10% to 19.9% loss of participants; (D) more than 20% loss of participants. (3) Performance bias (blinding of participants, researchers and outcome assessment): They have assessed blinding using the following criteria: (1) blinding of participants (yes/no/unclear); (2) blinding of caregiver (yes/no/unclear); (3) blinding of outcome assessment (yes/no/unclear). | Statistical analyses were performed using REVMAN. Authors used fixed-effect meta-analysis for combining data in the absence of substantial heterogeneity. On identifying high levels of heterogeneity among the trials (exceeding 50%), authors have explored outcomes by prespecified subgroup analysis. A random-effects meta-analysis was used as an overall summary as appropriate. For dichotomous data, authors presented results as risk ratio with 95% confidence intervals. For continuous data, they used the mean difference with 95% confidence intervals. |
| Mwansa-Kambafwile, J 45 | 2010 | Antenatal steroids in preterm labour for the prevention of neonatal deaths due to complications of preterm birth | To review the evidence globally for and estimate the effect on neonatal mortality due to preterm birth complications of antenatal steroid administration to women before anticipated preterm labour, compared with placebo or no treatment, with specific focus on variation on the effect size in low- and middle-income countries. | Neonates | Facility setting | Administration of corticosteroids to women in preterm labour. Antenatal steroids were given as therapy in premature labour and where birth occurred between 24 h and 7 days after treatment. All included studies incorporated a placebo or a suitable control group that was similar to the experimental group except that it did not receive antenatal steroids. | Cochrane Libraries, PubMed, LILACS, African Medicus, EMRO, all World Health Organization Regional Databases and publications in any language. Online searches of major conference proceedings were also conducted in order to identify unpublished literature. | 18 RCTs (14 in high-income countries and 4 in Brazil, South Africa, Tunisia and Jordan) There were 19 observational studies. All were high income countries except for two (one in Iran and the other in Brazil). | The authors assessed the quality of each of these studies using a standard approach developed by the Child Health Epidemiology Reference Group (CHERG) based on an adaptation of the GRADE approach. | Mantel–Haenszel pooled relative risk and corresponding 95% confidence interval, meta-analyses for RCT groups and observational study groups (High income countries, low middle income countries). |
| Duley, L 46 | 2010 | Magnesium sulphate versus phenytoin for eclampsia | To assess the effects of magnesium sulphate compared with phenytoin when used for the care of women with eclampsia. | 972 women. Women with a clinical diagnosis of eclampsia at randomisation irrespective of whether they were before or after delivery, had a singleton ormultiple pregnancy, or whether an anticonvulsant had been given before trial entry. | In a health setting | All randomised comparisons of magnesium sulphate (intravenous or intramuscular administration for the maintenance regimen) with phenytoin for women with eclampsia. As phenytoin is only used for prevention of further fits, another agent (usually a benzodiazapine) may have been used for control of the acute convulsion. | Authors searched the Cochrane Pregnancy and Childbirth Group’s Trials Register by contacting the Trials Search Co-ordinator (30 April 2010). The Cochrane Pregnancy and Childbirth Group’s Trials Register is maintained by the Trials Search Co-ordinator and contains trials identified from: 1. quarterly searches of the Cochrane Central Register of Controlled Trials (CENTRAL); 2. weekly searches of MEDLINE; 3. handsearches of 30 journals and the proceedings of major conferences; 4. weekly current awareness alerts for a further 44 journals plus monthly BioMed Central email alerts. No language restrictions were applied. | 7 trials. All adequately randomised trials comparing magnesium sulphate with phenytoin for treatment of women with eclampsia. There were 2 from the USA, two from South Africa, one from India, One from Brazil and one large Collaborative trial with 4 centres in South Africa and India. | At least two review authors independently assessed risk of bias for each study using the criteria outlined in the Cochrane Handbook for Systematic Reviews of Interventions. (1) Sequence generation (checking for possible selection bias). For each included study the method used to generate the allocation sequence in sufficient detail was described to allow an assessment of whether it should produce comparable groups. (2) Allocation concealment (checking for possible selection bias), authors described for each included study the method used to conceal the allocation sequence in sufficient detail and determine whether intervention allocation could have been foreseen in advance of, or during recruitment, or changed after assignment. (3) Blinding (checking for possible performance bias) authors described for each included study the methods used, if any, to blind study participants and personnel from knowledge of which intervention a participant received. Authors judged studies at low risk of bias if theywere blinded, or ifwe judged that the lack of blinding could not have affected the results.Blinding was assessed separately for different outcomes or classes of outcomes. (4) Incomplete outcome data (checking for possible attrition bias through withdrawals, dropouts, protocol deviations). Authors described for each included study, and for each outcome or class of outcomes, the completeness of data including attrition and exclusions from the analysis. (5) Selective reporting bias was described for each included study and the authors investigated the possibility of selective outcome reporting bias and what was found. (6) Other sources of bias were described for each included study with any important concerns authors had about other possible sources of bias. For example, if the trial stopped early due to some data-dependent process, or if there was an extreme baseline imbalance. Authors assessed whether each study was free of other problems that could put it at risk of bias and allocated a • yes; • no; • unclear. (7) Regarding the Overall risk of bias, authors made explicit judgements about whether studies were at high risk of bias, according to the criteria given in the Cochrane Hand-book for Systematic Reviews of Interventions | Authors performed statistical analyses using REVMAN with results presented as risk ratio and risk difference (RD). From1/RD authors calculated the number needed to treat for benefits, and for harmful or adverse effects. For each measure authors gave the 95% confidence intervals. Authors used the fixed-effect model for calculating risk ratio. If there was clear heterogeneity between the studies in any one outcome, they used a random-effects model |
| Hundley, V 47 | 2011 | Are birth kits a good idea? A systematic review of the evidence | To identify the current state of knowledge regarding the effects of births kits on clean birth practices and on newborn and maternal outcomes. | Traditonal birth attendants/ Skilled birth attendants/ SBA, Dayas (Traditonal birth attendants in Pakistan), Dais (traditional birth attendants in Egypt), All of these people were identified in the studies as using birth kits | The birth kit was primarily used in home settings. Although in some studies women delivered within a facility, only three compared birth kit use in home and facility settings. Geographic context: low-resource countries | The use of a birth kit defined as any disposable kit intended for routine use in the intrapartum period, specifically at the delivery of the baby | Electronic databases were searched from the starting date of the database to September 2009.The search strategy took into account the participants in low resource countries and the intervention (birthkit). The following electronic reference sources were searched: MEDLINE, EMBASE, CINAHL, POPLINE, MIDIRS, CENTRAL, BNI, AMED and Google Scholar. Databases of ongoing studies, such as www.ClinicalTrials.gov and www.who.int/trialsearch/, and ISI Web of Knowledge and ZETOC were searched to identify unpublished studies. The search was limited to literature published from 1987 onwards. A call for information was also distributed by email, mailbases and word of mouth to experts in maternal and child health, relevant research centres and specialist libraries. Information collected included published reports, grey literature and personal communication. | There were 12 papers with 9 studies included. Studies types were 5 Cross-sectional user/non-user comparisons, 2 Before andafter comparisons, 1 Contemporaneous controlled non-randomised trial, 1 Contemporaneous controlled randomised trial. The studies identified were all conducted within Africa or Asia. | Methodological quality was assessed and a simple quality score was applied. The scores, based on the Scottish Intercollegiate Guidelines Network (SIGN, 2008), reflect the researchers’ confidence that the study analysis was assessing a causal association. | Summary measures of Risk Ratios and Odds Ratios were reported on+Q7 |
| Yakoob, M 56 | 2011 | The effect of providing skilled birth attendance and emergency obstetric care in preventing stillbirths | To see the impact of skilled birth attendance (SBA) and the provision of Emergency Obstetric Care (EOC) on stillbirths and perinatal mortality. | Pregnant women | Not mentioned | Skilled birth attendance, and provision of basic and emergency obstetric care, for women with complications in pregnancy, childbirth or postpartum. The definition of a skilled attendant is “an accredited health professional – such as a midwife, doctor or nurse – who has been educated and trained to proficiency in the skills needed to manage normal (uncomplicated) pregnancies, childbirth and the immediate postnatal period, and in the identification, management and referral of complications in women and newborns | A systematic literature search was performed on PubMed/MEDLINE, Cochrane Database and the WHO regional libraries. A hand search of bibliographies of relevant reviews was also conducted. | 21 studies were included and used for data abstraction. Study types were randomized and quasi -randomized trials; (before-after) and observational studies. For the outcome of Skilled attendance at birth, there were 13 studies ( from Sudan, Bangladesh and Indonesia). For the outcome of Provision of emergency obstetric care there were 9 studies, mainly historical and ecologic studies that were in multiple countries. | A qualitative assessment of available evidence according to GRADE criteria and assessment of quantitative data based on rules developed by Child Health Epidemiology Review Group (CHERG). Individual studies were graded based on study design, quality of methods and relevance to study population (middle/lower income countries). Each study was assigned a quality grade of “high” “moderate” “low” or “very low” on the basis of strengths and limitations of the study. Any study with a final grade of ‘very low’ was excluded from the analysis. The grading of overall evidence was based on three components: (1) the volume and consistency of the evidence; (2) the size of the pooled effect and (3) the strength of the statistical evidence reflected in the p-value. A similar grading of ‘high’ ‘moderate’ ‘low’ and ‘very low’ was used for grading the overall evidence indicating the strength of an effect of the intervention on specific health outcome. | Meta-analyses were performed where data were available from more than one study for an outcome. The summary estimates were presented as relative risk (RR) or odd ratios (OR) with 95 % confidence interval (CI). Generic inverse method of meta-analysis was used to pool the data. The assessment of statistical heterogeneity among trials was done by visual inspection i.e. the overlap of the confidence intervals among the studies, and by the Chi square (P-value) of heterogeneity in the meta-analyses. A low P value (less than 0.10) or a large chi-squared statistic relative to its degree of freedom was considered as providing evidence of heterogeneity. The I2 values were also looked into and values greater than 50% were taken as substantial heterogeneity. In situations of substantial heterogeneity being present, causes were explored by sensitivity analysis and random effects model were used. Although random models are not a substitute for a thorough investigation of heterogeneity, it takes an ‘average’ effect from all the included studies compared to fixed models that take the exact contribution from the individual studies. All the analyses were performed using REVMAN. |
| Sibley, L 62 | 2007 | Traditional birth attendant training for improving health behaviours and pregnancy outcomes | To assess the effects of traditional birth attendant (TBA) training on TBA and maternal behaviours thought to mediate positive pregnancy outcomes, as well as on maternal, perinatal, and newborn mortality and morbidity. | Participants included traditional birth attendants (trained/ untrained TBAs) and lactating mothers living in the intervention and control areas, pregnant women living in the intervention and control clusters identified, recruited, and followed through the postpartum period, and women recently delivered or referred to a health facility by TBAs, or both. Over 2000 TBAs and nearly 27,000 women | Rural setting | Any TBA training intervention and comparison group data that are derived from trained and untrained TBAs (reference to target intervention), or mothers and neonates whose care is provided by trained and untrained TBAs, or who are living in areas where trained and untrained TBAs attend a majority of births | Authors searched the Cochrane Pregnancy and Childbirth Group’s Trials. Register by contacting the Trials Search Co-ordinator that is maintained by the Trials Search Co-ordinator and contains trials identified from: 1. quarterly searches of the Cochrane Central Register of Controlled Trials (CENTRAL); 2. weekly searches of MEDLINE; 3. handsearches of 30 journals and the proceedings of major conferences; 4. weekly current awareness alerts for a further 44 journals plus monthly BioMed Central email alerts.Cochrane Pregnancy and Childbirth Group’s Trials Register and the Cochrane Effective Practice and Organisation of Care Group’s (EPOC) Trials Register (June 2008). No language restrictions were applied. | 4 studies were included. There was one large cluster-randomized controlled trial from Pakistan, two smaller randomized controlled trials (one from Malawi and another from Bangladesh), and a controlled before/after study from Guatemala. | The authors assessed studies for methodological quality examining different sources of bias following criteria established by the Cochrane EPOC Group and summarizing risk of bias as outlined in the Cochrane Handbook for Systematic Reviews of Interventions. The Cochrane EPOC Group’s standard criteria for appraising the quality of studies include baseline measurement of outcomes, follow up of professionals, follow up of patients/episodes of care (protection against exclusion bias), blinded assessment of primary outcomes (protection against detection bias), reliable outcome measures, and protection against contamination. An additional criterion for randomized controlled trials and controlled clinical trials study includes concealment of allocation, while the analogous criterion for controlled before-after studies includes characteristics for studies using second site as control (protection against selection bias). To derive an overall summary assessment of how valid the results of each study are, authors used three categories in which a ’low risk’ of bias exists when all criteria are met (plausible bias unlikely to seriously alter results); a ’moderate risk’ of bias exists when one or more criteria are partially met (plausible bias raises some doubt about results); and a ’high risk’ of bias exists when one or more criteria were not met (plausible bias seriously weakens confidence results). | The authors used RevMan 2003 to calculate individual effect sizes of odds ratio and 95% confidence intervals or mean difference for the individual outcomes when possible. Descriptive statistics were used to report characteristics of the participants, interventions, and outcome measures detailed above. Due to the small number of studies, varied study designs and heterogeneity of the outcomes and measures, the authors were not able to calculate pooled summary effect sizes. |
| Darmstadt, G 58 | 2009 | 60 million non-facility births: Who can deliver in community settings to reduce intrapartum-related deaths? | To review the evidence for the effect of care by different community cadres (community-based attendants (SBAs), trained traditional birth attendants (TBAs) and community health workers (CHWs)) in improving perinatal and intrapartum-related outcomes during pregnancy and childbirth | Traditional birth attendants, community health workers, village health workers, community health aides, skilled birth attendant/skilled attendant, birthing centre, community midwives, Number of participants not mentioned. | Community | Care by different community cadres (community-based skilled birth attendants, trained traditional birth attendants, and community health workers) during pregnancy and childbirth | Searches of the following databases of the medical literature were conducted: PubMed, Popline, EMBASE, LILACS, IMEM, African Index Medicus, Cochrane, and World Health Organization (WHO) documents. The initial search was conducted in November 2002, and was updated May 2009 | For community-based skilled birth attendants 4 studies were used in meta-analyses for Perinatal mortality and 3 for Early neonatal mortality rate. For community health worker packages 4 studies were used in the Meta-analysis for Perinatal mortality and 3 for Early neonatal death. Studies were all from developing countries. | Modified GRADE criteria were used to evaluate the quality of the evidence (strong, moderate, low, or very low) and give a recommendation for programmatic application (strong, weak, conditional), as detailed in an earlier paper in this series. Authors used an adaptation of GRADE developed by the Child Health Epidemiology Reference Group (CHERG) specifically for low- and middle-income settings | Mortality reduction is reported as relative reduction unless other-wise reported. The authors conducted meta-analyses of studies evaluating packages of interventions provided by SBAsand CHWs using the Mantel-Haenszel (MH) pooled relative risk (RR) and corresponding 95% confidence interval (CI). When significant heterogeneity was detected (P<0.10), a random effects model was used to estimate the RR and CI. |
| **Neonatal** | | | | | | | | | | |
| Conde-Agudelo, A 59 | 2011 | Kangaroo mother care to reduce morbidity and mortality in low birthweight infants. | To determine whether there is evidence to support the use of KMC in LBW infants as an alternative to conventional neonatal care before or after the initial period of stabilization with conventional care. Beneficial and adverse effects were assessed. | 2518 LBW infants (defined as birthweight less than 2500 g) regardless of gestational age. | Hospital setting | 1. Comparisons of KMC with conventional neonatal care in LBW infants. This was regardless of duration of intervention, breastfeeding patterns, and irrespective of whether discharge from hospital was early or not. 2. Comparisons of early onset KMC (starting within 24 hours post-birth) with late onset KMC (starting after 24 hours post birth) in LBW infants, irrespective of infant stabilization status. | The standard search strategy of the Cochrane Neonatal Group was used. This included searches of MEDLINE, EMBASE, LILACS, POPLINE, CINAHL databases (from inception to January 31, 2011), and the Cochrane Central Register of Controlled Trials (The Cochrane Library, Issue 1, 2011). In addition, the authors searched the web page of the Kangaroo Foundation, conference and symposia proceedings on KMC, and Google scholar. | 16 studies were included and were randomised controlled trials and cluster randomised trials. The studies were from Low middle income countries (India, Ethiopia, Malaysia, Indonesia, Madagascar, Ecuador, Colombia, Mexico) and high income countries (United Stated, United Kingdom, Australia) | The risk of bias in each included trial was assessed individually by the two review authors who were not associated with any of the trials. Methodological assessments were not conducted blind to author, institution, journal of publication or results, as the reviewers were familiar with most of the studies. When differences in assessment of risk of bias existed, a consensus was reached. The authors assessed risk of bias using the dimensions outlined in the Cochrane Handbook for Systematic Reviews of Interventions. Five domains related to risk of bias were assessed in each included trial since there is evidence that these are associated with biased estimates of treatment effect: (1) sequence generation, (2) allocation concealment, (3) blinding of participants, clinical staff and outcome assessors, (4) incomplete outcome data, (5) selective outcome reporting, and (6) other potential threats to validity. Authors assigned a judgment relating to the risk of bias by answering a prespecified question about the adequacy of the study in relation to the entry, such that a judgment of “Yes” indicates low risk of bias, “No” indicates high risk of bias, and “Unclear” indicates unclear or unknown risk of bias. | Authors analyzed outcomes on an intention-to treat basis. If data for similar outcomes from two or more separate studies were available, they combined data in a meta-analysis and calculated a typical RR or MD with associated 95% CIs. |
| Moore, E 60 | 2012 | Early skin-to-skin contact for mothers and their healthy newborn infants. | To assess the effects of early skin-to-skin contact for healthy newborn infants compared to standard contact (infants held swaddled or dressed in their mothers arms, placed in open cribs or under radiant warmers). | Mothers and their healthy full term or late preterm newborn infants (34 to less than 37 completed weeks’ gestation) having early SSC starting less than 24 hours after birth, and controls undergoing standard patterns of care. 2177 mother-infant dyads. | Hospital setting | Early SSC for term or late preterm infants can be divided into several subcategories. (a) In ’birth SSC’, the infant is placed prone skin-to-skin on the mother’s abdomen or chest during the first minute postbirth. (b) In ’very early SSC’, beginning approximately 30 to 40 minutes post birth, the naked infant, with or without a cap, is placed prone on the mother’s bare chest. A blanket is placed across the infant’s back. (c) ’Early SSC’ can begin anytime between one and 24 hours post birth. The baby is naked (with or without a diaper and cap) and is placed prone on the mother’s bare chest between the breasts | Authors searched the Cochrane Pregnancy and Childbirth Group’s Trials Register by contacting the Trials Search Co-ordinator (30 November 2011). The Cochrane Pregnancy and Childbirth Group’s Trials Register is maintained by the Trials Search Co-ordinator and contains trials identified from: 1. quarterly searches of the Cochrane Central Register of Controlled Trials (CENTRAL); 2. weekly searches of MEDLINE; 3. weekly searches of EMBASE; 4. handsearches of 30 journals and the proceedings of major conferences; 5. weekly current awareness alerts for a further 44 journals plus monthly BioMed Central email alerts. | 34 studies were included. They were RCTs from Canada,Chile, Germany,Guatemala, Iran, Israel, Italy, Japan,Nepal, Poland,Russia, South Africa, Spain, Sweden, Taiwan, Thailand, the United Kingdom, and the United States. | Two review authors independently assessed risk of bias for each study using the criteria outlined in the Cochrane Handbook for Systematic Reviews of Interventions. They resolved any disagreement by discussion or by involving a third assessor. Authors used the electronic ’Risk of bias’ form in RevMan 2011 to describe study methodological quality. The following criteria were assessed: sequence generation, allocation concealment, blinding, incomplete outcome data, selective reporting bias, other sources of bias and overall risk of bias. | Fixed-effect meta-analysis for combining data when it was reasonable to assume that studies were estimating the same underlying treatment effect. If there was clinical heterogeneity sufficient to expect that the underlying treatment effects differed between trials, or if substantial statistical heterogeneity was detected, the authors used random-effects meta-analysis to produce an overall summary if an average treatment effect across trials was considered clinically meaningful. |
| Haider, B 62 | 2011 | Neonatal vitamin A supplementation for the prevention of mortality and morbidity in term neonates in developing countries | To evaluate the role of vitamin A supplementation in term neonates in developing countries with respect to the prevention of mortality and morbidity. | 51,446 neonates. All term neonates (born between 37 to 42 weeks of gestational age) up to 28 days after birth were included. | Most were in clincal settings and all from developing countries | Supplementation with vitamin A within the first 28 days of life was compared against a control (placebo or no supplementation). | Authors conducted the standard search strategy of the Cochrane Neonatal Review Group. The Cochrane Central Register of Controlled Trials (CENTRAL) (The Cochrane Library, 14 June 2010), EMBASE and MEDLINE (1966 to May 2010) via PubMed were searched using the following search terms: (Newborn OR infan* OR neonat*) AND (vitamin A OR retino*) Limit: publication type clinical trial. Authors limited the searches to human studies. They did not apply any language restrictions. They also searched related conference proceedings for relevant abstracts.Authors contacted organizations and researchers in the field for information on unpublished and ongoing trials.They searched reference lists of all trials identified by the above methods. | 7 trials were included and were all randomised controlled trials, both individual and cluster randomised. Some of these used a factorial design. Others were quasi-randomised trials. Studies were all from developing countires: Indonesion, Nepal, Bangladesh, Guinea Bissau, Zimbabwe, India, Pakistan, Ghana and Tanzania | The risk of bias for each study was done using the criteria outlined in the Cochrane Handbook for Systematic Reviews of Interventions (Higgins 2008). (1) Sequence generation (checking for possible selection bias). For each included study the method used to generate the allocation sequence in sufficient detail was described to allow an assessment of whether it should produce comparable groups. (2) Allocation concealment (checking for possible selection bias), authors described for each included study the method used to conceal the allocation sequence in sufficient detail and determine whether intervention allocation could have been foreseen in advance of, or during recruitment, or changed after assignment. (3) Blinding (checking for possible performance bias) authors described for each included study the methods used, if any, to blind study participants and personnel from knowledge of which intervention a participant received. Authors judged studies at low risk of bias if they were blinded, or ifwe judged that the lack of blinding could not have affected the results.Blinding was assessed separately for different outcomes or classes of outcomes. (4) Incomplete outcome data (checking for possible attrition bias through withdrawals, dropouts, protocol deviations). Authors described for each included study, and for each outcome or class of outcomes, the completeness of data including attrition and exclusions from the analysis. (5) Selective reporting bias was described for each included study and the authors investigated the possibility of selective outcome reporting bias and what was found. (6) Other sources of bias were described for each included study with any important concerns authors had about other possible sources of bias. For example, if the trial stopped early due to some data-dependent process, or if there was an extreme baseline imbalance. Authors assessed whether each study was free of other problems that could put it at risk of bias and allocated a • yes; • no; • unclear. (7) Regarding the Overall risk of bias, authors made explicit judgements about whether studies were at high risk of bias, according to the criteria given in the Cochrane Hand-book for Systematic Reviews of Interventions | Authors analysed the data using a generic inverse variance approach to meta-analysis, using REVMAN and generated risk ratio or rate ratio estimateswith 95% CIs for the dichotomous outcomes. For this approach, the data were entered as natural logarithms (as log risk ratios and SE of log risk ratio or log rate ratios and SE of log rate ratio) for each individual study, with data either extracted from the published papers or obtained from the authors if not presented in the papers. Authors used the fixed-effect method for combining data where trials were examining the same intervention and the trial populations and methods were judged to be sufficiently similar. |
| Imdad, A 69 | 2011 | Vitamin A supplementation for preventing morbidity and mortality in children from 6 months to 5 years of age | To evaluate the effect of vitamin A supplementation (VAS) for preventing morbidity and mortality in children aged 6 months to 5 years. | Children living in the community and aged 6 months to 5 years at the time of recruitment were eligible. Children in hospital and children with disease or infection were excluded. Authors contacted trial authors if the study population included some participants who were not eligible for this review (for example, children over 5 years) and requested disaggregated data. If such data were not available, authors included studies if the majority of participants (51%) met the inclusion criteria. If this could not be determined and the participants met the inclusion criteria on average (for example, the mean age was in the eligible range), then authors included the trials. Trials assigned approximately 215,633 participants, with sample sizes ranging between 35and approximately 29,236 and amedian sample size of 480. | not specified | Synthetic oral Vitamin A Supplementation (VAS) was compared to either placebo or treatmentas- usual control groups, including trials of various doses and frequencies. Co-interventions (for example,multiple vitamin ormineral supplementation), must have been identical in both groups. Authors excluded studies evaluating the effects of (i) food fortification, (ii) consumption of vitamin A rich foods and (iii) beta-carotene supplementation. If a trial included more than one eligible intervention group (for example, different doses), authors combined the groups for the main analysis, although the groups were treated separately for subgroup analyses where appropriate. If a trial included multiple control groups (for example, both placebo and treatment-as-usual), authors selected the control group that most closely replicated the nonspecific treatment of the intervention group (that is, placebo). | Authors searched CENTRAL (The Cochrane Library 2010, Issue 2), MEDLINE (1950 to April Week 2 2010), EMBASE (1980 to 2010 Week 16), Global Health (1973 to March 2010), Latin American Database (LILACS), metaRegister of Controlled Trials and African Index Medicus. All the searches were conducted on 27 April 2010. Authors also identified ongoing and unpublished trials, and used the World Health Organization International Clinical Trials Registry (ICTRP), which searches multiple trial registries. Reference lists of reviews, included studies and excluded studies were searched for additional citations. They contacted organisations and researchers. | Forty-three trials were included. They were Randomised controlled trials (RCTs) and cluster RCTs . | Two authors independently assessed the risk of bias associated with each included study using the Risk of Bias tool (Higgins 2008). For all studies, the following were assessed: sequence generation; allocation concealment; blinding of participants, providers and outcome assessors; incomplete outcome data; and selective outcome reporting.Authors specifically looked for the possibility of performance bias (differential treatment of the intervention and control groups) and detection bias (for example, differential effort to locate death records for the intervention and control groups). | Authors performed meta-analysis using REVMAN. When data were extracted in several formats that could not be combined directly in REVMAN, they used the generic inverse variance option; data were entered into Comprehensive Meta-Analysis Version 2 and the log RR and SE were entered into RevMan. |
| **Postnatal** | | | | | | | | | | |
| Howard, L | 2005 | Antidepressant prevention of postnatal depression | 1. To evaluate the effectiveness of antidepressant drugs in addition to standard clinical care in the prevention of postnatal depression. 2. To compare the effectiveness of different antidepressant drugs and with any other form of prevention for postnatal depression i.e. hormonal, psychological or social support. 3. To assess any adverse effects of antidepressant drugs in either the mother or the foetus/infant. | 73 participants (Women who were pregnant or had given birth in the last six weeks, who were not taking any antidepressant medication at the start of the trial). | Not mentioned | Any type of antidepressant medication at any dose alone or in combinationwith another treatment initiated in at least one arm of a trial compared with any other treatment, or placebo, or standard clinical care. | The Cochrane Collaboration Depression Anxiety and Neurosis review group trials registers (CCDAN TR-Studies and CCDANCTR- References). These registers are updated regularly adding the results on searches of TheCochrane Library,CINAHL, EMBASE, LILACS, MEDLINE, National Research Register, PSYCLIT, PSYCINFO, PSYNDEX and SIGLE. Also, quarterly systematic screening of relevant journals and conference proceedings takes place (for information on the fullCCDANsearch strategies, please see the CCDAN module). The original search of the CCDAN trials registers was carried out in July 2004 and an updating search was carried out in June 2007. Handsearches and personal comunication was carried out. | Two RCTs that were both from the USA | The methodological quality of the selected trials was assessed by three independent authors. Details of randomisation, concealment of allocation, blinding and exclusion analyses were recorded and evaluated. Unbiased methods of randomisation considered acceptable included random numbers generated by computer or sequentially numbered opaque sealed envelopes containing random allocation. A rating was assigned to each trial, based on the quality rating system developed by the Cochrane Collaboration Depression, Anxiety and Neurosis Group and the categories described in the Cochrane Handbook for Systematic Reviews of Interventions. Only categories A or B were to be included in meta-analyses. Data from other studies were to be described in the ’excluded studies’ tables. Where the three authors disagreed, the matter was discussed with the other authors until clear agreement was reached. | A meta-analysis could not be carried out because the two trials involved pharmacologically very different antidepressants |
| Penazzato, M | 2014 | Optimisation of antiretroviral therapy in HIV-infected children under 3 years of age | To evaluate (1) when to start ART in young children (less than 3 years); (2) what ART to start with, comparing first-line non-nucleoside reverse transcriptase inhibitor (NNRTI) and protease inhibitor (PI)-based regimens; and (3) whether alternative strategies should be used to optimize antiretroviral treatment in this population: induction (initiation with 4 drugs rather than 3 drugs) followed by maintenance ART, interruption of ART and substitution of PI with NNRTI drugs once virological suppression is achieved on a PI-based regimen | 3648 infants. perinatally HIV-infected children under 3 years of age | Clincial setting. All studies were in Africa | The following types of intervention were assessed: - Timing of treatment: use of early compared to deferred ART - Choice of treatment: use of NNRTI- versus PI-based regimens, in combination with any NRTI backbone - Substitution of LPV/r with NVP following initiation with PIbased regimens. - Interruption of treatment, compared to continuous early ART - Induction-maintenance treatment: Initiating ART with more than 3 antiretroviral drugs for an induction period, then moving to maintenance treatment with a standard 3-drug regimen | The search was performed in consultation with the HIV/AIDS Trials Search Co-ordinator. The original search was conducted on 1st November 2010 and was subsequently repeated on the 1st August 2012 for the purpose of this updated version of the review . Authors sought to identify all relevant studies, from 1997 to the search date, regardless of language or publication status, by searching the Cochrane HIV/AIDS Review Group Trials Register, The Cochrane Library, PubMed, EMBASE and the Cochrane Central Register of Controlled Trials (CENTRAL). In addition, the following specific search terms were used: infant, child, p(a)ediatric, highly active antiretroviral therapy, anti-retroviral agents, early antiretroviral therapy, deferred antiretroviral therapy,HIV infection, human immunodeficiency virus, acquired immunodeficiency syndrome, NNRTI, non-nucleoside reverse transcriptase inhibitors, NRTI, nucleoside reverse transcriptase inhibitors, PI, protease inhibitors, randomised controlled trial, and controlled clinical trial. Also, abstracts fromthe following relevant conference proceedings were screened for potentially eligible trials: World AIDS Conference; International AIDS Society conference (IAS) and Conference on Retroviruses and Opportunistic Infections (CROI), for abstracts presented through 2012. Authors also searched for unpublished and ongoing studies by considering prospective clinical trial registries (ClinicalTrials.gov and theWHO International Clinical TrialsRegistry Platform), and by contacting research organizations and experts in the field. | 8 trials were found. They were all randomised controlled trials. The numbers per intervention were found addressing when to start treatment (n=2), what to start (n=3), whether to substitute lopinavir/ritonavir (LPV/r) with nevirapine (NVP) (n=1), whether to use an induction-maintenance ART strategy (n=1) and whether to interrupt treatment (n=1) | Various aspects of the methodological quality of included studies were assessed independently by 2 authors using the risk of bias tool (Higgins 2011). Formal methods (as described by Egger in the Cochrane Handbook, (Higgins 2011) were planned to investigate the presence of reporting bias, but where few trials existed, the likelihood of reporting bias was instead described. | For meta-analysis of time-to-event outcomes, such as death and disease progression, the most appropriate statistic is the hazard ratio (HR). Where available, the HR and associated statistics were extracted directly from the trial report. When a HR was not provided in the trial report and only Kaplan-Meier curves were available, trial investigators were contacted to obtain the relevant HRs and related statistics. For dichotomous outcomes, such as decline by 10% in CD4%, a risk ratio (RR) of the rate of the occurrence was calculated from events and number of patients. For continuous outcomes, such as change in CD4 percentage or weight and height Z-score, the mean difference was calculated. Where more than one trial was identified for the questions being addressed, the HRs or RRs for each outcome for each trial were combined in meta-analysis to give a pooled HR or RR, using the fixed-effectmodel (FEM). A random-effectmodel (REM) was also used to test the robustness of the results to the choice of model. |
| **Across the first 1000days categories** |  |  |  |  |  |  |  |  |  |  |
| Lewin, S 71 | 2010 | Lay health workers in primary and community health care for maternal and child health and the management of infectious diseases | To assess the effects of lay health worker (LHW) interventions in primary and community health care on maternal and child health and the management of infectious diseases. | Any lay health worker (paid or voluntary) including community health workers, village health workers, birth attendants, peer counsellors, nutrition workers, home visitors. 3568 participants in total. | Studies were conducted in homes, primary care facilities, or a combination of home,primary care facilities and community-based interventions, by telephone or at community meetings. Others took place at workplaces, churches or homeless shelters. 55 studies (67%) were conducted in six high income countries: Australia, Canada, Ireland, New Zealand, the UK, and the USA. Forty-one of the 82 studies were conducted in the USA. Twelve studies (14.6%) were conducted in eight middle income countries (Brazil, China, India, Mexico, Philipines, Thailand, Turkey, and South Africa). Fifteen trials (18.3%) were from 10 low income countries (Bangladesh, Burkina Faso, Ethiopia, Ghana, Iraq, Jamaica, Nepal, Pakistan, Tanzania, and Vietnam). | Any intervention delivered by LHWs and intended to improve maternal or child health or the management of infectious diseases. Authors included interventions if the description was adequate for them to establish that it was a LHW intervention. Where such detail was unclear, they contacted study authors, whenever possible,to establish whether the personnel described were LHWs. For the purposes of this review, a MCH or infectious diseases intervention was defined as follows. Child health: any interventions aimed at improving the health of children aged less than five years. Maternal health: any interventions aimed at improving reproductive health, ensuring safe motherhood, or directed at women in their role as carers for children aged less than five years. Infectious diseases: any interventions aimed at preventing, diagnosing, or treating communicable diseases such as tuberculosis, malaria, and diarrhoeal diseases | Cochrane Central Register of Controlled Trials (CENTRAL) which includes citations uploaded from the EPOC and Cochrane Consumers and Communication Group Trial Registers (The Cochrane Library 2009, Issue 1) (searched 18 February 2009); MEDLINE, Ovid (1950 to February Week 1 2009, except August 2001 to December 2003 (searched 17 February 2009); • MEDLINE In-Process & Other Non-Indexed Citations, Ovid (February 13 2009) (searched 17 February 2009); EMBASE, Ovid (1980 to 2009 Week 05, except August 2001 to December 2003 (searched 18 February 2009); AMED, Ovid (1985 to February 2009) (searched 19 February 2009); British Nursing Index and Archive, Ovid (1985 to February 2009) (searched 17 February 2009); CINAHL, Ebsco (1982 to present) (searched 07 February 2010); POPLINE (searched 25 February 2009); WHOLIS (searched 16 April 2009). | 82 RCTs were included. 55 studies (67%) were conducted in six high income countries: Australia, Canada, Ireland, New Zealand, the UK, and the USA. Forty-one of the 82 studies were conducted in the USA. Twelve studies (14.6%) were conducted in eight middle income countries (Brazil, China, India, Mexico, Philipines, Thailand, Turkey, and South Africa). Fifteen trials (18.3%) were from 10 low income countries (Bangladesh, Burkina Faso, Ethiopia, Ghana, Iraq, Jamaica, Nepal, Pakistan, Tanzania, and Vietnam). | The authors used the approach recommended by The Cochrane Collaboration for assessing risk of bias in studies included in Cochrane reviews. Two review authors assessed independently the risk of bias of all included trials. They performed further analysis of the quality of evidence related to each of the key outcomes using the GRADE approach. Using this approach, theyrated the quality of the body of evidence for each key outcome as ’High’, ’Moderate’, ’Low’, or ’Very Low’. | Random-effects model meta-analysis |
| Lassi, Z 72 | 2010 | Community-based intervention packages for reducing maternal and neonatal morbidity and mortality and improving neonatal outcomes. | To assess the effectiveness of community-based intervention packages in reducing maternal and neonatal morbidity and mortality; and improving neonatal outcomes. | Women of reproductive age group, particularly pregnant women at any period of gestation. | Community setting. Five studies were conducted in India, five in Bangladesh, three in Pakistan, two in Gambia, one in Nepal, one in Indonesia, and one in Greece. | Intervention packages that included additional training of outreach workers (residents from community who are trained and supervised to deliver maternal and newborn care interventions to her target population) namely, lady health workers/visitors, community midwives, community/village health workers, facilitators or TBAs in maternal care during pregnancy, delivery and in the postpartum period; and routine newborn care. Additional training was defined as training other than the usual training that health workers received from their governmental or non-governmental organisation and could include a combination of training in providing basic antenatal, natal and postnatal care; preventive essential newborn care, breastfeeding counselling; management and referral of sick newborns; skills development in behaviour change communication and community mobilisation strategies to promote birth and newborn care preparedness. The training sessions have been lectures, supervised hands-on training in a healthcare facility and/or within the community. The control group in these studies was the one that received their usual maternal and newborn care services from local government and non-government facilities. | The authors contacted the Trials Search Co-ordinator to search the Cochrane Pregnancy and Childbirth Group’s Trials Register (January 2010). The Cochrane Pregnancy and Childbirth Group’s Trials Register is maintained by the Trials Search Co-ordinator and contains trials identified from: 1. quarterly searches of the Cochrane Central Register of Controlled Trials (CENTRAL); 2. weekly searches of MEDLINE; 3. Hand searches of 30 journals and the proceedings of major conferences; 4. weekly current awareness alerts for a further 44 journals plus monthly BioMed Central email alerts. In addition, the authors searched the World Bank’s JOLIS, British Library for Development Studies BLDS at IDS and IDEAS database of unpublished working papers, Google and Google Scholar. The authors carried out their search on January 12, 2010. | 18 Randomised and quasi-randomised controlled studies were included. Five studies were conducted in India, five in Bangladesh, three in Pakistan, two in Gambia, one in Nepal, one in Indonesia, and one in Greece. | Two review authors independently assessed risk of bias for each study using the criteria outlined in the Cochrane Handbook for Systematic Reviews of Interventions. Any disagreement was resolved by discussion. The following areas were reviewed: sequence generation, allocation concealment, blinding, incomplete outcome data, selective reporting bias, other sources of bias, and overall risk of bias. | Fixed-effect meta-analysis and random effects meta-analysis |
| Kidney, E 73 | 2009 | Systematic review of effect of community-level interventions to reduce maternal mortality | To provide a systematic review of the effectiveness of community level interventions to reduce maternal mortality. | General maternity populations or women of childbearing age (15 to 49 years) taking part in a community- level intervention | In a community setting, at a woman's home, village, school or local clinic, or delivered by any person within the community, and in a primary care setting | A"community-level" intervention was defined as either accessed locally at the woman's home, village, school or local clinic, or delivered by any person within the community, including health personnel or lay individual. Controls werre comparable populations experiencing either "usual care", including hospital based care, or other community interventions | Authors searched published papers using Medline, Embase, Cochrane library, CINAHL, BNI, CAB ABSTRACTS, IBSS, Web of Science, LILACS and African Index Medicus from inception or at least 1982 to June 2006; searched unpublished works using National Research Register website, metaRegister and the WHO International Trial Registry portal. Authors hand searched major references, contacted leading authors, and hand searched major relevant journals up to July 2007. They searched for unpublished work using the National Research Register website http://www.nrr.nhs.uk, 2006 issue 2, searched 30 June 2006), metaRegister http://www.controlled-trials.com/mrct/, searched 30 June 2006) and the WHO International Trial Registry portal http://www.who.int/trialsearch, searched 24 May 2007). No language restrictions were applied. | 13 studies were included. They were 5 cluster RCTs (1 from Nepal, Thailand, Pakistan and 2 from ZImbabwe), 1 Prospective cohort study with nested matched pairs from Switzerland, 1 Prospective survey of two cohorts from Senegal, 1 Cohort: Complex stratification of "randomly selected" project (from China) and matched non-project areas, 5 prospective cohorts (2 from Gambia, 2 bangladesh and 1 china) | Authors defined study quality as the extent to which design, methods, execution and analysis minimised bias in assessment of effectiveness, focusing on internal validity. They classified studies as high, medium, low (or unclear) quality with respect to selection, performance, measurement and attrition biases | Authors summarised results as odds ratios (OR) and confidence intervals (CI), combined using the Peto method for meta-analysis. |
| Curtis, V 75 | 2003 | Effect of washing hands with soap on diarrhoea risk in the community: a systematic review | To determine the impact of washing hands with soap on the risk of diarrhoeal diseases in the community | Adults and children | Three studies were set in childcare facilities, whereas the others reported domestic handwashing practice. Nine were done in urban settings, five in rural settings, one in both urban and rural settings, one in a refugee camp, and one did not specify the location. Ten studies were set in Asia, three in Africa, two in Latin America, one in the USA, and one in Australia. | Handwashing practices including soap use | Medline, CAB Abstracts, Embase, Web of Science, and the Cochrane Library were systematically searched using appropriate text words and thesaurus terms for papers relating to handwashing, use of soap, as well as disease terms such as diarrhoea, typhoid, enteric, cholera, shigellosis, dysentery, and mortality. Searches were also undertaken by hand with reference lists from these papers, the authors’ own collections, and review articles. | 17 studies were included. They were 7 intervention studies, 6 case-control, 2 cross-sectional, and 2 cohort studies. Ten studies were set in Asia, three in Africa, two in Latin America, one in the USA, and one in Australia. | Publication bias was explored via a funnel plot of all studies. | Random effects meta-analysis |
| Clasen, T 76 | 2007 | Interventions to improve water quality for preventing diarrhoea: a systematic review and meta-analysis. | To assess the effectiveness of interventions to improve the microbial quality of drinking water for preventing diarrhoea. | Adults or children in settings with endemic diarrhoeal disease ( 55 650 participants) | Rural and urban settings in 21 countries. The interventions to improve drinking water quality were undertaken at the level of either the water source (seven trials) or the household (35 trials). | Water source interventions included protected wells, bore holes, or distribution to public tap stands; none included piped in (reticulated) household connections. Household interventions comprised improved water storage (one trial) or one of four approaches for treating water in the home: chlorination (16 trials), solar disinfection (three trials), filtration (eight trials), or combined flocculation and disinfection (seven trials). | Cochrane Infectious Diseases Group’s trials register, CENTRAL, Medline, Embase, LILACS; hand searching; and correspondence with experts and relevant organisations | 33 studies were included. They were 22 randomised controlled trials, 11 quasi randomised controlled trials. All but two were in developing countries. The two were from urban areas in the USA | For randomised controlled trials authors extracted data on the methods used to generate the allocation sequence, allocation concealment, blinding of outcome assessment, and inclusion or losses to follow-up on the basis of criteria developed by Juni (Juni P, Altman DG, Egger M. Systematic reviews in health care: assessing the quality of controlled trials. BMJ 2001;323:42-6.) | Measures of effects reported were risk ratios, rate ratios, odds ratios, and longitudinal prevalence ratios (number of days or weeks with diarrhoea divided by number of days or weeks under observation in a person). As many of the trials were cluster randomised and had taken clustering into account in the data analysis (sometimes adjusting for covariates)authors could not use the reported data to recalculate a common measure and yet preserve such adjustments. They therefore present the results separately according to the reported measures of effect. Authors used a random effects inverse variance method on the log scale to calculate pooled estimates, and displayed the results graphically using RevMan. Heterogeneity was examined both visually by examining forest plots and statistically by using the χ2 test with a 10% level of statistical significance and the I2 test for consistency |

**Appendix 5 -** Table summary of included studies

|  | **SR without MA** | **SR with MA** | **SR/MA only RCT** | **SR/MA observational studies only** | **SR/MA mixed** | **Participants** | **Intervention and Comparator** | **Years of articles included** | **LIC** | **HIC** | **Neonatal and or child morbidity** | **Maternal morbidity** | **Neonatal and or child mortality** | **Maternal mortality** |
| --- | --- | --- | --- | --- | --- | --- | --- | --- | --- | --- | --- | --- | --- | --- |
|  |  |  |  |  |  |  |  |  |  |  |  |  |  |  |
| **Pre-pregnancy** | | | | | | | | | | | | | | |
| Conde-Agudelo A, 2006, JAMA30 |  | x |  | x |  | Women of childbearing age who have recently given birth | Birth spacing: interval from one birth and the next consecutive birth; interval between delivery and a following conception | 1973-2005 | x | x | x |  | x |  |
|  |  |  |  |  |  |  |  |  |  |  |  |  |  |  |
|  |  |  |  |  |  |  | Comparator: various time intervals (< 6 months; 6-11 months; 12-17 months; 18-23; 24-59 months; ≤60 months) |  |  |  |  |  |  |  |
|  |  |  |  |  |  |  |  |  |  |  |  |  |  |  |
|  |  |  |  |  |  |  |  |  |  |  |  |  |  |  |
|  |  |  |  |  |  |  |  |  |  |  |  |  |  |  |
| **Antenatal** | | | | | | | | | | | | | | |
| Dowswell T, 2010, 28 Cochrane Database |  | x | x |  |  | Pregnant women considered to be low-risk | Promotion of women receiving a minimum of 4 antenatal care visits (goal-orientated in LMIC) during each pregnancy, with a consideration of increasing the frequency in settings where perinatal mortality is high, the standard of care is poor and are considered to have high risk pregnancies. | 1995-2007 | x | x | x | x | x | x |
|  |  |  |  |  |  |  |  |  |  |  |  |  |  |  |
|  |  |  |  |  |  |  | Comparator: Usual care. In LMIC – to between 4-6 visits; HIC – from 13-14 visits to between 6-9 visits) |  |  |  |  |  |  |  |
| Haider B, 201130, BMC Public Health |  | x | x |  |  | Pregnant women | multiple micronutrient supplement formula called UNIMMAP (different constituents of same ingredients) Comparator: Usual care - iron-folate supplements | 2003-2009 | x |  | x | x | x |  |
| Imdad A, 201131, BMC Public Health |  | x |  |  | x | Pregnant women | Provision of folic acid supplementation, folate food fortification, MMN or balanced energy supplementation. | 1978-2009 | x |  | x |  | x |  |
|  |  |  |  |  |  |  | Comparator: usual care with folic acid alone |  |  |  |  |  |  |  |
|  |  |  |  |  |  |  |  |  |  |  |  |  |  |  |
|  |  |  |  |  |  |  |  |  |  |  |  |  |  |  |
| Kramer M, 2003, 32 Cochrane Database |  | x | x |  |  | Pregnant women | (1)Nutritional advice to increase energy and protein intake | 1938-2002 | x | x | x | x | x |  |
|  |  |  |  |  |  |  | (2)Balanced energy/protein supplementation (provides <25% total energy) |  |  |  |  |  |  |  |
|  |  |  |  |  |  |  | (3)High protein supplements (provides 25% total energy) |  |  |  |  |  |  |  |
|  |  |  |  |  |  |  | (4)Isocaloric protein supplements (protein replaced equal quantity of non-protein energy) |  |  |  |  |  |  |  |
|  |  |  |  |  |  |  | (5) Energy/protein restriction in women with overweight or high weight gain |  |  |  |  |  |  |  |
|  |  |  |  |  |  |  | Comparator: usual care |  |  |  |  |  |  |  |
| Peña-Rosas J,38 2012 Cochrane Database | x |  | x |  |  | Pregnant women | A range of interventions providing daily oral supplementation (e.g. tablets, capsules) containing iron alone, iron + folic acid or iron + other vitamins and minerals. | 1936-2011 | x | x | x | x | x | x |
|  |  |  |  |  |  |  | The oral supplements forms included tablets or capsules. Tablets (soluble tablets, effervescent tablets, tablets for use in the mouth, and modified-release tablets) are solid dosage forms containing one or more active ingredients. They are obtained by single or multiple compression (in certain cases they are moulded) and may be uncoated or coated. Capsules are solid dosage forms with hard or soft shells, various shapes and sizes, that contain a single dose of one or more active ingredients. Capsules may be hard, soft, and modified-release capsules and are generally intended for oral administration. Comparator: Daily oral supplements without iron / Supplements without iron + folic acid / No iron or placebo / No treatment / Daily folic acid alone (without iron) supplementation / other vitamins and minerals (without iron) supplementation |  |  |  |  |  |  |  |
| Blencowe H, 2011,34 BMC Public Health |  | x |  | x |  | Pregnant women | Physical examination, serological testing and treated with at least 2.4 million units of penicillin G | 1950-2009 | x |  | x |  | x |  |
|  |  |  |  |  |  |  | Comparator: Pregnant women with active syphilis who do not receive at least 2.4 million units at least 28 days prior to delivery. untreated infected women |  |  |  |  |  |  |  |
| Hofmeyr G, 2002, 35 Cochrane Database | x |  | x |  |  | Pregnant women | At least 1g/day of calcium | 1987-2001 | x | x | x | x | x | x |
|  |  |  |  |  |  |  | Comparator: Placebo |  |  |  |  |  |  |  |
| Imdad A, 201136, BMC Public Health |  | x | x |  |  | Pregnant women in LMIC settings | At least 2g/day of calcium | 1989-2009 | x |  | x | x | x | x |
|  |  |  |  |  |  |  | Comparator: |  |  |  |  |  |  |  |
|  |  |  |  |  |  |  | Placebo / observed as controls |  |  |  |  |  |  |  |
| Duley L, 2007, 37 Cochrane Database | x |  | x |  |  | Pregnant women with normal blood pressures, chronic hypertension, pregnancy-induced or gestational hypertension | Antiplatelet agents (primarily aspirin) Comparator: Placebo | 1982-2003 | x | x | x | x | x |  |
| Dennis C, 2007, 39 Cochrane Database | x |  | x |  |  | Impoverished women in HIC settings | 16, 45-minutes sessions of IPT over a 16-week period using cognitive behavioural therapy techniques | 2003 |  | x | x | x | x | x |
|  |  |  |  |  |  |  | Comparator: parenting education programme that consisted of 16 therapist-led weekly educational sessions for 45 minutes |  |  |  |  |  |  |  |
| Siegfried N, 2011, 40 Cochrane Database | x |  | x |  |  | Pregnant women | 1.Any antiretroviral regimen with the specific aim of decreasing the risk of mother-to-child transmission of HIV infection.2.Antiretrovirals versus placebo (Breastfeeding and not breastfeeding) 3. Longer versus shorter regimens using the same antiretrovirals (Breastfeeding and not breastfeeding) 4. Antiretroviral regimens using different drugs and durations of treatment (Breastfeeding and not breastfeeding) 5. TRIPLE regimens versus other (Breastfeeding and not breastfeeding) 6. TRIPLE regimen versus TRIPLE regimen (Breastfeeding and not breastfeeding) Comparator: Placebo / longer versus shorter regimens using the same antiretrovirals / regimens using different drugs and durations of treatment / triple regimens versus other/ triple antiretroviral regimens versus triple | 2001-2008 | x | x | x |  | x |  |
|  |  |  |  |  |  |  | Antiretroviral regimens |  |  |  |  |  |  |  |
| **Childbirth** |  |  |  |  |  |  |  |  |  |  |  |  |  |  |
| Kenyon S, 2010, 42 Cochrane Database | x |  | x |  |  | Pregnant women with PROM before 37 weeks and babies | Antibiotics: penicillin; beta-lactams; or macrolides. Erythromycin versus co- moxiclav. Use of surfactant. | 1988-2006 | x | x | x |  |  |  |
|  |  |  |  |  |  |  | Comparator: placebo |  |  |  |  |  |  |  |
| Cousens S, 2010, 43 Int J Epidemiol |  | x | x |  |  | Pregnant women with pPROM and newborns | Antibiotics: Women with pre-term rupture of membranes were treated with antibiotics: ampicillin, erythromycin, penicillin, or amoxicillin, mezlocillin, piperacillin, or combinations | 1988-2006 | x | x | x | x |  |  |
|  |  |  |  |  |  |  | Comparator: usual care |  |  |  |  |  |  |  |
| Brownfoot F, 2009,44 Cochrane Database |  | x | x |  |  | Women with a singleton or multiple pregnancy expected to give birth preterm (before 37 weeks) as a result of either spontaneous preterm labour, preterm prelabour rupture of membranes or elective preterm | Dexamethasone or betamethasone (various regimens, different timings and administration routes) | 1995-2007 | x | x | X |  |  |  |
|  |  |  |  |  |  |  | Comparator: Dexamethasone or betamethasone (various regimens, different timings and administration routes) |  |  |  |  |  |  |  |
| Mwansa-Kambofile J, 2010, 45 Int J Epidemiol |  | x |  |  | x | Women in preterm labour or at | Antenatal steroids administration of corticosteroids to women in preterm labour | 1972-2002 | x | x | x |  | x |  |
|  |  |  |  |  |  | high risk of preterm labour | Comparator: Placebo or no treatment |  |  |  |  |  |  |  |
| Duley L, 2010, 46 Cochrane Database | x |  | x |  |  | Pregnant Women | Magnesium sulphate (IV or IM) - 4 g as a loading dose, and maintenance therapy was either by an intramuscular regimen or an infusion of 1 g/hour | 1990-2003 | x | x | x | x | x | x |
|  |  |  |  |  |  |  | Comparator: phenytoin - a loading dose of 1000 to 1500mg as a short intravenous infusion, followed by a maintenance infusion of half the loading dose 12 hours later. As phenytoin is only used for prevention of further fits, another agent (usually a benzodiazepine) may have been used for control of the acute convulsion. |  |  |  |  |  |  |  |
| Hundley V, 2011,47 Midwifery | x |  |  |  |  | TBA /SBA/ Dais/ Dayas | The use of a birth kit defined as any disposable kit intended for routine use in the intrapartum period, specifically at the delivery of the baby primarily used at home | 1991-2009 | x |  | x |  | x |  |
|  |  |  |  |  |  |  | Comparator: Received usual care / no birth kits |  |  |  |  |  |  |  |
|  |  |  |  |  |  |  |  |  |  |  |  |  |  |  |
|  |  |  |  |  |  |  |  |  |  |  |  |  |  |  |
|  |  |  |  |  |  |  |  |  |  |  |  |  |  |  |
|  |  |  |  |  | x |  |  |  |  |  |  |  |  |  |
| Yakoob M, 2011, 56 BMC Public Health |  | x |  |  |  | Pregnant women who were delivering | SBA and/or emergency obstetric care Comparators: Either usual care or strengthening referral systems. No trained skilled attendants | 1979-2009 | x |  |  |  | X | x |
|  |  |  |  |  |  |  |  |  |  |  |  |  |  |  |
|  |  |  |  |  |  |  |  |  |  |  |  |  |  |  |
|  |  |  |  |  | x |  |  |  |  |  |  |  |  |  |
| Sibley L, 2007, 61 Cochrane Database | x |  |  |  | x | Trained/ untrained TBAs, lactating mothers, pregnant women followed through the postpartum period, and women recently delivered or referred to a health facility by TBAs, or both. | Trained TBAs on traditional birth attendance and maternal behaviours thought to mediate positive pregnancy outcomes. Specific training courses, over periods of time, supervision, continuing of education) | 1989-2005 | x |  |  | x | x | x |
|  |  |  |  |  |  |  | Comparator: no training or usual training |  |  |  |  |  |  |  |
| Darmstadt G, 2009, 58 Int J Gynaecol Obstet |  | x |  |  | x | TBAs, community health workers, village health workers, community health aides, SBA/skilled attendant, birthing centre, community midwives | Care by different community cadres (community-based skilled birth attendants, trained traditional birth attendants, and community health workers) during pregnancy and childbirth | 1990- 2009 | x |  | x | x | x |  |
| **Neonate** |  |  |  |  |  |  |  |  |  |  |  |  |  |  |
| Conde-Agudelo A, 2011, 63 Cochrane Database |  | x | x |  |  | Pregnant women and their low birth weight (<2500gm) infant regardless of gestational age | Comparisons of KMC with conventional neonatal care in LBW infants. Comparisons of early onset KMC (starting within 24 hours post-birth) with late onset KMC (starting after 24 hours post birth) in LBW infants | 1988-2010 | x | x | x |  | x |  |
|  |  |  |  |  |  |  | Comparator: conventional neonatal care, Late onset KMC |  |  |  |  |  |  |  |
| Moore E, 2012, 60 Cochrane Database |  | x | x |  |  | Mothers and their healthy full term or late preterm newborn infants | Early SSC (naked baby between breasts of mother) | 1976-2010 | x | x | x | x |  |  |
|  |  |  |  |  |  |  | Comparator: Routine care (swaddled infants, dressed and held in mother’s arms, placed in crib or under radiant warmers) |  |  |  |  |  |  |  |
| Imdad A, 2011, 69 Cochrane Database |  | x | x |  |  | Neonates at least 2500g without any serious medical condition | Oral dose of vitamin A (50,000 IU) within 24 hours of delivery or within the first 48 hours of delivery and the second dose within 24 hours of the first dose. Comparator: Placebo | 1976-2010 | x | x | x |  | x |  |
| Haider B, 2011, 62 Cochrane Database |  | x | x |  |  | Term neonates (born between 37 to 42 weeks of gestational age) up to 28 days after birth | Supplementation with vitamin A within the first 28 days of life | 1995-2010 | x |  | x |  | x |  |
|  |  |  |  |  |  |  | Comparator: control (placebo or no supplementation). |  |  |  |  |  |  |  |
| **Postnatal** |  |  |  |  |  |  |  |  |  |  |  |  |  |  |
| Howard L, 2005, 65 Cochrane Database | x |  | x |  |  | Women who were pregnant or had given birth in the last six weeks, who were not taking any antidepressant medication | Antidepressants: Nortyptiline or sertraline | 1999-2003 |  | x |  | x |  |  |
|  |  |  |  |  |  |  | Comparator: Any other treatment, or placebo, or standard clinical care |  |  |  |  |  |  |  |
| Penazzato M, 2014,66 Cochrane Database |  |  | x |  |  | Perinatally HIV-infected children under 3 years | 1. Timing of treatment: use of early compared to deferred ART | 2008-2013 | x |  | x |  | x |  |
|  |  |  |  |  |  |  | 2. Choice of treatment: use of NNRTI- versus PI-based regimens, in combination with any NRTI backbone |  |  |  |  |  |  |  |
|  |  |  |  |  |  |  | 3. Substitution of LPV/r with NVP following initiation with PI based regimens. |  |  |  |  |  |  |  |
|  |  |  |  |  |  |  | - Interruption of treatment, compared to continuous early ART |  |  |  |  |  |  |  |
|  |  |  |  |  |  |  | 4. Induction-maintenance treatment: Initiating ART with more than 3 antiretroviral drugs for an induction period, then moving to maintenance treatment with a standard 3-drug regimen |  |  |  |  |  |  |  |
|  |  |  |  |  |  |  | Comparators: NVP or EFV-based regimen / continuous PI based ART / |  |  |  |  |  |  |  |
| **Across the first 1000days categories** | | | | | | | | | | | | | | |
| Lewin S, 2010, 71 Cochrane Database |  | x | x |  |  | Mothers, neonates and children | LHWs (Paid or voluntary) – community health workers, village health corkers, birth attendants, peer counsellors, nutrition workers, home visitors | 1979-2009 | x | x | x | x | x |  |
|  |  |  |  |  |  |  | Comparator: usual care |  |  |  |  |  |  |  |
| Lassi Z, 2010, 72 Cochrane Database |  | x | x |  |  | Women of reproductive age group, particularly pregnant women at any period of gestation. | Additional MNCHW training (over and above usual training) of outreach workers in the form of lectures, supervised hands-on training in healthcare facility and/or within community | 1990-2010 | x |  |  | x | x | X |
|  |  |  |  |  |  |  | Training TBA who made home visits during antenatal period and delivery |  |  |  |  |  |  |  |
|  |  |  |  |  |  |  |  |  |  |  |  |  |  |  |
|  |  |  |  |  |  |  | Community based outreach workers |  |  |  |  |  |  |  |
|  |  |  |  |  |  |  |  |  |  |  |  |  |  |  |
|  |  |  |  |  |  |  | Building support through home visitations and community mobilization |  |  |  |  |  |  |  |
|  |  |  |  |  |  |  | Community mobilization and antenatal and post natal visits |  |  |  |  |  |  |  |
|  |  |  |  |  |  |  | Community support groups |  |  |  |  |  |  |  |
|  |  |  |  |  |  |  | Home-based care |  |  |  |  |  |  |  |
|  |  |  |  |  |  |  | TBAs made home visits antenatally and intrapartum |  |  |  |  |  |  |  |
|  |  |  |  |  |  |  | Comparator: Usual maternal and newborn care services from local government and non-government facilities. |  |  |  |  |  |  |  |
| Kidney E, 2009, 73 BMC Pregnancy and Childbirth |  | x |  |  | x | Pregnant women or women of childbearing age (15-49 years) participating in the community intervention | Interventions delivered in the community (home, school, local clinic; delivered by any person within the community (health personnel/lay person); primary care setting) | 1990-2007 | x | x |  |  |  | x |
|  |  |  |  |  |  |  | Comparator: |  |  |  |  |  |  |  |
|  |  |  |  |  |  |  | Minimal goal-oriented versus usual antenatal care |  |  |  |  |  |  |  |
|  |  |  |  |  |  |  | Comparator: Comparable populations experiencing either "usual care", including hospital based care, or other community interventions |  |  |  |  |  |  |  |
| Curtis V, 2003, 75 The Lancet |  | x |  |  | x | Adults including child carers and children | Using soap to wash hands in various settings in the community (urban, rural, childcare, refugee camp settings) Soap and hand washing with or without lessons. Studies were set in childcare facilities, or domestic hand washing practice. Many different types of, and occasions for, hand washing were recorded including washing by child carers, by children, and by adult study respondents. Hand wash occasions reported included: after defecation or after the toilet, after cleaning up a child or handling dirty nappies, before eating, and before preparing or handling food. In some studies soap was not specified. | 1981-2001 | x | x | x |  |  |  |
|  |  |  |  |  |  |  | Comparators: no hand washing |  |  |  |  |  |  |  |
|  |  |  |  |  |  |  |  |  |  |  |  |  |  |  |
|  |  |  |  |  |  |  |  |  |  |  |  |  |  |  |
|  |  |  |  |  |  |  |  |  |  |  |  |  |  |  |
|  |  |  |  |  |  |  |  |  |  |  |  |  |  |  |
|  |  |  |  |  |  |  |  |  |  |  |  |  |  |  |
|  |  |  |  |  |  |  |  |  |  |  |  |  |  |  |
|  |  |  |  |  |  |  |  |  |  |  |  |  |  |  |
|  |  |  |  |  |  |  |  |  |  |  |  |  |  |  |
|  |  |  |  |  |  |  |  |  |  |  |  |  |  |  |
|  |  |  |  |  |  |  |  |  |  |  |  |  |  |  |
|  |  |  |  |  |  |  |  |  |  |  |  |  |  |  |
|  |  |  |  |  |  |  |  |  |  |  |  |  |  |  |
|  |  |  |  |  |  |  |  |  |  |  |  |  |  |  |
|  |  |  |  |  |  |  |  |  |  |  |  |  |  |  |
|  |  |  |  |  |  |  |  |  |  |  |  |  |  |  |
| Clasen T, 2007, 76 BMJ |  | x | x |  |  | Residents living in the community (Adults or children in settings with endemic diarrhoeal disease) | Interventions to improve the microbial quality of drinking water for preventing diarrhoea at water source and; household (improved water storage, approaches for treating water in the, chlorination, solar disinfection, filtration, or combined flocculation and disinfection). | 1982-2006 | x | x | x |  |  |  |
|  |  |  |  |  |  |  | Comparator: Not clearly defined |  |  |  |  |  |  |  |
| **Total** | **10** | **21** | **22** | **2** | **7** |  |  |  | **30** | **22** | **27** | **16** | **22** | **10** |

**Appendix 6 -** Table showing Quality appraisal of studies using AMSTAR tool and PRISMA questions

| **Author** | **Year** | **Source** | **Title** | Was an 'a priori' design provided? | | Was there duplicate study selection and data extraction? | | Was a comprehensive literature search performed? | | Was the status of publication (i.e. grey literature) used as an inclusion criterion? | | Was a list of studies (included and excluded) provided? | | Were the characteristics of the included studies provided? | | Was the scientific quality of the included studies assessed and documented? | | Was the scientific quality of the included studies used appropriately in formulating conclusions? | | Were the methods used to combine the findings of studies appropriate? | | Was the likelihood of publication bias assessed? | | Was the conflict of interest included? | | Overall Score | * Were recommendations for policy and / or practice supported by the reported data? | **Were the specific directives for new research appropriate? |
| --- | --- | --- | --- | --- | --- | --- | --- | --- | --- | --- | --- | --- | --- | --- | --- | --- | --- | --- | --- | --- | --- | --- | --- | --- | --- | --- | --- | --- |
|  |  |  |  | Answer | Score | Answer | Score | Answer | Score | Answer | Score | Answer | Score | Answer | Score | Answer | Score | Answer | Score | Answer | Score | Answer | Score | Answer | Score |  |  |  |
| Adetifa | 2009 | Cochrane Database | Iron supplementation for reducing morbidity and mortality in children with HIV. | Yes | 1 | Yes | 1 | Yes | 1 | No | 0 | Yes | 1 | Yes | 1 | Yes | 1 | Yes | 1 | Yes | 1 | Yes | 1 | Yes | 1 | **10** |  |  |
| Alexander | 2010 | Cochrane Database | Repeat digital cervical assessment in pregnancy for identifying women at risk of preterm labour (Review) | Yes | 1 | No | 0 | Yes | 1 | No | 0 | No | 0 | Yes | 1 | Yes | 1 | Yes | 1 | Yes | 1 | Yes | 1 | Yes | 1 | **8** |  |  |
| Allen | 2010 | Cochrane Database | Probiotics for treating acute infectious diarrhoea | Yes | 1 | Yes | 1 | Yes | 1 | No | 0 | Yes | 1 | Yes | 1 | Yes | 1 | Yes | 1 | Yes | 1 | Yes | 1 | Yes | 1 | **10** |  |  |
| Aponte | 2013 | Cochrane Database | Probiotics for treating persistent diarrhoea in children | Yes | 1 | Yes | 1 | Yes | 1 | No | 0 | Yes | 1 | Yes | 1 | Yes | 1 | Yes | 1 | Yes | 1 | Yes | 1 | Yes | 1 | **10** |  |  |
| Askie | 2007 | Lancet | Antiplatelet agents for prevention of pre-eclampsia: a meta-analysis of individual patient data. | Yes | 1 | Yes | 1 | Yes | 1 | No | 0 | No | 0 | Yes | 1 | Yes | 1 | Yes | 1 | Yes | 1 | Yes | 1 | Yes | 1 | 9 |  |  |
| Austin | 2008 | Cochrane Database | Antenatal psychosocial assessment for reducing perinatal mental health morbidity | Yes | 1 | Yes | 1 | Yes | 1 | No | 0 | No | 0 | Yes | 1 | Yes | 1 | Yes | 1 | Yes | 1 | Yes | 1 | Yes | 1 | 9 |  |  |
| Bain | 2012 | Cochrane Database | Different magnesium sulphate regimens for neuroprotection of the fetus for women at risk of preterm birth | Yes | 1 | No | 0 | Yes | 1 | No | 0 | No | 0 | Yes | 1 | Yes | 1 | Yes | 1 | Yes | 1 | Yes | 1 | Yes | 1 | **8** |  |  |
| Bamigboye | 2009 | Cochrane Database | Oestrogen supplementation, mainly diethylstilbestrol, for preventing miscarriage | Yes | 1 | Yes | 1 | Yes | 1 | No | 0 | No | 0 | Yes | 1 | Yes | 1 | Yes | 1 | Yes | 1 | Yes | 1 | Yes | 1 | 9 |  |  |
| Barlow | 2010 | Cochrane Database | Group-based parent-training programmes for improving emotional and behavioural adjustment in children from birth to three years old | Yes | 1 | No | 0 | Yes | 1 | No | 0 | No | 0 | Yes | 1 | Yes | 1 | Yes | 1 | Yes | 1 | Yes | 1 | Yes | 1 | **8** |  |  |
| Begley | 2011 | Cochrane Database | Active versus expectant management for women in the third stage of labour. | Yes | 1 | No | 0 | Yes | 1 | No | 0 | No | 0 | Yes | 1 | Yes | 1 | Yes | 1 | Yes | 1 | Yes | 1 | Yes | 1 | **8** |  |  |
| Bennet | 2013 | Cochrane Database | Massage for promoting mental and physical health in typically developing infants under the age of six months | Yes | 1 | Yes | 1 | Yes | 1 | No | 0 | No | 0 | Yes | 1 | Yes | 1 | Yes | 1 | Yes | 1 | Yes | 1 | Yes | 1 | 9 |  |  |
| Bernaola | 2013 | Cochrane Database | Probiotics for trating persistent diarrhoea in children | Yes | 1 | Yes | 1 | Yes | 1 | No | 0 | No | 0 | Yes | 1 | Yes | 1 | Yes | 1 | Yes | 1 | Yes | 1 | Yes | 1 | 9 |  |  |
| Bessell | 2011 | Cochrane Database | Feeding interventions for growth and development in infants with cleft lip, cleft palate or cleft lip and palate | Yes | 1 | Yes | 1 | Yes | 1 | No | 0 | No | 0 | Yes | 1 | Yes | 1 | Yes | 1 | Yes | 1 | Yes | 1 | Yes | 1 | 9 |  |  |
| Bhutta | 2011 | BMC Public Health | Neonatal resuscitation and immediate newborn assessment and stimulation for the prevention of neonatal deaths: a systematic review, meta-analysis and Delphi estimation of mortality effect. | Yes | 1 | Yes | 1 | Yes | 1 | No | 0 | No | 0 | Yes | 1 | Yes | 1 | Yes | 1 | Yes | 1 | Yes | 1 | Yes | 1 | **8** |  |  |
| Bigirwa | 2009 | Department of Health Sciences of Uganda Martyrs University | Effectiveness of community health workers (CHWS) in the provision of basic preventive and curative maternal, newborn and child health (MNCH) interventions: A systematic review | Yes | 1 | Yes | 1 | Yes | 1 | No | 0 | No | 0 | Yes | 1 | Yes | 1 | Yes | 1 | Yes | 1 | Yes | 1 | Yes | 1 | 9 |  |  |
| Blencowe H | 2011 | BMC Public Health | Lives saved tool supplement detection and treatment of syphilis in pregnancy to reduce syphilis related stillbirths and neonatal mortality. | Yes | 1 | Yes | 1 | Yes | 1 | Yes | 1 | Yes | 1 | Yes | 1 | Yes | 1 | Yes | 1 | Yes | 1 | Yes | 1 | Yes | 1 | **11** | **yes** | **yes** |
| Brion | 2003 | Cochrane Database | Vitamin E supplementation for prevention of morbidity and mortality in preterm infants | Yes | 1 | Yes | 1 | Yes | 1 | No | 0 | No | 0 | Yes | 1 | Yes | 1 | Yes | 1 | Yes | 1 | Yes | 1 | Yes | 1 | 9 |  |  |
| Brocklehurst | 2011 | Cochrane Database | Interventions for reducing the risk of mother-to-child transmission of HIV infection (Review) | Yes | 1 | No | 0 | Yes | 1 | No | 0 | No | 0 | Yes | 1 | Yes | 1 | Yes | 1 | Yes | 1 | Yes | 1 | Yes | 1 | **8** |  |  |
| Brodribb | 2013 | Cochrane Database | Postpartum health professional contact for improving maternal and infant health outcomes for healthy women and their infants | Yes | 1 | Yes | 1 | Yes | 1 | No | 0 | No | 0 | Yes | 1 | Yes | 1 | Yes | 1 | Yes | 1 | Yes | 1 | Yes | 1 | 9 |  |  |
| Brown | 2004 | Acta Paediatr | Vitamin A for acute respiratory infection in developing countries: a meta-analysis. | Yes | 1 | Yes | 1 | Yes | 1 | No | 0 | No | 0 | Yes | 1 | Yes | 1 | Yes | 1 | Yes | 1 | Yes | 1 | Yes | 1 | 9 |  |  |
| Brown | 2009 | Cochrane Database | Early postnatal discharge from hospital for healthy mothers and term infants | Yes | 1 | No | 0 | Yes | 1 | No | 0 | No | 0 | Yes | 1 | Yes | 1 | Yes | 1 | Yes | 1 | Yes | 1 | Yes | 1 | **8** |  |  |
| Brownfoot | 2009 | Cochrane Database | Different corticosteroids and regimens for accelerating foetal lung maturation for women at risk of preterm birth. | Yes | 1 | Yes | 1 | Yes | 1 | Yes | 1 | Yes | 1 | Yes | 1 | Yes | 1 | Yes | 1 | Yes | 1 | Yes | 1 | Yes | 1 | **11** | **yes** | **yes** |
| Bryanton | 2013 | Cochrane Database | Postnatal parental education for optimizing infant general health and parent-infant relationships | Yes | 1 | Yes | 1 | Yes | 1 | No | 0 | No | 0 | Yes | 1 | Yes | 1 | Yes | 1 | Yes | 1 | Yes | 1 | Yes | 1 | 9 |  |  |
| Campbell | 2011 | BMC Public Health | Behavioural interventions for weight management in pregnancy: a systematic review of quantitative and qualitative data. | Yes | 1 | Yes | 1 | Yes | 1 | No | 0 | No | 0 | Yes | 1 | Yes | 1 | Yes | 1 | Yes | 1 | Yes | 1 | Yes | 1 | 9 |  |  |
| Car | 2011 | Cochrane Database | Integrating prevention of mother-to-child HIV transmission (PMTCT) programmes with other health services for preventing HIV infection and improving HIV outcomes in developing countries | Yes | 1 | Yes | 1 | Yes | 1 | No | 0 | No | 0 | Yes | 1 | Yes | 1 | Yes | 1 | Yes | 1 | Yes | 1 | Yes | 1 | 9 |  |  |
| Carroli | 2001 | Lancet | WHO systematic review of randomised controlled trials of routine antenatal care. | Yes | 1 | Yes | 1 | Yes | 1 | Yes | 1 | Yes | 1 | Yes | 1 | Yes | 1 | Yes | 1 | Yes | 1 | Yes | 1 | Yes | 1 | **11** | **yes** | **yes** |
| Chamberlain | 2013 | Cochrane Database | Psychosocial interventions for supporting women to stop smoking in pregnancy | Yes | 1 | Yes | 1 | Yes | 1 | No | 0 | No | 0 | Yes | 1 | Yes | 1 | Yes | 1 | Yes | 1 | Yes | 1 | Yes | 1 | 9 |  |  |
| Churchill | 2010 | Cochrane Database | Interventionist versus expectant care for severe preeclampsia before term | Yes | 1 | No | 0 | Yes | 1 | No | 0 | No | 0 | Yes | 1 | Yes | 1 | Yes | 1 | Yes | 1 | Yes | 1 | Yes | 1 | **8** |  |  |
| Clasen | 2007 | BMJ | Inteventions to improve water quality for preventing diarrhoea: Systematic Review and Meta-analysis | Yes | 1 | Yes | 1 | Yes | 1 | Yes | 1 | Yes | 1 | Yes | 1 | Yes | 1 | Yes | 1 | Yes | 1 | Yes | 1 | Yes | 1 | **11** | **yes** | yes |
| Cluver | 2012 | Cochrane Database | Interventions for helping to turn term breech babies to head first presentation when using external cephalic version. | Yes | 1 | Yes | 1 | Yes | 1 | No | 0 | No | 0 | Yes | 1 | Yes | 1 | Yes | 1 | Yes | 1 | Yes | 1 | Yes | 1 | 9 |  |  |
| Conde-Agudelo | 2006 | JAMA | Birth spacing and risk of adverse perinatal outcomes: a meta-analysis. | Yes | 1 | Yes | 1 | Yes | 1 | Yes | 1 | Yes | 1 | Yes | 1 | Yes | 1 | Yes | 1 | Yes | 1 | Yes | 1 | Yes | 1 | **11** | **yes** | **yes** |
| Conde-Agudelo | 2011 | Cochrane Database | Kangaroo mother care to reduce morbidity and mortality in low birthweight infants. | Yes | 1 | Yes | 1 | Yes | 1 | Yes | 1 | Yes | 1 | Yes | 1 | Yes | 1 | Yes | 1 | Yes | 1 | Yes | 1 | Yes | 1 | **11** | **yes** | **yes** |
| Cotter | 2001 | Cochrane Database | Prophylactic oxytocin for the third stage of labour. | Yes | 1 | Yes | 1 | Yes | 1 | No | 0 | No | 0 | Yes | 1 | Yes | 1 | Yes | 1 | Yes | 1 | Yes | 1 | Yes | 1 | 9 |  |  |
| Cousens | 2010 | Int J Epidemiol | Antibiotics for pre-term pre-labour rupture of membranes: prevention of neonatal deaths due to complications of pre-term birth and infection. | Yes | 1 | Yes | 1 | Yes | 1 | Yes | 1 | Yes | 1 | Yes | 1 | Yes | 1 | Yes | 1 | Yes | 1 | Yes | 1 | Yes | 1 | **11** | **yes** | **yes** |
| Crowley | 2003 | Cochrane Database | Prophylactic corticosteroids for preterm birth | Yes | 1 | Yes | 1 | Yes | 1 | No | 0 | No | 0 | Yes | 1 | Yes | 1 | Yes | 1 | Yes | 1 | Yes | 1 | Yes | 1 | 9 |  |  |
| Crowther | 2010 | Cochrane Database | Vitamin K prior to preterm birth for preventing neonatal periventricular haemorrhage | Yes | 1 | No | 0 | Yes | 1 | No | 0 | No | 0 | Yes | 1 | Yes | 1 | Yes | 1 | Yes | 1 | Yes | 1 | Yes | 1 | **8** |  |  |
| Curtis | 2003 | Lancet | Effect of washing hands with soap on diarrhoea risk in the community: a systematic review | Yes | 1 | Yes | 1 | Yes | 1 | Yes | 1 | Yes | 1 | Yes | 1 | Yes | 1 | Yes | 1 | Yes | 1 | Yes | 1 | Yes | 1 | **11** | **yes** | yes |
| Dangour | 2013 | Cochrane Database | Interventions to improve water quality and supply, sanitation and hygiene practices, and their effects on the nutritional status of children. | Yes | 1 | Yes | 1 | Yes | 1 | No | 0 | No | 0 | Yes | 1 | Yes | 1 | Yes | 1 | Yes | 1 | Yes | 1 | Yes | 1 | 9 |  |  |
| Darlow | 2011 | Cochrane Database | Vitamin A supplementation to prevent mortality and short- and long-term morbidity in very low birthweight infants | Yes | 2 | Yes | 2 | Yes | 2 | No | 1 | No | 1 | Yes | 2 | Yes | 2 | Yes | 2 | Yes | 2 | Yes | 2 | Yes | 1 | 9 |  |  |
| Darmstadt | 2009 | Int J Gynaecol Obstet | 60 Million non-facility births: who can deliver in community settings to reduce intrapartum-related deaths? | Yes | 1 | Yes | 1 | Yes | 1 | Yes | 1 | Yes | 1 | Yes | 1 | Yes | 1 | Yes | 1 | Yes | 1 | Yes | 1 | Yes | 1 | **11** | **yes** | **yes** |
| Dawood | 2013 | Cochrane Database | Intravenous fluids for reducing the duration of labour in low risk nulliparous women | Yes | 1 | Yes | 1 | Yes | 1 | No | 0 | No | 0 | Yes | 1 | Yes | 1 | Yes | 1 | Yes | 1 | Yes | 1 | Yes | 1 | 9 |  |  |
| Debes | 2013 | BMC Public Health | Time to initiation of breastfeeding and neonatal mortality and morbidity: a systematic review. | Yes | 1 | Yes | 1 | Yes | 1 | No | 0 | No | 0 | Yes | 1 | Yes | 1 | Yes | 1 | Yes | 1 | Yes | 1 | Yes | 1 | 9 |  |  |
| Demicheli | 2005 | Cochrane Database | Vaccines for women to prevent neonatal tetanus. | Yes | 1 | Yes | 1 | Yes | 1 | No | 0 | No | 0 | Yes | 1 | Yes | 1 | Yes | 1 | Yes | 1 | Yes | 1 | Yes | 1 | 9 |  |  |
| Dempsey | 2010 | Cochrane Database | Banked preterm versus banked term human milk to promote growth and development in very low birth weight infants | Yes | 1 | Yes | 1 | Yes | 1 | No | 0 | No | 0 | Yes | 1 | Yes | 1 | Yes | 1 | Yes | 1 | Yes | 1 | Yes | 1 | 9 |  |  |
| Dennis | 2013 | Cochrane Database | Psychosocial and psychological interventions for preventing postpartum depression | Yes | 1 | Yes | 1 | Yes | 1 | No | 0 | No | 0 | Yes | 1 | Yes | 1 | Yes | 1 | Yes | 1 | Yes | 1 | Yes | 1 | 9 |  |  |
| Dennis | 2008 | Cochrane Database | Oestrogens and progestins for preventing and treating postpartum depression | Yes | 1 | No | 0 | Yes | 1 | No | 0 | No | 0 | Yes | 1 | Yes | 1 | Yes | 1 | Yes | 1 | Yes | 1 | Yes | 1 | **8** |  |  |
| Dennis | 2007 | Cochrane Database | Psychosocial and psychological interventions for treating ante-natal depression | Yes | 1 | Yes | 1 | Yes | 1 | Yes | 1 | Yes | 1 | Yes | 1 | Yes | 1 | Yes | 1 | Yes | 1 | Yes | 1 | Yes | 1 | **11** | **yes** | **yes** |
| Dodd | 2004 | Cochrane Database | Treatment for women with postpartum iron deficiency anaemia. | Yes | 1 | No | 0 | Yes | 1 | No | 0 | No | 0 | Yes | 1 | Yes | 1 | Yes | 1 | Yes | 1 | Yes | 1 | Yes | 1 | **8** |  |  |
| Dowswell | 2010 | Cochrane Database | Intermittent iron supplementation for improving nutrition and development in children under 12 years of age | Yes | 1 | Yes | 1 | No | 0 | Yes | 1 | Yes | 1 | Yes | 1 | Yes | 1 | Yes | 1 | Yes | 1 | Yes | 1 | Yes | 1 | **10** |  |  |
| Dowswell | 2009 | Cochrane Database | Antenatal day care unites versus hospital admission for women with complicated pregnancy | Yes | 1 | Yes | 1 | No | 0 | Yes | 1 | Yes | 1 | Yes | 1 | Yes | 1 | Yes | 1 | Yes | 1 | Yes | 1 | Yes | 1 | **10** |  |  |
| Doswell | 2010 | Cochrane Database | Alternative versus standard packages of antenatal care for low-risk pregnancy. | Yes | 1 | Yes | 1 | Yes | 1 | Yes | 1 | Yes | 1 | Yes | 1 | Yes | 1 | Yes | 1 | Yes | 1 | Yes | 1 | Yes | 1 | **11** | **yes** | **yes** |
| Downe | 2013 | Cochrane Database | Routine vaginal examinations for assessing progress of labour to improve outcomes for women and babies at term. | Yes | 1 | Yes | 1 | Yes | 1 | No | 0 | No | 0 | Yes | 1 | Yes | 1 | Yes | 1 | Yes | 1 | Yes | 1 | Yes | 1 | 9 |  |  |
| Dror | 2012 | Paediatr Perinat Epidemiol | Interventions with vitamins B6, B12 and C in pregnancy. | Yes | 1 | Yes | 1 | Yes | 1 | No | 0 | No | 0 | Yes | 1 | Yes | 1 | Yes | 1 | Yes | 1 | Yes | 1 | Yes | 1 | 9 |  |  |
| Duley | 2010 | Cochrane Database | Magnesium sulphate versus phenytoin for eclampsia. | Yes | 1 | Yes | 1 | Yes | 1 | Yes | 1 | Yes | 1 | Yes | 1 | Yes | 1 | Yes | 1 | Yes | 1 | Yes | 1 | Yes | 1 | **11** | **yes** | yes |
| Duley | 2007 | Cochrane Database | Antiplatelet agents for preventing pre-eclampsia and its complications. | Yes | 1 | Yes | 1 | Yes | 1 | Yes | 1 | Yes | 1 | Yes | 1 | Yes | 1 | Yes | 1 | Yes | 1 | Yes | 1 | Yes | 1 | **11** | **yes** | yes |
| Earl | 2013 | Cochrane Database | Interventions for hyperthyroidism pre-pregnancy and during pregnancy | Yes | 1 | No | 0 | Yes | 1 | No | 0 | No | 0 | Yes | 1 | Yes | 1 | Yes | 1 | Yes | 1 | Yes | 1 | Yes | 1 | **8** |  |  |
| Ejemot-Nwadiaro | 2010 | Cochrane Database | Alternative versus standard packages of antenatal care for low-risk pregnancy | Yes | 1 | Yes | 1 | Yes | 1 | No | 0 | Yes | 1 | Yes | 1 | Yes | 1 | Yes | 1 | Yes | 1 | Yes | 1 | Yes | 1 | **10** |  |  |
| Flenady | 2013 | Cochrane Database | Prophylactic antibiotics for inhibiting preterm labour with intact membranes. | Yes | 1 | Yes | 1 | Yes | 1 | No | 0 | No | 0 | Yes | 1 | Yes | 1 | Yes | 1 | Yes | 1 | Yes | 1 | Yes | 1 | 9 |  |  |
| Flint | 2008 | Cochrane Database | Cup feeding versus other forms of supplemental enteral feeding for newborn infants unable to fully breastfeed | Yes | 1 | Yes | 1 | Yes | 1 | No | 0 | No | 0 | Yes | 1 | Yes | 1 | Yes | 1 | Yes | 1 | Yes | 1 | Yes | 1 | 9 |  |  |
| French | 2004 | Cochrane Database | Antibiotic regimens for endometritis after delivery. | Yes | 1 | No | 0 | Yes | 1 | No | 0 | No | 0 | Yes | 1 | Yes | 1 | Yes | 1 | Yes | 1 | Yes | 1 | Yes | 1 | **8** |  |  |
| Furber | 2013 | Cochrane Database | Antenatal interventions for reducing weight in obese women for improving pregnancy outcome | Yes | 1 | Yes | 1 | Yes | 1 | No | 0 | No | 0 | Yes | 1 | Yes | 1 | Yes | 1 | Yes | 1 | Yes | 1 | Yes | 1 | 9 |  |  |
| Gaunekar | 2013 | Cochrane Database | Maintenance therapy with calcium channel blockers for preventing preterm birth after threatened preterm labour | Yes | 1 | No | 0 | Yes | 1 | No | 0 | No | 0 | Yes | 1 | Yes | 1 | Yes | 1 | Yes | 1 | Yes | 1 | Yes | 1 | **8** |  |  |
| Gilmore | 2013 | BMC Public Health | Effectiveness of community health workers delivering preventive interventions for maternal and child health in low- and middle-income countries: a systematic review. | Yes | 1 | Yes | 1 | Yes | 1 | No | 0 | No | 0 | Yes | 1 | Yes | 1 | Yes | 1 | Yes | 1 | Yes | 1 | Yes | 1 | 9 |  |  |
| Gogia | 2011 | Indian Pediatr | Community based newborn care: a systematic review and metaanalysis of evidence: UNICEF-PHFI series on newborn and child health, India. | Yes | 1 | Yes | 1 | Yes | 1 | No | 0 | No | 0 | Yes | 1 | Yes | 1 | Yes | 1 | Yes | 1 | Yes | 1 | Yes | 1 | 9 |  |  |
| Gogia | 2011 | Cochrane Database | Vitamin A supplementation for the prevention of morbidity | Yes | 1 | Yes | 1 | Yes | 1 | No | 0 | No | 0 | Yes | 1 | Yes | 1 | Yes | 1 | Yes | 1 | Yes | 1 | Yes | 1 | 9 |  |  |
| Gogia | 2012 | Cochrane Database | Zinc supplementation for mental and motor development in children | Yes | 1 | Yes | 1 | Yes | 1 | No | 0 | No | 0 | Yes | 1 | Yes | 1 | Yes | 1 | Yes | 1 | Yes | 1 | Yes | 1 | 9 |  |  |
| Gordon | 2005 | Cochrane Database | Antibiotic regimens for suspected late onset sepsis in newborn infants | Yes | 1 | No | 0 | Yes | 1 | No | 0 | No | 0 | Yes | 1 | Yes | 1 | Yes | 1 | Yes | 1 | Yes | 1 | Yes | 1 | **8** |  |  |
| Greenough | 2008 | Cochrane Database | Synchronized mechanical ventilation for respiratory support in newborn infants. | Yes | 1 | Yes | 1 | Yes | 1 | No | 0 | No | 0 | Yes | 1 | Yes | 1 | Yes | 1 | Yes | 1 | Yes | 1 | Yes | 1 | 9 |  |  |
| Gregorio | 2011 | Cochrane Database | Early versus Delayed Refeeding for Children with Acute Diarrhoea | Yes | 1 | Yes | 1 | Yes | 1 | No | 0 | No | 0 | Yes | 1 | Yes | 1 | Yes | 1 | Yes | 1 | Yes | 1 | Yes | 1 | 9 |  |  |
| Grimwade | 2006 | Cochrane Database | Cotrimoxazole prophylaxis for opportunistic infections in children with HIV infection. | Yes | 1 | Yes | 1 | Yes | 1 | No | 0 | No | 0 | Yes | 1 | Yes | 1 | Yes | 1 | Yes | 1 | Yes | 1 | Yes | 1 | 9 |  |  |
| Grotto | 2003 | J Pediatr | Vitamin A supplementation and childhood morbidity from diarrhea and respiratory infections: a meta-analysis. | Yes | 1 | Yes | 1 | Yes | 1 | No | 0 | No | 0 | Yes | 1 | Yes | 1 | Yes | 1 | Yes | 1 | Yes | 1 | Yes | 1 | 9 |  |  |
| Gülmezoglu | 2007 | Cochrane Database | Prostaglandins for preventing postpartum haemorrhage. | Yes | 1 | Yes | 1 | Yes | 1 | No | 0 | No | 0 | Yes | 1 | Yes | 1 | Yes | 1 | Yes | 1 | Yes | 1 | Yes | 1 | 9 |  |  |
| Hahn | 2002 | Cochrane Database | Reduced osmolarity oral rehydration solution for treating dehydration caused by acute diarrhoea in children | Yes | 1 | Yes | 1 | Yes | 1 | No | 0 | Yes | 1 | Yes | 1 | Yes | 1 | Yes | 1 | Yes | 1 | Yes | 1 | Yes | 1 | **10** |  |  |
| Hahn | 2008 |  | Hand washing for preventing diarrhoea | Yes | 1 | Yes | 1 | Yes | 1 | No | 0 | No | 0 | Yes | 1 | Yes | 1 | Yes | 1 | Yes | 1 | Yes | 1 | Yes | 1 | 9 |  |  |
| Han | 2013 | Cochrane Database | Different types of dietary advice for women with gestational diabetes mellitus | Yes | 1 | No | 0 | Yes | 1 | No | 0 | No | 0 | Yes | 1 | Yes | 1 | Yes | 1 | Yes | 1 | Yes | 1 | Yes | 1 | **8** |  |  |
| Haider | 2011 | BMC Public Health | Effect of multiple micronutrient supplementation during pregnancy on maternal and birth outcomes | Yes | 1 | Yes | 1 | Yes | 1 | Yes | 1 | Yes | 1 | Yes | 1 | Yes | 1 | Yes | 1 | Yes | 1 | Yes | 1 | Yes | 1 | **11** | **yes** | yes |
| Haider | 2011 | Cochrane Database | Neonatal vitamin A supplementation for the prevention of mortality and morbidity in term neonates in developing countries. | Yes | 1 | Yes | 1 | Yes | 1 | Yes | 1 | Yes | 1 | Yes | 1 | Yes | 1 | Yes | 1 | Yes | 1 | Yes | 1 | Yes | 1 | **11** | **yes** | yes |
| Hartling | 2006 | Cochrane Database | Oral versus intravenous rehydration fro treating dehydration due to gastroenteritis in children | Yes | 1 | Yes | 1 | Yes | 1 | No | 0 | No | 0 | Yes | 1 | Yes | 1 | Yes | 1 | Yes | 1 | Yes | 1 | Yes | 1 | 9 |  |  |
| Henderson-Smart | 2002 | Cochrane Database | Mechanical ventilation for newborn infants with respiratory failure due to pulmonary disease | Yes | 1 | No | 0 | Yes | 1 | No | 0 | No | 0 | Yes | 1 | Yes | 1 | Yes | 1 | Yes | 1 | Yes | 1 | Yes | 1 | **8** |  |  |
| Henderson‐Smart | 2010 | Cochrane Database | Methylxanthine treatment for apnoea in preterm infants | Yes | 1 | Yes | 1 | Yes | 1 | No | 0 | No | 0 | Yes | 1 | Yes | 1 | Yes | 1 | Yes | 1 | Yes | 1 | Yes | 1 | 9 |  |  |
| Hewitt | 2005 | Int J Evid Based Healthc | Nursing and midwifery management of hypoglycaemia in healthy term neonates | Yes | 1 | Yes | 1 | Yes | 1 | No | 0 | No | 0 | Yes | 1 | Yes | 1 | Yes | 1 | Yes | 1 | Yes | 1 | Yes | 1 | 9 |  |  |
| Hodnett | 2012 | Cochrane Database | Alternative versus conventional institutional settings for birth | Yes | 1 | Yes | 1 | Yes | 1 | No | 0 | No | 0 | Yes | 1 | Yes | 1 | Yes | 1 | Yes | 1 | Yes | 1 | Yes | 1 | 9 |  |  |
| Hodnett | 2011 | Cochrane Database | Folic acid supplementation for the prevention of anaemia in preterm neonates | Yes | 1 | Yes | 1 | No | 0 | Yes | 1 | Yes | 1 | Yes | 1 | Yes | 1 | Yes | 1 | Yes | 1 | Yes | 1 | Yes | 1 | **10** |  |  |
| Hodnett | 2010 |  | Reduced osmolarity oral rehydration solution for trating dehydration caused by acute diarrohea in children | Yes | 1 | Yes | 1 | No | 0 | Yes | 1 | Yes | 1 | Yes | 1 | Yes | 1 | Yes | 1 | Yes | 1 | Yes | 1 | Yes | 1 | **10** |  |  |
| Howard | 2005 | Cochrane Database | Antidepressant treatment for post-natal depression | Yes | 1 | Yes | 1 | Yes | 1 | Yes | 1 | Yes | 1 | Yes | 1 | Yes | 1 | Yes | 1 | Yes | 1 | Yes | 1 | Yes | 1 | **11** | **yes** | yes |
| Hofmeyr | 2012 | Cochrane Database | Cephalic version by postural management for breech presentation | Yes | 1 | No | 0 | Yes | 1 | No | 0 | No | 0 | Yes | 1 | Yes | 1 | Yes | 1 | Yes | 1 | Yes | 1 | Yes | 1 | **8** |  |  |
| Hofmeyr | 2012 | Cochrane Database | External cephalic version for breech presentation at  term. | Yes | 1 | Yes | 1 | Yes | 1 | No | 0 | No | 0 | Yes | 1 | Yes | 1 | Yes | 1 | Yes | 1 | Yes | 1 | Yes | 1 | 9 |  |  |
| hofmeyr | 2013 | Cochrane Database | Uterine massage for preventing postpartum haemorrhage. | Yes | 1 | Yes | 1 | Yes | 1 | No | 0 | No | 0 | Yes | 1 | Yes | 1 | Yes | 1 | Yes | 1 | Yes | 1 | Yes | 1 | 9 |  |  |
| Hofmeyr | 2006 | Cochrane Database | Calcium supplementation during pregnancy for preventing hypertensive disorders and related problems. | Yes | 1 | Yes | 1 | Yes | 1 | Yes | 1 | Yes | 1 | Yes | 1 | Yes | 1 | Yes | 1 | Yes | 1 | Yes | 1 | Yes | 1 | **11** | **yes** | yes |
| Homer | 2012 | Cochrane Database | Group versus conventional antenatal care for women. | Yes | 1 | Yes | 1 | No | 0 | Yes | 1 | Yes | 1 | Yes | 1 | Yes | 1 | Yes | 1 | Yes | 1 | Yes | 1 | Yes | 1 | **10** |  |  |
| Horvarth | 2009 | Cochrane Database | Interventions for preventing late postnatal mother-to-child transmission of HIV | Yes | 1 | Yes | 1 | No | 0 | Yes | 1 | Yes | 1 | Yes | 1 | Yes | 1 | Yes | 1 | Yes | 1 | Yes | 1 | Yes | 1 | **10** |  |  |
| Humphreys | 2010 | Cochrane Database | Prevention of diarrhorea in children with HIV infection or exposure to maternal HIV infection | Yes | 1 | Yes | 1 | Yes | 1 | Yes | 1 | Yes | 1 | Yes | 1 | Yes | 1 | Yes | 1 | Yes | 1 | Yes | 1 | Yes | 1 | **11** | **yes** | yes |
| Hundley | 2011 | Midwifery | Are birth kits a good idea? A systematic review of the evidence | Yes | 1 | Yes | 1 | Yes | 1 | Yes | 1 | Yes | 1 | Yes | 1 | Yes | 1 | Yes | 1 | Yes | 1 | Yes | 1 | Yes | 1 | **11** | **yes** | **yes** |
| Hussain | 2011 | BMC Public Health | Elective induction for pregnancies at or beyond 41 weeks of gestation and its impact onstillbirths: a systematic review with meta-analysis. | Yes | 1 | Yes | 1 | Yes | 1 | No | 0 | No | 0 | Yes | 1 | Yes | 1 | Yes | 1 | Yes | 1 | Yes | 1 | Yes | 1 | 9 |  |  |
| Hutton | 2006 | Cochrane Database | External cephalic version for breech presentation before term. | Yes | 1 | No | 0 | Yes | 1 | No | 0 | No | 0 | Yes | 1 | Yes | 1 | Yes | 1 | Yes | 1 | Yes | 1 | Yes | 1 | **8** |  |  |
| Imdad | 2011 | Cochrane Database | Vitamin A supplementation for preventing morbidity and mortality in children from 6 months to 5 years of age | Yes | 1 | Yes | 1 | Yes | 1 | yes | 1 | yes | 1 | Yes | 1 | Yes | 1 | Yes | 1 | Yes | 1 | Yes | 1 | Yes | 1 | **11** | **yes** | **yes** |
| Imdad | 2011 | BMC Public Health | The effect of folic acid, protein energy and multiple micronutrient supplements in pregnancy on stillbirths. | Yes | 1 | Yes | 1 | Yes | 1 | Yes | 1 | Yes | 1 | Yes | 1 | Yes | 1 | Yes | 1 | Yes | 1 | Yes | 1 | Yes | 1 | **11** | **yes** | **unclear** |
| Imdad | 2011 | BMC Public Health | Effect of balanced protein energy supplementation during pregnancy. | Yes | 1 | Yes | 1 | Yes | 1 | Yes | 1 | Yes | 1 | Yes | 1 | Yes | 1 | Yes | 1 | Yes | 1 | Yes | 1 | Yes | 1 | **11** | **yes** | **yes** |
| Imdad | 2011 | BMC Public Health | Role of calcium supplementation during pregnancy in reducing risk of developing gestational hypertensive disorders: a meta-analysis of studies from developing countries. | Yes | 1 | Yes | 1 | Yes | 1 | Yes | 1 | Yes | 1 | Yes | 1 | Yes | 1 | Yes | 1 | Yes | 1 | Yes | 1 | Yes | 1 | **11** | **yes** | **yes** |
| Inglis | 2007 | Cochrane Database | Prophylactic antibiotics to reduce morbidity and mortality in ventilated newborn infants | Yes | 1 | Yes | 1 | Yes | 1 | No | 0 | No | 0 | Yes | 1 | Yes | 1 | Yes | 1 | Yes | 1 | Yes | 1 | Yes | 1 | 9 |  |  |
| Jacobs | 2013 | Cochrane Database | Cooling for newborns with hypoxic ischaemic encephalopathy | Yes | 1 | Yes | 1 | Yes | 1 | No | 0 | No | 0 | Yes | 1 | Yes | 1 | Yes | 1 | Yes | 1 | Yes | 1 | Yes | 1 | 9 |  |  |
| Jagannath | 2012 | Cochrane Database | Routine neonatal circumcision for the prevention of urinary tract infections in infancy | Yes | 1 | Yes | 1 | Yes | 1 | No | 0 | No | 0 | Yes | 1 | Yes | 1 | Yes | 1 | Yes | 1 | Yes | 1 | Yes | 1 | 9 |  |  |
| Kassab | 2013 | Cochrane Database | Furosemide for transient tachypnoea of the newborn. | Yes | 1 | No | 0 | Yes | 1 | No | 0 | No | 0 | Yes | 1 | Yes | 1 | Yes | 1 | Yes | 1 | Yes | 1 | Yes | 1 | **8** |  |  |
| Kelly | 2013 | Cochrane Database | Outpatient versus inpatient induction of labour for improving birth outcomes | Yes | 1 | Yes | 1 | Yes | 1 | No | 0 | No | 0 | Yes | 1 | Yes | 1 | Yes | 1 | Yes | 1 | Yes | 1 | Yes | 1 | 9 |  |  |
| Kendrick | 2007 | Cochrane Database | Parenting interventions for the prevention of unintentional injuries in childhood | Yes | 1 | Yes | 1 | Yes | 1 | Yes | 1 | Yes | 1 | Yes | 1 | Yes | 1 | Yes | 1 | Yes | 1 | No | 0 | Yes | 1 | **10** |  |  |
| Kenyon | 2010 | Cochrane Database | Antibiotics for preterm rupture of membranes. | Yes | 1 | Yes | 1 | Yes | 1 | Yes | 1 | Yes | 1 | Yes | 1 | Yes | 1 | Yes | 1 | Yes | 1 | Yes | 1 | Yes | 1 | **11** | **yes** | **yes** |
| Khan | 2013 | BMC Public Health | Interventions to reduce neonatal mortality from neonatal tetanus in low and middle income countries--a systematic review. | Yes | 1 | Yes | 1 | Yes | 1 | No | 0 | No | 0 | Yes | 1 | Yes | 1 | Yes | 1 | Yes | 1 | Yes | 1 | Yes | 1 | 9 |  |  |
| Kidney | 2009 | BMC Pregnancy Childbirth | Systematic review of effect of community-level interventions to reduce maternal mortality. | Yes | 1 | Yes | 1 | Yes | 1 | Yes | 1 | Yes | 1 | Yes | 1 | Yes | 1 | Yes | 1 | Yes | 1 | Yes | 1 | Yes | 1 | **11** | **yes** | **yes** |
| Kikuchi | 2013 | PROSPERO International prospective register of systematic reviews | Defining the continuum of care in maternal, newborn and child health: which linkages are effective at improving maternal and newborn outcomes in low and middle income countries? A systematic review of quantitative evidence | Yes | 1 | Yes | 1 | Yes | 1 | No | 0 | No | 0 | Yes | 1 | Yes | 1 | Yes | 1 | Yes | 1 | Yes | 1 | Yes | 1 | 9 |  |  |
| Kozuki | 2013 | BMC Public Health | The associations of birth intervals with small-forgestational- age, preterm, and neonatal and infant mortality: a metaanalysis. | Yes | 1 | No | 0 | Yes | 1 | No | 0 | No | 0 | Yes | 1 | Yes | 1 | Yes | 1 | Yes | 1 | Yes | 1 | Yes | 1 | **8** |  |  |
| Kramer | 2003 | Cochrane Database | Energy and protein intake in pregnancy. | Yes | 1 | Yes | 1 | Yes | 1 | Yes | 1 | Yes | 1 | Yes | 1 | Yes | 1 | Yes | 1 | Yes | 1 | Yes | 1 | Yes | 1 | **11** | **yes** | **yes** |
| Lassi | 2010 | Cochrane Database | Community-based intervention packages for reducing maternal and neonatal morbidity and mortality and improving neonatal outcomes. | Yes | 1 | Yes | 1 | Yes | 1 | Yes | 1 | Yes | 1 | Yes | 1 | Yes | 1 | Yes | 1 | Yes | 1 | Yes | 1 | Yes | 1 | **11** | **yes** | **yes** |
| Lavender | 2013 | Cochrane Database | Telephone support for women during pregnancy and the first six weeks postpartum. | Yes | 1 | No | 0 | Yes | 1 | No | 0 | No | 0 | Yes | 1 | Yes | 1 | Yes | 1 | Yes | 1 | Yes | 1 | Yes | 1 | **8** |  |  |
| Lazzarini | 2013 | Cochrane Database | Oral zinc for treating diarrhoea in children | Yes | 1 | Yes | 1 | Yes | 1 | Yes | 1 | Yes | 1 | Yes | 1 | Yes | 1 | Yes | 1 | Yes | 1 | Yes | 1 | Yes | 1 | **11** | **yes** | **yes** |
| Lee | 2011 | BMC Public Health | Neonatal resuscitation and immediate newborn assessment and stimulation for the prevention of neonatal deaths: a systematic review, meta-analysis and Delphi estimation of mortality effect | Yes | 1 | Yes | 1 | Yes | 1 | No | 0 | No | 0 | Yes | 1 | Yes | 1 | Yes | 1 | Yes | 1 | Yes | 1 | Yes | 1 | 9 |  |  |
| Lee | 2008 | Cochrane Database | Antenatal breast examination for promoting breastfeeding | Yes | 1 | Yes | 1 | Yes | 1 | No | 0 | No | 0 | Yes | 1 | Yes | 1 | Yes | 1 | Yes | 1 | Yes | 1 | Yes | 1 | 9 |  |  |
| Lenters | 2013 | BMC Public Health | Systematic review of strategies to increase use of oral rehydration solution at the household level. | Yes | 1 | Yes | 1 | Yes | 1 | Yes | 1 | Yes | 1 | Yes | 1 | Yes | 1 | Yes | 1 | Yes | 1 | Yes | 1 | Yes | 1 | **11** | **yes** | yes |
| Lenters | 2013 | BMC Public Health | Treatment of severe and moderate acute malnutrition in low- and middle-income settings: a systematic review, meta-analysis and Delphi process. | Yes | 1 | Yes | 1 | Yes | 1 | No | 0 | No | 0 | Yes | 1 | Yes | 1 | Yes | 1 | Yes | 1 | Yes | 1 | Yes | 1 | 9 |  |  |
| Lewin | 2010 | Cochrane Database | Lay health workers in primary and community health care for maternal and child health and the management of infectious diseases | Yes | 1 | Yes | 1 | Yes | 1 | Yes | 1 | Yes | 1 | Yes | 1 | Yes | 1 | Yes | 1 | Yes | 1 | Yes | 1 | Yes | 1 | **11** | **yes** | **yes** |
| Magee | 2013 | Cochrane Database | Prevention and treatment of postpartum hypertension | Yes | 1 | No | 0 | Yes | 1 | No | 0 | No | 0 | Yes | 1 | Yes | 1 | Yes | 1 | Yes | 1 | Yes | 1 | Yes | 1 | **8** |  |  |
| Magee | 2003 | Cochrane Database | Oral beta-blockers for mild to moderate hypertension during pregnancy. | Yes | 1 | No | 0 | Yes | 1 | No | 0 | No | 0 | Yes | 1 | Yes | 1 | Yes | 1 | Yes | 1 | Yes | 1 | Yes | 1 | **8** |  |  |
| Mayo-Wilson | 2011 | BMJ | Vitamin A supplements for preventing mortality, illness, and blindness in children aged under 5: systematic review and meta-analysis. | Yes | 1 | No | 0 | Yes | 1 | No | 0 | No | 0 | Yes | 1 | Yes | 1 | Yes | 1 | Yes | 1 | Yes | 1 | Yes | 1 | **8** |  |  |
| McCall | 2010 | Cochrane Database | Interventions to prevent hypothermia at birth in preterm and/or low birthweight infants | Yes | 1 | Yes | 1 | Yes | 1 | No | 0 | No | 0 | Yes | 1 | Yes | 1 | Yes | 1 | Yes | 1 | Yes | 1 | Yes | 1 | 9 |  |  |
| McDonald | 2013 | Cochrane Database | Effect of timing of umbilical cord clamping of term infants on maternal and neonatal outcomes | Yes | 1 | No | 0 | Yes | 1 | No | 0 | No | 0 | Yes | 1 | Yes | 1 | Yes | 1 | Yes | 1 | Yes | 1 | Yes | 1 | **8** |  |  |
| Meher | 2005 | Cochrane Database | Bed rest with or without hospitalisation for hypertension during pregnancy | Yes | 1 | Yes | 1 | Yes | 1 | No | 0 | No | 0 | Yes | 1 | Yes | 1 | Yes | 1 | Yes | 1 | Yes | 1 | Yes | 1 | 9 |  |  |
| Miller | 2013 | Cochrane Database | Dietary supplements for preventing postnatal depression | Yes | 1 | No | 0 | Yes | 1 | No | 0 | No | 0 | Yes | 1 | Yes | 1 | Yes | 1 | Yes | 1 | Yes | 1 | Yes | 1 | **8** |  |  |
| Miller | 2011 | Cochrane Database | Home based child development interventions for preschool children from socially disadvantaged families | Yes | 1 | No | 0 | Yes | 1 | No | 0 | No | 0 | Yes | 1 | Yes | 1 | Yes | 1 | Yes | 1 | Yes | 1 | Yes | 1 | **8** |  |  |
| Montgomery | 2009 |  | Media-based behavioural treatments for behavioural problems in children. | Yes | 1 | Yes | 1 | No | 1 | Yes | 1 | Yes | 1 | Yes | 1 | Yes | 1 | Yes | 1 | Yes | 1 | Yes | 1 | Yes | 1 | **10** |  |  |
| Moore | 2012 | Cochrane Database | Early skin-to-skin contact for mothers and their healthy newborn infants. | Yes | 1 | Yes | 1 | Yes | 1 | Yes | 1 | Yes | 1 | Yes | 1 | Yes | 1 | Yes | 1 | Yes | 1 | Yes | 1 | Yes | 1 | **11** | **yes** | **yes** |
| Motaze | 2013 | Cochrane Database | Treatment interventions for diarrhoea in HIV‐infected and HIV‐exposed children | Yes | 1 | Yes | 1 | No | 1 | Yes | 1 | Yes | 1 | Yes | 1 | Yes | 1 | Yes | 1 | Yes | 1 | Yes | 1 | Yes | 1 | **10** |  |  |
| Mousa | 2007 | Cochrane Database | Treatment for primary postpartum haemorrhage. | Yes | 1 | No | 0 | No | 0 | Yes | 1 | Yes | 1 | Yes | 1 | Yes | 1 | Yes | 1 | Yes | 1 | Yes | 1 | Yes | 1 | **9** |  |  |
| Mtitimila | 2004 | Cochrane Database | Antibiotic regimens for suspected early neonatal sepsis | Yes | 1 | No | 0 | Yes | 1 | No | 0 | No | 0 | Yes | 1 | Yes | 1 | Yes | 1 | Yes | 1 | Yes | 1 | Yes | 1 | **8** |  |  |
| Muktabhant | 2012 | Cochrane Database | Interventions for preventing excessive weight gain during pregnancy | Yes | 1 | No | 0 | Yes | 1 | No | 0 | No | 0 | Yes | 1 | Yes | 1 | Yes | 1 | Yes | 1 | Yes | 1 | Yes | 1 | **8** |  |  |
| Musekiwa | 2011 | Cochrane Database | Oral rehydration salt solution for treating cholera: ≤ 270 mOsm/L solutions vs ≥ 310 mOsm/L solutions | Yes | 1 | Yes | 1 | Yes | 1 | No | 0 | Yes | 1 | Yes | 1 | Yes | 1 | Yes | 1 | Yes | 1 | Yes | 1 | Yes | 1 | **10** |  |  |
| Mwansa-Kambafwile | 2010 | Int J Epidemiol | Antenatal steroids in preterm labour for the prevention of neonatal deaths due to complications of preterm birth | Yes | 1 | Yes | 1 | Yes | 1 | Yes | 1 | Yes | 1 | Yes | 1 | Yes | 1 | Yes | 1 | Yes | 1 | Yes | 1 | Yes | 1 | **11** | **yes** | **unclear** |
| Nissensohn | 2013 | Matern Child Nutr | Effect of zinc intake on serum/plasma zinc status in infants: a meta-analysis. | Yes | 1 | No | 0 | Yes | 1 | No | 0 | No | 0 | Yes | 1 | Yes | 1 | Yes | 1 | Yes | 1 | Yes | 1 | Yes | 1 | **8** |  |  |
| O'Donnell | 2004 | Cochrane Database | Positive end-expiratory pressure for resuscitation of newborn infants at birth. | Yes | 1 | No | 0 | Yes | 1 | No | 0 | No | 0 | Yes | 1 | Yes | 1 | Yes | 1 | Yes | 1 | Yes | 1 | Yes | 1 | **8** |  |  |
| Ohlsson | 2013 | Cochrane Database | Intravenous immunoglobulin for preventing infection in preterm and/or low birth weight infants | Yes | 1 | Yes | 1 | Yes | 1 | No | 0 | No | 0 | Yes | 1 | Yes | 1 | Yes | 1 | Yes | 1 | Yes | 1 | Yes | 1 | 9 |  |  |
| Oken | 2008 | International Journal of Obesity | Maternal smoking during pregnancy and child overweight: systematic review and meta-analysis | Yes | 1 | Yes | 1 | Yes | 1 | No | 0 | No | 0 | Yes | 1 | Yes | 1 | Yes | 1 | Yes | 1 | Yes | 1 | Yes | 1 | 9 |  |  |
| Okoromah | 2004 | Cochrane Database | Antifungal agents for the treatment of mucocutaneous candidiasis in neonates and children | Yes | 1 | No | 0 | Yes | 1 | No | 0 | No | 0 | Yes | 1 | Yes | 1 | Yes | 1 | Yes | 1 | Yes | 1 | Yes | 1 | **8** |  |  |
| Oloyede | 2012 | Cochrane Database | Specialized antenatal clinics for women with a pregnancy at high risk of preterm birth (excluding multiple pregnancy) to improve maternal and infant outcomes | Yes | 1 | Yes | 1 | Yes | 1 | No | 0 | No | 0 | Yes | 1 | Yes | 1 | Yes | 1 | Yes | 1 | Yes | 1 | Yes | 1 | 9 |  |  |
| Oude Luttikuis | 2009 |  | Interventions for treating obesity in children | Yes | 1 | Yes | 1 | Yes | 1 | No | 0 | No | 0 | Yes | 1 | Yes | 1 | Yes | 1 | Yes | 1 | Yes | 1 | Yes | 1 | 9 |  |  |
| Panpaniach | 2009 | Cochrane Database | Growth monitoring in children | Yes | 1 | No | 0 | Yes | 1 | No | 0 | No | 0 | Yes | 1 | Yes | 1 | Yes | 1 | Yes | 1 | Yes | 1 | Yes | 1 | **8** |  |  |
| Parab | 2013 | Cochrane Database | Specialist home-based nursing services for children with acute and chronic illnesses | Yes | 1 | Yes | 1 | Yes | 1 | No | 0 | No | 0 | Yes | 1 | Yes | 1 | Yes | 1 | Yes | 1 | Yes | 1 | Yes | 1 | 9 |  |  |
| Pattinson | 2009 | Cochrane Database | Critical incident audit and feedback to improve perinatal and maternal mortality and morbidity | Yes | 1 | No | 0 | Yes | 1 | No | 0 | No | 0 | Yes | 1 | Yes | 1 | Yes | 1 | Yes | 1 | Yes | 1 | Yes | 1 | **8** |  |  |
| Pena-Rosas | 2012 | Cochrane Database | Daily oral iron supplementation during pregnancy. | Yes | 1 | Yes | 1 | Yes | 1 | Yes | 1 | Yes | 1 | Yes | 1 | Yes | 1 | Yes | 1 | Yes | 1 | Yes | 1 | Yes | 1 | **11** | **yes** | **yes** |
| Penazzato | 2014 | Cochrane Database | Optimisation of antiretroviral therapy in HIV-infected children under 3 years of age | Yes | 1 | Yes | 1 | Yes | 1 | Yes | 1 | Yes | 1 | Yes | 1 | Yes | 1 | Yes | 1 | Yes | 1 | Yes | 1 | Yes | 1 | **11** | **yes** | **yes** |
| Prendiville | 2000 | Cochrane Database | Active versus expectant management in the third stage of labour (Review). | Yes | 1 | Yes | 1 | Yes | 1 | No | 0 | No | 0 | Yes | 1 | Yes | 1 | Yes | 1 | Yes | 1 | Yes | 1 | Yes | 1 | 9 |  |  |
| Priest | 2008 | Cochrane Database | Policy interventions implemented through sporting organisations for promoting healthy behaviour change | Yes | 1 | Yes | 1 | Yes | 1 | No | 0 | No | 0 | Yes | 1 | Yes | 1 | Yes | 1 | Yes | 1 | Yes | 1 | Yes | 1 | 9 |  |  |
| Priest | 2008 | Cochrane Database | Interventions implemented through sporting organisations for increasing participation in sport | Yes | 1 | No | 0 | Yes | 1 | No | 0 | No | 0 | Yes | 1 | Yes | 1 | Yes | 1 | Yes | 1 | Yes | 1 | Yes | 1 | **8** |  |  |
| Prost | 2013 | Lancet | Women’s groups practising participatory learning and action to improve maternal and newborn health in low-resource settings: a systematic review and meta-analysis | Yes | 1 | No | 0 | Yes | 1 | No | 0 | No | 0 | Yes | 1 | Yes | 1 | Yes | 1 | Yes | 1 | Yes | 1 | Yes | 1 | **8** |  |  |
| Rahman | 2013 | Bull World Health Organ | Interventions for common perinatal mental disorders in women in lowand middle-income countries: a systematic review and meta-analysis | Yes | 1 | Yes | 1 | Yes | 1 | No | 0 | No | 0 | Yes | 1 | Yes | 1 | Yes | 1 | Yes | 1 | Yes | 1 | Yes | 1 | 9 |  |  |
| Ramakrishnan | 2012 | Paediatr Perinat Epidemiol | Effect of women's nutrition before and during early pregnancy on maternal and infant outcomes: a systematic review. | Yes | 1 | Yes | 1 | Yes | 1 | No | 0 | No | 0 | Yes | 1 | Yes | 1 | Yes | 1 | Yes | 1 | Yes | 1 | Yes | 1 | 9 |  |  |
| Read | 2005 | Cochrane Database | Efficacy and safety of cesarean delivery for prevention of mother‐to‐child transmission of HIV‐1 | Yes | 1 | Yes | 1 | Yes | 1 | No | 0 | No | 0 | Yes | 1 | Yes | 1 | Yes | 1 | Yes | 1 | Yes | 1 | Yes | 1 | 9 |  |  |
| Reid | 2012 | Cochrane Database | Interventions for clinical and subclinical hypothyroidism in pregnancy | Yes | 1 | Yes | 1 | Yes | 1 | No | 0 | No | 0 | Yes | 1 | Yes | 1 | Yes | 1 | Yes | 1 | Yes | 1 | Yes | 1 | 9 |  |  |
| Renfrew | 2012 | Cochrane Database | Support for healthy breastfeeding mothers with healthy term babies | Yes | 1 | Yes | 1 | Yes | 1 | No | 0 | No | 0 | Yes | 1 | Yes | 1 | Yes | 1 | Yes | 1 | Yes | 1 | Yes | 1 | 9 |  |  |
| Roberts | 2006 | Cochrane Database | Antenatal corticosteroids for accelerating fetal lung maturation for women at risk of preterm birth. | Yes | 1 | Yes | 1 | Yes | 1 | Yes | 1 | Yes | 1 | Yes | 1 | Yes | 1 | Yes | 1 | Yes | 1 | Yes | 1 | Yes | 1 | **11** | **yes** | **yes** |
| Sado | 2012 | Cochrane Database | Hypnosis during pregnancy, childbirth, and the postnatal period for preventing postnatal depression | Yes | 1 | Yes | 1 | Yes | 1 | No | 0 | No | 0 | Yes | 1 | Yes | 1 | Yes | 1 | Yes | 1 | Yes | 1 | Yes | 1 | 9 |  |  |
| Say | 2009 | Cochrane Database | Calcium channel blockers for potential impaired fetal growth | Yes | 1 | Yes | 1 | Yes | 1 | No | 0 | No | 0 | Yes | 1 | Yes | 1 | Yes | 1 | Yes | 1 | Yes | 1 | Yes | 1 | 9 |  |  |
| Sazawal | 2003 | Lancet Infect Dis | Effect of pneumonia case management on mortality in neonates, infants, and preschool children: a meta-analysis of community-based trials | Yes | 1 | Yes | 1 | Yes | 1 | No | 0 | No | 0 | Yes | 1 | Yes | 1 | Yes | 1 | Yes | 1 | Yes | 1 | Yes | 1 | 9 |  |  |
| Shrimpton | 2009 | Food Nutr Bull | Multiple micronutrient supplementation during pregnancy in developing-country settings: policy and program implications of the results of a meta-analysis. | Yes | 1 | Yes | 1 | Yes | 1 | No | 0 | No | 0 | Yes | 1 | Yes | 1 | Yes | 1 | Yes | 1 | Yes | 1 | Yes | 1 | 9 |  |  |
| Sibley | 2007 | Cochrane Database | Traditional birth attendant training for improving health behaviours and pregnancy outcomes. | Yes | 1 | Yes | 1 | Yes | 1 | Yes | 1 | Yes | 1 | Yes | 1 | Yes | 1 | Yes | 1 | Yes | 1 | Yes | 1 | Yes | 1 | **11** | **yes** | **yes** |
| Siegfried | 2012 | Cochrane Database | Micronutrient supplementation in pregnant women with HIV infection | Yes | 1 | No | 0 | Yes | 1 | No | 0 | No | 0 | Yes | 1 | Yes | 1 | Yes | 1 | Yes | 1 | Yes | 1 | Yes | 1 | **8** |  |  |
| Siegfried | 2011 | Cochrane Database | Antiretrovirals for reducing the risk of mother-to-child transmission of HIV infection. | Yes | 1 | Yes | 1 | Yes | 1 | Yes | 1 | Yes | 1 | Yes | 1 | Yes | 1 | Yes | 1 | Yes | 1 | Yes | 1 | Yes | 1 | **11** | **yes** | **yes** |
| Sinclair | 2011 | Cochrane Database | Oral vaccines for preventing cholera | Yes | 1 | Yes | 1 | Yes | 1 | No | 0 | No | 0 | Yes | 1 | Yes | 1 | Yes | 1 | Yes | 1 | Yes | 1 | Yes | 1 | 9 |  |  |
| Soares-Weiser | 2004 | Cochrane Database | Rotavirus vaccine for preventing diarrhoea | Yes | 1 | No | 0 | Yes | 1 | No | 0 | No | 0 | Yes | 1 | Yes | 1 | Yes | 1 | Yes | 1 | Yes | 1 | Yes | 1 | **8** |  |  |
| Soares‐Weiser | 2012 | Cochrane Database | Vaccines for preventing rotavirus diarrhoea: vaccines in use | Yes | 1 | No | 0 | Yes | 1 | No | 0 | No | 0 | Yes | 1 | Yes | 1 | Yes | 1 | Yes | 1 | Yes | 1 | Yes | 1 | **8** |  |  |
| Soll | 2009 | Cochrane Database | Multiple versus single doses of exogenous surfactant for the prevention or treatment of neonatal respiratory distress syndrome. | Yes | 1 | Yes | 1 | Yes | 1 | No | 0 | No | 0 | Yes | 1 | Yes | 1 | Yes | 1 | Yes | 1 | Yes | 1 | Yes | 1 | 9 |  |  |
| Soltani | 2010 | Cochrane Database | Timing of prophylactic uterotonics for the third stage of labour after vaginal birth. | Yes | 1 | No | 0 | Yes | 1 | No | 0 | No | 0 | Yes | 1 | Yes | 1 | Yes | 1 | Yes | 1 | Yes | 1 | Yes | 1 | **8** |  |  |
| Sturt | 2010 | Cochrane Database | Antiretroviral therapy (ART) for treating HIV infection in ART-eligible pregnant women | Yes | 1 | Yes | 1 | Yes | 1 | No | 0 | No | 0 | Yes | 1 | Yes | 1 | Yes | 1 | Yes | 1 | Yes | 1 | Yes | 1 | 9 |  |  |
| Sunguya | 2013 | Nutr J | Effectiveness of nutrition training of health workers toward improving caregivers' feeding practices for children aged six months to two years: a systematic review. | Yes | 1 | No | 0 | Yes | 1 | No | 0 | No | 0 | Yes | 1 | Yes | 1 | Yes | 1 | Yes | 1 | Yes | 1 | Yes | 1 | **8** |  |  |
| Tan | 2012 | Cochrane Database | Maternal glucose administration for facilitating tests of fetal wellbeing |  |  |  |  |  |  |  |  |  |  |  |  |  |  |  |  |  |  |  |  |  |  |  |  |  |
| Tan | 2005 | Cochrane Database | Clinical decision support systems for neonatal care | Yes | 1 | No | 0 | Yes | 1 | No | 0 | No | 0 | Yes | 1 | Yes | 1 | Yes | 1 | Yes | 1 | Yes | 1 | Yes | 1 | **8** |  |  |
| Tan | 2005 | Cochrane Database | Air versus oxygen for resuscitation of infants at birth. | Yes | 1 | Yes | 1 | Yes | 1 | No | 0 | No | 0 | Yes | 1 | Yes | 1 | Yes | 1 | Yes | 1 | Yes | 1 | Yes | 1 | 9 |  |  |
| Tang | 2012 | Cochrane Database | Hormonal and intrauterine methods for contraception for women aged 25 years and younger | Yes | 1 | Yes | 1 | Yes | 1 | No | 0 | No | 0 | Yes | 1 | Yes | 1 | Yes | 1 | Yes | 1 | Yes | 1 | Yes | 1 | 9 |  |  |
| Thangaratinam | 2012 | BMJ | Effects of interventions in pregnancy on maternal weight and obstetric outcomes: meta-analysis of randomised evidence | Yes | 1 | Yes | 1 | Yes | 1 | No | 0 | No | 0 | Yes | 1 | Yes | 1 | Yes | 1 | Yes | 1 | Yes | 1 | Yes | 1 | 9 |  |  |
| Thaver | 2009 | Cochrane Database | The effect of community health educational interventions on newborn survival in developing countries | Yes | 1 | Yes | 1 | Yes | 1 | No | 0 | No | 0 | Yes | 1 | Yes | 1 | Yes | 1 | Yes | 1 | Yes | 1 | Yes | 1 | 9 |  |  |
| Thorne-Lyman | 2013 | Paediatr Perinat Epidemiol | Vitamin A and carotenoids during pregnancy and maternal, neonatal and infant health outcomes: A systematic review and meta-analysis | Yes | 1 | No | 0 | Yes | 1 | No | 0 | No | 0 | Yes | 1 | Yes | 1 | Yes | 1 | Yes | 1 | Yes | 1 | Yes | 1 | **8** |  |  |
| Tieu | 2011 | Cochrane Database | neonatal vitamin A supplementation for the prevention of mortality and morbidity in term neonates in developing countries (Review) | Yes | 1 | Yes | 1 | No | 0 | Yes | 1 | Yes | 1 | Yes | 1 | Yes | 1 | Yes | 1 | Yes | 1 | Yes | 1 | Yes | 1 | **10** |  |  |
| Tieu | 2010 | Cochrane Database | Oral anti-diabetic agents for women with pre-existing diabetes mellitus/imparied glucose tolerance or previous gestational diabets mellitus | Yes | 1 | Yes | 1 | Yes | 1 | No | 0 | No | 0 | Yes | 1 | Yes | 1 | Yes | 1 | Yes | 1 | Yes | 1 | Yes | 1 | 9 |  |  |
| Tudor Car | 2011 | Cochrane Database | Integrating prevention of mother to child HIV transmission programmes with other health services | Yes | 1 | Yes | 1 | Yes | 1 | No | 0 | No | 0 | Yes | 1 | Yes | 1 | Yes | 1 | Yes | 1 | Yes | 1 | Yes | 1 | 9 |  |  |
| Tuncalp | 2012 | Cochrane Database | Prostaglandins for preventing postpartum haemorrhage. | Yes | 1 | Yes | 1 | Yes | 1 | No | 0 | No | 0 | Yes | 1 | Yes | 1 | Yes | 1 | Yes | 1 | Yes | 1 | Yes | 1 | 9 |  |  |
| Turnball | 2012 | Cochrane Database | Home visits during pregnancy and after birth for women with an alcohol or drug problem | Yes | 1 | Yes | 1 | Yes | 1 | Yes | 1 | Yes | 1 | Yes | 1 | Yes | 1 | Yes | 1 | Yes | 1 | Yes | 1 | Yes | 1 | **11** | **yes** | **yes** |
| Ungerer | 2004 | Cochrane Database | Prophylactic versus selective antibiotics for term newborn infants of mothers with risk factors for neonatal infection | Yes | 1 | Yes | 1 | Yes | 1 | No | 0 | No | 0 | Yes | 1 | Yes | 1 | Yes | 1 | Yes | 1 | Yes | 1 | Yes | 1 | 9 |  |  |
| Utama | 2012 | Cochrane Database | Transplacental versus direct fetal corticosteroid treatment for accelerating fetal lung maturation | Yes | 1 | Yes | 1 | Yes | 1 | No | 0 | No | 0 | Yes | 1 | Yes | 1 | Yes | 1 | Yes | 1 | Yes | 1 | Yes | 1 | 9 |  |  |
| van Lonkhuijzen | 2012 | Cochrane Database | Maternity waiting facilities for improving maternal and neonatal outcome in low-resource countries | Yes | 1 | No | 0 | Yes | 1 | No | 0 | No | 0 | Yes | 1 | Yes | 1 | Yes | 1 | Yes | 1 | Yes | 1 | Yes | 1 | **8** |  |  |
| Villar | 2001 | Cochrane Database | Patterns of routine antenatal care for low-risk pregnancy. | Yes | 1 | Yes | 1 | Yes | 1 | No | 0 | No | 0 | Yes | 1 | Yes | 1 | Yes | 1 | Yes | 1 | Yes | 1 | Yes | 1 | 9 |  |  |
| Walker | 2001 | Cochrane Database | Antibiotics for syphilis diagnosed during pregnancy. | Yes | 1 | Yes | 1 | Yes | 1 | No | 0 | No | 0 | Yes | 1 | Yes | 1 | Yes | 1 | Yes | 1 | Yes | 1 | Yes | 1 | 9 |  |  |
| Waters | 2011 | Cochrane Database | Interventions for preventing obesity in children | Yes | 1 | Yes | 1 | Yes | 1 | No | 0 | No | 0 | Yes | 1 | Yes | 1 | Yes | 1 | Yes | 1 | Yes | 1 | Yes | 1 | 9 |  |  |
| Webster | 2009 | Cochrane Database | Gowning by attendants and visitors in newborn nurseries for prevention of neonatal morbidity and mortality | Yes | 1 | Yes | 1 | Yes | 1 | No | 0 | No | 0 | Yes | 1 | Yes | 1 | Yes | 1 | Yes | 1 | Yes | 1 | Yes | 1 | 9 |  |  |
| Westhoff | 2013 | Cochrane Database | Prophylactic oxytocin for the third stage of labour to prevent postpartum haemorrhage | Yes | 1 | No | 0 | Yes | 1 | No | 0 | No | 0 | Yes | 1 | Yes | 1 | Yes | 1 | Yes | 1 | Yes | 1 | Yes | 1 | **8** |  |  |
| Whitworth | 2012 | Cochrane Database | Routine pre-pregnancy health promotion for improving pregnancy outcomes | Yes | 1 | Yes | 1 | Yes | 1 | Yes | 1 | Yes | 1 | Yes | 1 | Yes | 1 | Yes | 1 | Yes | 1 | Yes | 1 | Yes | 1 | **11** | **yes** | **yes** |
| Wiysonge | 2005 | Cochrane Database | Vaginal disinfection for preventing mother‐to‐child transmission of HIV infection | Yes | 1 | Yes | 1 | Yes | 1 | No | 0 | No | 0 | Yes | 1 | Yes | 1 | Yes | 1 | Yes | 1 | Yes | 1 | Yes | 1 | 9 |  |  |
| Yakoob | 2011 | BMC Public Health | The effect of providing skilled birth attendance and emergency obstetric care in preventing stillbirths. | Yes | 1 | Yes | 1 | Yes | 1 | Yes | 1 | Yes | 1 | Yes | 1 | Yes | 1 | Yes | 1 | Yes | 1 | Yes | 1 | Yes | 1 | **11** | **yes** | **yes** |
| Yonemoto | 2013 | Cochrane Database | Schedules for home visits in the early postpartum period. | Yes | 1 | No | 0 | Yes | 1 | Yes | 1 | Yes | 1 | Yes | 1 | Yes | 1 | Yes | 1 | Yes | 1 | Yes | 1 | Yes | 1 | **10** |  |  |
| Zaidi | 2009 | Cochrane Database | Community based management of neonatal sepsis in developing countries | Yes | 1 | No | 0 | No | 0 | Yes | 1 | Yes | 1 | Yes | 1 | Yes | 1 | Yes | 1 | Yes | 1 | Yes | 1 | Yes | 1 | **9** |  |  |
| Zoritch | 2009 |  | Day care for pre-school children | Yes | 1 | Yes | 1 | Yes | 1 | No | 0 | No | 0 | Yes | 1 | Yes | 1 | Yes | 1 | Yes | 1 | Yes | 1 | Yes | 1 | 9 |  |  |
| Zupan | 2004 | Cochrane Database | Topical umbilical cord care at birth. | Yes | 1 | No | 0 | No | 0 | Yes | 1 | Yes | 1 | Yes | 1 | Yes | 1 | Yes | 1 | Yes | 1 | Yes | 1 | Yes | 1 | **9** |  |  |
|  |  |  |  |  |  |  |  |  |  |  |  |  |  |  |  |  |  |  |  |  |  |  |  |  |  |  |  |  |

For the purposes of this article ‒ which uses the JBI umbrella review methods for analysing and presenting the data ‒ the authors revisited the quality-appraisal steps and, using the JBI critical appraisal checklist tool, assessed two questions for the included studies that were not assessed in the original quality appraisal which involved AMSTAR.

* Question 10 (Were recommendations for policy and / or practice supported by the reported data?)

** Question 11 (Were the specific directives for new research appropriate?)

However, this step has not altered the reporting of the number of studies included. Of the 40 studies that scored 11/11 for the AMSTAR tool, 34 were assessed using questions 10 and 11. All studies had a ‘Yes’ for question 10, and 32 scored ‘Yes’ for question 11. Two studies [^[1]^,^[2]^] had scored unclear for question 11.

**Appendix 7 -** Table showing Ranking of studies with Modified Bhutta et al criteria

|  |  |  |  |  | **Reasons for selection** | | |
| --- | --- | --- | --- | --- | --- | --- | --- |
| **Author** | **Year** | **Journal** | **Title** | **Intervention** | **Evidence of effect on morbidity and mortality** | **Evidence and potential for delivery and inclusion in primary care programmes** | **Evidence on determinants of child health and/ or behaviour change** |
| Blencowe, H [34] | 2011 | BMC Public Health | Lives saved tool supplement detection and treatment of syphilis in pregnancy to reduce syphilis related stillbirths and neonatal mortality. | Syphilis treatment and detection | 2 | 1 | NA |
| Brownfoot, F [44] | 2009 | Cochrane Database | Different corticosteroids and regimens for accelerating foetal lung maturation for women at risk of preterm birth. | Corticosteroids for lung maturation | 1 | 1 | NA |
| Carroli, G^20^ | 2001 | Lancet | WHO systematic review of randomised controlled trials of routine antenatal care. | Reducing antenatal care visits | 2 | 1 | NA |
| Clasen, T [76] | 2007 | BMJ | Interventions to improve water quality for preventing diarrhoea: Systematic Review and Meta-analysis | Improved water quality | 2 | 2 | 2 |
| Conde-Agudelo, A [27] | 2006 | JAMA | Birth spacing and risk of adverse perinatal outcomes: a meta-analysis. | Family planning and birth spacing promotion | 1 | 2 | 2 |
| Conde-Agudelo, A [59] | 2011 | Cochrane Database | Kangaroo mother care to reduce morbidity and mortality in low birth-weight infants. | Kangaroo mother Care | 1 | 1 | 1 |
| Cousens, S [49] | 2010 | Int J Epidemiol | Antibiotics for pre-term pre-labour rupture of membranes: prevention of neonatal deaths due to complications of pre-term birth and infection. | Antibiotics for PROM | 1 | 1 | NA |
| Curtis, V [75] | 2003 | Lancet | Effect of washing hands with soap on diarrhoea risk in the community: a systematic review | Hand washing with soap | 1 | 2 | 2 |
| Darmstadt G, [58] | 2009 | Int J Gynaecol Obstet | 60 Million Non-facility births: who can deliver in community settings to reduce intrapartum-related deaths? | Traditional birth attendants and non- facility births | 1 | 2 | 1 |
| Dennis, C [39] | 2007 | Cochrane Database | Psychosocial and psychological interventions for treating ante-natal depression | Prevention and treatment of antenatal depression | 2 | 2 | 2 |
| Dowswell, T [28] | 2010 | Cochrane Database | Alternative versus standard packages of antenatal care for low-risk pregnancy. | Reducing antenatal care visits | 2 | 1 | NA |
| Duley, L [46] | 2010 | Cochrane Database | Magnesium sulphate versus phenytoin for eclampsia. | Magnesium sulphate usage for eclampsia | 1 | 1 | NA |
| Duley, L [37] | 2007 | Cochrane Database | Antiplatelet agents for preventing pre-eclampsia and its complications. | Antiplatelet agents for preventing pre-eclampsia | 2 | 1 | NA |
| Haider, B [30] | 2011 | BMC Public Health | Effect of multiple micronutrient supplementation during pregnancy on maternal and birth outcomes | Multiple micronutrient supplementation | 1 | 1 | NA |
| Haider, B [62] | 2011 | Cochrane Database | Neonatal vitamin A supplementation for the prevention of mortality and morbidity in term neonates in developing countries. | Preventative vitamin A | 1 | 1 | NA |
| Howard, L [65] | 2005 | Cochrane Database | Antidepressant treatment for post-natal depression | Pharmacological treatment for depression | 1 | 1 | NA |
| Hofmeyr, G [35] | 2006 | Cochrane Database | Calcium supplementation during pregnancy for preventing hypertensive disorders and related problems. | Calcium supplementation for hypertensive disorders | 1 | 1 | NA |
| Humphreys, E[i] | 2010 | Cochrane Database | Prevention of diarrhoea in children with HIV infection or exposure to maternal HIV infection | PMTCT | 2 | 3 | NA |
| Hundley, V [47] | 2011 | Midwifery | Are birth kits a good idea? A systematic review of the evidence | Clean delivery kits for non-facility births | 2 | 1 | 2 |
| Imdad, A [31] | 2011 | BMC Public Health | The effect of folic acid, protein energy and multiple micronutrient supplements in pregnancy on stillbirths. | Folic acid, Protein energy supplementation and multiple micronutrients | 1 | 1 | NA |
| Imdad, A[ii] | 2011 | BMC Public Health | Effect of balanced protein energy supplementation during pregnancy. | Protein energy supplementation | 3 | 2 | NA |
| Imdad, A [36] | 2011 | BMC Public Health | Role of calcium supplementation during pregnancy in reducing risk of developing gestational hypertensive disorders: a meta-analysis of studies from developing countries. | Calcium supplementation for hypertensive disorders | 1 | 1 | NA |
| Imdad, A [69] | 2011 | Cochrane Database | Vitamin A supplementation for preventing morbidity and mortality in children from 6 months to 5 years of age | Vitamin A supplementation | 1 | 1 | NA |
|  |  |  |  |  |  |  |  |
| Kenyon, S^42^ | 2010 | Cochrane Database | Antibiotics for preterm rupture of membranes. | Antibiotics for PROM | 1 | 1 | NA |
| Kidney, E^73^ | 2009 | BMC Pregnancy Childbirth | Systematic review of effect of community-level interventions to reduce maternal mortality. | Community led interventions | 1 | 1 | 1 |
| Kramer, M [32] | 2003 | Cochrane Database | Energy and protein intake in pregnancy. | Protein energy supplementation | 2 | 1 | NA |
| Lassi, Z ^72^ | 2010 | Cochrane Database | Community-based intervention packages for reducing maternal and neonatal morbidity and mortality and improving neonatal outcomes. | Lay health workers and community based interventions | 2 | 2 | 2 |
| Lazzerini, M[iii] | 2013 | Cochrane Database | Oral zinc for treating diarrhoea in children | Oral zinc for treating diarrhoea | 3 | 3 | 3 |
| Lenters, L[iv] | 2013 | BMC Public Health | Systematic review of strategies to increase use of | Increasing use of | 2 | 2 | 3 |
|  |  |  | oral rehydration solution at the household level | oral rehydration solution |  |  |  |
| Lewin, S [71] | 2010 | Cochrane Database | Lay health workers in primary and community health care for maternal and child health and the management of infectious diseases | Lay health workers | 1 | 1 | 1 |
| Moore, E [60] | 2012 | Cochrane Database | Early skin-to-skin contact for mothers and their healthy newborn infants. | Kangaroo mother Care | 1 | 1 | 1 |
| Mwansa-Kambafwile, J [45] | 2010 | Int J Epidemiol | Antenatal steroids in preterm labour for the prevention of neonatal deaths due to complications of preterm birth | Antenatal steroids | 1 | 1 | NA |
| Pena-Rosas,J [33] | 2012 | Cochrane Database | Daily oral iron supplementation during pregnancy. | Iron supplementation | 1 | 1 | NA |
| Penazzato, M [66] | 2014 | Cochrane Database | Optimisation of antiretroviral therapy in HIV-infected children under 3 years of age | ART initiation to prevent PMTCT | 1 | 1 | NA |
| Roberts, D^21^ | 2006 | Cochrane Database | Antenatal corticosteroids for accelerating fetal lung maturation for women at risk of preterm birth. | Antenatal steroids | 1 | 1 | NA |
| Sibley, L^62^ | 2007 | Cochrane Database | Traditional birth attendant training for improving health behaviours and pregnancy outcomes. | Traditional birth attendant training | 1 | 1 | 1 |
| Siegfried, N [40] | 2011 | Cochrane Database | Anti-retrovirals for reducing the risk of mother-to-child transmission of HIV infection. | ART initiation to prevent PMTCT | 1 | 1 | NA |
| Turnball, C[v] | 2012 | Cochrane Database | Home visits during pregnancy and after birth for women with an alcohol or drug problem | Home visits during pregnancy and after birth for women with an alcohol or drug problem | 2 | 2 | 3 |
| Whitworth, M[vi] | 2012 | Cochrane Database | Routine pre-pregnancy health promotion for improving pregnancy outcomes | Routine pre-pregnancy health promotion | 2 | 2 | 3 |
| Yakoob, M [56] | 2011 | BMC Public Health | The effect of providing skilled birth attendance and emergency obstetric care in preventing stillbirths. | Skilled birth attendants | 2 | 1 | 2 |

**Appendix 8 –** Table showing the different stages in study selection

| Stage | Criteria |
| --- | --- |
| Eligibility Criteria for selecting studies | (1) relevance to the SA government’s strategic priorities, policies and guidelines, and the local disease burden ;  (2) the intervention had to be implemented during the first 1 000 days, and  (3) articles were to consider low- to middle-income settings. Studies occurring in high-income countries, where study populations comprised of minority groups, immigrants or vulnerable populations comparable to the SA context, were included. |
| AMSTAR criteria | Quality assessment criteria were:   (1) size, (2) setting, (3) design, (4) methodological quality, (5) adequacy of allocation concealment, (6) blinding (of participants, providers of care, and investigators), (7) loss to follow-up, attrition, and exclusions.   This 11-point questionnaire was applied to all papers, and one score assigned per positive response. Only SRs which received an AMSTAR score of 11/11 were included. |
| Revised Bhutta criteria | The authors proposed three categories:  (1) evidence of impact on mortality and morbidity,  (2) evidence of impact on delivery and inclusion in PHC programmes, and  (3) cost-effectiveness.  Authors modified the ranking criteria by replacing cost-effectiveness with *effect on determinants of child health and behaviour change*.  For each of these categories, the study intervention was ranked for strength of evidence:  1 ‒ strong evidence of benefit in efficacy and effectiveness settings and recommended for inclusion in health systems;  2 ‒ moderate level of evidence of effect but recommended for inclusion; and  3 ‒ low level of evidence but recommended for further assessment  Only those articles that were assigned ratings of (1) ‘strong’ or (2) ‘moderate/plausible’ evidence were included. |
| Alignment to local policy criteria | Alignment was agreed upon if:  (1) the identified intervention was incorporated as an intervention of choice and the specifics thereof in the local documents matched that of the intervention identified in the review; and  (2) when the level of detail made available in key documents was sufficient to enable the end-user to implement the intervention (e.g. guideline, step-by-step instructions, standard operating procedures) (Figure1). |

**Appendix 9 -** Table of excluded studies

| **Author** | **Year** | **Journal** | **Title** |
| --- | --- | --- | --- |
| Abbott | 2013 | BMC Public Health | Improving immunisation timeliness in Aboriginal children through personalised calendars |
| Abdulwadud | 2012 | Cochrane Database | Interventions in the workplace to support breastfeeding for women in employment |
| Adetifa | 2009 | Cochrane Database | Iron supplementation for reducing morbidity and mortality in children with HIV. |
| Alexander | 2010 | Cochrane Database | Repeat digital cervical assessment in pregnancy for identifying women at risk of preterm labour (Review) |
| Allen | 2010 | Cochrane Database | Probiotics for treating acute infectious diarrhoea |
| Aluisio | 2013 | Pediatrics | Vitamin D supplementation and childhood diarrhoea: a randomized controlled trial. |
| Anene | 2013 | Rural Remote Health | Mobile primary healthcare services and health outcomes of children in rural Namibia. |
| Aponte | 2013 | Cochrane Database | Probiotics for treating persistent diarrhoea in children |
| Arifeen | 2009 | Lancet | Effect of the Integrated Management of Childhood Illness strategy on childhood mortality and nutrition in a rural area in Bangladesh: a cluster randomised trial. |
| Arrowsmith | 2010 | Cochrane Database | Strategies for improving the acceptability and acceptance of the copper intrauterine device (Review) |
| Askie | 2007 | Lancet | Antiplatelet agents for prevention of pre-eclampsia: a meta-analysis of individual patient data. |
| Austin | 2008 | Cochrane Database | Antenatal psychosocial assessment for reducing perinatal mental health morbidity |
| Awoonor-Williams | 2013 | BMC Health Serv Res | The Ghana essential health interventions program: a plausibility trial of the impact of health systems strengthening on maternal & child survival. |
| Azad | 2010 | Lancet | Effect of scaling up women's groups on birth outcomes in three rural districts in Bangladesh: a cluster-randomised controlled trial. |
| Bain | 2012 | Cochrane Database | Different magnesium sulphate regimens for neuroprotection of the fetus for women at risk of preterm birth |
| Bamigboye | 2009 | Cochrane Database | Oestrogen supplementation, mainly diethylstilbestrol, for preventing miscarriage |
| Baqui | 2008 | Bull World Health Organ | Impact of an integrated nutrition and health programme on neonatal mortality in rural northern India. |
| Barlow | 2010 | Cochrane Database | Group-based parent-training programmes for improving emotional and behavioural adjustment in children from birth to three years old |
| Becker | 2011 | Cochrane Database | Methods of milk expression for lactating women (Review) |
| Begley | 2011 | Cochrane Database | Active versus expectant management for women in the third stage of labour. |
| Bennet | 2013 | Cochrane Database | Massage for promoting mental and physical health in typically developing infants under the age of six months |
| Bernaola | 2013 | Cochrane Database | Probiotics for treating persistent diarrhoea in children |
| Bessell | 2011 | Cochrane Database | Feeding interventions for growth and development in infants with cleft lip, cleft palate or cleft lip and palate |
| Bhandari | 2003 | Lancet | Effect of community-based promotion of exclusive breastfeeding on diarrhoeal illness and growth: a cluster randomised controlled trial. |
| Bhutta | 2011 | BMC Public Health | Neonatal resuscitation and immediate newborn assessment and stimulation for the prevention of neonatal deaths: a systematic review, meta-analysis and Delphi estimation of mortality effect. |
| Bigirwa | 2009 | Department of Health Sciences of Uganda Martyrs University | Effectiveness of community health workers (CHWS) in the provision of basic preventive and curative maternal, newborn and child health (MNCH) interventions: A systematic review |
| Bin Nisar | 2011 | Resuscitation | Impact of essential surgical skills with an emphasis on emergency maternal, neonatal and child health training on the practice of doctors: a cluster randomised controlled trial in Pakistan. |
| Black | 2013 | Med J Aust | Health outcomes of a subsidised fruit and vegetable program for Aboriginal children in northern New South Wales |
| Boisson | 2013 | PLOS Med | Effect of household-based drinking water chlorination on diarrhoea among children under five in Orissa, India: a double-blind randomised placebo-controlled trial. |
| Boo | 2007 | J Paediatr Child Health | Short duration of skin-to-skin contact: effects on growth and breastfeeding. |
| Bredemeyer | 2012 | Cochrane Database | Body positioning for spontaneously breathing preterm infants with apnoea |
| Brion | 2003 | Cochrane Database | Vitamin E supplementation for prevention of morbidity and mortality in preterm infants |
| Brocklehurst | 2011 | Cochrane Database | Interventions for reducing the risk of mother-to-child transmission of HIV infection (Review) |
| Brodribb | 2013 | Cochrane Database | Postpartum health professional contact for improving maternal and infant health outcomes for healthy women and their infants |
| Brown | 2004 | Acta Paediatr | Vitamin A for acute respiratory infection in developing countries: a meta-analysis. |
| Brown | 2009 | Cochrane Database | Early postnatal discharge from hospital for healthy mothers and term infants |
| Brusamento | 2012 | Cochrane Database | Male involvement for increasing the effectiveness of prevention of mother-to-child HIV transmission (PMTCT) programmes |
| Bryanton | 2013 | Cochrane Database | Postnatal parental education for optimizing infant general health and parent-infant relationships |
| Bundy | 2013 | Acad Pediatr | Improving immunization delivery using an electronic health record: the ImmProve project. |
| Campbell | 2011 | BMC public health | Behavioural interventions for weight management in pregnancy: a systematic review of quantitative and qualitative data. |
| Car | 2011 | Cochrane Database | Integrating prevention of mother-to-child HIV transmission (PMTCT) programmes with other health services for preventing HIV infection and improving HIV outcomes in developing countries |
| Carelsen | 2013 | Am J Clin Nutr | Telephone-based support prolongs breastfeeding duration in obese women: a randomized trial. |
| Carfoot | 2005 | Midwifery | A randomised controlled trial in the north of England examining the effects of skin-to-skin care on breast feeding. |
| Carroli | 2001 | The Lancet | WHO systematic review of randomised controlled trials of routine antenatal care |
| Chamberlain | 2013 | Cochrane Database | Psychosocial interventions for supporting women to stop smoking in pregnancy |
| Chi | 2007 | Lancet | Single-dose tenofovir and emtricitabine for reduction of viral resistance to non-nucleoside reverse transcriptase inhibitor drugs in women given intrapartum nevirapine for perinatal HIV prevention: an open-label randomised trial |
| Churchill | 2010 | Cochrane Database | Interventionist versus expectant care for severe preeclampsia before term |
| Clasen | 2010 | Cochrane Database | Interventions to improve disposal of human excreta for preventing diarrhoea |
| Cluver | 2012 | Cochrane Database | Interventions for helping to turn term breech babies to head first presentation when using external cephalic version. |
| Coleman | 2012 | Cochrane Database | Pharmacological interventions for promoting smoking cessation during pregnancy. |
| Coren | 2010 | Cochrane Database | Parent training support for intellectually disabled parents |
| Cotter | 2001 | Cochrane Database | Prophylactic oxytocin for the third stage of labour. |
| Crowley | 2003 | Cochrane Database | Prophylactic corticosteroids for preterm birth |
| Crowther | 2010 | Cochrane Database | Vitamin K prior to preterm birth for preventing neonatal periventricular haemorrhage |
| Cupples | 2011 | Arch Dis Child | A RCT of peer-mentoring for first-time mothers in socially disadvantaged areas (the MOMENTS Study). |
| Dangour | 2013 | Cochrane Database | Interventions to improve water quality and supply, sanitation and hygiene practices, and their effects on the nutritional status of children. |
| Daniels | 2013 | Pediatrics. | Outcomes of an early feeding practices intervention to prevent childhood obesity. |
| Daniels | 2012 | Int J Obes (Lond) | Evaluation of an intervention to promote protective infant feeding practices to prevent childhood obesity: outcomes of the NOURISH RCT at 14 months of age and 6 months post the first of two intervention modules. |
| Darlow | 2011 | Cochrane Database | Vitamin A supplementation to prevent mortality and short- and long-term morbidity in very low birthweight infants |
| Dawood | 2013 | Cochrane Database | Intravenous fluids for reducing the duration of labour in low risk nulliparous women |
| Debes | 2013 | BMC Public Health | Time to initiation of breastfeeding and neonatal mortality and morbidity: a systematic review. |
| Demicheli | 2005 | Cochrane Database | Vaccines for women to prevent neonatal tetanus. |
| Dempsey | 2010 | Cochrane Database | Banked preterm versus banked term human milk to promote growth and development in very low birth weight infants |
| Dennis | 2008 | Cochrane Database | Oestrogens and progestins for preventing and treating postpartum depression |
| Dennis | 2013 | Cochrane Database | Interventions (other than pharmacological, psychosocial or psychological) for treating antenatal depression |
| De-Regill | 2013 | Evid Based Child Health | Home fortification of foods with multiple micronutrient powders for health and nutrition in children under two years of age (Review). |
| Dewey | 2008 | Maternal & Child Nutrition | Systematic review of the efficacy and effectiveness of complementary feeding interventions in developing countries. |
| Dodd | 2004 | Cochrane Database | Treatment for women with postpartum iron deficiency anaemia. |
| Dodge | 2013 | Pediatrics | Randomized controlled trial of universal postnatal nurse home visiting: impact on emergency care. |
| De-Regil | 2010 | Cochrane Database | Intermittent iron supplementation for improving nutrition and development in children under 12 years of age |
| Dowswell | 2009 | Cochrane Database | Antenatal day care unites versus hospital admission for women with complicated pregnancy |
| Downe | 2013 | Cochrane Database | Routine vaginal examinations for assessing progress of labour to improve outcomes for women and babies at term. |
| Dror | 2012 | Paediatr Perinat Epidemiol | Interventions with vitamins B6, B12 and C in pregnancy. |
| Dugravier | 2013 | PLOS One | Impact of a manualized multifocal perinatal home-visiting program using psychologists on postnatal depression: the CAPEDP randomized controlled trial. |
| Duley | 2013 | Cochrane Database | Drugs for treatment of very high blood pressure during pregnancy. |
| Dyson | 2005 | Cochrane Database | Interventions for promoting the initiation of breastfeeding |
| Eames | 2013 | Cochrane Database | Metformin for women who are obese during pregnancy for improving maternal and infant outcomes |
| Earl | 2013 | Cochrane Database | Interventions for hyperthyroidism pre-pregnancy and during pregnancy |
| Edwards | 2013 | Breastfeeding and complementary food: randomized trial of community doula home visiting. | Breastfeeding and complementary food: randomized trial of community doula home visiting. |
| Findley | 2013 | Int J Womens Health | Comparison of high- versus low-intensity community health worker intervention to promote newborn and child health in Northern Nigeria |
| Flenady | 2013 | Cochrane Database | Prophylactic antibiotics for inhibiting preterm labour with intact membranes. |
| Flint | 2008 | Cochrane Database | Cup feeding versus other forms of supplemental enteral feeding for newborn infants unable to fully breastfeed |
| Fottrell | 2013 | JAMA Pediatr | The effect of increased coverage of participatory women's groups on neonatal mortality in Bangladesh: A cluster randomized trial. |
| French | 2004 | Cochrane Database | Antibiotic regimens for endometritis after delivery. |
| Furber | 2013 | Cochrane Database | Antenatal interventions for reducing weight in obese women for improving pregnancy outcome |
| Gagnon | 2007 | Cochrane Database | Individual or group antenatal education for childbirth or parenthood, or both |
| Garcés | 2012 | Acta Obstet Gynecol Scand. | Training traditional birth attendants on the WHO Essential Newborn Care reduces perinatal mortality. |
| Gaunekar | 2013 | Cochrane Database | Maintenance therapy with calcium channel blockers for preventing preterm birth after threatened preterm labour |
| Geelhoed | 2013 | BMC Health Serv Res | Integrated maternal and child health services in Mozambique: structural health system limitations overshadow its effect on follow-up of HIV-exposed infants. |
| Gill | 2011 | BMJ | Effect of training traditional birth attendants on neonatal mortality (Lufwanyama Neonatal Survival Project): randomised controlled study. |
| Gilmore | 2013 | BMC Public Health | Effectiveness of community health workers delivering preventive interventions for maternal and child health in low- and middle-income countries: a systematic review. |
| Glenton | 2013 | Cochrane Database | Factors that can influence the success of lay health worker programmes for maternal and child health |
| Gogia | 2011 | Indian Pediatr | Community based newborn care: a systematic review and meta-analysis of evidence: UNICEF-PHFI series on newborn and child health, India. |
| Gogia | 2011 | Cochrane Database | Vitamin A supplementation for the prevention of morbidity |
| Gogia | 2012 | Cochrane Database | Zinc supplementation for mental and motor development in children |
| Gordon | 2005 | Cochrane Database | Antibiotic regimens for suspected late onset sepsis in newborn infants |
| Greenland | 2013 | BMC Public Health | The context and practice of handwashing among new mothers in Serang, Indonesia: a formative research study. |
| Greenough | 2008 | Cochrane Database | Synchronized mechanical ventilation for respiratory support in newborn infants. |
| Gregorio | 2011 | Cochrane Database | Early versus Delayed Refeeding for Children with Acute Diarrhoea |
| Grilli | 2002 | Cochrane Database | Mass media interventions: effects on health services utilisation. |
| Grimwade | 2006 | Cochrane Database | Cotrimoxazole prophylaxis for opportunistic infections in children with HIV infection. |
| Grotto | 2003 | J Pediatr | Vitamin A supplementation and childhood morbidity from diarrhoea and respiratory infections: a meta-analysis. |
| Gülmezoglu | 2007 | Cochrane Database | Prostaglandins for preventing postpartum haemorrhage. |
| Hahn | 2002 | Cochrane Database | Reduced osmolarity oral rehydration solution for treating dehydration caused by acute diarrhoea in children |
| Hahn | 2008 | Cochrane Database | Hand washing for preventing diarrhoea |
| Haider | 2000 | Lancet | Effect of community-based peer counsellors on exclusive breastfeeding practices in Dhaka, Bangladesh: a randomised controlled trial |
| Han | 2013 | Cochrane Database | Different types of dietary advice for women with gestational diabetes mellitus |
| Hartling | 2006 | Cochrane Database | Oral versus intravenous rehydration fro treating dehydration due to gastroenteritis in children |
| Henderson-Smart | 2002 | Cochrane Database | Mechanical ventilation for newborn infants with respiratory failure due to pulmonary disease |
| Henderson‐Smart | 2010 | Cochrane Database | Methylxanthine treatment for apnoea in preterm infants |
| Hewitt | 2005 | Int J Evid Based Healthc | Nursing and midwifery management of hypoglycaemia in healthy term neonates |
| Hodnett | 2013 | Cochrane Database | Continuous support for women during childbirth |
| Hodnett | 2012 | Cochrane Database | Alternative versus conventional institutional settings for birth |
| Hodnett | 2011 | Cochrane Database | Folic acid supplementation for the prevention of anaemia in preterm neonates |
| Hofmeyer | 2007 | BJOG | Dietary calcium supplementation for prevention of pre-eclampsia and related problems: a systematic review and commentary. |
| Hofmeyr | 2012 | Cochrane Database | Cephalic version by postural management for breech presentation |
| Hofmeyr | 2012 | Cochrane Database | External cephalic version for breech presentation at  term. |
| Hofmeyr | 2013 | Cochrane Database | Uterine massage for preventing postpartum haemorrhage. |
| Homer | 2012 | Cochrane Database | Group versus conventional antenatal care for women. |
| Horvarth | 2009 | Cochrane Database | Interventions for preventing late postnatal mother-to-child transmission of HIV |
| Hovell | 2000 | BMJ | Effect of counselling mothers on their children's exposure to environmental tobacco smoke: randomised controlled trial. |
| Humphreys | 2010 | Cochrane Database | Prevention of diarrhoea in children with HIV infection or exposure to maternal HIV infection |
| Hussain | 2011 | BMC Public Health | Elective induction for pregnancies at or beyond 41 weeks of gestation and its impact on stillbirths: a systematic review with meta-analysis. |
| Hutton | 2006 | Cochrane Database | External cephalic version for breech presentation before term. |
| Imdad | 2013 | BMC Public Health | The effect of umbilical cord cleansing with chlorhexidine on omphalitis and neonatal mortality in community settings in developing countries: a meta-analysis. |
| Inglis | 2007 | Cochrane Database | Prophylactic antibiotics to reduce morbidity and mortality in ventilated newborn infants |
| Jaafar | 2012 | Cochrane Database | Separate care for new mother and infant versus rooming-in for increasing the duration of breastfeeding |
| Jacobs | 2013 | Cochrane Database | Cooling for newborns with hypoxic ischaemic encephalopathy |
| Jagannath | 2012 | Cochrane Database | Routine neonatal circumcision for the prevention of urinary tract infections in infancy |
| Jamieson | 2012 | Lancet | Maternal and infant antiretroviral regimens to prevent postnatal HIV-1 transmission: 48-week follow-up of the BAN randomised controlled trial. |
| Jeffrey | 2004 | Medical Education | The impact of evidence-based education on a perinatal capacity- building initiative in Macedonia |
| Karp | 2013 | Breastfeed Med | Breastfeeding initiation in the context of a home intervention to promote better birth outcomes. |
| Kassab | 2013 | Cochrane Database | Furosemide for transient tachypnoea of the newborn. |
| Kelly | 2013 | Cochrane Database | Outpatient versus inpatient induction of labour for improving birth outcomes |
| Kempe | 2013 | Am J Public Health | Population-based versus practice-based recall for childhood immunizations: a randomized controlled comparative effectiveness trial. |
| Kendrick | 2007 | Cochrane Database | Parenting interventions for the prevention of unintentional injuries in childhood |
| Kenyon | 2012 | BMC Pregnancy Childbirth | Evaluation of Lay Support in Pregnant women with Social risk (ELSIPS): a randomised controlled trial. |
| Khan | 2013 | BMC Public Health. | Interventions to reduce neonatal mortality from neonatal tetanus in low and middle income countries--a systematic review. |
| Khan | 2013 | Indian J Pediatr. | Impact of behavior change communication among pregnant women regarding neonatal care. |
| Kikuchi | 2013 | PROSPERO International prospective register of systematic reviews | Defining the continuum of care in maternal, newborn and child health: which linkages are effective at improving maternal and newborn outcomes in low and middle income countries? A systematic review of quantitative evidence |
| Kimani-Murage | 2013 | Trials | Effectiveness of personalised, home-based nutritional counselling on infant feeding practices, morbidity and nutritional outcomes among infants in Nairobi slums: study protocol for a cluster randomised controlled trial. |
| Kindra | 2011 | BMC Public health | Effect of nutritional supplementation of breastfeeding HIV positive mothers on maternal and child health: findings from a randomized controlled clinical trial |
| Kirkwood | 2013 | Lancet | Effect of the Newhints home-visits intervention on neonatal mortality rate and care practices in Ghana: a cluster randomised controlled trial |
| Kozuki | 2013 | BMC Public Health | The associations of birth intervals with small-for gestational- age, preterm, and neonatal and infant mortality: a meta-analysis. |
| Kramer | 2012 | Cochrane Database | Optimal duration of exclusive breastfeeding |
| Kristjansson | 2010 | Cochrane Database | School feeding for improving the physical and psychosocial health of disadvantaged students |
| Kumar | 2008 | Lancet | Effect of community-based behaviour change management on neonatal mortality in Shivgarh, Uttar Pradesh, India: a cluster-randomised controlled trial. |
| Kuo | 2009 | J Clin Nurs | Evaluating the effects of an Internet education programme on newborn care in Taiwan |
| Lassi | 2013 | BMC Public Health | Impact of education and provision of complementary feeding on growth and morbidity in children less than 2 years of age in developing countries: a systematic review. |
| Lassi | 2013 | Cochrane Database | Folic acid supplementation during pregnancy for maternal health and pregnancy outcomes |
| Lauzon | 2001 | Cochrane Database | Labour assessment programs to delay admission to labour wards |
| Lavender | 2013 | Cochrane Database | Telephone support for women during pregnancy and the first six weeks postpartum. |
| Lazzarini | 2013 | Cochrane Database | Oral zinc for treating diarrhoea in children |
| Lee | 2011 | BMC Public Health | Neonatal resuscitation and immediate newborn assessment and stimulation for the prevention of neonatal deaths: a systematic review, meta-analysis and Delphi estimation of mortality effect |
| Lee | 2008 | Cochrane Database | Antenatal breast examination for promoting breastfeeding |
| Lenters | 2013 | BMC Public Health | Systematic review of strategies to increase use of oral rehydration solution at the household level. |
| Lenters | 2013 | BMC Public Health | Treatment of severe and moderate acute malnutrition in low- and middle-income settings: a systematic review, meta-analysis and Delphi process. |
| Lewycka | 2010 | Trials | A cluster randomised controlled trial of the community effectiveness of two interventions in rural Malawi to improve health care and to reduce maternal, newborn and infant mortality |
| Lewycka | 2013 | Lancet | Effect of women's groups and volunteer peer counselling on rates of mortality, morbidity, and health behaviours in mothers and children in rural Malawi (MaiMwana): a factorial, cluster-randomised controlled trial. |
| Lopex | 2012 | Cochrane Database | Education for contraceptive use by women after birth |
| Lopez | 2012 | Cochrane Database | Education for contraceptive use by women after childbirth. |
| Lori | 2013 | Int J Gynaecol Obstet | Maternity waiting homes and traditional midwives in rural Liberia. |
| Lucas | 2008 | Cochrane Database | Financial benefits for child health and well being in low income or socially disadvantaged families in developed world countries. |
| Lui | 2008 | Cochrane Database | Psychosocial interventions for women enrolled in alcohol treatment during pregnancy |
| Lumley | 2009 | Cochrane Database | Interventions for promoting smoking cessation during pregnancy |
| Luoto | 2010 | Br J Nutr | Impact of maternal probiotic-supplemented dietary counselling on pregnancy outcome and prenatal and postnatal growth: a double-blind, placebo-controlled study |
| Magee | 2013 | Cochrane Database | Prevention and treatment of postpartum hypertension |
| Magee | 2003 | Cochrane Database | Oral beta-blockers for mild to moderate hypertension during pregnancy. |
| Målqvist | 2013 | PLOS One | Targeted Interventions for Improved Equity in Maternal and Child Health in Low- and Middle-Income Settings: A Systematic Review and Meta-Analysis |
| Mayo-Wilson | 2011 | BMJ | Vitamin A supplements for preventing mortality, illness, and blindness in children aged under 5: systematic review and meta-analysis. |
| McCall | 2010 | Cochrane Database | Interventions to prevent hypothermia at birth in preterm and/or low birthweight infants |
| McDonald | 2013 | Cochrane Database | Effect of timing of umbilical cord clamping of term infants on maternal and neonatal outcomes |
| Mcqueen | 2013 | J Perinat Neonatal Nurs | Feasibility of a Caregiver-Assisted Exercise Program for Preterm Infants |
| Meher | 2005 | Cochrane Database | Bed rest with or without hospitalisation for hypertension during pregnancy |
| Mengistie | 2013 | PLOS One | Household water chlorination reduces incidence of diarrhoea among under-five children in rural Ethiopia: a cluster randomized controlled trial. |
| Miller | 2013 | Cochrane Database | Dietary supplements for preventing postnatal depression |
| Miller | 2011 | Cochrane Database | Home based child development interventions for preschool children from socially disadvantaged families |
| Minozzi | 2008 | Cochrane Database | Maintenance agonist treatments for opiate dependent pregnant women (Review) |
| Mohan | 2004 | BMJ | Impact of counselling on care seeking behaviour in families with sick children: cluster randomised trial in rural India. |
| Monterrosa | 2013 | J Nutr | Scripted messages delivered by nurses and radio changed beliefs, attitudes, intentions, and behaviors regarding infant and young child feeding in Mexico. |
| Montgomery | 2009 | Cochrane Database | Media-based behavioural treatments for behavioural problems in children. |
| Moore | 2012 | BMC Pregnancy Childbirth | A randomized trial to investigate the effects of pre-natal and infant nutritional supplementation on infant immune development in rural Gambia: the ENID trial: Early Nutrition and Immune Development |
| Morrow | 2000 | Lancet | Efficacy of home-based peer counselling to promote exclusive breastfeeding: a randomised controlled trial. |
| Motaze | 2013 | Cochrane Database | Treatment interventions for diarrhoea in HIV‐infected and HIV‐exposed children |
| Mousa | 2007 | Cochrane Database | Treatment for primary postpartum haemorrhage. |
| Mtitimila | 2004 | Cochrane Database | Antibiotic regimens for suspected early neonatal sepsis |
| Muktabhant | 2012 | Cochrane Database | Interventions for preventing excessive weight gain during pregnancy |
| Musekiwa | 2011 | Cochrane Database | Oral rehydration salt solution for treating cholera: ≤ 270 mOsm/L solutions vs ≥ 310 mOsm/L solutions |
| Myer | 2003 | Sex Transm Infect | Impact of on-site testing for maternal syphilis on treatment delays, treatment rates, and perinatal mortality in rural South Africa: a randomised controlled trial |
| Neilson | 2009 | Cochrane Database | Symphysis-fundal height measurement in pregnancy |
| Newnham | 2009 | Infant Behav Dev | Effectiveness of a modified Mother-Infant Transaction Program on outcomes for preterm infants from 3 to 24 months of age |
| Nicolson | 2013 | Arch Womens Ment Health | Supporting the adolescent mother-infant relationship: preliminary trial of a brief perinatal attachment intervention. |
| Nissensohn | 2013 | Matern Child Nutr | Effect of zinc intake on serum/plasma zinc status in infants: a meta-analysis. |
| O'Donnell | 2004 | Cochrane Database | Positive end-expiratory pressure for resuscitation of newborn infants at birth. |
| Ohlsson | 2013 | Cochrane Database | Intravenous immunoglobulin for preventing infection in preterm and/or low birth weight infants |
| Oken | 2008 | International Journal of Obesity | Maternal smoking during pregnancy and child overweight: systematic review and meta-analysis |
| Okoromah | 2004 | Cochrane Database | Antifungal agents for the treatment of mucocutaneous candidiasis in neonates and children |
| Olayemi | 2011 | Cochrane Database | Parenteral opioids for maternal pain relief in labour: |
| Oloyede | 2012 | Cochrane Database | Specialized antenatal clinics for women with a pregnancy at high risk of preterm birth (excluding multiple pregnancy) to improve maternal and infant outcomes |
| Opiyo | 2010 | Cochrane Database | In-service training for health professionals to improve care of the seriously ill newborn or child in low and middle-income countries |
| Oude Luttikuis | 2009 | Cochrane Database | Interventions for treating obesity in children |
| Panpaniach | 2009 | Cochrane Database | Growth monitoring in children |
| Parab | 2013 | Cochrane Database | Specialist home-based nursing services for children with acute and chronic illnesses |
| Pattinson | 2009 | Cochrane Database | Critical incident audit and feedback to improve perinatal and maternal mortality and morbidity |
| Prendiville | 2000 | Cochrane Database | Active versus expectant management in the third stage of labour (Review). |
| Priest | 2008 | Cochrane Database | Policy interventions implemented through sporting organisations for promoting healthy behaviour change |
| Priest | 2008 | Cochrane Database | Interventions implemented through sporting organisations for increasing participation in sport |
| Prost | 2013 | Lancet | Women’s groups practising participatory learning and action to improve maternal and newborn health in low-resource settings: a systematic review and meta-analysis |
| Radhakrishna | 2013 | Plos One | Effectiveness of zinc supplementation to full term normal infants: a community based double blind, randomized, controlled, clinical trial. |
| Rahman | 2013 | Bull World Health Organ | Interventions for common perinatal mental disorders in women in low and middle-income countries: a systematic review and meta-analysis |
| Ramakrishnan | 2012 | Paediatr Perinat Epidemiol | Effect of women's nutrition before and during early pregnancy on maternal and infant outcomes: a systematic review. |
| Ramsey | 2013 | BMC Health Serv Res | The Tanzania Connect Project: a cluster-randomized trial of the child survival impact of adding paid community health workers to an existing facility-focused health system |
| Read | 2005 | Cochrane Database | Efficacy and safety of caesarean delivery for prevention of mother‐to‐child transmission of HIV‐1 |
| Reid | 2012 | Cochrane Database | Interventions for clinical and subclinical hypothyroidism in pregnancy |
| Reifsnider | 2013 | BMC Public Health | A randomized controlled trial to prevent childhood obesity through early childhood feeding and parenting guidance: rationale and design of study. |
| Renfrew | 2012 | Cochrane Database | Support for healthy breastfeeding mothers with healthy term babies |
| Reynolds | 2010 | East Afr Med J. | Cluster randomised trial of the uptake of a take-home infant dose of nevirapine in Kenya. |
| Roberts | 2006 | Cochrane Database | Antenatal corticosteroids for accelerating fetal lung maturation for women at risk of preterm birth |
| Robertson | 2013 | Lancet | Effects of unconditional and conditional cash transfers on child health and development in Zimbabwe: a cluster-randomised trial. |
| Rosen | 2012 | Pediatrics | Parental Smoking Cessation to Protect Young Children: A Systematic Review and Meta-analysis |
| Ruiz | 2013 | Mayo Clin Proc | Supervised exercise-based intervention to prevent excessive gestational weight gain: a randomized controlled trial. |
| Sado | 2012 | Cochrane Database | Hypnosis during pregnancy, childbirth, and the postnatal period for preventing postnatal depression |
| Santos | 2013 | Nutr Hosp | Impact of an intervention nutrition program during prenatal on the weight of newborns from teenage mothers. |
| Say | 2009 | Cochrane Database | Calcium channel blockers for potential impaired fetal growth |
| Sazawal | 2003 | Lancet Infect Dis | Effect of pneumonia case management on mortality in neonates, infants, and preschool children: a meta-analysis of community-based trials |
| Schoetzau | 2002 | Arch Dis Child | Maternal compliance with nutritional recommendations in an allergy preventive programme. |
| Shaw | 2006 | Birth | Systematic review of the literature on postpartum care: effectiveness of postpartum support to improve maternal parenting, mental health, quality of life, and physical health |
| Shrimpton | 2009 | Food Nutr Bull | Multiple micronutrient supplementation during pregnancy in developing-country settings: policy and program implications of the results of a meta-analysis. |
| Siegfried | 2012 | Cochrane Database | Micronutrient supplementation in pregnant women with HIV infection |
| Sinclair | 2011 | Cochrane Database | Oral vaccines for preventing cholera |
| Soares-Weiser | 2004 | Cochrane Database | Rotavirus vaccine for preventing diarrhoea |
| Soares‐Weiser | 2012 | Cochrane Database | Vaccines for preventing rotavirus diarrhoea: vaccines in use |
| Soll | 2009 | Cochrane Database | Multiple versus single doses of exogenous surfactant for the prevention or treatment of neonatal respiratory distress syndrome. |
| Soltani | 2010 | Cochrane Database | Timing of prophylactic uterotonics for the third stage of labour after vaginal birth. |
| Spinelli | 2013 | Breastfeed Med | Increased breastfeeding rates in black women after a treatment intervention. |
| Stade | 2009 | Cochrane Database | Psychological and/or educational interventions for reducing alcohol consumption in pregnant women and women planning pregnancy |
| Steiner | 2003 | Obstet Gynecol | Understanding risk: a randomized controlled trial of communicating contraceptive effectiveness. |
| Sturt | 2010 | Cochrane Database | Antiretroviral therapy (ART) for treating HIV infection in ART-eligible pregnant women |
| SUMMIT study group (Shankar) | 2008 | Lancet | Effect of maternal multiple micronutrient supplementation on fetal loss and infant death in Indonesia: a double-blind cluster-randomised trial |
| Sunguya | 2013 | Nutr J | Effectiveness of nutrition training of health workers toward improving caregivers' feeding practices for children aged six months to two years: a systematic review. |
| Tahir | 2013 | Int J Nurs Stud | Does telephone lactation counselling improve breastfeeding practices? A randomised controlled trial |
| Tan | 2012 | Cochrane Database | Maternal glucose administration for facilitating tests of fetal wellbeing |
| Tan | 2005 | Cochrane Database | Clinical decision support systems for neonatal care |
| Tan | 2005 | Cochrane Database | Air versus oxygen for resuscitation of infants at birth. |
| Tang | 2012 | Cochrane Database | Hormonal and intrauterine methods for contraception for women aged 25 years and younger |
| Thangaratinam | 2012 | BMJ | Effects of interventions in pregnancy on maternal weight and obstetric outcomes: meta-analysis of randomised evidence |
| Thaver | 2009 | Cochrane Database | The effect of community health educational interventions on newborn survival in developing countries |
| Thorne-Lyman | 2013 | Paediatr Perinat Epidemiol | Vitamin A and carotenoids during pregnancy and maternal, neonatal and infant health outcomes: A systematic review and meta-analysis |
| Tieu | 2010 | Cochrane Database | Oral anti-diabetic agents for women with pre-existing diabetes mellitus/impaired glucose tolerance or previous gestational diabetes mellitus |
| Tripathy | 2010 | The Lancet | Effect of a participatory intervention with women's groups on birth outcomes and maternal depression in Jharkhand and Orissa, India: a cluster-randomised controlled trial. |
| Tudor Car | 2011 | Cochrane Database | Integrating prevention of mother to child HIV transmission programmes with other health services |
| Tuncalp | 2012 | Cochrane Database | Prostaglandins for preventing postpartum haemorrhage. |
| Turab | 2013 | BMC Pregnancy Childbirth | Improved accessibility of emergency obstetrics and newborn care(EmONC) services for maternal and newborn health: a community based project |
| Turab | 2013 | BMC Pregnancy Childbirth | Improved accessibility of emergency obstetrics and newborn care (EmONC) services for maternal and newborn health: a community based project. |
| Turnball | 2012 | Cochrane Database | Home visits during pregnancy and after birth for women with an alcohol or drug problem |
| Ullman | 2010 | Cochrane Database | Parenteral opioids for maternal pain management in labour |
| Ungerer | 2004 | Cochrane Database | Prophylactic versus selective antibiotics for term newborn infants of mothers with risk factors for neonatal infection |
| Utama | 2012 | Cochrane Database | Transplacental versus direct fetal corticosteroid treatment for accelerating fetal lung maturation |
| Uwimana | 2013 | Int J Tuberc Lung Dis | Community-based intervention to enhance provision of integrated TB-HIV and PMTCT services in South Africa |
| van Lonkhuijzen | 2012 | Cochrane Database | Maternity waiting facilities for improving maternal and neonatal outcome in low-resource countries |
| Verkerk | 2012 | Early Hum Dev | The infant behavioral assessment and intervention program in very low birth weight infants; outcome on executive functioning, behaviour and cognition at preschool age. |
| Vesel | 2013 | Trop Med Int Health | Promoting skin-to-skin care for low birthweight babies: findings from the Ghana. Newhints cluster-randomised trial. |
| Villar | 2001 | Cochrane Database | Patterns of routine antenatal care for low-risk pregnancy. |
| Wade | 2006 | BMC Pregnancy and Childbirth | Behaviour change in perinatal care practices among rural women exposed to a women’s group intervention in Nepal. |
| Waiswa | 2012 | Trials | The Uganda Newborn Study (UNEST): an effectiveness study on improving newborn health and survival in rural Uganda through a community-based intervention linked to health facilities - study protocol for a cluster randomized controlled trial |
| Walker | 2001 | Cochrane Database | Antibiotics for syphilis diagnosed during pregnancy. |
| Waters | 2011 | Cochrane Database | Interventions for preventing obesity in children |
| Webster | 2009 | Cochrane Database | Gowning by attendants and visitors in newborn nurseries for prevention of neonatal morbidity and mortality |
| Welch | 2012 | BMC Pediatr | Family nurture intervention (FNI): methods and treatment protocol of a randomized controlled trial in the NICU |
| Welch | 2013 | BMC Pediatr | Randomized controlled trial of Family Nurture Intervention in the NICU: assessments of length of stay, feasibility and safety. |
| Westhoff | 2013 | Cochrane Database | Prophylactic oxytocin for the third stage of labour to prevent postpartum haemorrhage |
| Whitworth | 2012 | Cochrane Database | Routine pre-pregnancy health promotion for improving pregnancy outcomes |
| Wiysonge | 2005 | Cochrane Database | Vaginal disinfection for preventing mother‐to‐child transmission of HIV infection |
| Yonemoto | 2013 | Cochrane Database | Schedules for home visits in the early postpartum period. |
| Zaidi | 2009 | Cochrane Database | Community based management of neonatal sepsis in developing countries |
| Zoritch | 2009 | Cochrane Database | Day care for pre-school children |
| Zupan | 2004 | Cochrane Database | Topical umbilical cord care at birth. |

**Search String**

The search was designed to identify all pharmacological (prevention and treatment) and non-pharmacological (behaviours, approach or practices) that aimed to improve maternal, neonatal and child health outcomes during the first 1 000 days, and which also occurred at primary health care level with a focus on LMICs or vulnerable populations in HICs. Medline was searched using the following search string: ‘maternal’ [Medical Subject Headings (MeSH)] *or* ‘mother’ [MeSH] or ‘women’ [MeSH] ‘newborn’ [MeSH*]* *or* ‘neonatal’ [MeSH] *or* ‘child’ [MeSH] ‘infant’ [MeSH] *or* ‘intervention’ [MeSH]. Search terms specific to the 56 previously identified interventions, such as ‘family planning’ or ‘birth spacing’, ‘hypertension’, and ‘folate supplementation’ were combined with the previous search terms. Intervention type and terms specific to platforms or levels of health-system delivery were also used to expand the search and to identify studies conducted in relevant health-system settings, such as ‘community based’ and ‘primary health care’.

These are both seen in the following search string: (‘mothers’[MeSH Terms] *or* ‘mothers’[All Fields] *or* ‘maternal’[All Fields]) and (‘Mother Child’[Journal] or (‘mother’[All Fields] and ‘child’[All Fields]) *or* ‘mother child’[All Fields]) *and* (‘infant’[MeSH Terms] *or* ‘infant’[All Fields]) *and* (‘infant, newborn’[MeSH Terms] *or* (‘infant’[All Fields] *and* ‘newborn’[All Fields]) *or* ‘newborn infant’[All Fields] *or* ‘neonate’[All Fields]) *and* (‘infant, newborn’[MeSH Terms] *or* (‘infant’[All Fields] *and* ‘newborn’[All Fields]) *or* ‘newborn infant’[All Fields] *or* ‘newborn’[All Fields]) *or* ‘intervention’[All Fields] *and* ((‘family planning’[All Fields] *or* ‘sexually transmitted infections’ [All Fields] *or* ‘antiretroviral medicines’ [All Fields] *or* ‘folic acid supplementation’ [All Fields] *or* ‘iron supplementation’ [All Fields] *or* ‘tetanus vaccination’ [All Fields] *or* ‘calcium supplementation’ [All Fields] *or* ‘smoking cessation’ [All Fields] *or* ‘low dose aspirin’ [All Fields] *or* ‘hypertension’ [All Fields] *or* ‘magnesium sulphate’ [All Fields] *or* ‘antibiotics’ [All Fields] *or* ‘corticosteroids’[All Fields] ‘abortion’ [All Fields] ‘contraception’ [All Fields] ‘External Cephalic Version’ [All Fields] ‘labour’ [All Fields] *or* ‘Prophylactic uterotonics’[All Fields] *or* ‘uterine massage’ [All Fields] *or* ‘postpartum haemorrhage’ [All Fields] *or* ‘HIV’ [All Fields] *or* ‘Caesarean section’ [All Fields] *or* ‘prophylactic antibiotics’ [All Fields] *or* ‘nutrition counselling’ [All Fields] *or* ‘postpartum sepsis’ [All Fields] *or* ‘thermal care’ [All Fields] *or* ‘breastfeeding’ [All Fields] *or* ‘umbilical cord’ [All Fields] *or* ‘neonatal resuscitation’ [All Fields] *or* ‘Kangaroo Mother Care’ [All Fields] *or* ‘preterm babies’ [All Fields] *or* ‘jaundice’ [All Fields] *or* ‘antiretroviral therapy’ [All Fields] *or* ‘surfactant’ [All Fields] *or* ‘neonatal sepsis’ [All Fields] *or* ‘pneumonia’ [All Fields] *or* ‘Vitamin A supplementation’ [All Fields] *or* ‘immunisation’ [All Fields] *or* ‘malnutrition’ [All Fields] *or* ‘diarrhoea’ [All Fields] *or* ‘home visits’ [All Fields] *or* ‘community-based’ [All Fields] *or* ‘women’s groups’ [All Fields] *or* ‘primary health care’ [All Fields] *or* ‘1 000 days’ [All Fields] or LMIC [All Fields]))

For CINAHL, Scielo, Google Scholar and WHORHL the search terms were ‘maternal’, ‘newborn’, ‘child’, ‘interventions’, ‘1 000 days’, ‘primary health’, and the 56 interventions mentioned in the Medline search

1. Mwansa-Kambafwile J, Cousens S, Hansen T, Lawn JE. Antenatal steroids in preterm labour for the prevention of neonatal deaths due to complications of preterm birth. Int J Epidemiol. 2010; 39(1):i122‒i133.
2. Imdad A, Yakoob MY, Bhutta ZA. The effect of folic acid, protein energy and multiple micronutrient supplements in pregnancy on stillbirths. BMC Public Health. 2011: 11(3):S4.
